# Design and Synthesis of Novel Anti-Proliferative Emodin Derivatives and Studies on their Cell Cycle Arrest, Apoptosis Pathway and Migration

**DOI:** 10.3390/molecules24050884

**Published:** 2019-03-02

**Authors:** Kun Yang, Ming-Ji Jin, Zhe-Shan Quan, Hu-Ri Piao

**Affiliations:** 1 Key Laboratory of Natural Resources and Functional Molecules of the Changbai Mountain, Affiliated Ministry of Education; Yanbian University College of Pharmacy, Yanji 133002, Jilin Province, China; yangkun815@163.com; 2 Institute of Materia Medica, Chinese Academy of Medical Sciences, Beijing 100050, China; mingji612@126.com

**Keywords:** emodin derivatives, amino acids, anti-proliferative activity, HepG2 cells, MCF-7 cells

## Abstract

Emodin is a cell arrest and apoptosis-inducing compound that is widely distributed in different plants (rhubarb, aloe), lichens and terrestrial fungi, and also isolated from marine-derived fungi and marine sponge-associated fungi. In this study, we designed and synthesized a novel series of emodin derivatives by binding emodin to an amino acid using linkers of varying lengths and composition, and evaluated their anti-proliferative activities using HepG2 cells (human hepatic carcinoma), MCF-7 cells (human breast cancer) and human normal liver L02 cells. Most of these derivatives showed moderate to potent anti-proliferative activities. Notably, compound **7a** exhibited potent anti-proliferative activity against HepG2 cells with the half maximal inhibitory concentration (IC_50_) value of 4.95 µM, which was enhanced 8.8-fold compared to the parent compound emodin (IC_50_ = 43.87 µM), and it also exhibited better selective anti-proliferative activity and specificity than emodin. Moreover, further experiments demonstrated that compound **7a** displayed a significant efficacy of inducing apoptosis through mitochondrial pathway via release of cytochrome c from mitochondria and subsequent activation of caspase-9 and caspase-3, inducing cell arrest at G0/G1 phase, as well as suppression of cell migration of tumor cells. The preliminary results suggested that compound **7a** could be a promising lead compound for the discovery of novel anti-tumor drugs and has the potential for further investigations as an anti-cancer drug.

## 1. Introduction

**Figure 1:**
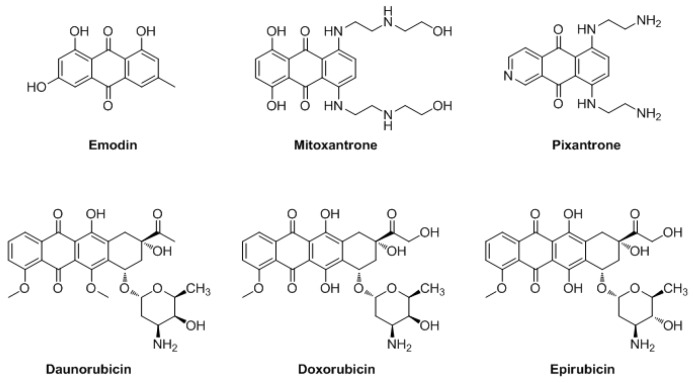
Structures of emodin and antitumor drugs with anthraquinone skeleton.

Emodin (3-methyl-1,6,8-trihydroxyanthraquinone, Figure 1) is a naturally occurring anthraquinone derivative that is widely distributed in different plants (rhubarb, aloe), lichens and terristrial fungi, that has also been isolated from marine-derived fungi and marine sponge-associated fungi [1,2,3,4]. It is also an active ingredient in Chinese medicinal herbs used in the treatment of constipation, jaundice, gastro-intestinal hemorrhage, and ulcers. Emodin has the same tricyclic planar chromophore skeleton as certain anti-tumor antibiotics such as daunorubicin, doxorubicin, epirubicin, and several synthetic analogs distributed such as mitoxantrone and pixantrone (Figure 1); all of which are in used for clinical treatment of various cancers. Many reports have demonstrated that emodin possesses a wide spectrum of pharmacological effects such as anti-tumour [5,6,7], anti-inflammatory [8,9], antiviral [10], antibacterial [11], anti-allergic [12], anti-osteoporotic [13], anti-diabetic [14,15], immunosuppressive [16], neuroprotective [17], and hepatoprotective [18,19] activities, etc. Among these effects, anti-tumor activity is the most widely reported. In recent decades, pharmacological studies have shown that emodin is capable of inhibiting cellular proliferation [20], inducing of cell differentiation [21] and apoptosis [22], and activating of caspase cascade pathway [23,24] and mitochondrial death pathway [25,26] in different cancer cells.

Chemical transformation of bioactive compounds of natural products is one of the most common approaches in drug discovery to improve therapeutic properties. To date, the anti-tumor activity of emodin has been improved by structural modification, mainly to the side chain including methyl, hydroxyl, and/or aryl ring groups. For example, Wang et al. reported that emodin quaternary ammonium salt derivatives showed significant anti-cancer activities against hepatoma cells [27,28,29]. Xing et al. reported the effects of emodin rhamnoside derivatives against human cancer cells [30], and Tan et al. reported DNA-binding pyrazole emodin derivatives [31].

**Figure 2:**
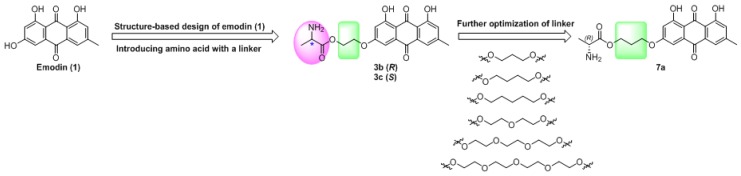
Design of emodin derivatives with varying lengths of diol linkers.

However, emodin is not an ideal chemotherapeutic agent for cancer due to its poor bioavailability, low solubility, and high toxicity in vivo. The bioavailability of a drug is positively correlated with its solubility; thus amino acids, which possess the carboxylic and amino functionality, are ideal as a moiety for the structural modification of bioactive natural products. Amino acids can interact with other biomolecules via secondary interactions such as hydrogen bonding to improve their pharmacological profiles in both potency and bioavailability [32]. For example, conjugation of an amino acid to betulinic acid is able to improve its water-solubility as well as its anti-melanoma activity [33]. These findings prompted us to design and synthesize a series of novel emodin derivatives linked with various amino acids as TFA salt (Figure 2).

Among these derivatives, compound **3b** derived from (*R*)-2-aminopropanoic acid exhibited the most anti-proliferative activity against both HepG2 cells and MCF-7 cells. This prompted us to screen the different length of diol linker between 2-aminopropanoic acid and emodin. Our results also suggest that compared to compound **3a**, compound **7a** exhibited a slightly more potent anti-proliferative activity against HepG2 cells and MCF-7 cells. Following this, the anti-cancer mechanism of compound **7a** was evaluated against HepG2 cells.

## 2. Results and Discussion

### 2.1. Chemistry

**Scheme 1:**
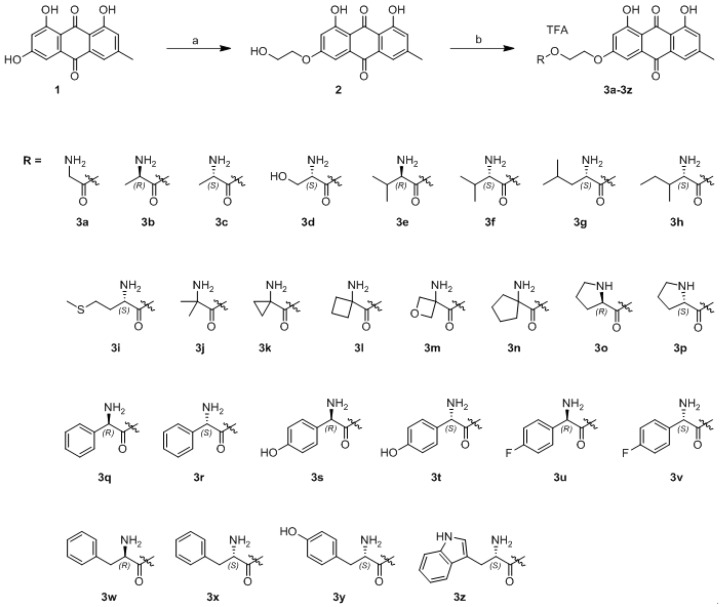
Synthesis of compounds **3a**–**3z**. *Reagents and conditions*: (**a**) 2-iodoethanol, Cs_2_CO_3_, DMF, 60 °C, 65%; (**b**) (i) various *N*-Boc amino acids, DCC, DCM, 0 °C; (ii) 20% TFA in DCM, r.t., 20%–50% over two steps.

**Scheme 2:**
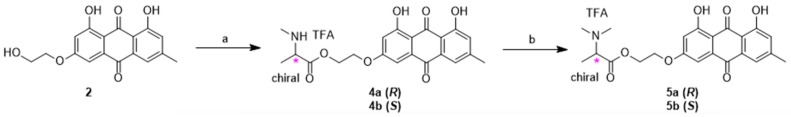
Synthesis of compounds **4a**, **4b**, **5a** and **5b**. *Reagents and conditions*: (**a**) (i) Boc-*N*-Me-*R*/S-Ala-OH, DCC, DCM, 0 °C; (ii) 20% TFA in DCM, r.t., 40%–50% over two steps; (**b**) formaldehyde (37% aqueous solution), NaBH_3_CN, MeOH, r.t., 85%–89%.

**Scheme 3:**
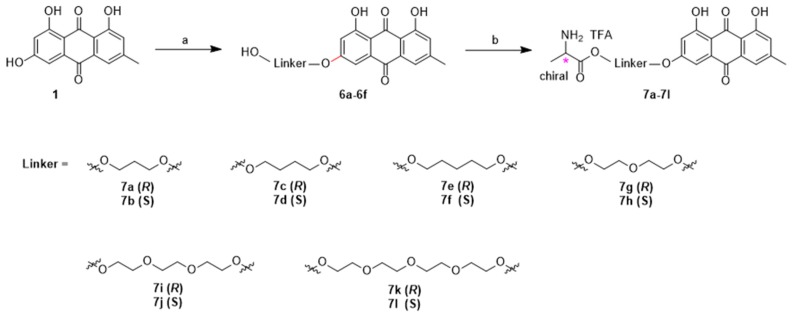
Synthesis of compounds **7a**–**7l**. *Reagents and conditions*: (**a**) various hydroxyalkyl bromides or iodides, Cs_2_CO_3_, DMF, 60 °C, 30%–55%; (**b**) (i) *R*/*S*-*N*-Boc-Ala-OH, DCC, DCM, 0 °C; (ii) 20% TFA in DCM, r.t., 65%–70% over two steps.

A novel series of emodin derivatives linked with amino acids were designed and synthesized. The synthesis strategies for these emodin derivatives are outlined in Scheme 1, Scheme 2 and Scheme 3. For the synthesis of compounds **3a**–**3z**, compound **1** was subjected to an alkylation reaction with 2-iodoethanol and Cs_2_CO_3_ in DMF that resulted in compound **2**. Then coupling reactions under the conventional coupling condition (DCC/DMAP) between compound **2** and various commercially available *N*-Boc protected amino acids resulted in the formation of condensation products. After removal of the protecting group usinng 20% TFA in DCM, the target compounds **3a**–**3z** were obtained as TFA salts.

Following the DCC/DMAP coupling condition and deprotection under 20% TFA in DCM for the synthesis of compounds **3a**–**3z**, the target compounds **4a** and **4b** were obtained as TFA salts (Scheme 2). 

These products were then coupled with formaldehyde (37% aqueous solution) and reduced utilizing NaBH_3_CN to provide **5a** and **5b** as TFA salt in 85% and 89% yields, respectively. As shown in Scheme 3, by employing the route for the synthesis of compounds **3a**–**3z**, compounds **7a**–**7l** were obtained as TFA salts.

### 2.2. Biological Activity against Cancer Cell Lines

The in vitro anti-proliferative activities of all the novel synthesized compounds were evaluated against HepG2 cells and MCF-7 cells by CellTiter-Glo^®^ Luminescent Cell Viability Assay, using paclitaxel (a clinically used drug) as a positive control. Emodin (**1**) and compound **2** were also included for comparison. 

As shown in Table 1, emodin (**1**) showed weak inhibitory activity against HepG2 cells and MCF-7 cells with IC_50_ values of 43.87 ± 1.28 μM and 52.72 ± 2.22 μM, respectively. Compared to emodin (**1**), compound **2** showed reduced activity due to the introduction of the hydroxyethyl group at the 3-position of emodin. Among compounds **3a**–**3z** containing amino acid groups, it is worth noting that most of the compounds displayed more potent activity than emodin (**1**). Compound **3a** with the Gly group displayed near double the activity of emodin (**1**) against HepG2 cells, but had slightly decreased activity against MCF-7 cells.

Compound **3b** with the D-Ala group displayed the most potent anti-proliferative activity against HepG2 cells and MCF-7 cells with IC_50_ values of 8.22 ± 0.13 μM and 8.99 ± 0.64 μM, respectively. Compound **3c** with the L-Ala group displayed slightly weaker activity than compound **3b**, but still displayed stronger activity than emodin (**1**). Compounds **3d**–**3j** containing alkyl chain amino acid groups also displayed stronger inhibitory activities than emodin (**1**). Overall, with the exception of compound **3n**, compounds **3k**–**3m** with cyclic alkyl amino acid groups displayed worse inhibitory activities than emodin (**1**); especially compound **3m** containing a heterocycle. Interestingly, compound **3n** which contains a cyclopentane amino acid group displayed higher anti-proliferative activity against HepG2 cells and MCF-7 cells with IC_50_ values of 12.48 ± 0.59 μM and 23.51 ± 1.93 μM, respectively. Compounds **3o** and **3p** with D- and L-Ala groups exhibited similar inhibitory activity to compound **3n**. Compared to emodin (**1**), compounds **3q**–**3z** with aryl (phenyl, substituted phenyl, benzyl, substituted benzyl, etc.) amino acid groups displayed enhanced activity in varying degrees when tested against two cancer cell lines, with IC_50_ values ranging from 11.41 ± 0.77 to 42.44 ± 1.16 μM. 

Following our initial results, we performed further modification of compounds **3b** and **3c** to improve the anti-proliferative activity, thus mono- and dimethylation of the amino moiety in the amino acid afforded compounds **4a**, **4b**, **5a** and **5b**. Unfortunately, this modification led to decreased activity to varying degrees against both the HepG2 and MCF-7 cell lines, with IC_50_ values ranging from 13.69 ± 0.89 to 68.81 ± 2.25 μM, as shown in Table 2. Notably, compound **4b** displayed a 6-fold activity reduction against MCF-7 cell lines compared to emodin (**1**). The results revealed that “NH_2_” was the moiety that favors improving the anti-proliferative activity. The preliminary explanation was that amino group can interact with other biomolecules to improve the anti-proliferative activity via formation of hydrogen bond. Mono- or dimethylation of amino group might weaken the trend of hydrogen bonding and decrease the anti-proliferative activity.

Next, as shown in Table 3, the linking group of compounds **3b** and **3c** was further optimized by increasing the linker length in compounds **3b** and **3c** by one additional methylene group. The resulting compounds, **7a** and **7b**, displayed further enhanced potency at inhibiting cell growth against both HepG2 cells and MCF-7 cells. However, compounds **7c**–**7f** initially failed to increase the potency of cell growth inhibition against both tested cancer cell lines; this was successfully addressed by increasing the linker length using additional methylene bridge. On the other hand, elongation of the linker by two to four ethanediol groups resulted in compounds **7g**–**7l**, which showed decreased activity compared to compounds **3b** and **3c**.

Three pairs of compounds possessing stronger anti-proliferative activities were screened out and their cytotoxic activities were preformed against human normal liver cells (L02) in vitro. As shown in Table 4, the parent compound emodin exhibited stronger cytotoxic activity against L02 cells with an IC_50_ value of 22.52 ± 0.18 μM than against cancer cell lines (IC_50_ = 43.87 ± 1.28 μM against HepG2 cells and IC_50_ = 52.72 ± 2.22 μM against MCF-7 cells). However, all the six compounds exhibited weaker cytotoxic activities against L02 cells compared with their corresponding cancer cell lines. Above all, we finally chose to investigate the mechanism of action for compound **7a** because it had the strongest anti-proliferative activity against HepG2 cells.

### 2.3. Selective Inhibition of Cancer Cell Growth by Compounds ***7a***

Lack of selective cytotoxicity is the main factor that hinders conventional chemotherapeutic agents. Thus, to evaluate the selective anti-proliferative activity of the compound **7a**, the selectivity index (SI) between cancer and normal cells was calculated and the results are summarized in Table 5. The SI was calculated by dividing the IC_50_ values in normal cells by the IC_50_ values in cancer cells. Emodin displayed stronger cytotoxic activity, with SI values of 0.51 (HepG2) and 0.43 (MCF-7), respectively, indicating that both normal cells and cancer cells would be killed. However, compound **7a** exhibited weaker cytotoxic activity with SI value of 2.82 (HepG2) and 2.36 (MCF-7), respectively. It means that **7a** exhibited a better selective anti-proliferative activity and specificity than emodin.

### 2.4. Compound ***7a*** Induced Cell Apoptosis through the Mitochondrial Pathway

**Figure 3:**
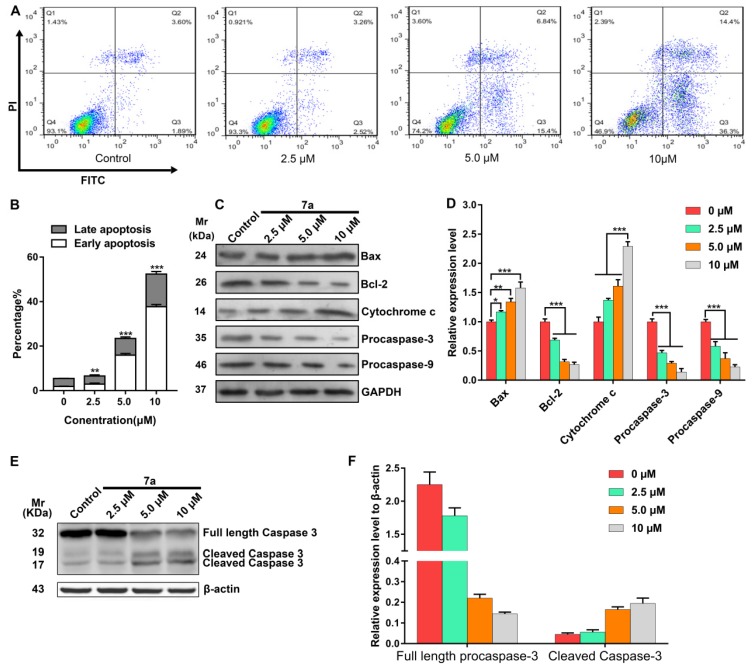
Compound **7a** induced cell apoptosis in HepG2 cells. (**A**) The representative images and statistical results of cell apoptosis assays. (**B**) The quantitative analysis of apoptosis. Data are expressed as means ± SD of the percentages of apoptotic cells from three independent experiments. Statistical significance is determined by two-tailed Student *t*-test: “***” denote *p* < 0.001, “**” denote *p* < 0.01, respectively (Supplementary Table S1). (**C**, **E**) Western blot analysis effect of compound **7a** on the levels of Bax, Bcl-2, cytochrome c, procaspase-3, caspase-3 and procaspase-9 expression in HepG2 cells. (**D**, **F**) An equal amount of protein was loaded on SDS-PAGE gel for western blot analysis. Data are expressed as means ± SD of the percentages of apoptotic cells from three independent experiments. Statistical significance is determined by two-tailed Student *t*-test: “***” denote *p* < 0.001, “**” denote *p* < 0.01, “*” denote *p* < 0.05, respectively (Supplementary Table S2).

In order to verify whether compound **7a** is able to induce apoptosis in HepG2 cells, we utilized FITC-Annexin V/PI staining and estimated the percentage of apoptotic cells by flow cytometry. We noted a concentration-dependent increase in the percentage of apoptotic cells when the cells were treated with compound **7a** for 48 h at concentrations 2.5, 5, and 10 μM. As shown in Figure 3A, few (5.5%) apoptotic cells were present in the control panel. In contrast, the percentage of apoptotic cells increased to 22.2% after treatment with compound **7a** at 5 μM for 48 h and further increased to 50.7% after treatment with **7a** at the concentration of 10 μM. As illustrated in Figure 3B, the quantitative analysis of apoptosis strongly suggests that treatment with compound **7a** effectively induced apoptosis in HepG2 cells in a concentration-dependent manner in comparison to the control.

To verify the molecular mechanisms of apoptosis induction of compound **7a**, we performed a western blot assay. It is well known that the Bcl-2 family of pro-apoptotic and anti-apoptotic proteins regulates the mitochondrial pathway of apoptosis. These Bcl-2 family proteins stimulate the permeabilization of the mitochondrial outer membrane, which results in the release of cytochrome c into the cytosol and in turn promotes the activation of the caspase cascade. The activation of the caspase cascade ultimately leads to the induction of apoptotic cell death. As shown in Figure 3C and 3D, in comparison with the control cells, compound **7a** induced an increase in the levels of Bax and a decrease in the expression of Bcl-2 in a concentration-dependent manner. Meanwhile, the release of cytochrome c from mitochondria increased after the treatment of compound **7a**, while procaspase 9 and procaspase 3 decreased after treatment with **7a**, indicating that the caspase 9 and caspase 3 were activated. As shown in Figure 3E,F, the increased expression of cleaved caspase-3 after treatment with **7a** provided a further evidence that compound **7a** induced cell apoptosis through mitochondrial pathway in a concentration-dependent manner. The apoptosis process can be summarized as follows: The mitochondrial apoptosis-induced channel (MAC) of HepG2 cells was formed by pro-apoptotic protein Bax after the treatment of compound **7a**. The formation of MAC led to the releasing of cytochrome c from mitochondria. Once cytochrome c was released, it binded with apoptotic protease activating factor-1 (Apaf-1) and ATP, which then binded to procaspase-9 to create a protein complex known as apoptosome. The apoptosome cleaved the pro-caspase-9 to its active form of initiator caspase-9, which in turn activated procaspase-3 and then the effector caspase-3 and finally resulted in cell apoptosis.

### 2.5. Compound ***7a*** Induced G0/G1 Phase Arrest

**Figure 4:**
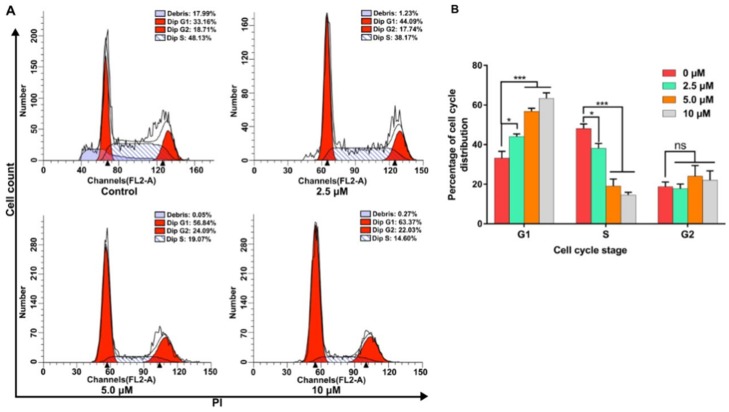
Compound **7a** induced G0/G1 phase arrest. (**A**) The representative images of cell cycle distribution after treatment of compound **7a** at indicated concentrations. (**B**) The statistical results of cell cycle distribution after treatment of compound **7a**. Data are expressed as means ± SD of the percentages of apoptotic cells from three independent experiments. Statistical significance is determined by two-tailed Student *t*-test: “***” denote *p* < 0.001, “*” denote *p* < 0.05 and ns means no significance, respectively (Supplementary Table S3).

To further examine how compound **7a** suppressed the growth of HepG2 cells, the effect of compound **7a** on cell cycle distribution with different concentrations was investigated by flow cytometric analysis following staining the DNA with propidium iodide (PI). The results of a typical experiment are shown in Figure 4A. As determined by flow cytometry, the exposure of HepG2 cells to compound **7a** for 48 h resulted in an obvious increase in the percentage of cells in G0/G1 phase in comparison with the control. Treatment with compound **7a** resulted in an increase of G1 phase cells (63.37%) compared to the control (33.16%). Inversely, S phase cell population decreased to 14.60% compared to the control (48.13%). Therefore, compound **7a** resulted in a significant G0/G1 phase arrest in a concentration-dependent manner with a concomitant decrease in the number of cells in the S phase of the cycle.

### 2.6. Compound ***7a*** Inhibited the Migration of HepG2 Cells

**Figure 5:**
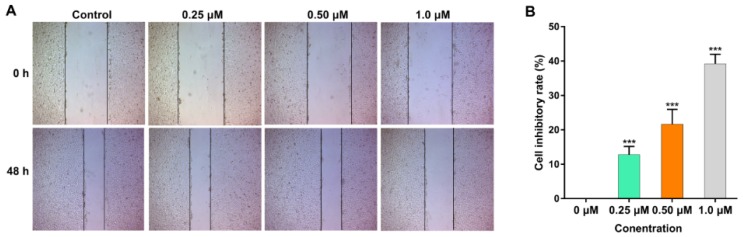
Compound **7a** inhibited migration of HepG2 cells. (**A**) Compound **7a** decreased the HepG2 cell motility compared to the control group. (**B**) Data are expressed as means ± SD of cell inhibitory rate (%) for the migration of HepG2 cells from three independent experiments. Statistical significance is determined by two-tailed Student *t*-test: “***” denote *p* < 0.001 (Supplementary Table S4).

To evaluate the effect of compound **7a** on cancer migration, a wound healing assay was conducted to determine whether compound **7a** could prevent HepG2 cell migration. After culturing HepG2 cells for 48 h in the presence and absence of compound **7a** at 0, 0.25, 0.5, and 1 μM, a pipette tip was streaked through the cell culture, resulting in a cell deficient space. The territory recovered by the HepG2 cells was used to observe the migration inhibition capability of compound **7a**. The empty space reduced significantly in size in the absence of compound **7a** because of cell proliferation and migration and results indicate compound **7a** suppressed the mobility of tumor cells in a concentration-dependent manner (Figure 5). 

## 3. Materials and Methods

### 3.1. General Information

The starting materials and reagents, purchased from commercial suppliers, were used without further purification. Emodin extracted from *Polygonum cuspidatum* was purchased from China Xi’an Sino-Herb Bio-technology Co., Ltd. (Xi’an, China). All reactions were monitored by thin-layer chromatography (TLC) on aluminum sheets (Silica gel 60-F254, E. Merck, Darmstadt, Germany). Compounds were visualized by UV light. Column chromatography was carried out using silica gel (200–300 mesh). All reaction solvents were dried prior to use according to standard procedures. All primary reagents were commercially available. Silica gel chromatography solvents were of analytical grade. NMR spectra were recorded in DMSO-**d*_6_* on a Bruker-250 spectrometer (Bruker Biospin, Fällanden, Switzerlahd), at 400 MHz for ^1^H-NMR, 101 MHz for ^13^C-NMR and 376 MHz for ^19^F-NMR with TMS as the internal standard. Chemical shifts were expressed in δ (ppm) and coupling constants (*J*) in Hz. Multiplicity was indicated as follows: s (singlet), d (doublet), t (triplet), p (quintet), dd (doublet of doublets), brs (broad singlet), etc. Mass spectra were obtained on an Agilent 1100 Series LC/MSD Trap mass spectrometer (ESI-MS, Agilent, Santa Clara, CA, USA).

### 3.2. Chemistry

*1,8-Dihydroxy-3-(2-hydroxyethoxy)-6-methylanthracene-9,10-dione* (**2**) To a mixture of emodin (10.0 g, 37.0 mmol) in dry DMF (150 mL) were added Cs_2_CO_3_ (13.2 g, 40.5 mmol) and 2-iodoethanol (19.1 g, 111 mmol) at room temperature. After stirring for 36 h at 60 °C, the resulting mixture was evaporated under reduced pressure and then mixed with water (500 mL). The pH value of aqueous phase was adjusted to around 5 with 10% hydrochloric acid solution. The yellow precipitate was collected and washed with water to give the crude product, which was in further purification by triturating twice with ethyl acetate (100 mL) and filtered to afford compound **2** (7.6 g, 65%) as a brown solid; ^1^H-NMR δ 12.17 (s, 1H), 11.99 (s, 1H), 7.54 (d, *J* = 1.6 Hz, 1H), 7.21 (d, *J* = 2.6 Hz, 2H), 6.89 (d, *J* = 2.5 Hz, 1H), 4.98 (t, *J* = 5.5 Hz, 1H), 4.26–4.15 (m, 2H), 3.76 (q, *J* = 5.2 Hz, 2H), 2.44 (s, 3H); ^13^C-NMR δ 189.75, 180.93, 165.58, 164.30, 161.41, 148.42, 134.61, 132.66, 124.11, 120.48, 113.26, 109.60, 107.95, 106.89, 70.76, 59.20, 21.49; ESI, *m*/*z*: 315.05 [M + H]^+^.

General procedure **A** for preparation of compounds **3a**–**3z, 4a**–**4b**, and **7a**–**7l**

To a mixture of compound **2** (1.00 mmol) in dry dichloromethane (20 mL) were added various *N*-Boc amino acids (1.20 mmol), dicyclohexyl carbodiimide (DCC) (4.50 mmol) and 4-(*N*,*N*-dimethlyamino)pyridine (DMAP) (1.00 mmol) at 0 °C. After stirring about 30 min to 5 h at 0 °C, TLC analysis showed the complete consumption of compound **2**, and then the resulting mixture was added dropwise TFA (5 mL) at the same temperature and kept stirring for anther about 2 h. The insoluble side product was filtered out and the filtrate was evaporated to give the residue, which was purified by reverse phase flash chromatography with the following conditions: Column: Spherical C18, 20–40 μm, 330 g; Mobile Phase A: Water (plus 5 mM TFA); Mobile Phase B: ACN; Flow rate: 80 mL/min; Gradient: 5% B gradient in 10 min, 25% B–45% B gradient in 25 min; Detector: 254 nm. The fractions containing the desired product were collected at around 40% B and concentrated under reduced pressure to afford compounds **3a**–**3z** in 20% to 50% yields.

*2-(2-(4,5-Dihydroxy-7-methyl-9,10-dioxo-9,10-dihydroanthracen-2-yloxy)ethoxy)-2-oxoethanaminium 2,2,2-trifluoroacetate* (**3a**). According to the general procedure **A**, compound **2** was treated with *N*-Boc-glycine and then purified by reverse phase flash chromatography to give compound **3a**: Yellow solid; yield, 45%; ^1^H-NMR δ 9.76 (brs, 3H), 7.50 (s, 1H), 7.19 (s, 1H), 7.16 (s, 1H), 6.90 (s, 1H), 4.56 (d, *J* = 4.9 Hz, 2H), 4.44 (d, *J* = 5.1 Hz, 2H), 3.90 (s, 2H), 2.42 (s, 3H); ^13^C-NMR δ 189.89, 181.04, 167.77, 164.74, 164.25, 161.46, 148.58, 134.82, 132.73, 124.23, 120.57, 113.38, 110.06, 107.80, 107.08, 66.71, 63.44, 21.50; ^19^F-NMR δ − 73.56; ESI, *m*/*z*: 372.1 [M + H − CF_3_COOH]^+^.

*(R)-1-(2-(4,5-Dihydroxy-7-methyl-9,10-dioxo-9,10-dihydroanthracen-2-yloxy)ethoxy)-1-oxopropan-2-aminium 2,2,2-trifluoroacetate* (**3b**). According to the general procedure **A**, compound **2** was treated with *N*-Boc-d-alanine and then purified by reverse phase flash chromatography to give compound **3b**: Yellow solid; yield, 50%; ^1^H-NMR δ 9.50 (brs, 3H), 7.44 (d, *J* = 1.7 Hz, 1H), 7.14 (s, 1H), 7.11 (d, *J* = 2.5 Hz, 1H), 6.86 (d, *J* = 2.5 Hz, 1H), 4.65–4.50 (m, 2H), 4.43 (t, *J* = 4.5 Hz, 2H), 4.18 (q, *J* = 7.2 Hz, 1H), 2.40 (s, 3H), 1.44 (d, *J* = 7.2 Hz, 3H); ^13^C-NMR δ 189.78, 180.88, 170.03, 164.76, 164.24, 161.44, 148.53, 134.71, 132.62, 124.17, 120.52, 113.26, 109.96, 107.76, 107.04, 66.66, 63.58, 47.87, 21.49, 15.72; ^19^F-NMR δ − 73.53; ESI, *m*/*z*: 386.15 [M + H − CF_3_COOH]^+^.

*(S)-1-(2-(4,5-Dihydroxy-7-methyl-9,10-dioxo-9,10-dihydroanthracen-2-yloxy)ethoxy)-1-oxopropan-2-aminium 2,2,2-trifluoroacetate* (**3c**). According to the general procedure **A**, compound **2** was treated with *N*-Boc-l-alanine and then purified by reverse phase flash chromatography to give compound **3c**: Yellow solid; yield, 48%; ^1^H-NMR δ 9.85 (brs, 3H), 7.47 (d, *J* = 1.7 Hz, 1H), 7.16 (s, 1H), 7.14 (d, *J* = 2.5 Hz, 1H), 6.88 (d, *J* = 2.5 Hz, 1H), 4.64–4.53 (m, 2H), 4.45 (d, *J* = 4.3 Hz, 2H), 4.17 (q, *J* = 7.2 Hz, 1H), 2.41 (s, 3H), 1.43 (d, *J* = 7.1 Hz, 3H); ^13^C-NMR δ 189.82, 180.95, 170.13, 164.77, 164.25, 161.45, 148.54, 134.76, 132.67, 124.19, 120.54, 113.31, 110.00, 107.77, 107.07, 66.67, 63.56, 47.88, 21.49, 15.80; ^19^F-NMR δ − 73.54; ESI, *m*/*z*: 386.15 [M + H − CF_3_COOH]^+^.

*(S)-1-(2-(4,5-Dihydroxy-7-methyl-9,10-dioxo-9,10-dihydroanthracen-2-yloxy)ethoxy)-3-hydroxy-1-oxopropan-2-aminium 2,2,2-trifluoroacetate* (**3d**). According to the general procedure **A**, compound **2** was treated with *N*-Boc-*O*-*tert*-butyl-l-serine and then purified by reverse phase flash chromatography to give compound **3d**: Yellow solid; yield, 20%; ^1^H-NMR δ 7.51 (s, 1H), 7.18 (s, 1H), 7.17 (d, *J* = 2.5 Hz, 1H), 6.90 (d, *J* = 2.5 Hz, 1H), 5.53 (s, 1H), 4.52 (d, *J* = 3.6 Hz, 2H), 4.42 (d, *J* = 3.3 Hz, 2H), 4.16 (s, 1H), 3.83–3.73 (m, 2H), 2.40 (s, 3H); ^13^C-NMR δ 189.93, 181.12, 168.16, 164.81, 164.25, 161.46, 148.57, 134.85, 132.77, 124.23, 120.57, 113.41, 110.07, 107.85, 107.11, 66.69, 63.63, 59.53, 54.25, 21.49; ^19^F-NMR δ − 73.48; ESI, *m*/*z*: 402.05 [M + H − CF_3_COOH]^+^.

*(R)-1-(2-(4,5-Dihydroxy-7-methyl-9,10-dioxo-9,10-dihydroanthracen-2-yloxy)ethoxy)-3-methyl-1-oxobutan-2-aminium 2,2,2-trifluoroacetate* (**3e**). According to the general procedure **A**, compound **2** was treated with *N*-Boc-d-valine and then purified by reverse phase flash chromatography to give compound **3e**: Yellow solid; yield, 46%; ^1^H-NMR δ 8.90 (brs, 2H), 7.49 (s, 1H), 7.18 (s, 1H), 7.15 (d, *J* = 2.5 Hz, 1H), 6.90 (d, *J* = 2.5 Hz, 1H), 4.67 (d, *J* = 12.7 Hz, 1H), 4.60–4.39 (m, 3H), 4.00 (d, *J* = 4.5 Hz, 1H), 2.42 (s, 3H), 2.18 (dd, *J* = 12.4, 6.5 Hz, 1H), 0.99 (dd, *J* = 14.8, 6.9 Hz, 6H); ^13^C-NMR δ 189.89, 181.04, 168.97, 164.68, 164.26, 161.46, 148.57, 134.85, 132.73, 124.23, 120.57, 113.37, 110.09, 107.74, 107.05, 66.65, 63.45, 57.24, 29.44, 21.50, 18.13, 17.37; ^19^F-NMR δ − 73.55; ESI, *m*/*z*: 414.15 [M + H − CF_3_COOH]^+^.

*(S)-1-(2-(4,5-Dihydroxy-7-methyl-9,10-dioxo-9,10-dihydroanthracen-2-yloxy)ethoxy)-3-methyl-1-oxobutan-2-aminium 2,2,2-trifluoroacetate* (**3f**). According to the general procedure **A**, compound **2** was treated with *N*-Boc-l-valine and then purified by reverse phase flash chromatography to give compound **3f**: Yellow solid; yield, 48%; ^1^H-NMR δ 8.84 (brs, 2H), 7.48 (d, *J* = 1.6 Hz, 1H), 7.17 (s, 1H), 7.14 (d, *J* = 2.5 Hz, 1H), 6.89 (d, *J* = 2.6 Hz, 1H), 4.74–4.62 (m, 1H), 4.57–4.39 (m, 3H), 4.00 (d, *J* = 4.5 Hz, 1H), 2.42 (s, 3H), 2.22–2.14 (m, 1H), 0.99 (dd, *J* = 15.0, 6.9 Hz, 6H); ^13^C-NMR δ 189.87, 181.00, 168.95, 164.67, 164.26, 161.46, 148.56, 134.82, 132.71, 124.22, 120.56, 113.35, 110.07, 107.74, 107.04, 66.65, 63.45, 57.23, 29.43, 21.50, 18.13, 17.37; ^19^F-NMR δ − 73.54; ESI, *m*/*z*: 414.15 [M + H − CF_3_COOH]^+^.

*(S)-1-(2-(4,5-Dihydroxy-7-methyl-9,10-dioxo-9,10-dihydroanthracen-2-yloxy)ethoxy)-4-methyl-1-oxopentan-2-aminium 2,2,2-trifluoroacetate* (**3g**). According to the general procedure **A**, compound **2** was treated with *N*-Boc-l-leucine and then purified by reverse phase flash chromatography to give compound **3g**: Yellow solid; yield, 42%; ^1^H-NMR δ 9.43 (brs, 3H), 7.48 (d, *J* = 1.7 Hz, 1H), 7.17 (s, 1H), 7.14 (d, *J* = 2.5 Hz, 1H), 6.89 (d, *J* = 2.5 Hz, 1H), 4.68–4.58 (m, 1H), 4.56–4.42 (m, 3H), 4.05 (t, *J* = 7.1 Hz, 1H), 2.42 (s, 3H), 1.81–1.54 (m, 3H), 0.88 (d, *J* = 2.2 Hz, 3H), 0.87 (d, *J* = 2.3 Hz, 3H); ^13^C-NMR δ 189.84, 180.97, 170.02, 164.77, 164.27, 161.46, 148.57, 134.78, 132.68, 124.22, 120.57, 113.33, 110.02, 107.82, 107.03, 66.59, 63.59, 50.56, 23.68, 22.06, 21.85, 21.50; ^19^F-NMR δ − 73.51; ESI, *m*/*z*: 428.1 [M + H − CF_3_COOH]^+^. 

*(2S)-1-(2-(4,5-dihydroxy-7-methyl-9,10-dioxo-9,10-dihydroanthracen-2-yloxy)ethoxy)-3-methyl-1-oxo-pentan-2-aminium 2,2,2-trifluoroacetate* (**3h**). According to the general procedure **A**, compound **2** was treated with *N*-Boc-l-isoleucine and then purified by reverse phase flash chromatography to give compound **3h**: Yellow solid; yield, 42%; ^1^H-NMR δ 7.52 (s, 1H), 7.21 (s, 1H), 7.17 (d, *J* = 2.5 Hz, 1H), 6.92 (d, *J* = 2.5 Hz, 1H), 4.73–4.63 (m, 1H), 4.56–4.41 (m, 3H), 4.06 (d, *J* = 3.9 Hz, 1H), 2.44 (s, 3H), 1.89 (s, 1H), 1.47 (m, 1H), 1.35–1.24 (m, 1H), 0.93 (d, *J* = 6.9 Hz, 3H), 0.87 (t, *J* = 7.3 Hz, 3H); ^13^C-NMR δ 189.93, 181.10, 168.86, 164.69, 164.28, 161.47, 148.59, 134.88, 132.77, 124.26, 120.59, 113.42, 110.11, 107.79, 107.05, 66.62, 63.43, 56.13, 36.05, 25.09, 21.50, 14.06, 11.44; ^19^F-NMR δ − 73.50; ESI, *m*/*z*: 428.1 [M + H − CF_3_COOH]^+^.

*(S)-1-(2-(4,5-Dihydroxy-7-methyl-9,10-dioxo-9,10-dihydroanthracen-2-yloxy)ethoxy)-4-(methylthio)-1-oxobutan-2-aminium 2,2,2-trifluoroacetate* (**3i**). According to the general procedure **A**, compound **2** was treated with *N*-Boc-l-methionine and then purified by reverse phase flash chromatography to give compound **3i**: Yellow solid; yield, 38%; ^1^H-NMR δ 7.51 (d, *J* = 1.7 Hz, 1H), 7.20 (s, 1H), 7.18 (d, *J* = 2.5 Hz, 1H), 6.91 (d, *J* = 2.6 Hz, 1H), 4.63 (d, *J* = 12.5 Hz, 1H), 4.58–4.37 (m, 3H), 4.18 (t, *J* = 6.2 Hz, 1H), 2.67–2.52 (m, 2H), 2.42 (s, 3H), 2.05 (dd, *J* = 8.2, 5.9 Hz, 2H), 2.00 (s, 3H); ^13^C-NMR δ 189.95, 181.10, 169.41, 164.76, 164.28, 161.48, 148.62, 134.89, 132.78, 124.26, 120.61, 113.42, 110.13, 107.79, 107.11, 66.63, 63.71, 50.99, 29.61, 28.21, 21.53, 14.20; ^19^F-NMR δ − 73.47; ESI, *m*/*z*: 446.1 [M + H − CF_3_COOH]^+^.

*1-(2-(4,5-Dihydroxy-7-methyl-9,10-dioxo-9,10-dihydroanthracen-2-yloxy)ethoxy)-2-methyl-1-oxopropan-2-aminium 2,2,2-trifluoroacetate* (**3j**). According to the general procedure **A**, compound **2** was treated with *N*-Boc-Aib-OH and then purified by reverse phase flash chromatography to give compound **3j**: Yellow solid; yield, 38%; ^1^H-NMR δ 11.96 (br, 2H), 8.51 (br, 3H), 7.54 (s, 1H), 7.34–7.14 (m, 2H), 6.94 (d, *J* = 2.5 Hz, 1H), 4.65–4.54 (m, 2H), 4.47 (t, *J* = 4.3 Hz, 2H), 2.44 (s, 3H), 1.47 (s, 6H); ^13^C-NMR δ 189.33, 180.18, 171.67, 164.71, 164.21, 161.37, 148.37, 134.23, 132.13, 123.96, 120.35, 112.78, 109.52, 107.66, 106.79, 66.61, 63.79, 55.98, 23.37, 21.47; ^19^F-NMR δ − 73.54; ESI, *m*/*z*: 400.2 [M + H − CF_3_COOH]^+^.

*1-((2-(4,5-Dihydroxy-7-methyl-9,10-dioxo-9,10-dihydroanthracen-2-yloxy)ethoxy)carbonyl)cyclopropan-aminium 2,2,2-trifluoroacetate* (**3k**). According to the general procedure **A**, compound **2** was treated with 1-(Boc-amino)cyclopropanecarboxylic acid and then purified by reverse phase flash chromatography to give compound **3k**: Yellow solid; yield, 28%; ^1^H-NMR δ 11.96 (brs, 1H), 8.87 (brs, 2H), 7.52 (d, *J* = 1.7 Hz, 1H), 7.24–7.10 (m, 2H), 6.92 (d, *J* = 2.6 Hz, 1H), 4.53 (dd, *J* = 5.9, 2.7 Hz, 2H), 4.43 (dd, *J* = 5.8, 2.8 Hz, 2H), 2.43 (s, 3H), 1.50–1.41 (m, 2H), 1.38–1.30 (m, 2H); ^13^C-NMR δ 189.28, 180.10, 169.83, 164.69, 164.22, 161.39, 148.39, 134.15, 132.07, 123.98, 120.37, 112.73, 109.54, 107.67, 106.74, 66.57, 63.78, 33.87, 21.51, 13.69; ^19^F-NMR δ − 73.55; ESI, *m*/*z*: 398.2 [M + H − CF_3_COOH]^+^.

*1-((2-(4,5-Dihydroxy-7-methyl-9,10-dioxo-9,10-dihydroanthracen-2-yloxy)ethoxy)carbonyl)cyclobutan-aminium 2,2,2-trifluoroacetate* (**3l**). According to the general procedure **A**, compound **2** was treated with 1-(Boc-amino)cyclobutanecarboxylic acid and then purified by reverse phase flash chromatography to give compound **3l**: Yellow solid; yield, 28%; ^1^H-NMR δ 11.95 (brs, 1H), 8.73 (brs, 2H), 7.52 (d, *J* = 1.6 Hz, 1H), 7.30–7.09 (m, 2H), 6.95 (d, *J* = 2.6 Hz, 1H), 4.74–4.58 (m, 2H), 4.51 (t, *J* = 4.3 Hz, 2H), 2.54 (m, 2H), 2.43 (s, 3H), 2.37 (m, , 2H), 2.03 (m, 2H); ^13^C-NMR δ 189.10, 179.79, 170.89, 164.71, 164.22, 161.36, 148.28, 134.00, 131.88, 123.85, 120.24, 112.54, 109.30, 107.63, 106.62, 66.66, 63.68, 56.86, 29.63, 21.42, 14.41; ^19^F-NMR δ − 73.51; ESI, *m*/*z*: 412.2 [M + H − CF_3_COOH]^+^.

*3-((2-(4,5-Dihydroxy-7-methyl-9,10-dioxo-9,10-dihydroanthracen-2-yloxy)ethoxy)carbonyl)oxetan-3-aminium 2,2,2-trifluoroacetat*e (**3m**). According to the general procedure **A**, compound **2** was treated with 3-Boc-amino-3-oxetanecarboxylic acid and then purified by reverse phase flash chromatography to give compound **3m**: Yellow solid; yield, 37%; ^1^H-NMR δ 11.97 (brs, 2H), 8.76 (brs, 3H), 7.55 (d, *J* = 1.7 Hz, 1H), 7.23 (d, *J* = 2.3 Hz, 2H), 6.97 (d, *J* = 2.5 Hz, 1H), 4.83 (d, *J* = 7.6 Hz, 2H), 4.65 (dd, *J* = 9.7, 6.2 Hz, 4H), 4.53 (dd, *J* = 5.3, 2.9 Hz, 2H), 2.44 (s, 3H); ^13^C-NMR δ 189.35, 180.21, 168.11, 164.71, 164.22, 161.38, 148.43, 134.24, 132.14, 124.01, 120.40, 112.80, 109.54, 107.69, 106.82, 75.75, 66.58, 64.24, 57.15, 21.48; ^19^F-NMR δ − 73.51; ESI, *m*/*z*: 414.1 [M + H − CF_3_COOH]^+^.

*1-((2-(4,5-Dihydroxy-7-methyl-9,10-dioxo-9,10-dihydroanthracen-2-yloxy)ethoxy)carbonyl)cyclopentan-aminium 2,2,2-trifluoroacetate* (**3n**). According to the general procedure **A**, compound **2** was treated with *N*-Boc-aminocyclopentanecarboxylic acid and then purified by reverse phase flash chromatography to give compound **3n**: Yellow solid; yield, 31%; ^1^H-NMR δ 11.98 (brs, 2H), 8.47 (brs, 3H), 7.55 (d, *J* = 1.6 Hz, 1H), 7.30–7.12 (m, 2H), 6.96 (d, *J* = 2.5 Hz, 1H), 4.58 (dd, *J* = 6.0, 2.8 Hz, 2H), 4.48 (t, *J* = 4.2 Hz, 2H), 2.45 (s, 3H), 2.18 (dd, *J* = 14.0, 6.0 Hz, 2H), 1.89–1.69 (m, 6H); ^13^C-NMR δ 189.38, 180.26, 172.16, 164.74, 164.23, 161.39, 148.39, 134.31, 132.19, 123.99, 120.36, 112.84, 109.58, 107.69, 106.81, 66.63, 64.33, 63.73, 36.31, 25.09, 21.46; ^19^F-NMR δ − 73.49; ESI, *m*/*z*: 425.1 [M + H − CF_3_COOH]^+^.

*(R)-2-((2-(4,5-Dihydroxy-7-methyl-9,10-dioxo-9,10-dihydroanthracen-2-yloxy)ethoxy)carbonyl)-pyrrolidinium 2,2,2-trifluoroacetate* (**3o**). According to the general procedure **A**, compound **2** was treated with *N*-Boc-d-proline and then purified by reverse phase flash chromatography to give compound **3o**: Yellow solid; yield, 44%; ^1^H-NMR δ 7.52 (d, *J* = 1.7 Hz, 1H), 7.20 (s, 1H), 7.18 (d, *J* = 2.6 Hz, 1H), 6.92 (d, *J* = 2.6 Hz, 1H), 4.64–4.53 (m, 2H), 4.51–4.42 (m, 3H), 3.27–3.16 (m, 2H), 2.43 (s, 3H), 2.36–2.19 (m, 1H), 2.07–1.86 (m, 3H); ^13^C-NMR δ 189.96, 181.16, 168.92, 164.80, 164.28, 161.49, 148.62, 134.91, 132.80, 124.30, 120.62, 113.44, 110.14, 107.78, 107.17, 66.67, 63.87, 58.63, 45.59, 27.79, 23.00, 21.52; ^19^F-NMR δ − 73.51; ESI, *m*/*z*: 412.1 [M + H − CF_3_COOH]^+^.

*(S)-2-((2-(4,5-Dihydroxy-7-methyl-9,10-dioxo-9,10-dihydroanthracen-2-yloxy)ethoxy)carbonyl)-pyrrolidinium 2,2,2-trifluoroacetate* (**3p**). According to the general procedure **A**, compound **2** was treated with *N*-Boc-l-proline and then purified by reverse phase flash chromatography to give compound **3p**: Yellow solid; yield, 37%; ^1^H-NMR δ 7.50 (d, *J* = 1.7 Hz, 1H), 7.19 (t, *J* = 1.3 Hz, 1H), 7.17 (d, *J* = 2.6 Hz, 1H), 6.91 (d, *J* = 2.5 Hz, 1H), 4.67–4.54 (m, 2H), 4.52–4.38 (m, 3H), 3.30–3.16 (m, 2H), 2.42 (s, 3H), 2.32–2.25 (m, 1H), 2.09–1.82 (m, 3H); ^13^C-NMR δ 189.93, 181.09, 168.91, 164.79, 164.27, 161.48, 148.61, 134.87, 132.77, 124.26, 120.60, 113.41, 110.11, 107.77, 107.15, 66.67, 63.86, 58.62, 45.58, 27.80, 23.00, 21.52; ^19^F-NMR δ − 73.50; ESI, *m*/*z*: 412.1 [M + H − CF_3_COOH]^+^.

*(R)-2-(2-(4,5-Dihydroxy-7-methyl-9,10-dioxo-9,10-dihydroanthracen-2-yloxy)ethoxy)-2-oxo-1-phenyl-ethanaminium 2,2,2-trifluoroacetate* (**3q**). According to the general procedure **A**, compound **2** was treated with *N*-Boc-d-phenylglycine and then purified by reverse phase flash chromatography to give compound **3q**: Yellow solid; yield, 39%; ^1^H-NMR δ 11.97 (brs, 2H), 8.89 (brs, 3H), 7.56 (d, *J* = 1.7 Hz, 1H), 7.49 (dd, *J* = 6.8, 3.0 Hz, 2H), 7.41 (dd, *J* = 5.1, 2.1 Hz, 3H), 7.23 (s, 1H), 7.08 (d, *J* = 2.5 Hz, 1H), 6.82 (d, *J* = 2.5 Hz, 1H), 5.38 (s, 1H), 4.58 (q, *J* = 5.0 Hz, 2H), 4.38–4.31 (m, 2H), 2.44 (s, 3H); ^13^C-NMR δ 189.06, 179.81, 168.64, 164.44, 164.18, 161.33, 148.26, 133.91, 132.65, 131.90, 129.70, 129.09, 128.48, 123.79, 120.33, 112.54, 109.26, 107.69, 106.46, 66.40, 63.82, 55.81, 21.41; ^19^F-NMR δ − 73.46; ESI, *m*/*z*: 448.1 [M + H − CF_3_COOH]^+^.

*(S)-2-(2-(4,5-Dihydroxy-7-methyl-9,10-dioxo-9,10-dihydroanthracen-2-yloxy)ethoxy)-2-oxo-1-phenyl-ethanaminium 2,2,2-trifluoroacetate* (**3r**). According to the general procedure **A**, compound **2** was treated with *N*-Boc-l-phenylglycine and then purified by reverse phase flash chromatography to give compound **3r**: Yellow solid; yield, 45%; ^1^H-NMR δ 11.95 (brs, 1H), 8.84 (br, 2H), 7.56 (d, *J* = 1.7 Hz, 1H), 7.49 (dd, *J* = 6.8, 3.0 Hz, 2H), 7.41 (dd, *J* = 5.1, 2.1 Hz, 3H), 7.23 (s, 1H), 7.08 (d, *J* = 2.5 Hz, 1H), 6.82 (d, *J* = 2.5 Hz, 1H), 5.37 (s, 1H), 4.58 (q, *J* = 5.0 Hz, 2H), 4.38–4.31 (m, 2H), 2.44 (s, 3H); ^13^C-NMR δ 189.90, 181.06, 168.50, 164.62, 164.23, 161.47, 148.57, 134.73, 132.76, 132.41, 129.51, 128.89, 128.15, 124.22, 120.58, 113.39, 110.01, 107.93, 106.94, 66.50, 63.82, 55.41, 21.50; ^19^F-NMR δ − 73.45; ESI, *m*/*z*: 448.1 [M + H − CF_3_COOH]^+^.

*(R)-2-(2-(4,5-Dihydroxy-7-methyl-9,10-dioxo-9,10-dihydroanthracen-2-yloxy)ethoxy)-1-(4-hydroxyphenyl)-2-oxoethanaminium 2,2,2-trifluoroacetate* (**3s**). According to the general procedure **A**, compound **2** was treated with *N*-Boc-d-4-hydroxyphenylglycine and then purified by reverse phase flash chromatography to give compound **3s**: Yellow solid; yield, 42%; ^1^H-NMR δ 11.99 (brs, 2H), 9.73 (s, 1H), 8.66 (brs, 3H), 7.57 (s, 1H), 7.26 (d, *J* = 7.8 Hz, 3H), 7.13 (s, 1H), 6.85 (s, 1H), 6.72 (d, *J* = 8.1 Hz, 2H), 5.22 (s, 1H), 4.57 (s, 2H), 4.35 (s, 2H), 2.46 (s, 3H); ^13^C-NMR δ 189.65, 180.61, 168.86, 164.62, 164.25, 161.45, 158.62, 148.47, 134.46, 132.48, 129.67, 124.09, 122.44, 120.50, 115.64, 113.12, 109.76, 107.81, 106.79, 66.57, 63.71, 55.11, 21.51; ^19^F-NMR δ − 73.43; ESI, *m*/*z*: 464.2 [M + H − CF_3_COOH]^+^.

*(S)-2-(2-(4,5-Dihydroxy-7-methyl-9,10-dioxo-9,10-dihydroanthracen-2-yloxy)ethoxy)-1-(4-hydroxyphenyl)-2-oxoethanaminium 2,2,2-trifluoroacetate* (**3t**). According to the general procedure **A**, compound **2** was treated with *N*-Boc-l-4-hydroxyphenylglycine and then purified by reverse phase flash chromatography to give compound **3t**: Yellow solid; yield, 40%; ^1^H-NMR δ 11.96 (brs, 1H), 9.73 (s, 1H), 8.73 (brs, 2H), 7.57 (s, 1H), 7.25 (d, *J* = 9.1 Hz, 3H), 7.15–7.11 (m, 1H), 6.84 (s, 1H), 6.72 (d, *J* = 8.2 Hz, 2H), 5.21 (s, 1H), 4.63–4.49 (m, 2H), 4.35 (s, 2H), 2.45 (s, 3H); ^13^C-NMR δ 189.65, 180.61, 168.90, 164.63, 164.27, 161.46, 158.63, 148.49, 134.46, 132.49, 129.69, 124.11, 122.47, 120.51, 115.65, 113.13, 109.76, 107.83, 106.79, 66.59, 63.72, 55.12, 21.53; ^19^F-NMR δ − 73.44; ESI, *m*/*z*: 464.1 [M + H − CF_3_COOH]^+^.

*(R)-2-(2-(4,5-Dihydroxy-7-methyl-9,10-dioxo-9,10-dihydroanthracen-2-yloxy)ethoxy)-1-(4-fluorophenyl)-2-oxoethanaminium 2,2,2-trifluoroacetate* (**3u**). According to the general procedure **A**, compound **2** was treated with (*R*)-*N*-Boc-4-fluorophenylglycine and then purified by reverse phase flash chromatography to give compound **3u**: Yellow solid; yield, 45%; ^1^H-NMR δ 11.97 (brs, 2H), 8.87 (brs, 3H), 7.57–7.49 (m, 3H), 7.27–7.19 (m, 3H), 7.06 (d, *J* = 2.6 Hz, 1H), 6.81 (d, *J* = 2.5 Hz, 1H), 5.41 (s, 1H), 4.72–4.45 (m, 2H), 4.35 (t, *J* = 4.3 Hz, 2H), 2.44 (s, 3H); ^13^C-NMR δ 189.41, 180.29, 168.43, 164.53, 164.20, 161.44, 148.39, 134.23, 132.24, 130.77, 130.68, 128.86, 128.83, 123.99, 120.39, 115.98, 115.77, 112.87, 109.54, 107.74, 106.64, 66.44, 63.88, 54.79, 21.44; ^19^F-NMR δ − 73.44, − 111.56; ESI, *m*/*z*: 466.1 [M + H − CF_3_COOH]^+^.

*(S)-2-(2-(4,5-Dihydroxy-7-methyl-9,10-dioxo-9,10-dihydroanthracen-2-yloxy)ethoxy)-1-(4-fluorophenyl)-2-oxoethanaminium 2,2,2-trifluoroacetate* (**3v**). According to the general procedure **A**, compound **2** was treated with (*S*)-*N*-Boc-4-fluorophenylglycine and then purified by reverse phase flash chromatography to give compound **3v**: Yellow solid; yield, 43%; ^1^H-NMR (400 MHz, DMSO-*d*_6_) δ 11.95 (brs, 2H), 8.89 (brs, 3H), 7.57–7.49 (m, 3H), 7.27–7.19 (m, 3H), 7.05 (d, *J* = 2.6 Hz, 1H), 6.80 (d, *J* = 2.5 Hz, 1H), 5.41 (s, 1H), 4.74–4.47 (m, 2H), 4.34 (t, *J* = 4.3 Hz, 2H), 2.44 (s, 3H); ^13^C-NMR (101 MHz, DMSO-*d*_6_) δ 189.87, 180.99, 168.35, 164.62, 164.23, 161.46, 148.57, 134.69, 132.73, 130.60, 130.52, 128.72, 128.69, 124.20, 120.57, 115.92, 115.70, 113.38, 109.96, 107.85, 106.88, 66.48, 63.90, 54.63, 21.49; ^19^F-NMR (376 MHz, DMSO-*d*_6_) δ − 73.48, − 111.55; ESI, *m*/*z*: 466.1 [M + H − CF_3_COOH]^+^.

*(R)-1-(2-(4,5-Dihydroxy-7-methyl-9,10-dioxo-9,10-dihydroanthracen-2-yloxy)ethoxy)-1-oxo-3-phenyl-propan-2-aminium 2,2,2-trifluoroacetate* (**3w**). According to the general procedure **A**, compound **2** was treated with *N*-Boc-d-phenylalanine and then purified by reverse phase flash chromatography to give compound **3w**: Yellow solid; yield, 48%; ^1^H-NMR δ 11.90 brs, 1H), 8.60 (brs, 2H), 7.47 (s, 1H), 7.29–7.21 (m, 5H), 7.16 (s, 1H), 7.13–7.09 (m, 1H), 6.83 (d, *J* = 2.5 Hz, 1H), 4.58–4.22 (m, 5H), 3.23–3.00 (m, 2H), 2.40 (s, 3H); ^13^C-NMR δ 189.88, 181.02, 169.06, 164.69, 164.27, 161.48, 148.57, 134.76, 134.47, 132.71, 129.41, 128.54, 127.28, 124.22, 120.59, 113.37, 110.02, 107.87, 107.01, 66.52, 63.60, 53.14, 36.01, 21.52; ^19^F-NMR δ − 73.47; ESI, *m*/*z*: 462.1 [M + H − CF_3_COOH]^+^.

*(S)-1-(2-(4,5-Dihydroxy-7-methyl-9,10-dioxo-9,10-dihydroanthracen-2-yloxy)ethoxy)-1-oxo-3-phenyl-propan-2-aminium 2,2,2-trifluoroacetate* (**3x**). According to the general procedure **A**, compound **2** was treated with *N*-Boc-l-phenylalanine and then purified by reverse phase flash chromatography to give compound **3x**: Yellow solid; yield, 45%; ^1^H-NMR δ 11.90 (brs, 1H), 8.59 (brs, 2H), 7.47 (d, *J* = 1.7 Hz, 1H), 7.31–7.21 (m, 5H), 7.16 (d, *J* = 1.6 Hz, 1H), 7.12 (d, *J* = 2.5 Hz, 1H), 6.84 (d, *J* = 2.5 Hz, 1H), 4.67–4.13 (m, 5H), 3.26–2.96 (m, 2H), 2.40 (s, 3H); ^13^C-NMR δ 189.88, 181.03, 169.05, 164.69, 164.27, 161.48, 148.57, 134.76, 134.46, 132.72, 129.41, 128.54, 127.28, 124.22, 120.59, 113.37, 110.02, 107.87, 107.01, 66.53, 63.60, 53.14, 36.00, 21.52; ^19^F-NMR δ − 73.51; ESI, *m*/*z*: 462.1 [M + H − CF_3_COOH]^+^.

*(R)-1-(2-(4,5-Dihydroxy-7-methyl-9,10-dioxo-9,10-dihydroanthracen-2-yloxy)ethoxy)-3-(4-hydroxyphenyl)-1-oxopropan-2-aminium 2,2,2-trifluoroacetate* (**3y**). According to the general procedure **A**, compound **2** was treated with *N*-Boc-*O*-*tert*-butyl-l-tyrosine and then purified by reverse phase flash chromatography to give compound **3y**: Yellow solid; yield, 26%; ^1^H-NMR δ 9.33 (s, 1H), 7.54 (d, *J* = 1.6 Hz, 1H), 7.22 (s, 1H), 7.20 (d, *J* = 2.5 Hz, 1H), 7.01 (d, *J* = 2.0 Hz, 1H), 6.99 (d, *J* = 2.1 Hz, 1H), 6.91 (d, *J* = 2.5 Hz, 1H), 6.64 (d, *J* = 2.0 Hz, 1H), 6.62 (d, *J* = 1.9 Hz, 1H), 4.59–4.31 (m, 4H), 4.27 (t, *J* = 6.2 Hz, 1H), 3.06–2.91 (m, 2H), 2.43 (s, 3H); ^13^C-NMR δ 190.03, 181.19, 169.29, 164.76, 164.30, 161.50, 156.67, 148.62, 134.95, 132.86, 130.44, 124.27, 124.18, 120.64, 115.32, 113.49, 110.17, 107.82, 107.09, 66.63, 63.60, 53.39, 35.30, 21.54; ^19^F-NMR δ − 73.49; ESI, *m*/*z*: 478.1 [M + H − CF_3_COOH]^+^.

*(S)-1-(2-(4,5-Dihydroxy-7-methyl-9,10-dioxo-9,10-dihydroanthracen-2-yloxy)ethoxy)-3-(4-hydroxyphenyl)-1-oxopropan-2-aminium 2,2,2-trifluoroacetate* (**3z**). According to the general procedure **A**, compound **2** was treated with *N*-Boc-l-tyrosine and then purified by reverse phase flash chromatography to give compound **3z**: Yellow solid; yield, 23%; ^1^H-NMR δ 11.04 (s, 1H), 7.50 (d, *J* = 5.7 Hz, 2H), 7.34 (d, *J* = 8.0 Hz, 1H), 7.26–7.11 (m, 3H), 7.06 (t, *J* = 7.5 Hz, 1H), 6.96 (t, *J* = 7.5 Hz, 1H), 6.85 (d, *J* = 2.5 Hz, 1H), 4.56–4.18 (m, 5H), 3.27 (d, *J* = 6.5 Hz, 2H), 2.41 (s, 3H); ^13^C-NMR δ 189.93, 181.08, 169.46, 164.76, 164.25, 161.48, 148.59, 136.18, 134.83, 132.77, 126.88, 124.89, 124.23, 121.17, 120.60, 118.60, 117.87, 113.39, 111.56, 110.09, 107.80, 107.09, 106.24, 66.49, 63.71, 52.70, 26.20, 21.52; ^19^F-NMR δ − 73.47; ESI, *m*/*z*: 501.15 [M + H − CF_3_COOH]^+^.

*(R)-1-(2-(4,5-Dihydroxy-7-methyl-9,10-dioxo-9,10-dihydroanthracen-2-yloxy)ethoxy)-N-methyl-1-oxo-propan-2-aminium 2,2,2-trifluoroacetate* (**4a**). According to the general procedure **A**, compound **2** was treated with Boc-*N*-methyl-d-alanine and then purified by reverse phase flash chromatography to give compound **4a**: Yellow solid; yield, 48%; ^1^H-NMR δ 11.93 (brs, 2H), 9.18 (brs, 2H), 7.50 (d, *J* = 1.6 Hz, 1H), 7.29–7.12 (m, 2H), 6.91 (d, *J* = 2.5 Hz, 1H), 4.65–4.53 (m, 2H), 4.47 (d, *J* = 4.3 Hz, 2H), 4.20 (q, *J* = 7.1 Hz, 1H), 2.61 (s, 3H), 2.42 (s, 3H), 1.45 (d, *J* = 7.1 Hz, 3H); ^13^C-NMR δ 189.11, 179.90, 169.48, 164.62, 164.18, 161.36, 148.27, 134.02, 131.90, 123.98, 120.23, 112.58, 109.48, 107.57, 106.63, 66.56, 63.68, 55.23, 30.60, 21.38, 14.04; ^19^F-NMR δ − 73.51; ESI, *m*/*z*: 400.2 [M + H − CF_3_COOH]^+^.

*(S)-1-(2-(4,5-Dihydroxy-7-methyl-9,10-dioxo-9,10-dihydroanthracen-2-yloxy)ethoxy)-N-methyl-1-oxo-propan-2-aminium 2,2,2-trifluoroacetate* (**4b**). According to the general procedure **A**, compound **2** was treated with Boc-N-methyl-l-alanine and then purified by reverse phase flash chromatography to give compound **4b**: Yellow solid; yield, 45%; ^1^H-NMR δ 12.15 (brs, 1H), 11.96 (brs, 1H), 9.11 (s, 2H), 7.53 (d, *J* = 1.6 Hz, 1H), 7.24–7.17 (m, 2H), 6.93 (d, *J* = 2.6 Hz, 1H), 4.68–4.52 (m, 2H), 4.47 (t, *J* = 4.6 Hz, 2H), 4.19 (q, *J* = 7.2 Hz, 1H), 2.61 (s, 3H), 2.44 (s, 3H), 1.44 (d, *J* = 7.2 Hz, 3H); ^13^C-NMR δ 189.12, 179.88, 169.52, 164.55, 164.14, 161.31, 148.29, 134.03, 131.92, 123.87, 120.24, 112.58, 109.36, 107.49, 106.66, 66.54, 63.67, 55.29, 30.62, 21.40, 14.07; ^19^F-NMR δ − 73.48; ESI, *m*/*z*: 400.2 [M + H − CF_3_COOH]^+^.

General procedure **B** for preparation of compounds **5a** and **5b**

To a solution of compound **4a** or **4b** (100 mg, 0.25 mmol) and paraformaldehyde (37% aqueous solution, 62 mg, 0.76 mmol) in MeOH (15 mL) was added NaBH_3_CN (24 mg, 0.38 mmol) at 0 °C. After stirring 2 h at room temperature, the reaction was quenched by TFA (0.1 mL). The resulting reaction solution was used directly in purification by reverse phase flash chromatography with the following conditions: Column: Spherical C18, 20–40 μm, 330 g; Mobile Phase A: Water (plus 5 mM TFA); Mobile Phase B: ACN; Flow rate: 80 mL/min; Gradient: 5% B gradient in 10 min, 25% B–45% B gradient in 25 min; Detector: 254 nm. The fractions containing the desired product were collected at around 40% B and concentrated under reduced pressure to afford compounds **5a** or **5b** in 85% or 89% yield, respectively.

*(R)-1-(2-(4,5-Dihydroxy-7-methyl-9,10-dioxo-9,10-dihydroanthracen-2-yloxy)ethoxy)-N,N-dimethyl-1-oxo-propan-2-aminium 2,2,2-trifluoroacetate* (**5a**). Yellow solid; yield, 85%; ^1^H-NMR δ 12.17 (s, 1H), 11.95 (s, 1H), 10.23 (s, 1H), 7.53 (s, 1H), 7.25–7.14 (m, 2H), 6.93 (d, *J* = 2.5 Hz, 1H), 4.63–4.56 (m, 2H), 4.52–4.45 (m, 2H), 4.37 (q, *J* = 7.2 Hz, 1H), 2.80 (s, 6H), 2.43 (s, 3H), 1.48 (d, *J* = 7.2 Hz, 3H); ^13^C-NMR δ 189.60, 180.57, 168.53, 164.69, 164.23, 161.43, 148.51, 134.54, 132.41, 124.14, 120.49, 113.05, 109.81, 107.65, 107.00, 66.56, 63.87, 61.48, 21.52, 11.71; ^19^F-NMR δ − 73.59; ESI, *m*/*z*: 414.2 [M + H − CF_3_COOH]^+^.

*(S)-1-(2-(4,5-Dihydroxy-7-methyl-9,10-dioxo-9,10-dihydroanthracen-2-yloxy)ethoxy)-N,N-dimethyl-1-oxo-propan-2-aminium 2,2,2-trifluoroacetate* (**5b**). Yellow solid; yield, 89%; ^1^H-NMR δ 12.15 (s, 1H), 11.93 (s, 1H), 10.35 (s, 1H), 7.50 (s, 1H), 7.27–7.08 (m, 2H), 6.91 (d, *J* = 2.6 Hz, 1H), 4.59 (d, *J* = 4.5 Hz, 2H), 4.47 (t, *J* = 4.3 Hz, 2H), 4.39 (q, *J* = 7.1 Hz, 1H), 2.82 (s, 6H), 2.42 (s, 3H), 1.49 (d, *J* = 7.2 Hz, 3H); ^13^C-NMR δ 189.12, 179.87, 168.50, 164.53, 164.15, 161.32, 148.31, 134.04, 131.91, 123.87, 120.26, 112.57, 109.37, 107.48, 106.68, 66.47, 63.85, 61.55, 21.41, 11.82; ^19^F-NMR δ − 73.75; ESI, *m*/*z*: 414.2 [M + H − CF_3_COOH]^+^.

General procedure **C** for preparation of compounds **6a**–**6f**

To a mixture of emodin (10 mmol) in dry DMF (50 mL) were added Cs_2_CO_3_ (12 mmol) and hydroxybromides or iodides (30 mmol) at room temperature. After stirring for 36 h at 60 °C, the resulting mixture was evaporated under reduced pressure and then mixed with water (100 mL). The pH value of aqueous phase was adjusted to around 5 with 10% hydrochloric acid solution, extracted with dichloromethane (2 × 100 mL). The combined organic layer was washed with brine (200 mL), dried over anhydrous sodium sulfate and evaporated to dryness. The crude product was purified by silica gel column chromatography with 1%–10% ethyl acetate in petroleum to afford compounds **6a**–**6f**.

*1,8-Dihydroxy-3-(3-hydroxypropsoxy)-6-methylanthracene-9,10-dione* (**6a**). According to the general procedure **C**, emodin was treated with 3-iodopropan-1-ol and then purified by silica gel column chromatography to give compound **6a**: Brown solid; yield, 55%; ^1^H-NMR δ 12.14 (s, 1H), 11.96 (s, 1H), 7.51 (s, 1H), 7.21–7.13 (m, 2H), 6.85 (d, *J* = 2.5 Hz, 1H), 4.62 (t, *J* = 5.2 Hz, 1H), 4.23 (t, *J* = 6.3 Hz, 2H), 3.58 (q, *J* = 5.9 Hz, 2H), 2.43 (s, 3H), 1.95–1.87 (m, 2H); ^13^C-NMR δ 189.78, 180.98, 165.53, 164.32, 161.42, 148.44, 134.66, 132.69, 124.13, 120.51, 113.30, 109.60, 107.84, 106.83, 65.99, 57.00, 31.71, 21.49; ESI, *m*/*z*: 329.1 [M + H]^+^.

*1,8-Dihydroxy-3-(4-hydroxybutoxy)-6-methylanthracene-9,10-dione* (**6b**). According to the general procedure **C**, emodin was treated with 4-bromobutan-1-ol and then purified by silica gel column chromatography to give compound **6b**: Brown solid; yield, 47%; ^1^H-NMR δ 12.16 (s, 1H), 11.98 (s, 1H), 7.53 (s, 1H), 7.23–7.14 (m, 2H), 6.86 (d, *J* = 2.5 Hz, 1H), 4.49 (d, *J* = 5.5 Hz, 1H), 4.18 (t, *J* = 6.5 Hz, 2H), 3.47 (q, *J* = 6.0 Hz, 2H), 2.43 (s, 3H), 1.80 (t, *J* = 7.5 Hz, 2H), 1.58 (p, *J* = 6.7 Hz, 2H); ^13^C-NMR δ 189.83, 181.06, 165.53, 164.36, 161.44, 148.47, 134.71, 132.74, 124.16, 120.54, 113.35, 109.62, 107.92, 106.84, 68.81, 60.29, 28.76, 25.14, 21.52; ESI, *m*/*z*: 343.1 [M + H]^+^.

*1,8-Dihydroxy-3-(5-hydroxypentyloxy)-6-methylanthracene-9,10-dione* (**6c**). According to the general procedure **C**, emodin was treated with 5-bromopentan-1-ol and then purified by silica gel column chromatography to give compound **6c**: Brown solid; yield, 52%; ^1^H-NMR δ 12.15 (s, 1H), 11.96 (s, 1H), 7.51 (s, 1H), 7.17 (d, *J* = 16.9 Hz, 2H), 6.84 (s, 1H), 4.40 (s, 1H), 4.15 (t, *J* = 6.5 Hz, 2H), 3.45 (t, *J* = 6.5 Hz, 2H), 2.43 (s, 3H), 1.77 (d, *J* = 10.1 Hz, 2H), 1.48 (s, 4H); ^13^C-NMR δ 189.50, 180.55, 165.39, 164.26, 161.35, 148.28, 134.34, 132.41, 123.97, 120.36, 113.02, 109.30, 107.75, 106.58, 68.79, 60.57, 32.12, 28.20, 21.94, 21.44; ESI, *m*/*z*: 357.1 [M + H]^+^.

*1,8-Dihydroxy-3-(2-(2-hydroxyethoxy)ethoxy)-6-methylanthracene-9,10-dione* (**6d**). According to the general procedure **C**, emodin was treated with 2-(2-bromoethoxy)ethanol and then purified by silica gel column chromatography to give compound **6d**: Brown solid; yield, 38%; ^1^H-NMR δ 12.14 (s, 1H), 11.95 (s, 1H), 7.50 (d, *J* = 1.6 Hz, 1H), 7.19 (d, *J* = 1.6 Hz, 1H), 7.17 (d, *J* = 2.5 Hz, 1H), 6.88 (d, *J* = 2.5 Hz, 1H), 4.65 (s, 1H), 4.35–4.25 (m, 2H), 3.86–3.76 (m, 2H), 3.53 (d, *J* = 13.1 Hz, 4H), 2.42 (s, 3H); ^13^C-NMR δ 189.80, 180.93, 165.31, 164.29, 161.42, 148.47, 134.66, 132.67, 124.14, 120.52, 113.28, 109.71, 107.85, 106.95, 72.48, 68.49, 68.47, 60.22, 21.51; ESI, *m*/*z*: 359.2 [M + H]^+^.

*1,8-Dihydroxy-3-(2-(2-(2-hydroxyethoxy)ethoxy)ethoxy)-6-methylanthracene-9,10-dione* (**6e**). According to the general procedure **C**, emodin was treated with 2-(2-(2-bromoethoxy)ethoxy)ethanol and then purified by silica gel column chromatography to give compound **6e**: Brown solid; yield, 42%; ^1^H-NMR δ 12.01 (s, 1H), 11.82 (s, 1H), 7.34 (d, *J* = 1.6 Hz, 1H), 7.06 (d, *J* = 1.6 Hz, 1H), 7.00 (d, *J* = 2.6 Hz, 1H), 6.75 (d, *J* = 2.5 Hz, 1H), 4.57 (br, 1H), 4.33–4.14 (m, 2H), 3.86–3.73 (m, 2H), 3.70–3.55 (m, 4H), 3.53–3.41 (m, 4H), 2.36 (s, 3H); ^13^C-NMR δ 189.52, 180.51, 165.18, 164.21, 161.35, 148.36, 134.35, 132.37, 124.00, 120.39, 112.99, 109.44, 107.74, 106.79, 72.39, 70.01, 69.78, 68.50, 68.37, 60.22, 21.46; ESI, *m*/*z*: 403.2 [M + H]^+^.

*1,8-Dihydroxy-3-(2-(2-(2-(2-hydroxyethoxy)ethoxy)ethoxy)ethoxy)-6-methylanthracene-9,10-dione* (**6f**). According to the general procedure **C**, emodin was treated with 2-(2-(2-(2-bromoethoxy)ethoxy)ethoxy)ethanol and then purified by silica gel column chromatography to give compound **6f**: Brown solid; yield, 30%; ^1^H-NMR δ 12.13 (s, 1H), 11.94 (s, 1H), 7.49 (d, *J* = 1.6 Hz, 1H), 7.18 (t, *J* = 1.3 Hz, 1H), 7.15 (d, *J* = 2.5 Hz, 1H), 6.87 (d, *J* = 2.5 Hz, 1H), 4.57 (t, *J* = 5.5 Hz, 1H), 4.34–4.26 (m, 2H), 3.83–3.75 (m, 2H), 3.62 (dd, *J* = 6.1, 3.5 Hz, 2H), 3.58–3.45 (m, 8H), 3.42 (d, *J* = 5.1 Hz, 2H), 2.42 (s, 3H); ^13^C-NMR δ 189.74, 180.83, 165.29, 164.27, 161.41, 148.45, 134.59, 132.60, 124.11, 120.50, 113.21, 109.65, 107.85, 106.95, 72.34, 69.97, 69.84, 69.80, 69.76, 68.51, 68.41, 60.19, 21.50; ESI, *m*/*z*: 447.2 [M + H]^+^.

*(R)-1-(3-(4,5-Dihydroxy-7-methyl-9,10-dioxo-9,10-dihydroanthracen-2-yloxy)propoxy)-1-oxopropan-2-aminium 2,2,2-trifluoroacetate* (**7a**). According to the general procedure **A**, compound **6a** was treated with *N*-Boc-d-alanine and then purified by reverse phase flash chromatography to give compound **7a**: Yellow solid; yield, 70%; ^1^H-NMR δ 7.52 (d, *J* = 1.7 Hz, 1H), 7.23–7.16 (m, 2H), 6.88 (d, *J* = 2.5 Hz, 1H), 4.40–4.33 (m, 2H), 4.28 (t, *J* = 6.2 Hz, 2H), 4.15 (q, *J* = 7.1 Hz, 1H), 2.43 (s, 3H), 2.14 (p, *J* = 6.3 Hz, 2H), 1.42 (d, *J* = 7.1 Hz, 3H); ^13^C-NMR δ 189.85, 181.06, 169.89, 165.17, 164.26, 161.45, 148.52, 134.77, 132.73, 124.19, 120.55, 113.35, 109.86, 107.75, 107.00, 65.30, 62.31, 47.92, 27.59, 21.49, 15.68; ^19^F-NMR δ − 73.50; ESI, *m*/*z*: 400.2 [M + H − CF_3_COOH]^+^.

*(S)-1-(3-(4,5-Dihydroxy-7-methyl-9,10-dioxo-9,10-dihydroanthracen-2-yloxy)propoxy)-1-oxopropan-2-aminium 2,2,2-trifluoroacetate* (**7b**). According to the general procedure **A**, compound **6a** was treated with *N*-Boc-l-alanine and then purified by reverse phase flash chromatography to give compound **7b**: Yellow solid; yield, 68%; ^1^H-NMR δ 7.54 (s, 1H), 7.22–7.20 (m, 2H), 6.89 (s, 1H), 4.38–4.33 (m, 2H), 4.31 (t, *J* = 6.2 Hz, 2H), 4.15 (q, *J* = 7.1 Hz, 1H), 2.44 (s, 3H), 2.14 (p, *J* = 6.3 Hz, 2H), 1.41 (d, *J* = 7.1 Hz, 3H); ^13^C-NMR δ 189.19, 180.04, 170.06, 164.99, 164.19, 161.34, 148.26, 134.04, 132.04, 123.88, 120.28, 112.69, 109.21, 107.56, 106.56, 65.34, 62.38, 48.11, 27.71, 21.45, 15.78; ^19^F-NMR δ − 73.47; ESI, *m*/*z*: 400.2 [M + H − CF_3_COOH]^+^.

*(R)-1-(4-(4,5-Dihydroxy-7-methyl-9,10-dioxo-9,10-dihydroanthracen-2-yloxy)butoxy)-1-oxopropan-2-aminium 2,2,2-trifluoroacetate* (**7c**). According to the general procedure **A**, compound **6b** was treated with *N*-Boc-d-alanine and then purified by reverse phase flash chromatography to give compound **7c**: Yellow solid; yield, 65%; ^1^H-NMR δ 8.31 (brs, 2H), 7.56 (s, 1H), 7.21 (dd, *J* = 13.9, 1.9 Hz, 2H), 6.90(d, *J* = 2.5 Hz, 1H), 4.30–4.22 (m, 4H), 4.14 (q, *J* = 7.2 Hz, 1H), 2.45 (s, 3H), 1.92–1.76 (m, 4H), 1.41 (d, *J* = 7.2 Hz, 3H); ^13^C-NMR δ 189.85, 181.11, 170.03, 165.36, 164.33, 161.45, 148.50, 134.77, 132.76, 124.20, 120.54, 113.38, 109.76, 107.86, 106.92, 68.32, 65.24, 47.90, 24.73, 24.62, 21.49, 15.74; ^19^F-NMR δ − 73.44; ESI, *m*/*z*: 414.2 [M + H − CF_3_COOH]^+^.

*(S)-1-(4-(4,5-Dihydroxy-7-methyl-9,10-dioxo-9,10-dihydroanthracen-2-yloxy)butoxy)-1-oxopropan-2-aminium 2,2,2-trifluoroacetate* (**7d**). According to the general procedure **A**, compound **6b** was treated with *N*-Boc-l-alanine and then purified by reverse phase flash chromatography to give compound **7d**: Yellow solid; yield, 68%; ^1^H-NMR δ 7.53 (d, *J* = 1.6 Hz, 1H), 7.20 (dd, *J* = 13.9, 1.9 Hz, 2H), 6.88 (d, *J* = 2.5 Hz, 1H), 4.31–4.20 (m, 4H), 4.14 (q, *J* = 7.2 Hz, 1H), 2.44 (s, 3H), 1.92–1.76 (m, 4H), 1.41 (d, *J* = 7.2 Hz, 3H); ^13^C-NMR δ 189.24, 180.16, 170.12, 165.17, 164.23, 161.33, 148.23, 134.08, 132.13, 123.89, 120.27, 112.77, 109.16, 107.63, 106.48, 68.28, 65.24, 48.01, 24.84, 24.70, 21.45, 15.81; ^19^F-NMR δ − 73.47; ESI, *m*/*z*: 414.1 [M + H − CF_3_COOH]^+^.

*(R)-1-(5-(4,5-Dihydroxy-7-methyl-9,10-dioxo-9,10-dihydroanthracen-2-yloxy)pentyloxy)-1-oxopropan-2-aminium 2,2,2-trifluoroacetate* (**7e**). According to the general procedure **A**, compound **6c** was treated with *N*-Boc-d-alanine and then purified by reverse phase flash chromatography to give compound **7e**: Yellow solid; yield, 70%; ^1^H-NMR δ 12.00 (brs, 2H), 8.32 (brs, 3H), 7.54 (d, *J* = 1.6 Hz, 1H), 7.22 (s, 1H), 7.18 (d, *J* = 2.5 Hz, 1H), 6.88 (d, *J* = 2.5 Hz, 1H), 4.27–4.10 (m, 5H), 2.44 (s, 3H), 1.83–1.76 (m, 2H), 1.75–1.68 (m, 2H), 1.56–1.46 (m, 2H), 1.40 (d, *J* = 7.1 Hz, 3H); ^13^C-NMR δ 189.15, 180.02, 170.15, 165.20, 164.22, 161.32, 148.17, 133.96, 132.05, 123.84, 120.22, 112.69, 109.02, 107.59, 106.36, 68.55, 65.45, 48.03, 27.95, 27.74, 21.76, 21.44, 15.81; ^19^F-NMR δ − 73.46; ESI, *m*/*z*: 428.2 [M + H − CF_3_COOH]^+^.

*(S)-1-(5-(4,5-Dihydroxy-7-methyl-9,10-dioxo-9,10-dihydroanthracen-2-yloxy)pentyloxy)-1-oxopropan-2-aminium 2,2,2-trifluoroacetate* (**7f**). According to the general procedure **A**, compound **6c** was treated with *N*-Boc-l-alanine and then purified by reverse phase flash chromatography to give compound **7f**: Yellow solid; yield, 66%; ^1^H-NMR δ 11.97 (brs, 2H), 8.38 (brs, 3H), 7.52 (s, 1H), 7.21 (s, 1H), 7.17 (d, *J* = 2.5 Hz, 1H), 6.87 (d, *J* = 2.5 Hz, 1H), 4.27–4.10 (m, 5H), 2.43 (s, 3H), 1.83–1.76 (m, 2H), 1.75–1.68 (m, 2H), 1.56–1.46 (m, 2H), 1.40 (d, *J* = 7.1 Hz, 3H); ^13^C-NMR δ 189.72, 180.94, 170.02, 165.41, 164.31, 161.42, 148.42, 134.63, 132.65, 124.12, 120.48, 113.27, 109.60, 107.82, 106.79, 68.59, 65.42, 47.86, 27.81, 27.57, 21.67, 21.47, 15.72; ^19^F-NMR δ − 73.49; ESI, *m*/*z*: 428.2 [M + H − CF_3_COOH]^+^.

*(R)-1-(2-(2-(4,5-Dihydroxy-7-methyl-9,10-dioxo-9,10-dihydroanthracen-2-yloxy)ethoxy)ethoxy)-1-oxo-propan-2-aminium 2,2,2-trifluoroacetate* (**7g**). According to the general procedure **A**, compound **6d** was treated with *N*-Boc-d-alanine and then purified by reverse phase flash chromatography to give compound **7g**: Yellow solid; yield, 65%; ^1^H-NMR δ 9.46 (brs, 3H), 7.53 (s, 1H), 7.22 (s, 1H), 7.19 (s, 1H), 6.90 (s, 1H), 4.39–4.27 (m, 4H), 4.11 (q, *J* = 7.1 Hz, 1H), 3.82 (d, *J* = 4.6 Hz, 2H), 3.76 (d, *J* = 4.6 Hz, 2H), 2.44 (s, 3H), 1.38 (d, *J* = 7.1 Hz, 3H); ^13^C-NMR δ 189.83, 181.01, 170.27, 165.24, 164.29, 161.46, 148.52, 134.73, 132.71, 124.21, 120.54, 113.33, 109.81, 107.84, 107.01, 68.48, 68.36, 68.12, 64.78, 47.88, 21.52, 15.85; ^19^F-NMR δ − 73.46; ESI, *m*/*z*: 430.2 [M + H − CF_3_COOH]^+^.

*(S)-1-(2-(2-(4,5-Dihydroxy-7-methyl-9,10-dioxo-9,10-dihydroanthracen-2-yloxy)ethoxy)ethoxy)-1-oxo-propan-2-aminium 2,2,2-trifluoroacetate* (**7h**). According to the general procedure **A**, compound **6d** was treated with *N*-Boc-l-alanine and then purified by reverse phase flash chromatography to give compound **7h**: Yellow solid; yield, 67%; ^1^H-NMR δ 7.54 (d, *J* = 1.7 Hz, 1H), 7.22 (s, 1H), 7.20 (d, *J* = 2.6 Hz, 1H), 6.91 (d, *J* = 2.5 Hz, 1H), 4.40–4.26 (m, 4H), 4.12 (q, *J* = 7.2 Hz, 1H), 3.82 (t, *J* = 4.4 Hz, 2H), 3.75 (t, *J* = 4.6 Hz, 2H), 2.44 (s, 3H), 1.38 (d, *J* = 7.2 Hz, 3H); ^13^C-NMR δ 189.86, 181.05, 170.26, 165.25, 164.30, 161.46, 148.53, 134.76, 132.74, 124.21, 120.55, 113.36, 109.84, 107.85, 107.03, 68.48, 68.36, 68.11, 64.79, 47.88, 21.52, 15.85; ^19^F-NMR δ − 73.45; ESI, *m*/*z*: 430.1 [M + H − CF_3_COOH]^+^.

*(R)-1-(2-(2-(2-(4,5-Dihydroxy-7-methyl-9,10-dioxo-9,10-dihydroanthracen-2-yloxy)ethoxy)ethoxy)ethoxy)-1-oxopropan-2-aminium 2,2,2-trifluoroacetate* (**7i**). According to the general procedure **A**, compound **6e** was treated with *N*-Boc-d-alanine and then purified by reverse phase flash chromatography to give compound **7i**: Yellow solid; yield, 66%; ^1^H-NMR δ 9.64 (s, 3H), 7.53 (s, 1H), 7.22 (s, 1H), 7.19 (d, *J* = 2.5 Hz, 1H), 6.90 (d, *J* = 2.6 Hz, 1H), 4.29 (d, *J* = 15.1 Hz, 4H), 4.18–4.05 (m, 1H), 3.80 (d, *J* = 4.5 Hz, 2H), 3.67 (t, *J* = 4.5 Hz, 6H), 2.44 (s, 3H), 1.39 (d, *J* = 7.1 Hz, 3H); ^13^C-NMR δ 189.70, 180.82, 170.23, 165.25, 164.27, 161.42, 148.46, 134.59, 132.58, 124.14, 120.48, 113.20, 109.67, 107.80, 106.94, 69.93, 69.78, 68.53, 68.41, 68.04, 64.85, 47.87, 21.50, 15.82; ^19^F-NMR δ − 73.46; ESI, *m*/*z*: 474.2 [M + H − CF_3_COOH]^+^.

*(S)-1-(2-(2-(2-(4,5-Dihydroxy-7-methyl-9,10-dioxo-9,10-dihydroanthracen-2-yloxy)ethoxy)ethoxy)ethoxy)-1-oxopropan-2-aminium 2,2,2-trifluoroacetate* (**7j**). According to the general procedure **A**, compound **6e** was treated with *N*-Boc-l-alanine and then purified by reverse phase flash chromatography to give compound **7j**: Yellow solid; yield, 65%; ^1^H-NMR δ 9.53 (brs, 3H), 7.51 (d, *J* = 1.6 Hz, 1H), 7.20 (d, *J* = 1.6 Hz, 1H), 7.17 (d, *J* = 2.5 Hz, 1H), 6.88 (d, *J* = 2.5 Hz, 1H), 4.37–4.21 (m, 4H), 4.12 (q, *J* = 7.1 Hz, 1H), 3.84–3.76 (m, 2H), 3.67 (t, *J* = 4.6 Hz, 2H), 3.65–3.56 (m, 4H), 2.43 (s, 3H), 1.39 (d, *J* = 7.2 Hz, 3H); ^13^C-NMR δ 189.87, 181.06, 170.21, 165.31, 164.30, 161.46, 148.53, 134.76, 132.75, 124.21, 120.56, 113.37, 109.82, 107.86, 107.04, 69.92, 69.76, 68.53, 68.44, 68.02, 64.88, 47.86, 21.52, 15.80; ^19^F-NMR δ − 73.49; ESI, *m*/*z*: 474.2 [M + H − CF_3_COOH]^+^.

*(R)-1-(4,5-Dihydroxy-7-methyl-9,10-dioxo-9,10-dihydroanthracen-2-yloxy)-13-oxo-3,6,9,12-tetraoxapenta-decan-14-aminium 2,2,2-trifluoroacetate* (**7k**). According to the general procedure **A**, compound **6f** was treated with *N*-Boc-d-alanine and then purified by reverse phase flash chromatography to give compound **7k**: Yellow solid; yield, 66%; ^1^H-NMR δ 9.76 (brs, 3H), 7.46 (d, *J* = 1.6 Hz, 1H), 7.16 (s, 1H), 7.13 (d, *J* = 2.6 Hz, 1H), 6.85 (d, *J* = 2.5 Hz, 1H), 4.37–4.21 (m, 4H), 4.11 (q, *J* = 7.2 Hz, 1H), 3.85–3.76 (m, 2H), 3.68–3.60 (m, 4H), 3.59–3.52 (m, 6H), 2.41 (s, 3H), 1.40 (d, *J* = 7.1 Hz, 3H); ^13^C-NMR δ 189.81, 181.00, 170.17, 165.30, 164.28, 161.44, 148.49, 134.71, 132.71, 124.17, 120.52, 113.32, 109.73, 107.86, 107.02, 69.96, 69.75, 68.51, 68.42, 67.97, 64.84, 47.85, 21.50, 15.78; ^19^F-NMR δ − 73.54; ESI, *m*/*z*: 518.2 [M + H − CF_3_COOH]^+^.

*(S)-1-(4,5-Dihydroxy-7-methyl-9,10-dioxo-9,10-dihydroanthracen-2-yloxy)-13-oxo-3,6,9,12-tetraoxa-pentadecan-14-aminium 2,2,2-trifluoroacetate* (**7l**). According to the general procedure **A**, compound **6f** was treated with *N*-Boc-l-alanine and then purified by reverse phase flash chromatography to give compound **7l**: Yellow solid; yield, 65%; ^1^H-NMR δ 9.73 (brs, 3H), 7.53 (d, *J* = 1.7 Hz, 1H), 7.21 (s, 1H), 7.19 (d, *J* = 2.5 Hz, 1H), 6.89 (d, *J* = 2.6 Hz, 1H), 4.31 (q, *J* = 5.6, 4.2 Hz, 3H), 4.28–4.24 (m, 1H), 4.11 (q, *J* = 7.1 Hz, 1H), 3.80 (dd, *J* = 5.5, 3.2 Hz, 2H), 3.67–3.59 (m, 4H), 3.58–3.53 (m, 6H), 2.44 (s, 3H), 1.39 (d, *J* = 7.1 Hz, 3H); ^13^C-NMR δ 189.74, 180.89, 170.20, 165.28, 164.29, 161.44, 148.48, 134.63, 132.63, 124.16, 120.50, 113.24, 109.70, 107.84, 106.97, 69.98, 69.77, 68.52, 68.42, 67.99, 64.86, 47.86, 21.51, 15.80; ^19^F-NMR δ − 73.47; ESI, *m*/*z*: 518.2 [M + H − CF_3_COOH]^+^.

### 3.3. Cell Lines and Culture

HepG2 cells and MCF-7 cells were purchased from American Type Culture Collection (ATCC, Manassas, VA, USA). Cells were cultured in T75 flask (430641, Corning, New York, NY, USA), supplemented with high-glucose DMEM medium (Dulbecco’s modified Eagle’s medium, 11965-092, Gibco, New York, NY, USA) containing 10% Fetal bovine serum (FBS) and 1% penicillin (100 units/mL)-streptomycin (100 µg/mL) in a humidified incubator at 37 °C under 5% CO_2_ and 95% relative humidity (RH) atmosphere. The cells were harvested for metabolomics experiments using cell scrapers.

### 3.4. CellTiter-Glo^®^ Luminescent Cell Viability Assay

HepG2 cells and MCF-7 cells in 40 µL DMEM supplemented with 10% FBS were seeded at a density of 500 cells/well in 384-well cell culture plate (3570, Corning), respectively. After 24 h, the medium was substituted with fresh medium containing various sample solutions in DMSO (0.5%, v/v) at a dose range of 0.14–100 µM with 3-fold serial dilutions, and cells were cultured for another 48 h. Cells were cultured in medium without samples but 0.5% DMSO served as a control. All measurements were determined in triplicate for each treatment. After the sample treatment for 48 h, 20 µL CellTiter-Glo (G7573, Promega, Fitchburg, WI, USA) was added to each well containing cells and the contents were mixed for 2 min on an orbital shaker to induce cell lysis. The plates were then incubated at room temperature for 20 min to stabilize the luminescent signal. The luminescence was recorded on an Envision 2014 (Perkin Elmer, Waltham, MA, UAS). The IC_50_ values represented the samples’ concentrations required to inhibit 50% of cell proliferation and were calculated from a calibration curve by linear regression using GraphPad Prism 7.0 software (GraphPad Software, San Diego, CA, USA).

### 3.5. Apoptosis Analysis

Apoptosis was discriminated with the annexin V-FITC/PI test. Cells were seeded at 2 × 10^5^/well in 10% FBS-DMEM into 6-well plates and treated with compounds **7a** for 48 h. The cells were washed twice with cold phosphate buffered saline (PBS) and then resuspend in 1× Binding Buffer (0.1M Hepes/NaOH (pH 7.4), 1.4M NaCl, 25 mM CaCl_2_)) at a concentration of 1 × 10^5^ cells/mL. 100 μL of the solution (1 × 10^4^ cells) was transferred to a 5 mL culture tube, and 5 μL of FITC Annexin V (BD, Pharmingen, Franklin Lakes, NJ, USA) and 5 μL PI were added to each tube. The cells were gently resuspended by vortexing, and then were incubated for 30 min at RT (25 °C) in the dark. After incubation, 200 μL PBS was added to each tube. The analysis was performed with the system software (CellQuest; BD Biosciences, Franklin Lakes, NJ, USA). Analysis is as follows: lower left quadrant, viable cells (Annexin V−/PI−); lower right quadrant, early apoptotic cells (Annexin V+/PI−); upper right quadrant, late apoptotic cells (annexin V+/PI+); upper left quadrant, necrotic cells (annexin V−/PI+). The percentage of cells positive for PI and Annexin V-FITC was reported inside the quadrants.

### 3.6. Western Blot

Total cell lysates from cultured HepG2 cells treated with different concentrations of compound 7a for 48 h were obtained by lysing the cells in ice-cold RIPA buffer (1 PBS, 1% NP-40, 0.5% sodium deoxycholate and 0.1% SDS) containing 100 mg/mL PMSF, 5 mg/mL aprotinin, 5 mg/mL leupeptin, 5 mg/mL pepstatin and 100 mg/mL NaF. After centrifugation at 12,000 rpm for 10 min, the protein in the supernatant was quantified by the Bradford method (BIO-RAD, Hercules, CA, USA) using a Multimode Varioscan instrument (Thermo Fischer Scientific, Waltham, MA, USA). Twenty micrograms of protein per lane was applied in 12% SDS polyacrylamide gel. After electrophoresis, the protein was transferred to a polyvinylidine difluoride membrane (Amersham Biosciences, Marlborough, MA, USA). The membrane was blocked at room temperature for 2 h in TBST containing 5% blocking powder (Santa Cruz, Dallas, TX, USA). The membrane was washed with TBST for 5 min, and the primary antibody was added and incubated at 4 °C overnight (O/N). Bax (5023, CST, Danvers, MA, USA), Bcl-2 (15071, CST), cytochrome c (4280, CST), procaspase-9 (AB138412, Abcam, Cambridge, MA, USA), procaspase-3 (AB32150, Abcam), caspase-3 (66470-2-lg, Proteintech Group, Rosemont, IL, USA) and GAPDH (AB8245, Abcam) antibodies were employed. After three TBST washes, the membrane was incubated with the corresponding horseradish peroxidase-labeled secondary antibody (1:5000) (Santa Cruz) at room temperature for 2 h. Membranes were washed with TBST for 15 min five times and the protein blots were visualized with chemiluminescence reagent (Thermo Fischer Scientific Ltd.). The X-ray films were developed with a developer and fixed with fixer solution. The grey levels were analyzed using ImageQuant LAS 4000 system (GE, Marlborough, MA, USA).

### 3.7. Cell Cycle Analysis

The HepG2 cells were treated with indicated concentrations of compound **7a**. After incubation for 48 h, cells were washed twice with ice-cold PBS, fixed and permeabilized with ice cold 70% ethanol at −20 °C overnight. The cells were treated with 100 μg/mL RNase A at 37 °C for 30min, then washed with ice-cold PBS and finally stained with 1mg/mL PI in the dark at 4 °C for 30 min. The cellular DNA content for the cell cycle distribution analysis was performed with the system software (CellQuest; BD Biosciences), plotting at least 30,000 events per sample. The percentage of cells in the G1, S and G2 phases of the cell cycle were determined using the ModFit LT version 5.0 software package (Verity Software, Topsham, ME, USA).

### 3.8. Wound Healing Assay

HepG2 cells were grown in DMEM medium containing growth factors at a cell density of 1 × 10^5^ cells/mL for 24 h. A disposable 200 mL plastic pipette tip was used to scratch the monolayer of cells in a streaking motion. Compounds were added to the streaked cell culture at the indicated concentrations. The streaked cells were then cultured in serum-free medium for an additional 48 h and photographed. To quantify the experimental results, the % cell inhibitory rate was calculated by the equation: cell inhibitory rate (%) = (1 − D_drug_/D_control_) × 100%, where D_drug_ is the mean distance of cell migration in drug group and D_control_ is the mean distance of cell migration in control group. Pictures of the initial wounded monolayers were compared with the corresponding pictures of cells at the end of the incubation, and data were presented as mean ± SD from three independent experiments.

## 4. Conclusions

In conclusion, a novel series of emodin derivatives via the introduction of an amino acid were designed and synthesized. Their in vitro anti-proliferation tests revealed that these derivatives exhibited moderate to potent anti-proliferative activity against HepG2 cells and MCF-7 cells. Among these compounds, the most potent compound, **7a**, exhibited better selective anti-proliferative activity and specificity than emodin, displayed a significant effect in inducing cell cycle arrest at G0/G1 phase and inducing cell apoptosis in HepG2 cells via release of cytochrome c and subsequent activation of caspase-9 and caspase-3, which revealed that the possible molecular mechanism of **7a** apoptosis induction may mainly through the mitochondrial death pathway. Moreover, Compound **7a** also resulted in inhibition of HepG2 cells migration in the wound healing assay. These preliminary molecular mechanism results suggest that compound **7a** could be a promising lead compound for the development of novel antitumor drugs and has the potential for further investigations as an anti-cancer drug.

## Figures and Tables

**Table 1:** Anti-proliferative activities of compounds **3a–3z**.

Compounds	IC_50_ (μM) ^a^		Compounds	IC_50_ (μM)	
	HepG2	MCF-7		HepG2	MCF-7
**1(Emodin)**	43.87 ± 1.28	52.72 ± 2.22	**3n**	17.48 ± 0.59	18.51 ± 1.93
**2**	64.07 ± 2.37	86.39 ± 3.98	**3o**	17.64 ± 0.43	17.63 ± 0.29
**3a**	22.65 ± 0.78	66.86 ± 1.65	**3p**	18.99 ± 0.60	20.04 ± 1.82
**3b**	8.22 ± 0.13	8.99 ± 0.64	**3q**	25.94 ± 0.48	28.53 ± 1.74
**3c**	9.52 ± 0.22	10.15 ± 0.96	**3r**	19.28 ± 1.08	25.67 ± 0.61
**3d**	11.99 ± 1.19	33.97 ± 1.98	**3s**	18.46 ± 0.55	25.94 ± 1.09
**3e**	14.36 ± 1.09	14.19 ± 0.23	**3t**	26.09 ± 1.44	39.61 ± 1.97
**3f**	12.42 ± 0.35	15.97 ± 0.51	**3u**	14.32 ± 1.04	20.75 ± 0.32
**3g**	15.93 ± 0.33	16.07 ± 0.23	**3v**	11.41 ± 0.77	22.58 ± 0.90
**3h**	14.38 ± 0.27	12.78 ± 1.05	**3w**	31.48 ± 2.72	42.44 ± 1.16
**3i**	17.22 ± 0.20	17.19 ± 0.41	**3x**	16.19 ± 0.36	18.06 ± 0.32
**3j**	25.78 ± 5.28	29.78 ± 1.78	**3y**	20.34 ± 1.40	38.32 ± 1.92
**3k**	53.17 ± 8.7	> 100 ^b^	**3z**	14.07 ± 1.22	17.33 ± 0.26
**3l**	67.49 ± 0.56	>100	**Paclitaxel**	10.32 ± 0.26	4.89 ± 0.11
**3m**	>100	>100			

^a^ IC_50_ values are presented as mean values of three independent experiments done in triplicates; ^b^ when 50% inhibition could not reach at the highest concentration, then >100 μM was given.

**Table 2:** Anti-proliferative activities of compounds **4a**, **4b**, **5a and 5b**.

Compounds	IC_50_ (μM) ^a^		Compounds	IC_50_ (μM)	
	HepG2	MCF-7		HepG2	MCF-7
**4a**	13.69 ± 0.89	23.24 ± 0.33	**5a**	18.10 ± 2.79	25.23 ± 1.92
**4b**	39.95 ± 2.85	68.81 ± 2.25	**5b**	18.61 ± 2.07	16.22 ± 0.67

^a^ IC_50_ values are presented as mean values of three independent experiments done in triplicates.

**Table 3:** Anti-proliferative activities of compounds **7a**–**7l**.

Compounds	IC_50_ (μM) ^a^		Compounds	IC_50_ (μM)	
	HepG2	MCF-7		HepG2	MCF-7
**7a**	4.95 ± 0.14	5.92 ± 0.36	**7g**	15.21 ± 0.66	12.59 ± 0.46
**7b**	6.47 ± 0.21	6.47 ± 0.18	**7h**	11.38 ± 0.47	12.49 ± 0.44
**7c**	11.43 ± 1.69	12.72 ± 0.55	**7i**	28.72 ± 0.87	20.79 ± 1.87
**7d**	11.01 ± 1.47	14.88 ± 0.57	**7j**	31.88 ± 2.77	25.64 ± 0.58
**7e**	8.58 ± 0.77	9.11 ± 0.67	**7k**	37.20 ± 2.44	37.76 ± 2.44
**7f**	10.58 ± 0.97	15.79 ± 1.45	**7l**	60.65 ± 4.26	41.80 ± 2.24

^a^ IC_50_ values are presented as mean values of three independent experiments done in triplicates.

**Table 4:** Anti-proliferative activities of several more active compounds.

Compounds	IC_50_ (μM) ^a^		
	HepG2	MCF-7	L02
**Emodin**	43.87 ± 1.28	52.72 ± 2.22	22.52 ± 0.18
**3b**	8.22 ± 0.13	8.99 ± 0.64	33.56 ± 0.36
**3c**	9.52 ± 0.22	10.15 ± 0.96	37.29 ± 0.78
**7a**	4.95 ± 0.14	5.92 ± 0.36	13.96 ± 0.27
**7a**	6.47 ± 0.21	6.47 ± 0.18	18.53 ± 0.19
**7e**	8.58 ± 0.77	9.11 ± 0.67	27.65 ± 0.54
**7f**	10.58 ± 0.97	15.79 ± 1.45	24.32 ± 0.68

^a^ IC_50_ values are presented as mean values of three independent experiments.

**Table 5:** In vitro anti-proliferative activities of compound **7a** against normal cell line (L02).

Compounds	IC_50_ (μM)	Selectivity Index ^a^	
	L02	HepG2	MCF-7
**7a**	13.96 ± 0.27	2.82	2.36
**Emodin**	22.52 ± 0.18	0.51	0.43

^a^ SI: selective index (IC_50_ on normal cells/IC_50_ on cancer cells).

## Supplementary Materials

The following are available online at https://www.mdpi.com/1420-3049/24/5/884/s1, Tables S1–S4: Biochemistry analytical data from three independent experiments; Figures S1–S140: ^1^H-NMR, ^13^C-NMR and ^19^F-NMR spectra of these compounds.
