# Supplementary material for: Design and Synthesis of Novel Anti-Proliferative Emodin Derivatives and Studies on their Cell Cycle Arrest, Apoptosis Pathway and Migration
Source: Molecules. 2019 Mar 2;24(5):884. doi: 10.3390/molecules24050884 (PMC6429262; doi:10.3390/molecules24050884)
Supplement: Supplementary file 1 [file molecules-24-00884-s001.pdf]

# Supplementary Materials

## Design and synthesis of novel anti-proliferative emodin derivatives and studies on their cell cycle arrest, apoptosis pathway and migration

**Kun Yang [1], Ming-Ji Jin [2], Zhe-Shan Quan [1,*], Hu-Ri Piao [1,*]**

[1] Key Laboratory of Natural Resources and Functional Molecules of the Changbai Mountain, Affiliated Ministry of Education; Yanbian University College of Pharmacy, Yanji, Jilin Province 133002, PR China; yangkun815@163.com (K.Y.)

[2] Institute of Materia Medica, Chinese Academy of Medical Sciences, Beijing, 100050, China; mingji612@126.com (M.-J.J.)

* Correspondence: zsquan@ybu.edu.cn (Z.-S.Q.); piaohr@ybu.edu.cn (H.-R.P.); Tel.: +86-433-243-6149 (H.-R.P.)

### Table of contents

Table S1 ........................................................................................................S1
Table S2 ........................................................................................................S1
Table S3 ........................................................................................................S2
Table S4 ........................................................................................................S2
NMR Spectra .................................................................................................S3

**Table S1.** The apoptosis rates of HepG2 cells treated with different concentration compound **7a**.

| Apoptosis stage | Apoptosis percentage (%)[a] | | | |
|---|---|---|---|---|
| | 0 μM | 2.5 μM | 5 μM | 10 μM |
| Early apoptosis | 1.85 ± 0.13 | 2.91 ± 0.45 | 15.93 ± 0.76 | 37.73 ± 0.97 |
| Late apoptosis | 3.62 ± 0.07 | 3.69 ± 0.48 | 7.51 ± 0.68 | 14.63 ± 1.17 |
| Overall apoptosis | 5.47 ± 0.06 | 6.59 ± 0.93[b] | 23.450 ± 1.07[c] | 52.00 ± 1.41[c] |

[a] Data are expressed as means ± SD of the percentages of apoptotic cells from three independent experiments.
[b] Statistical significance is determined by two-tailed Student t-test: $p < 0.01$.
[c] Statistical significance is determined by two-tailed Student t-test: $p < 0.001$.

**Table S2.** Western blot analysis effect of compound **7a** in HepG2 cells.

| Antibody | Relative expression level[a] | | | |
|---|---|---|---|---|
| | 0 μM | 2.5 μM | 5 μM | 10 μM |
| Bax | 1.011 ± 0.113 | 1.180 ± 0.046[b] | 1.347 ± 0.081[c] | 1.593 ± 0.112[d] |
| Bcl-2 | 1.052 ± 0.107 | 0.685 ± 0.025[d] | 0.312 ± 0.012[d] | 0.268 ± 0.022[d] |
| cytochrome c | 1.023 ± 0.078 | 1.407 ± 0.015[d] | 1.663 ± 0.110[d] | 2.220 ± 0.387[d] |
| procaspase-3 | 1.106 ± 0.033 | 0.510 ± 0.132[d] | 0.317 ± 0.031[d] | 0.170 ± 0.053[d] |
| procaspase-9 | 1.050 ± 0.013 | 0.600 ± 0.082[d] | 0.297 ± 0.058[d] | 0.223 ± 0.029[d] |
| Full length procaspase-3 | 0.852 ± 0.019 | 0.78 ± 0.012[d] | 0.220 ± 0.019[d] | 0.152 ± 0.009[d] |
| Cleaved Caspase-3 | 0.045 ± 0.007 | 0.056 ± 0.011[d] | 0.143 ± 0.013[d] | 0.185 ± 0.026[d] |

[a] Data are expressed as means ± SD of the percentages of apoptotic cells from three independent experiments.
[b] Statistical significance is determined by two-tailed Student t-test: $p < 0.05$.
[c] Statistical significance is determined by two-tailed Student t-test: $p < 0.01$.
[d] Statistical significance is determined by two-tailed Student t-test: $p < 0.001$.

**Table S3.** The statistical results of cell cycle distribution after treatment of compound **7a** in HepG2 cells.

| Cell cycle stage | Cell cycle distribution (%)[a] in different concentrations | | | |
|---|---|---|---|---|
| | 0 μM | 0.25 μM | 0.5 μM | 1 μM |
| G0/G1 | 33.73 ± 1.93 | 44.18 ± 1.79[b] | 56.49 ± 1.40[c] | 63.96 ± 2.42[c] |
| S | 48.10 ± 2.52 | 38.25 ± 1.65[b] | 19.21 ± 1.33[c] | 14.52 ± 1.43[c] |
| G2/M | 18.30 ± 1.64 | 17.55 ± 0.90[d] | 24.81 ± 1.79[d] | 22.22 ±1.16[d] |

[a] Data are expressed as means ± SD of the percentages of apoptotic cells from three independent experiments.
[b] Statistical significance is determined by two-tailed Student t-test: $p < 0.05$.
[c] Statistical significance is determined by two-tailed Student t-test: $p < 0.001$.
[d] No significance.

**Table S4.** Compound **7a** inhibited migration of HepG2 cells.

| Concentration | 0 μM | 0.25 μM | 0.5 μM | 1 μM |
|---|---|---|---|---|
| Inhibition (%)[a] | 0 | 12.58 ± 1.69[b] | 23.71 ± 0.59[b] | 40.18 ± 2.92[b] |

[a] Data are expressed as means ± SD of cell inhibitory rate (%) for the migration of HepG2 cells from three independent experiments.
[b] Statistical significance is determined by two-tailed Student t-test: $p < 0.001$.

**NMR Spectra**

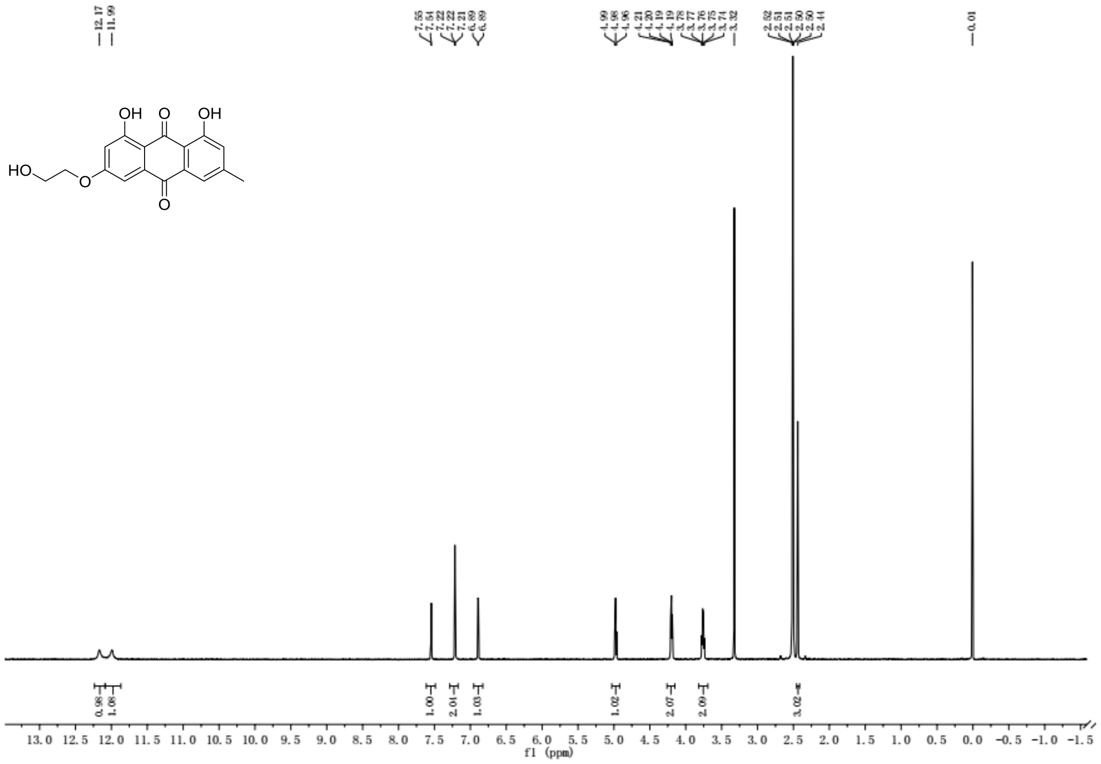

**Figure S1.** $^{1}H$ NMR (400 MHz, DMSO–$d_6$) spectrum of compound **2**.

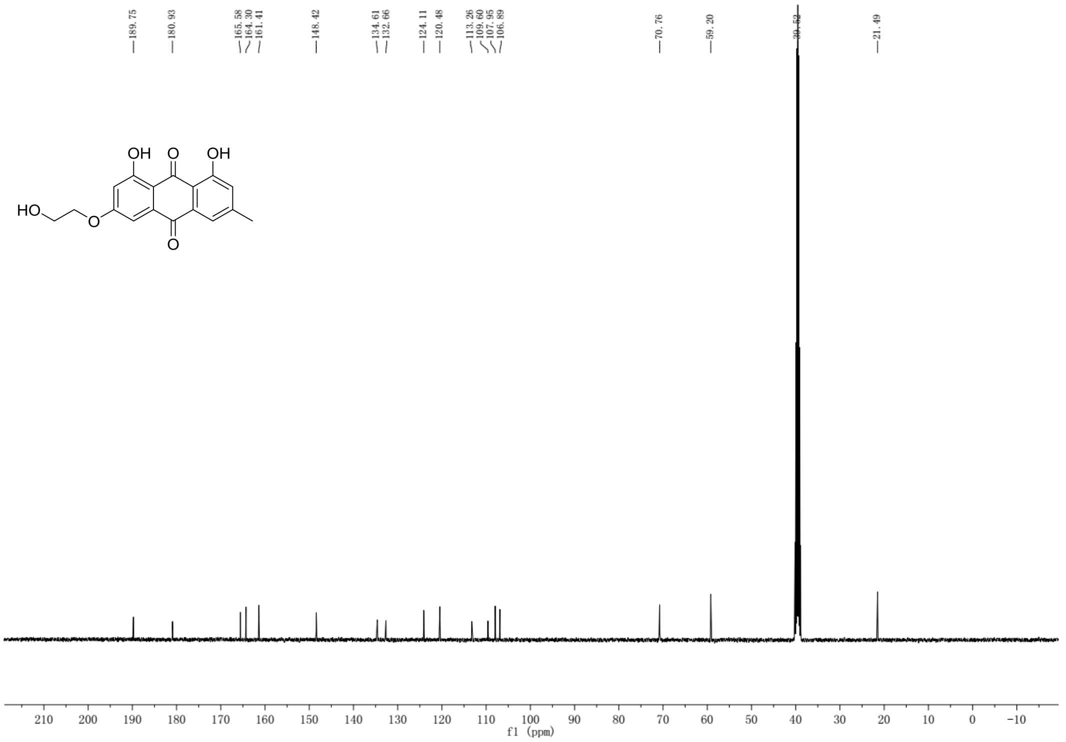

**Figure S2.** $^{13}C$ NMR (101 MHz, DMSO–$d_6$) spectrum of compound **2**.

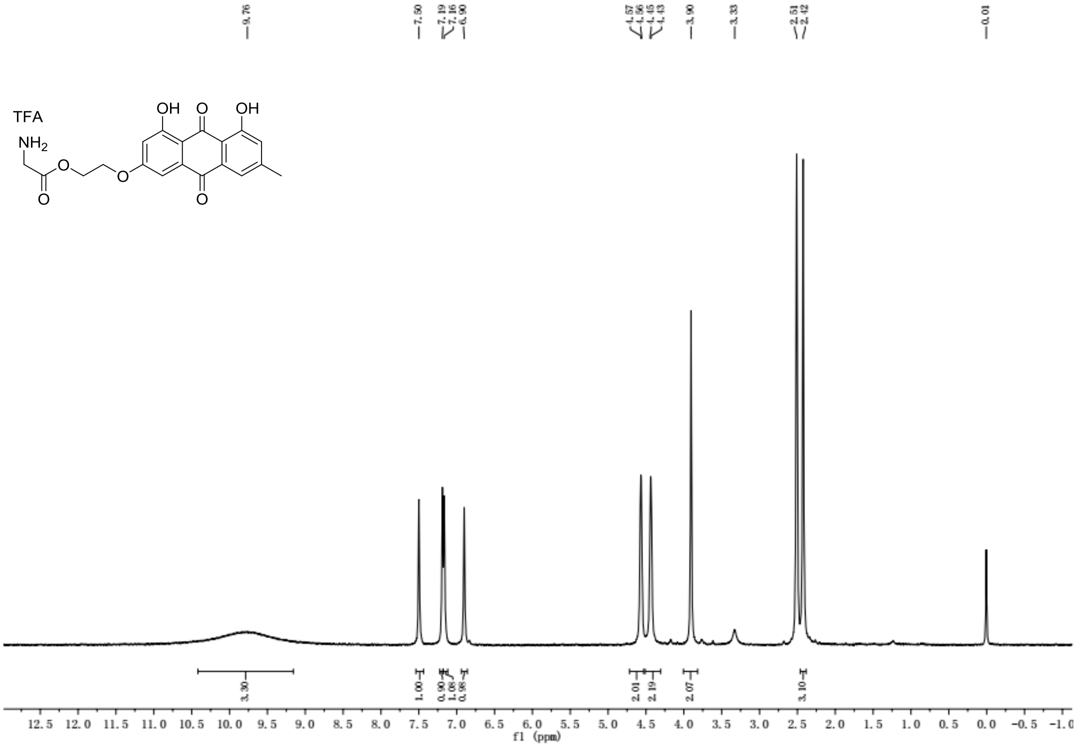


**Figure S3.** $^{1}$H NMR (400 MHz, DMSO–$d_6$) spectrum of compound **3a**.

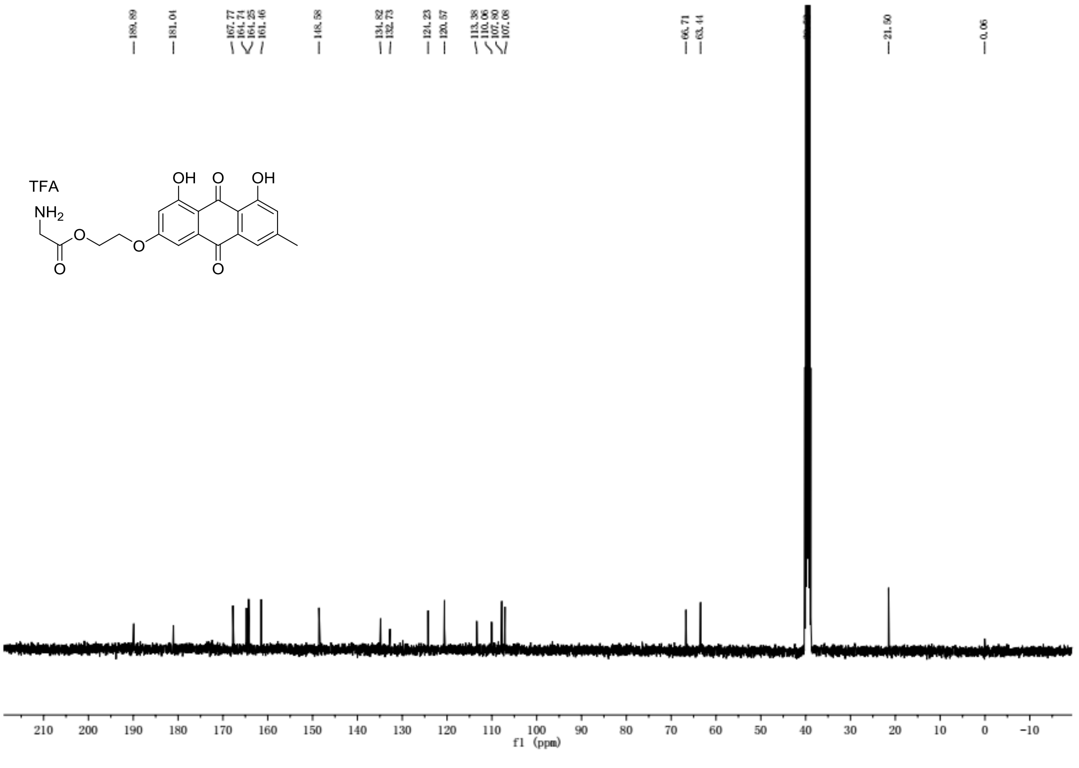


**Figure S4.** $^{13}$C NMR (101 MHz, DMSO–$d_6$) spectrum of compound **3a**.

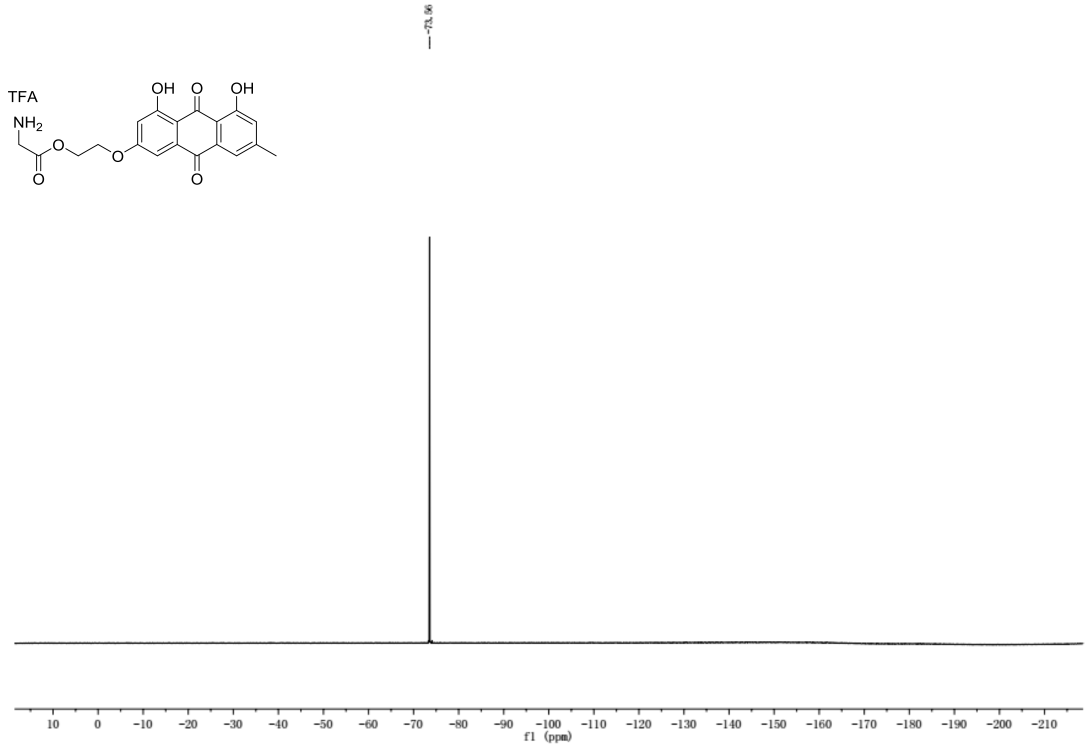

**Figure S5.** $^{19}F$ NMR (376 MHz, DMSO–$d_6$) spectrum of compound **3a**.

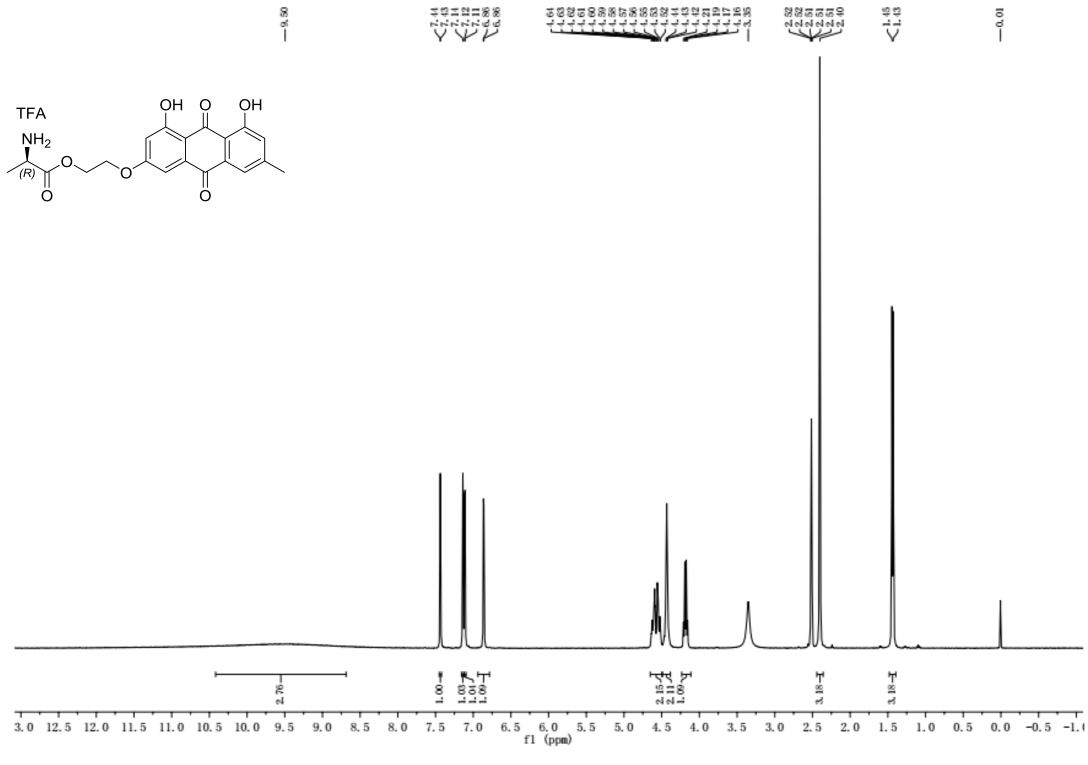

**Figure S6.** $^{1}H$ NMR (400 MHz, DMSO–$d_6$) spectrum of compound **3b**.

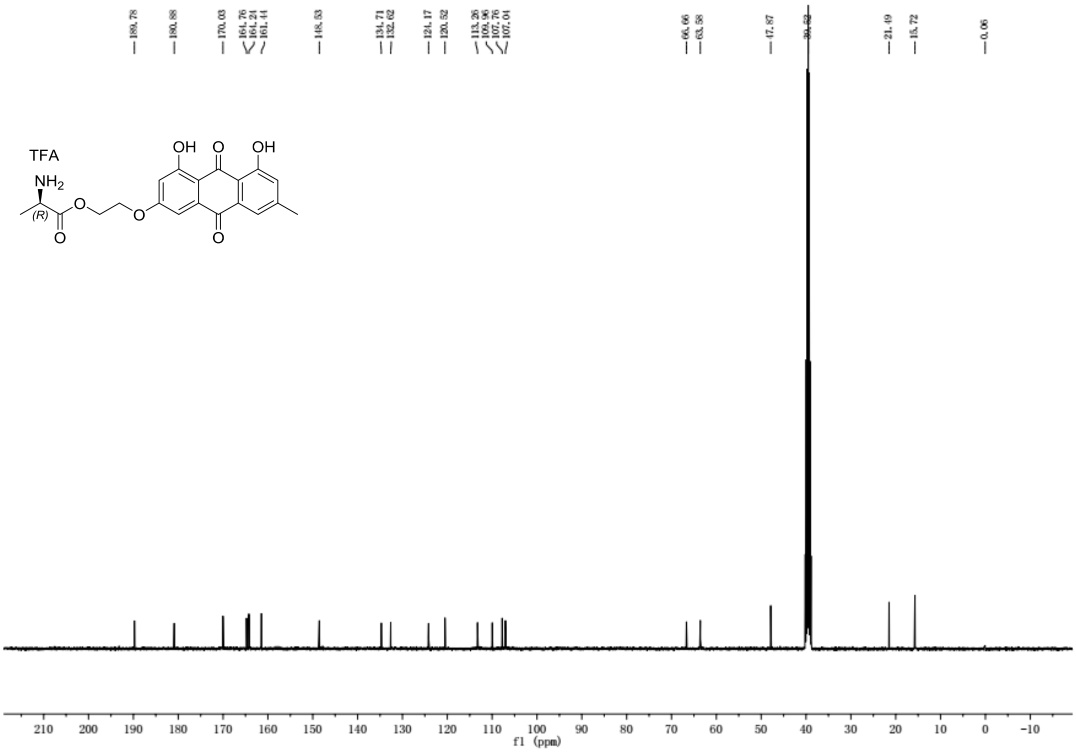


**Figure S7.** $^{13}$C NMR (101 MHz, DMSO–$d_6$) spectrum of compound **3b**.

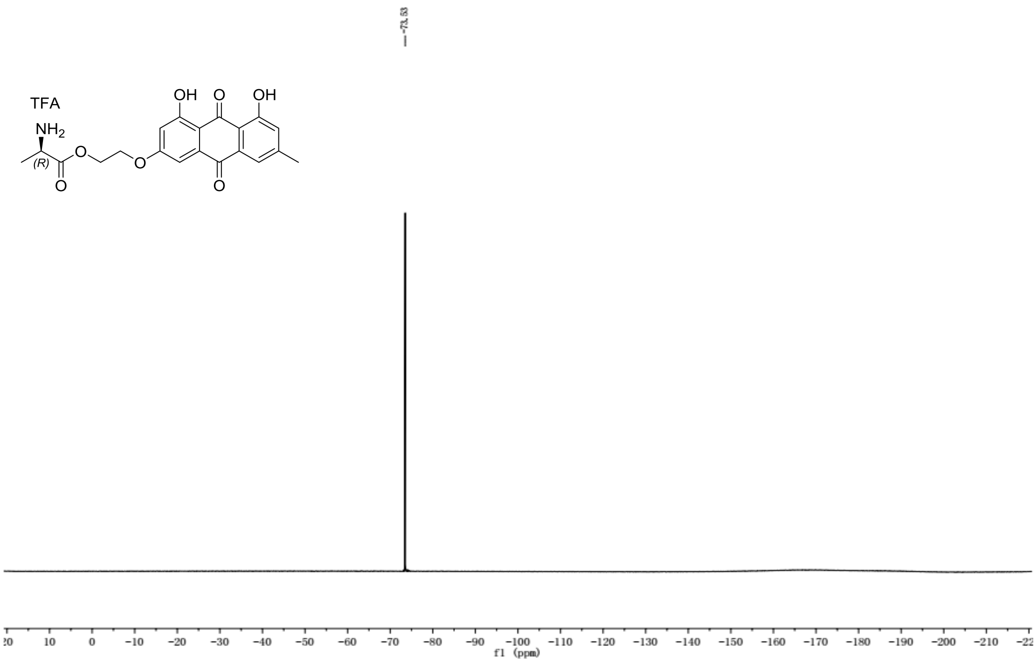


**Figure S8.** $^{19}$F NMR (376 MHz, DMSO–$d_6$) spectrum of compound **3b**.

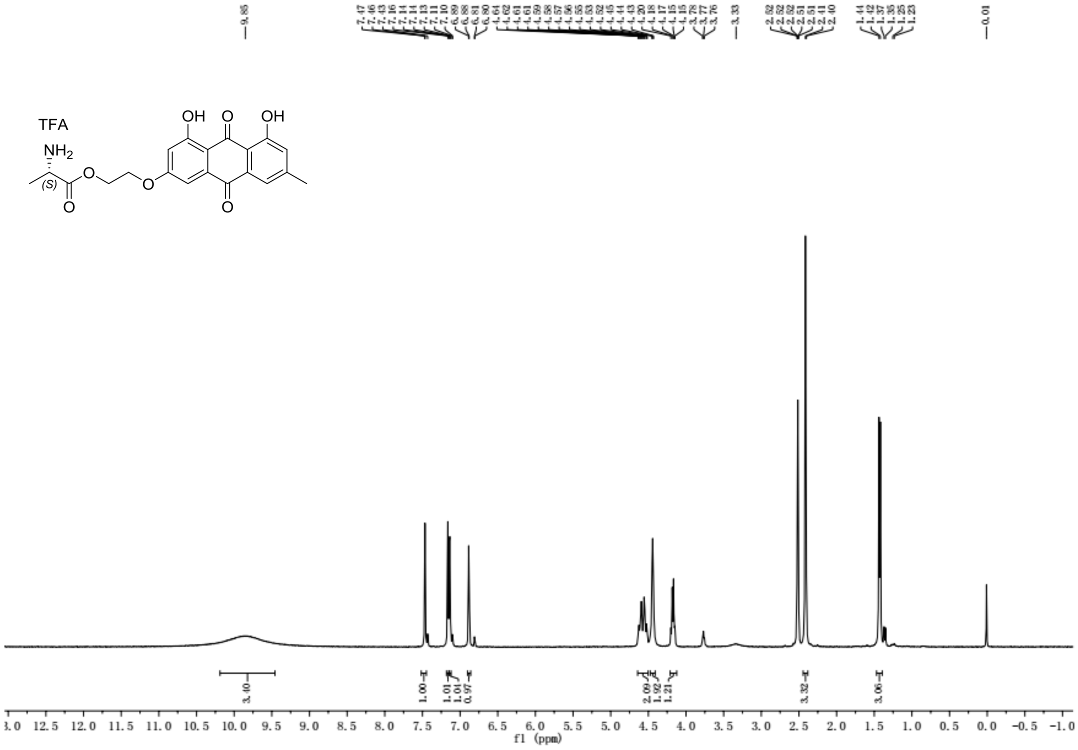

**Figure S9.** $^{1}$H NMR (400 MHz, DMSO–$d_6$) spectrum of compound **3c**.

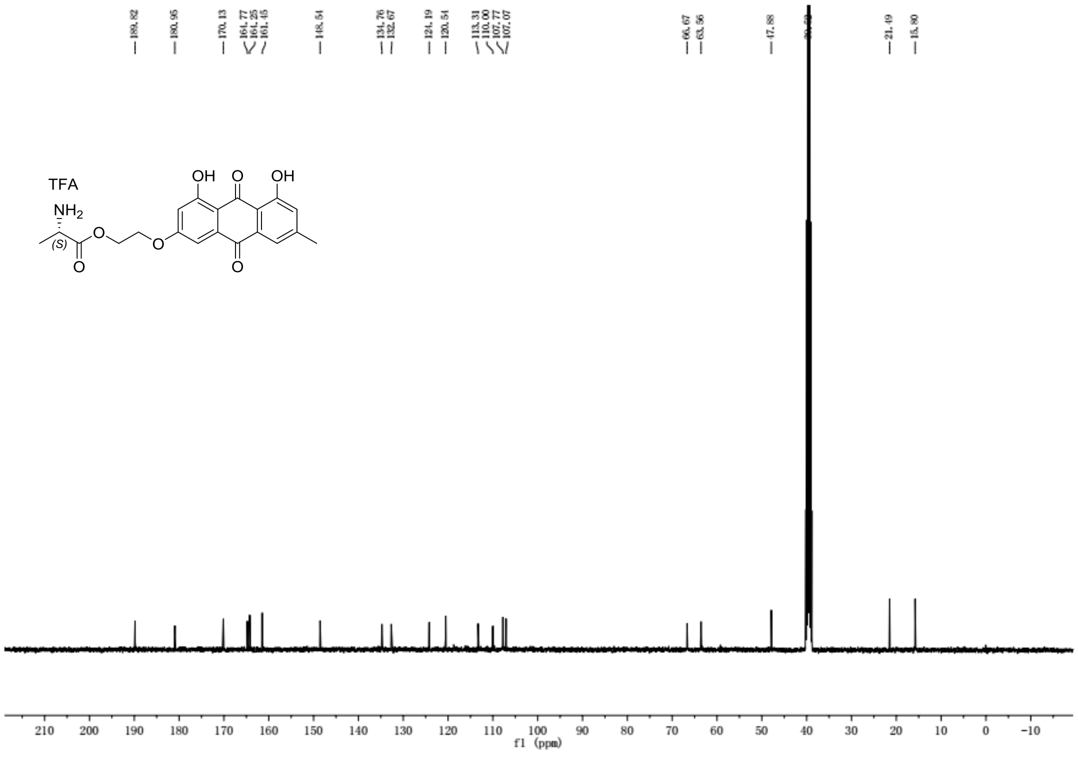

**Figure S10.** $^{13}$C NMR (101 MHz, DMSO–$d_6$) spectrum of compound **3c**.

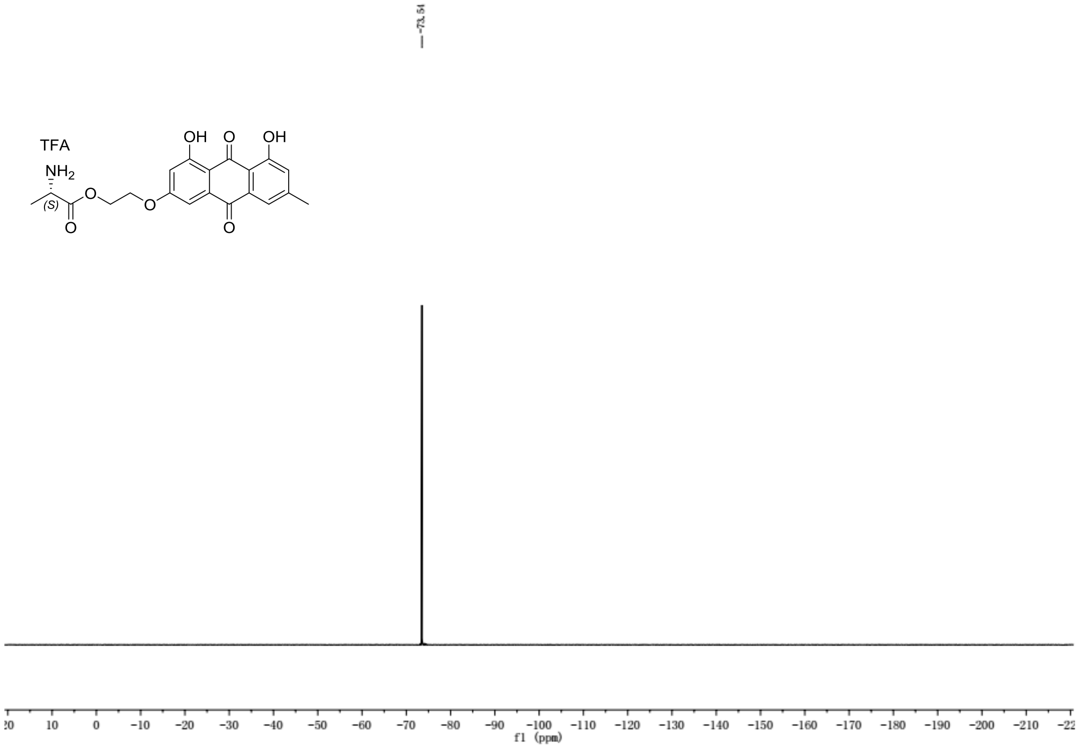


**Figure S11.** $^{19}$F NMR (376 MHz, DMSO–$d_6$) spectrum of compound **3c**.

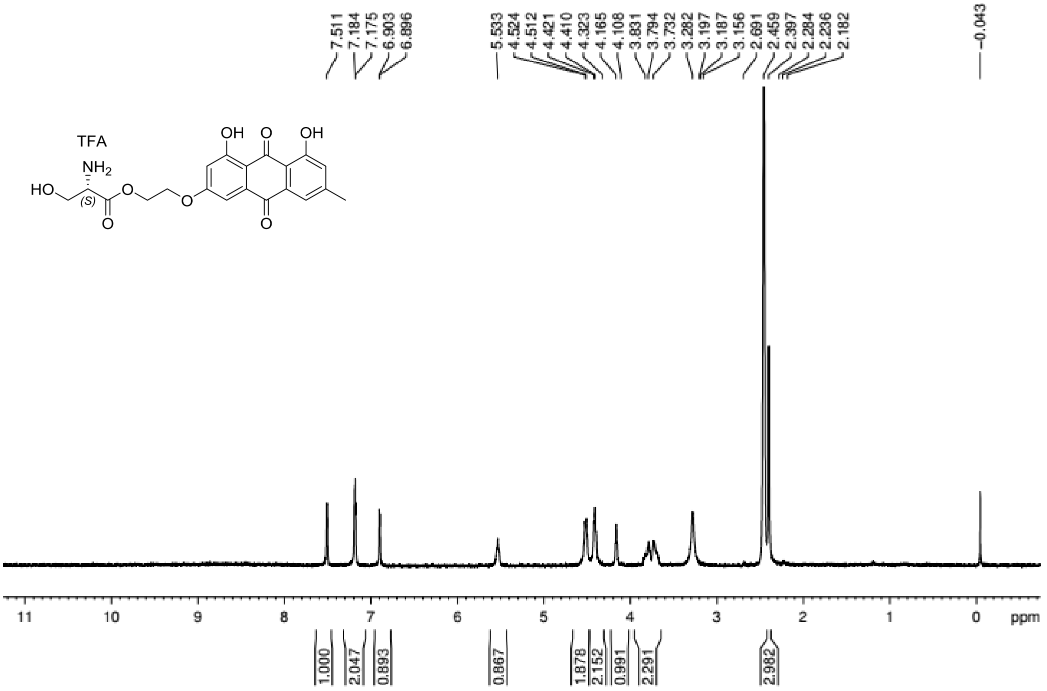


**Figure S12.** $^{1}$H NMR (400 MHz, DMSO–$d_6$) spectrum of compound **3d**.

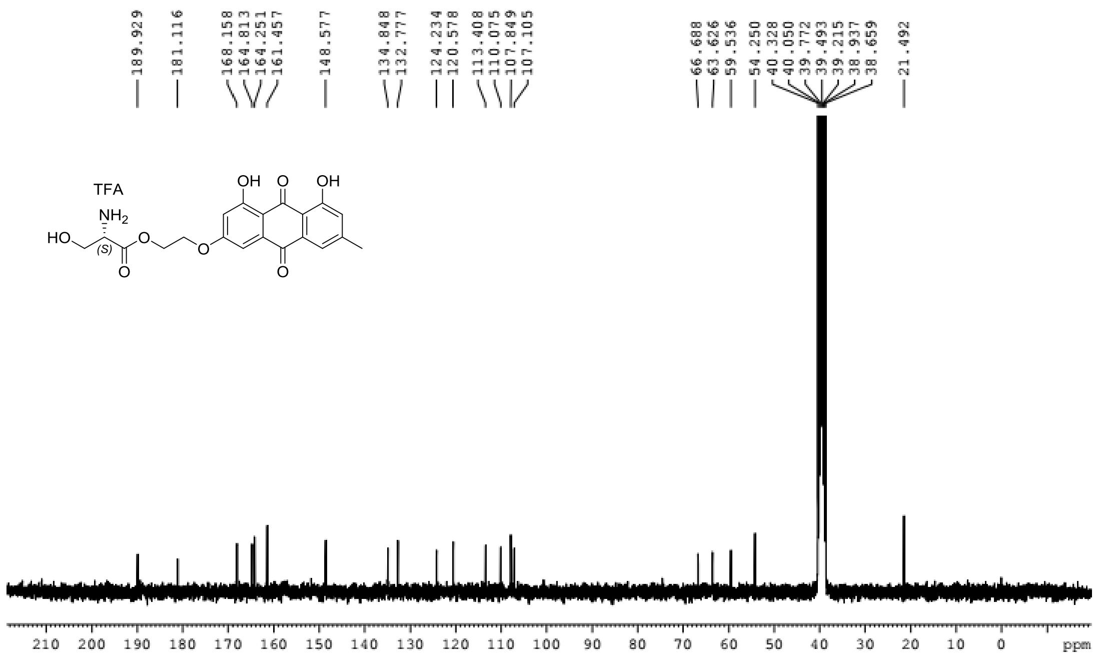

**Figure S13.** $^{13}C$ NMR (101 MHz, DMSO–$d_6$) spectrum of compound **3d**.

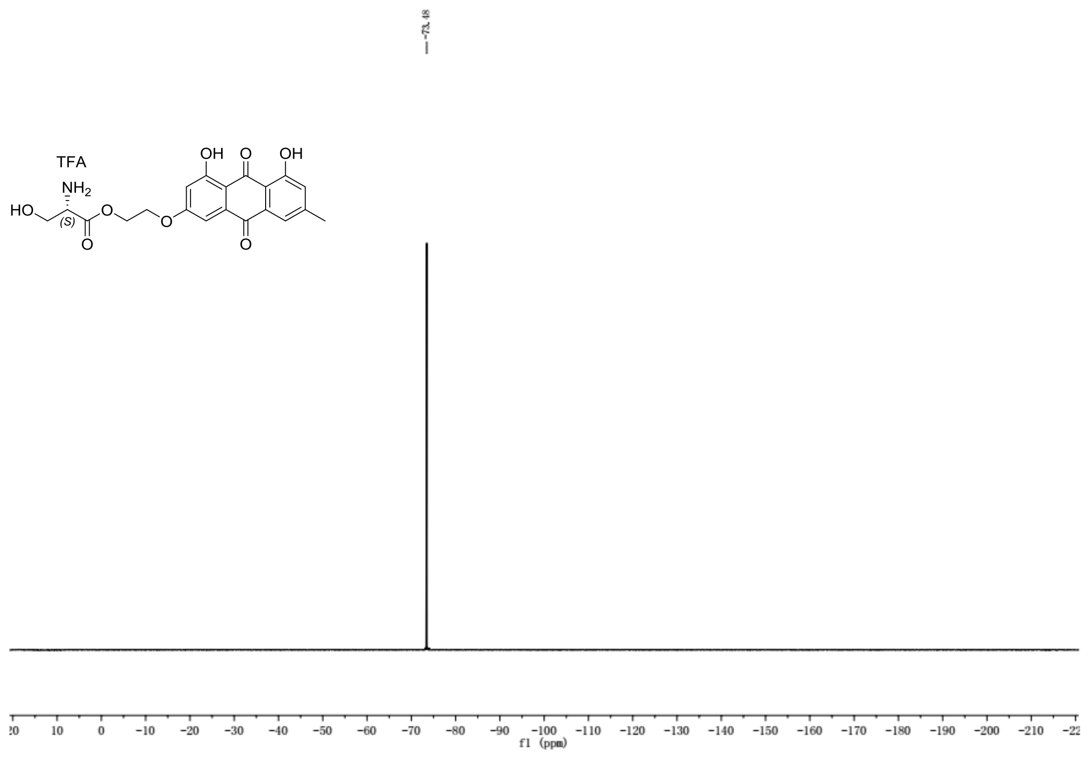

**Figure S14.** $^{19}F$ NMR (376 MHz, DMSO–$d_6$) spectrum of compound **3d**.

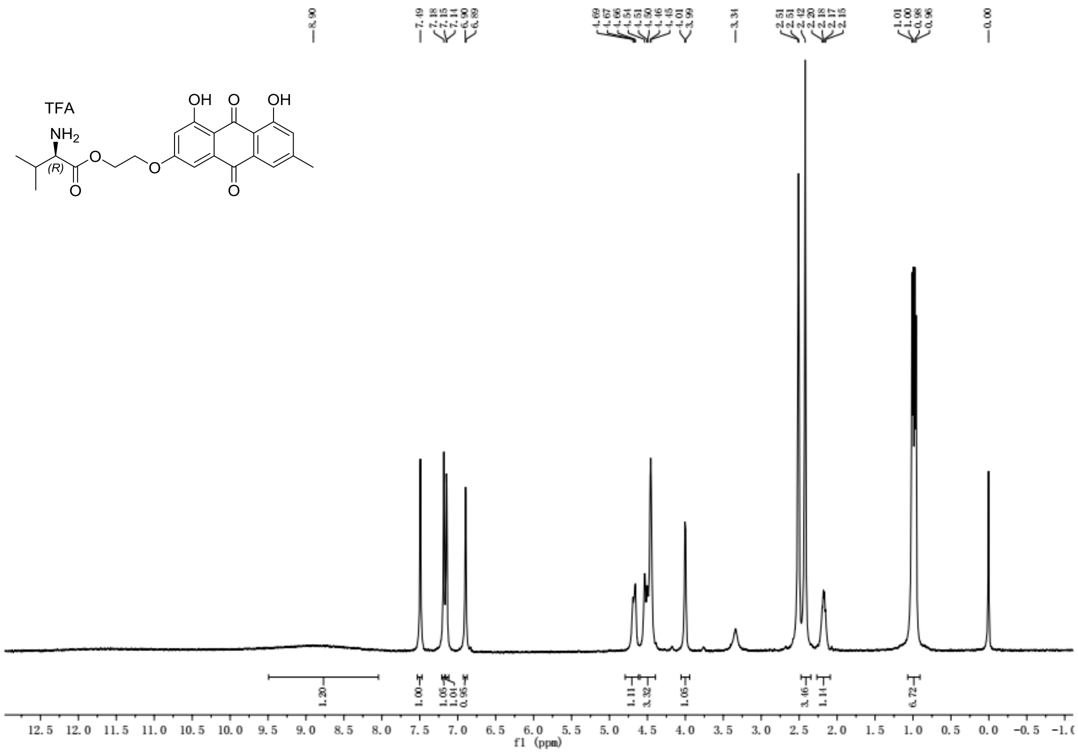

**Figure S15.** $^{1}$H NMR (400 MHz, DMSO–$d_6$) spectrum of compound **3e**.

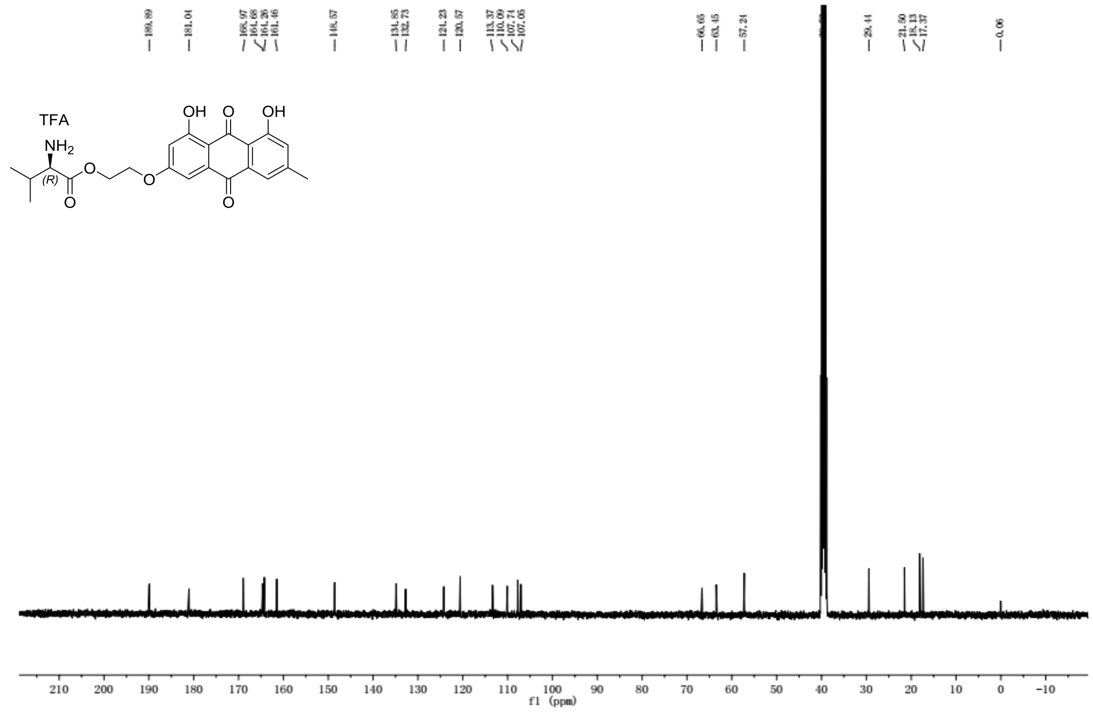

**Figure S16.** $^{13}$C NMR (101 MHz, DMSO–$d_6$) spectrum of compound **3e**.

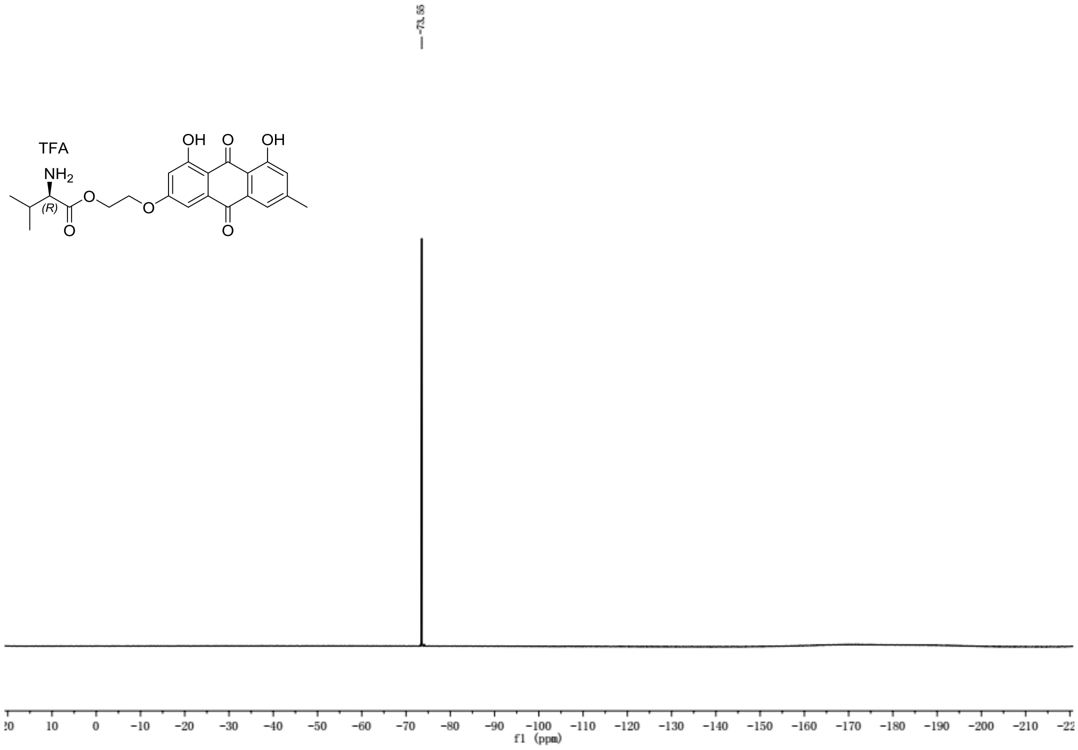

**Figure S17.** $^{19}$F NMR (376 MHz, DMSO–$d_6$) spectrum of compound **3e**.

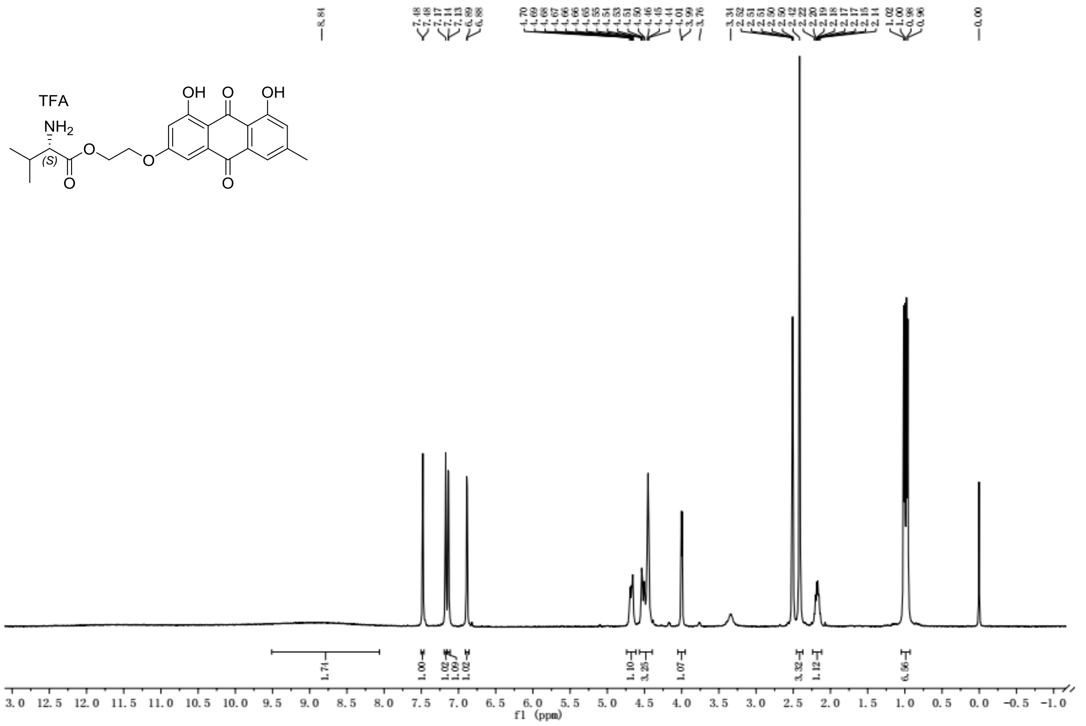

**Figure S18.** $^{1}$H NMR (400 MHz, DMSO–$d_6$) spectrum of compound **3f**.

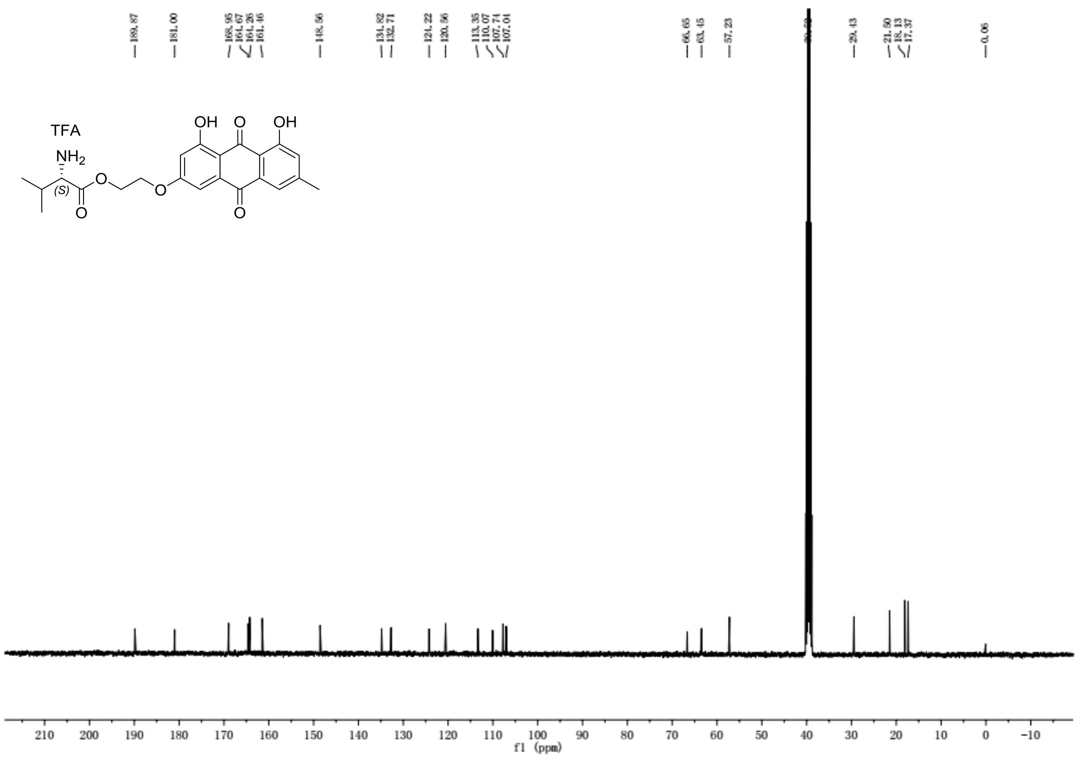

**Figure S19.** $^{13}$C NMR (101 MHz, DMSO–$d_6$) spectrum of compound **3f**.

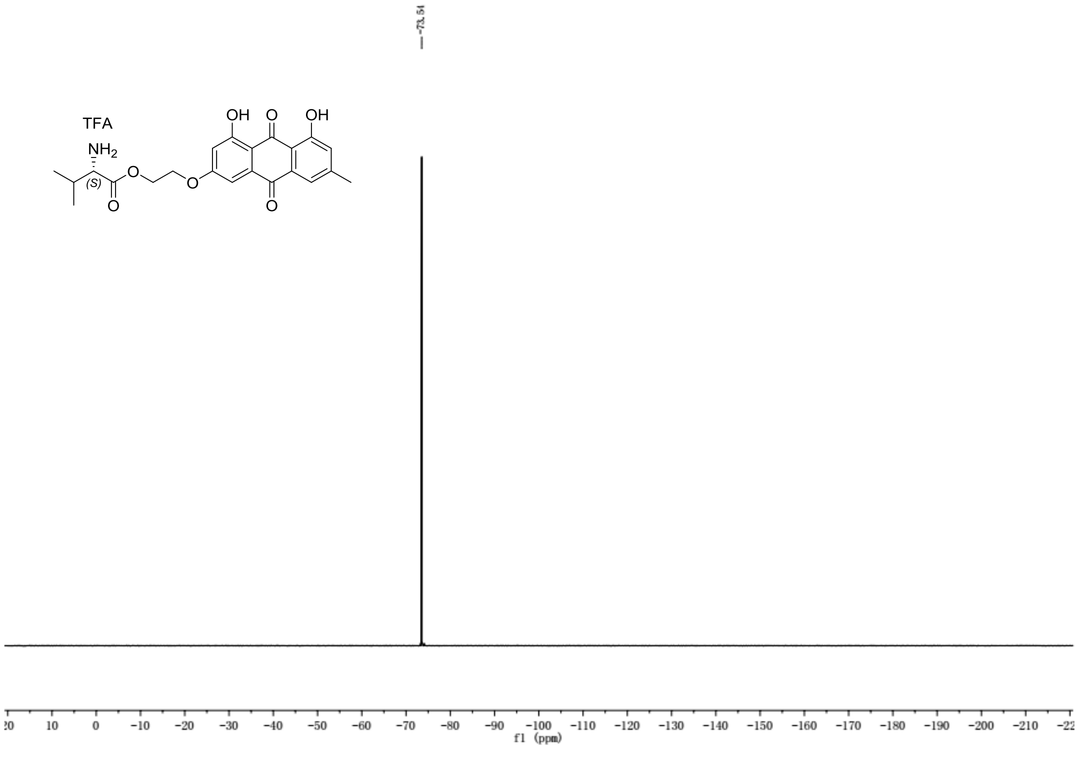

**Figure S20.** $^{19}$F NMR (376 MHz, DMSO–$d_6$) spectrum of compound **3f**.

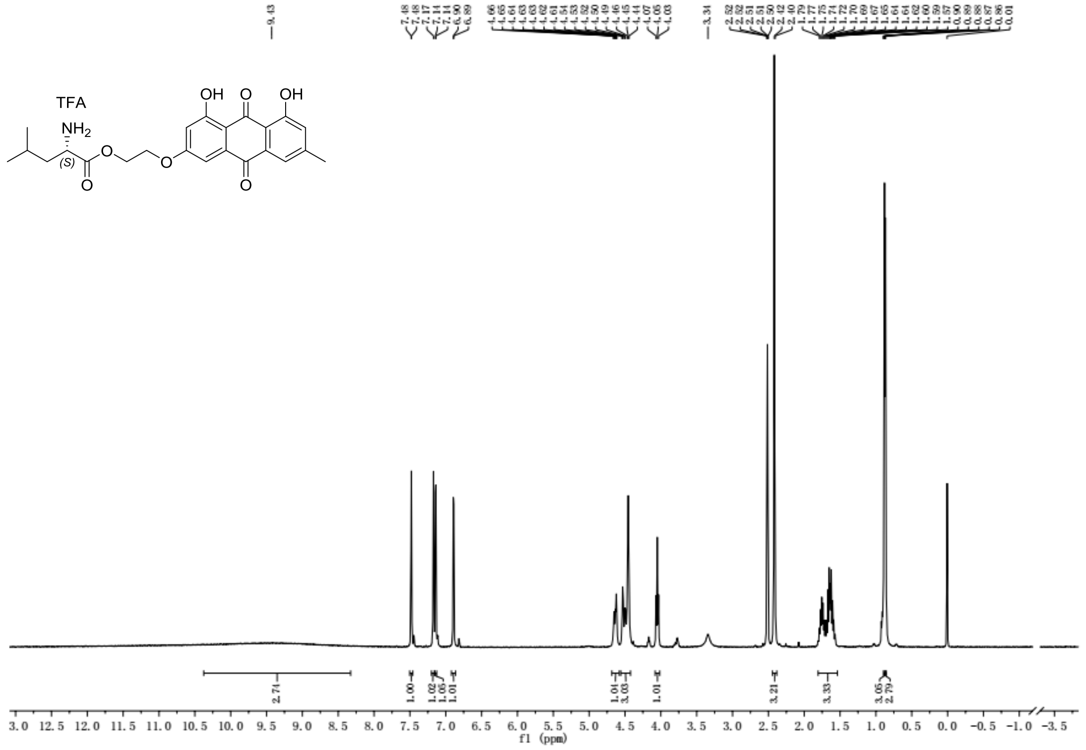

**Figure S21.** $^{1}$H NMR (400 MHz, DMSO–$d_6$) spectrum of compound **3g**.

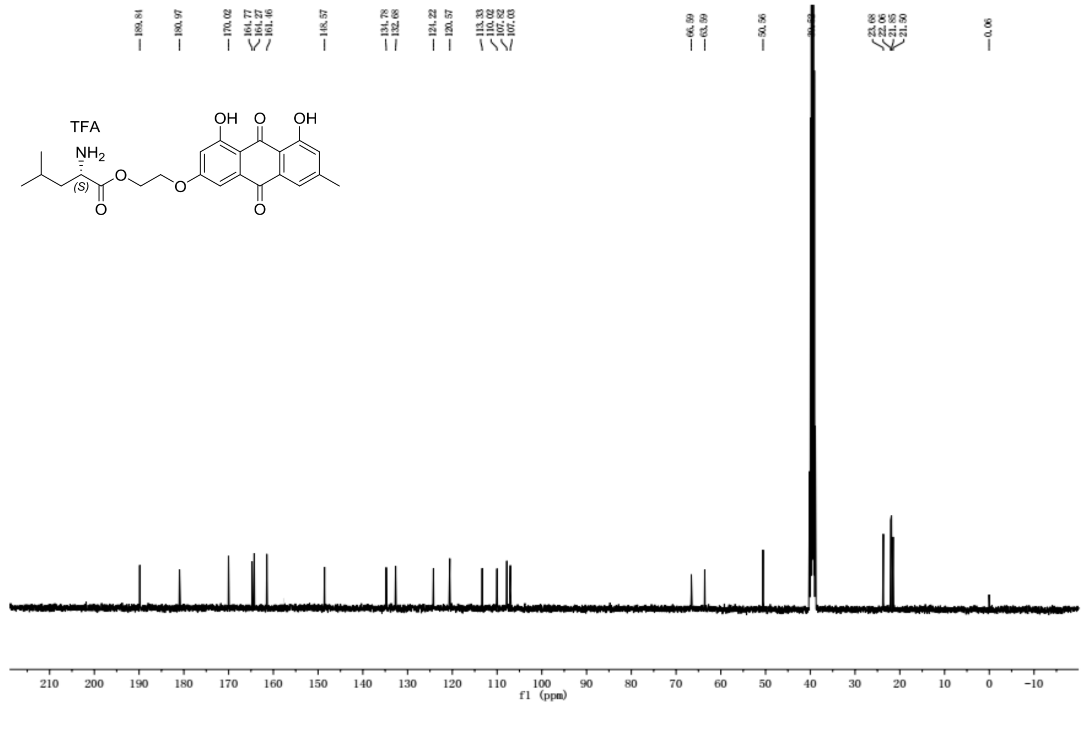

**Figure S22.** $^{13}$C NMR (101 MHz, DMSO–$d_6$) spectrum of compound **3g**.

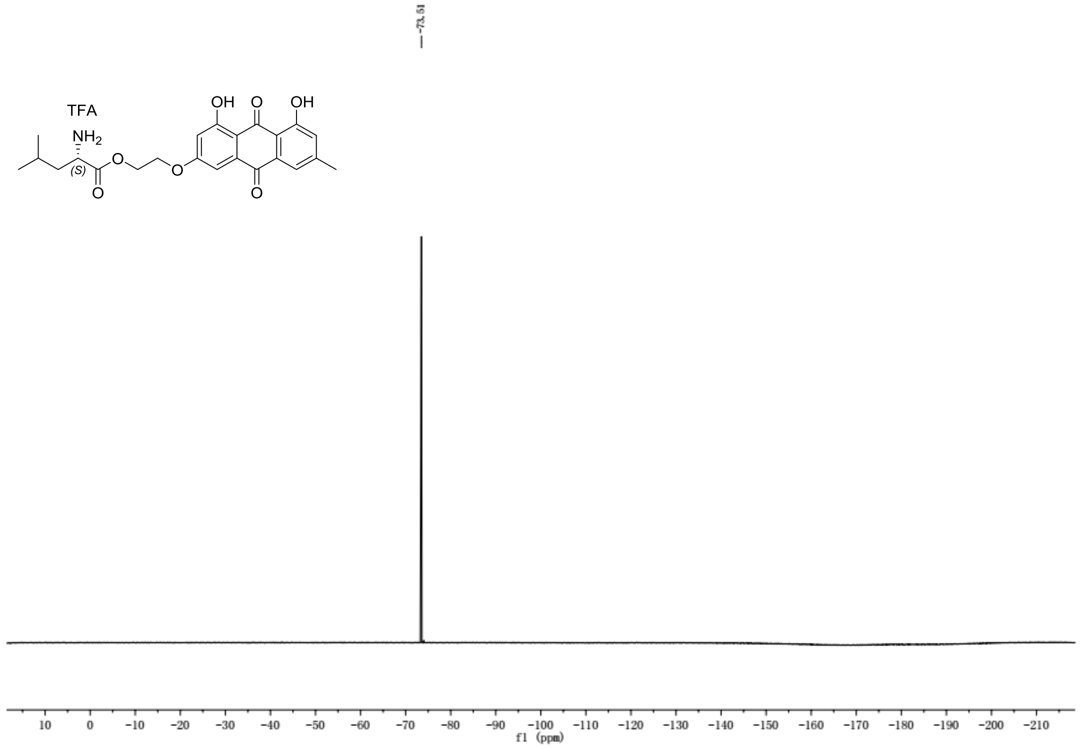


**Figure S23.** $^{19}$F NMR (376 MHz, DMSO–$d_6$) spectrum of compound **3g**.

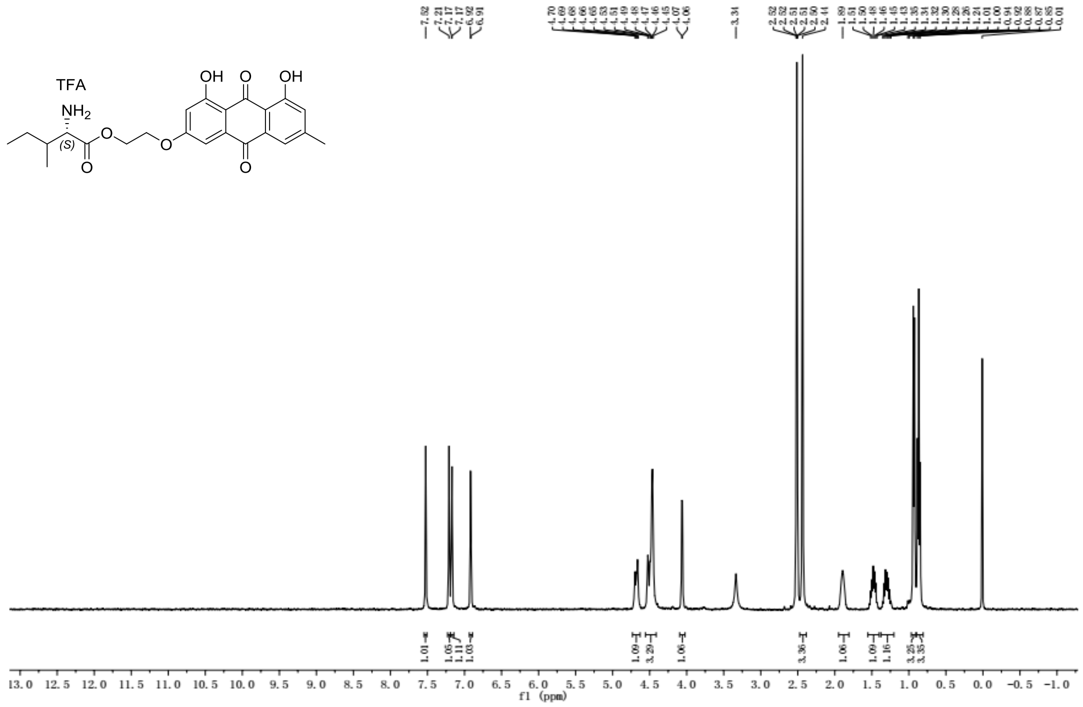


**Figure S24.** $^{1}$H NMR (400 MHz, DMSO–$d_6$) spectrum of compound **3h**.

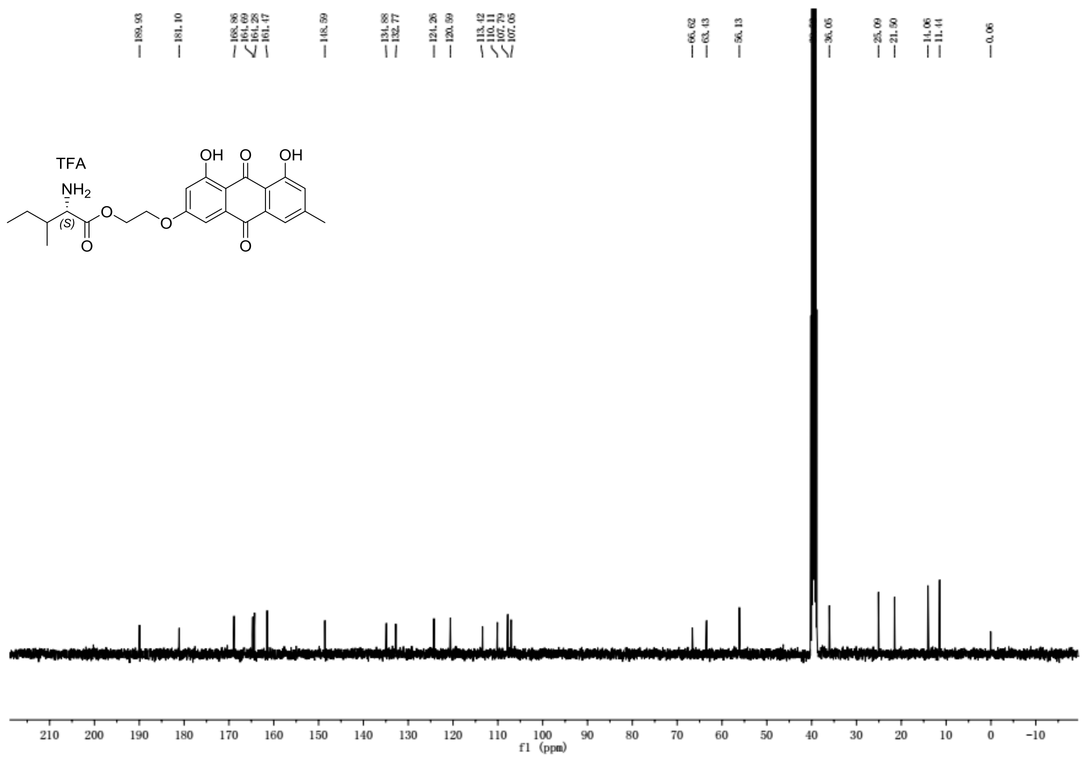

**Figure S25.** $^{13}C$ NMR (101 MHz, DMSO–$d_6$) spectrum of compound **3h**.

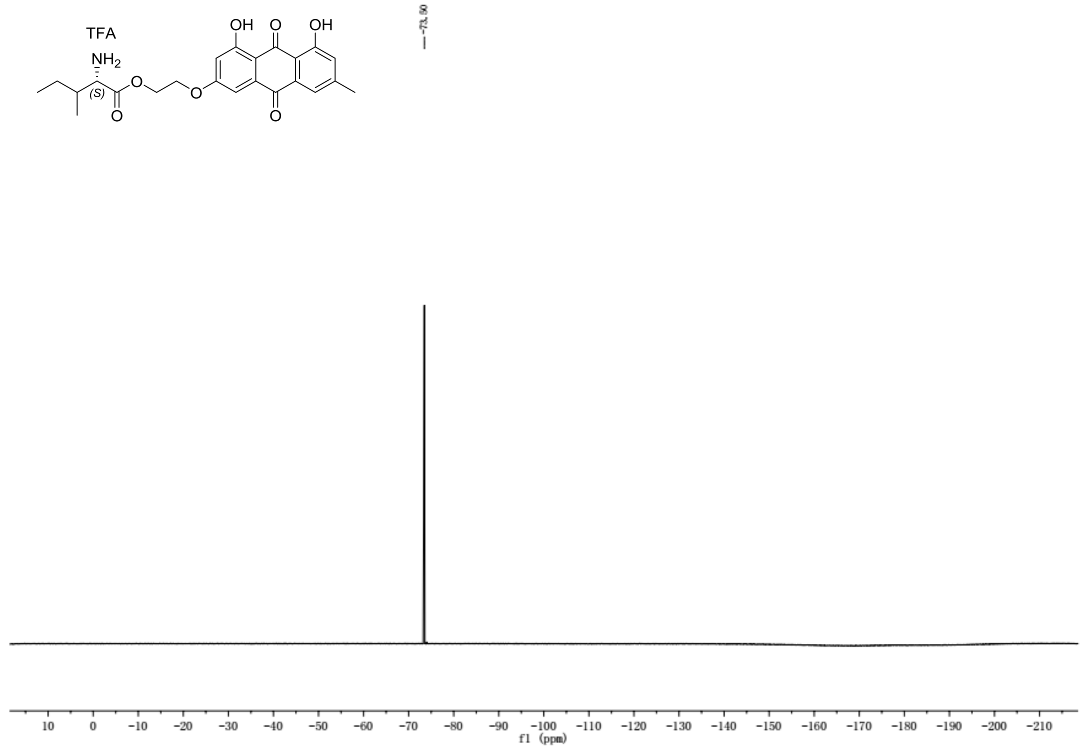

**Figure S26.** $^{19}F$ NMR (376 MHz, DMSO–$d_6$) spectrum of compound **3h**.

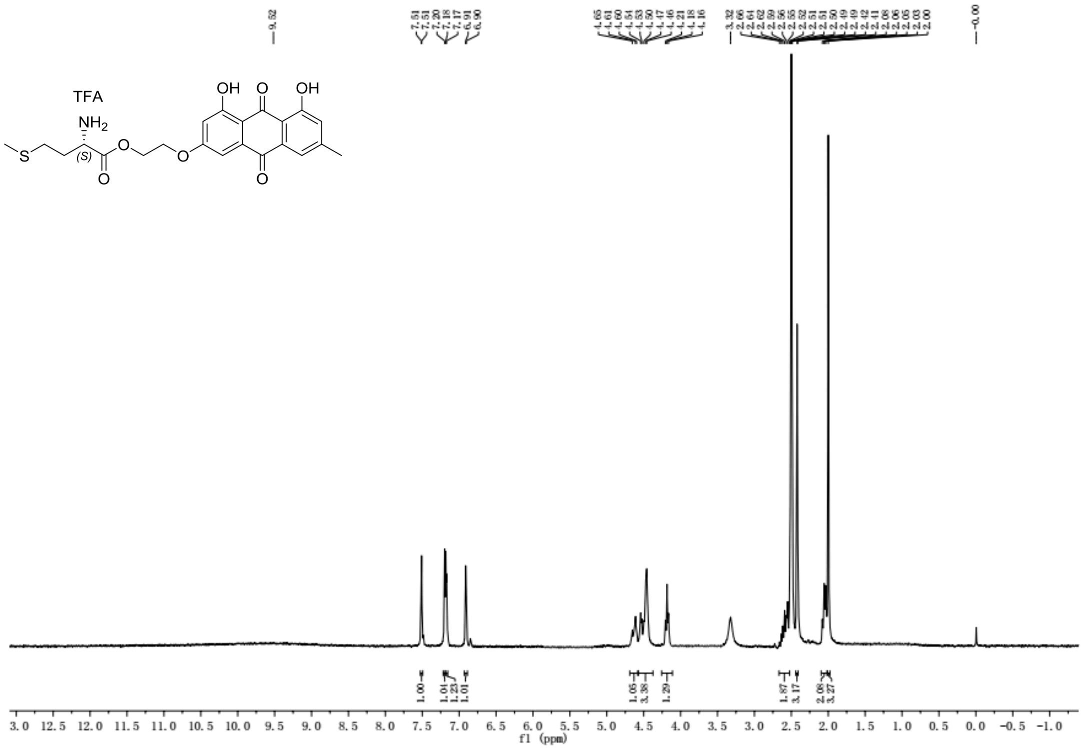


**Figure S27.** $^1$H NMR (400 MHz, DMSO–$d_6$) spectrum of compound **3i**.

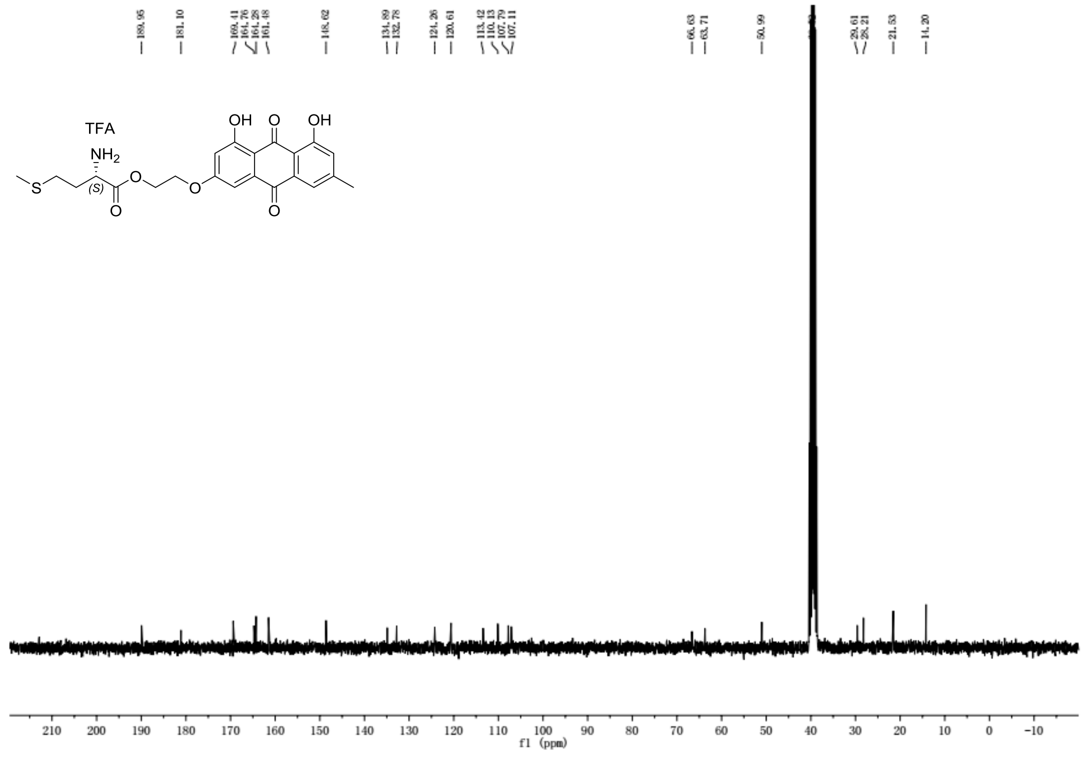


**Figure S28.** $^{13}$C NMR (101 MHz, DMSO–$d_6$) spectrum of compound **3i**

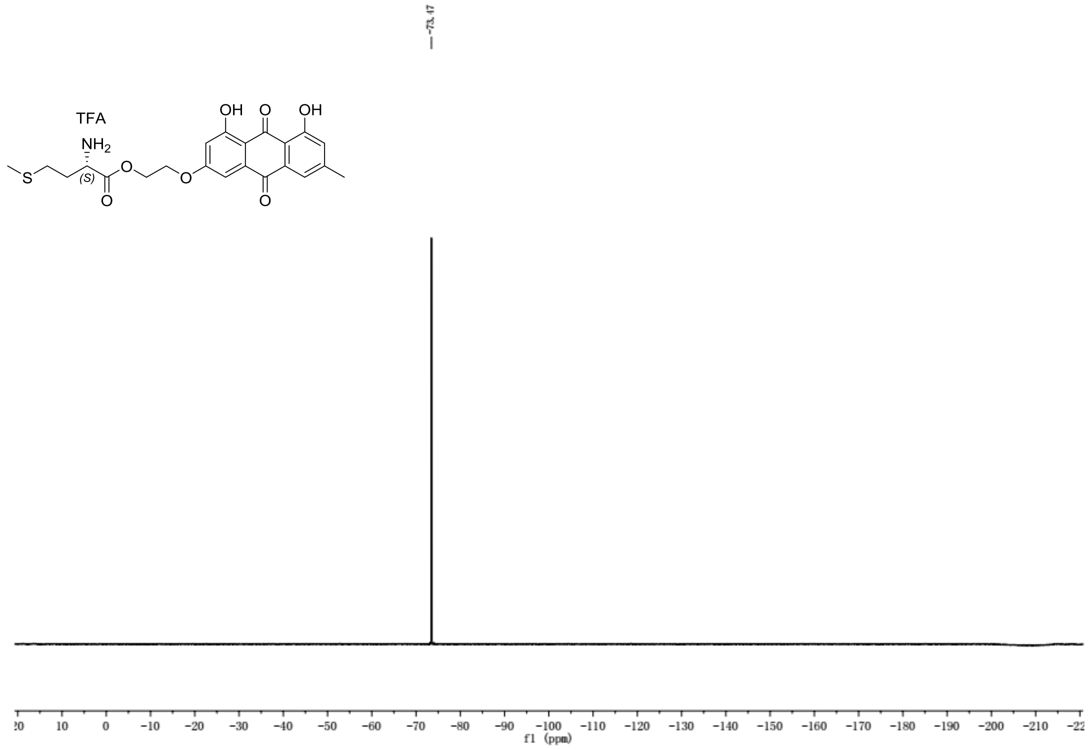


**Figure S29.** $^{19}$F NMR (376 MHz, DMSO–$d_6$) spectrum of compound **3i**.

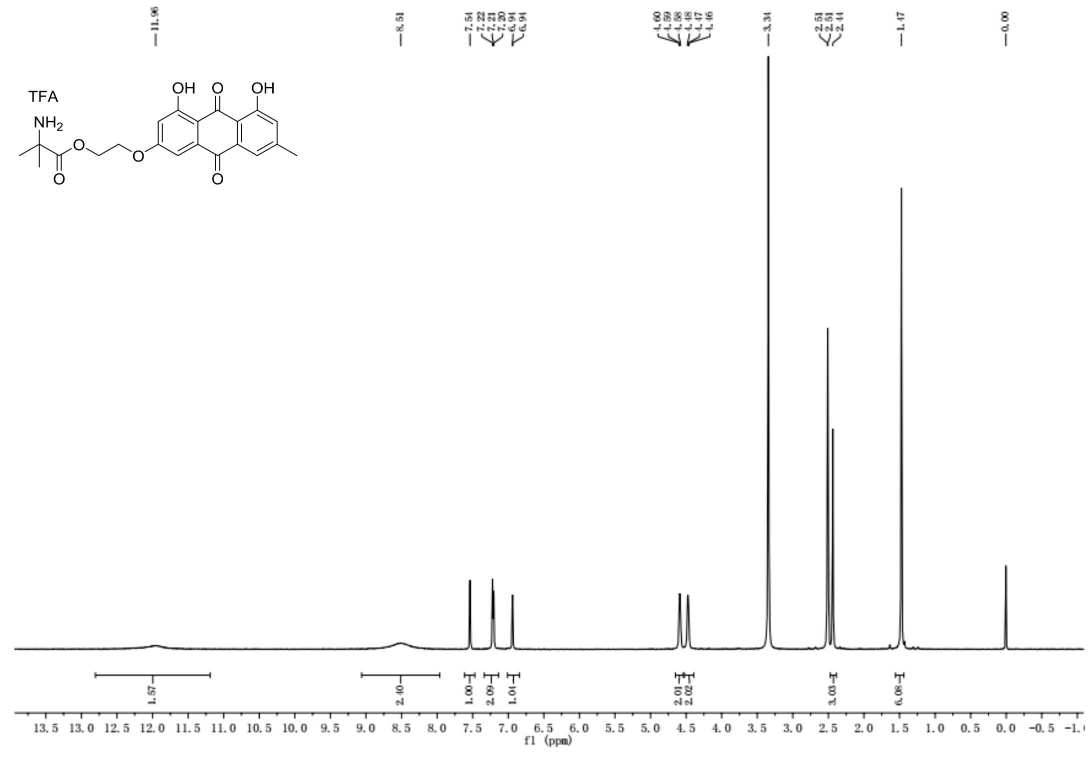


**Figure S30.** $^{1}$H NMR (400 MHz, DMSO–$d_6$) spectrum of compound **3j**.

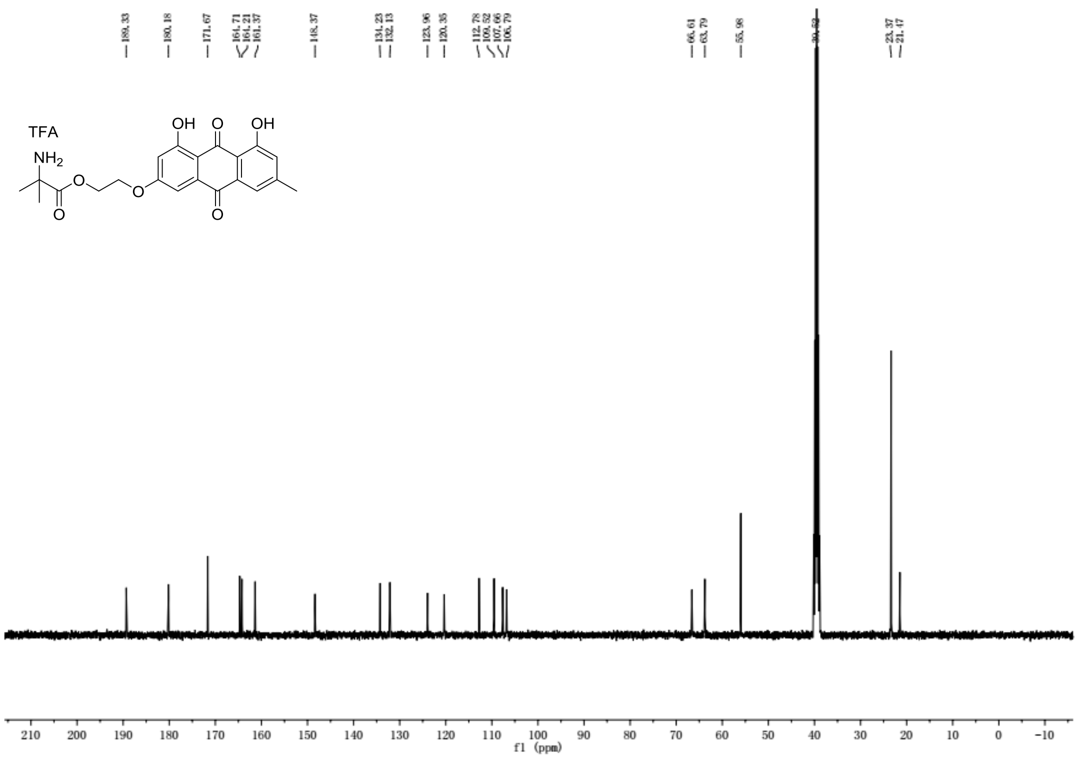


**Figure S31.** $^{13}$C NMR (101 MHz, DMSO–$d_6$) spectrum of compound **3j**.

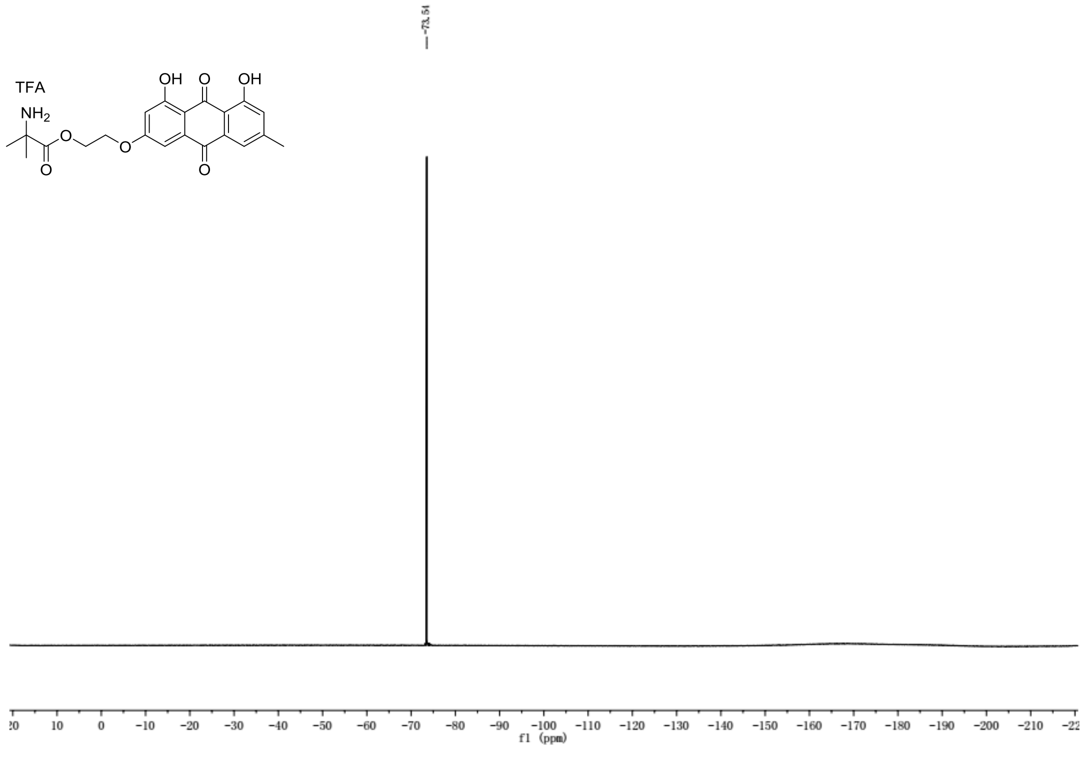


**Figure S32.** $^{19}$F NMR (376 MHz, DMSO–$d_6$) spectrum of compound **3j**.

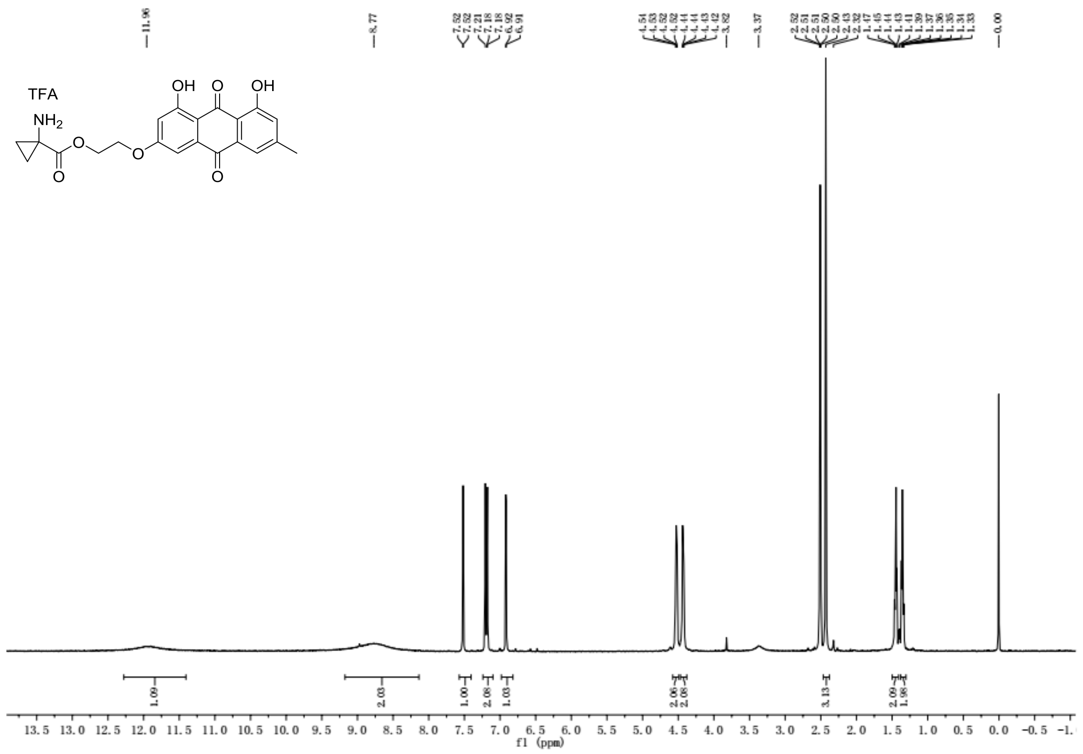


**Figure S33.** $^{1}$H NMR (400 MHz, DMSO–$d_6$) spectrum of compound **3k**.

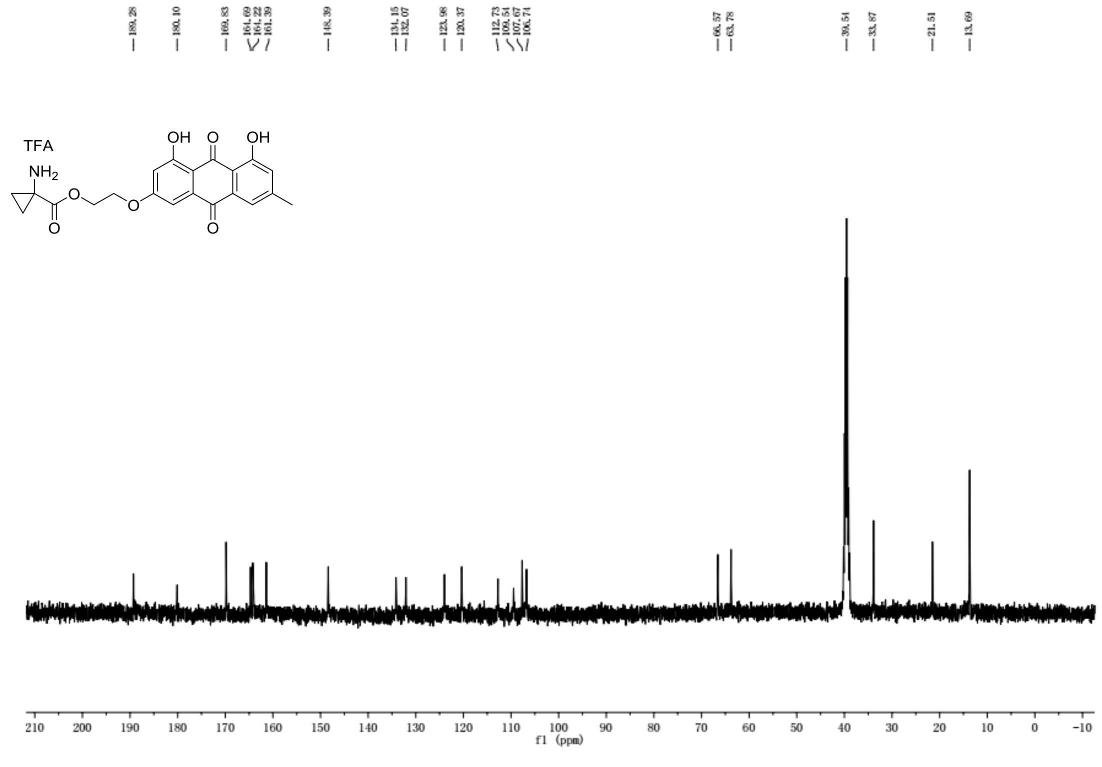


**Figure S34.** $^{13}$C NMR (101 MHz, DMSO–$d_6$) spectrum of compound **3k**.

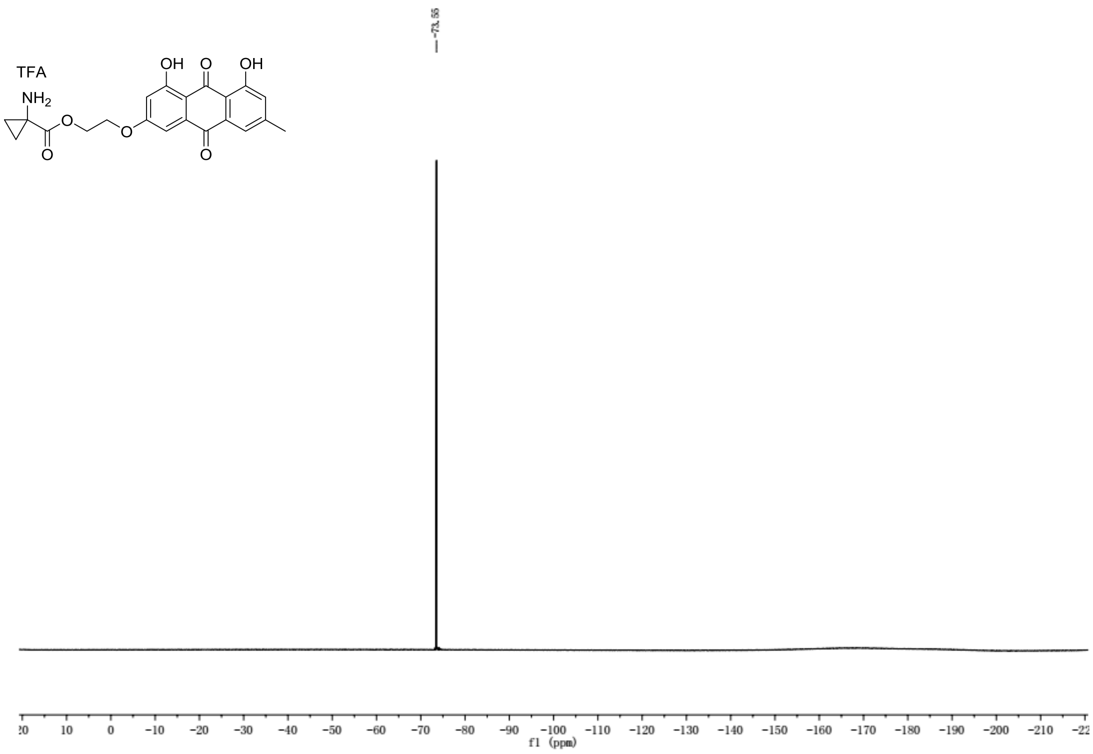

**Figure S35.** $^{19}$F NMR (376 MHz, DMSO–$d_6$) spectrum of compound **3k**.

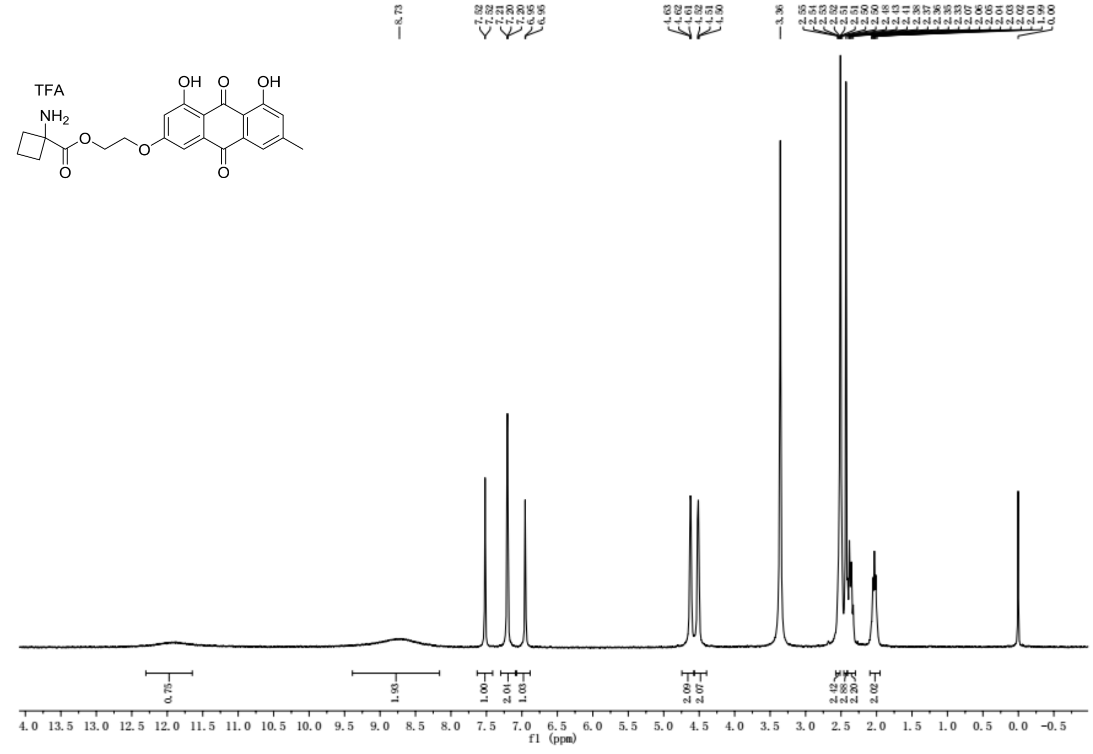

**Figure S36.** $^{1}$H NMR (400 MHz, DMSO–$d_6$) spectrum of compound **3l**.

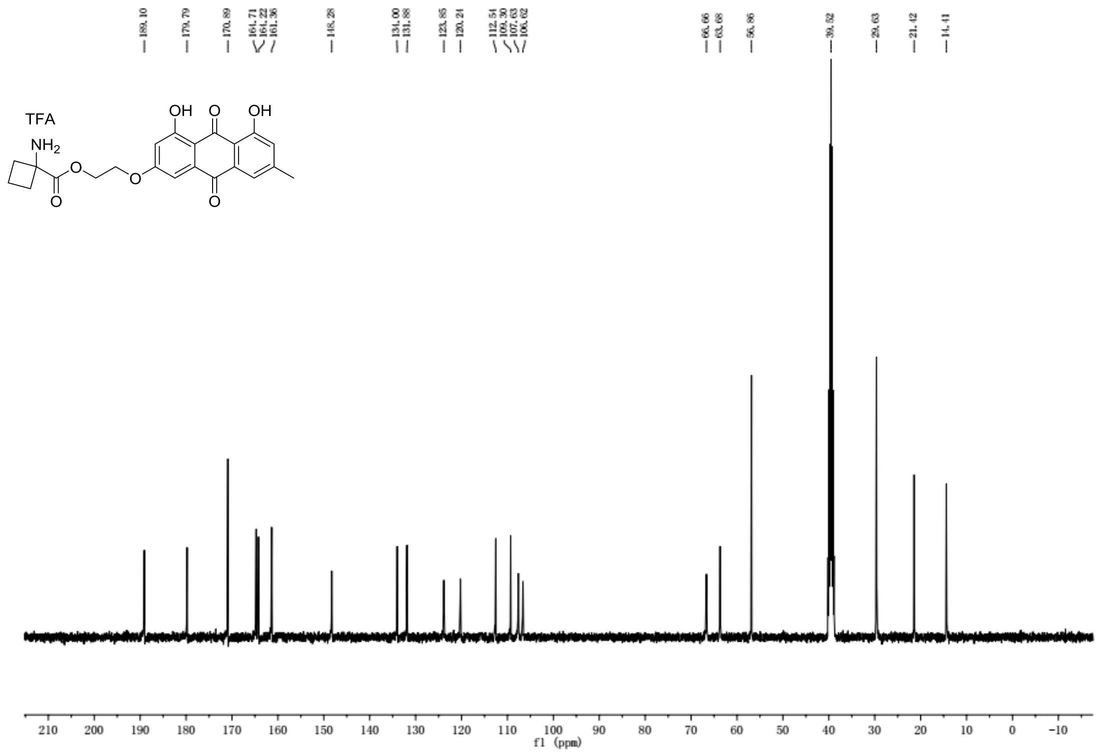

**Figure S37.** $^{13}C$ NMR (101 MHz, DMSO–$d_6$) spectrum of compound **3l**.

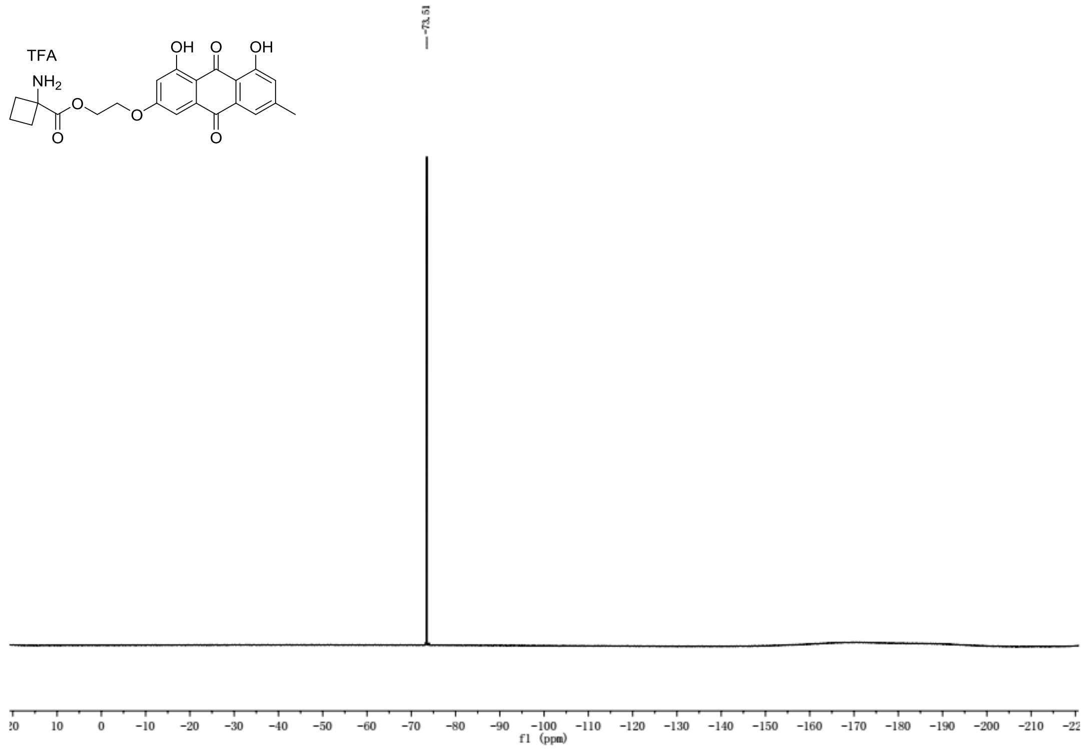

**Figure S38.** $^{19}F$ NMR (376 MHz, DMSO–$d_6$) spectrum of compound **3l**.

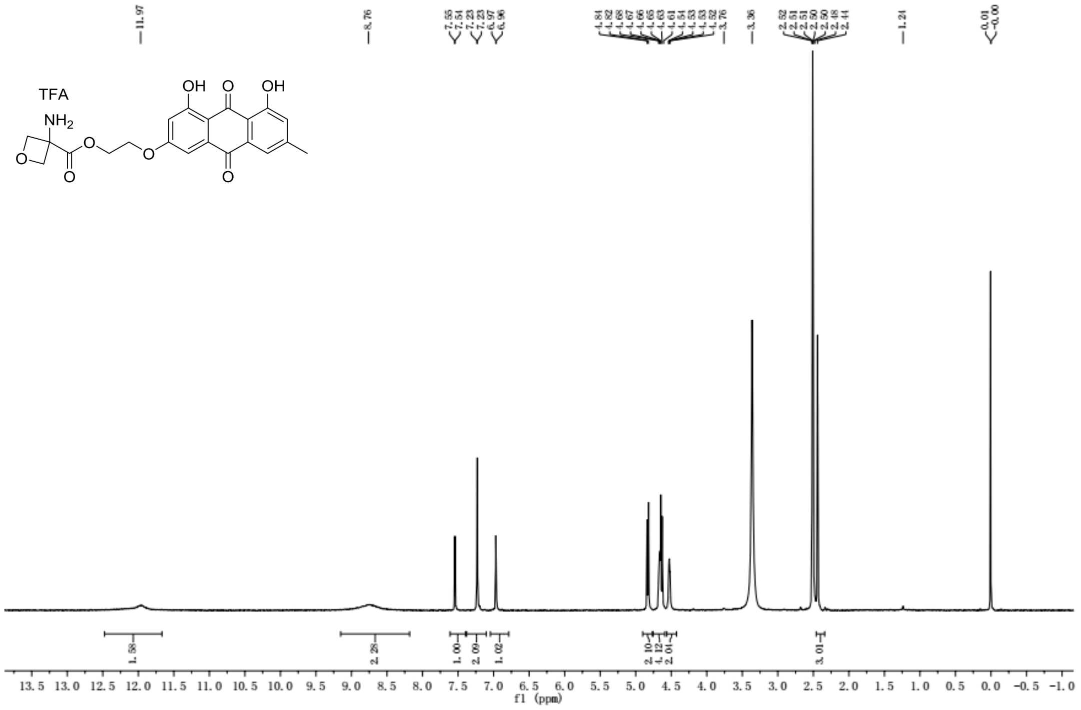

**Figure S39.** $^{1}$H NMR (400 MHz, DMSO–$d_6$) spectrum of compound **3m**.

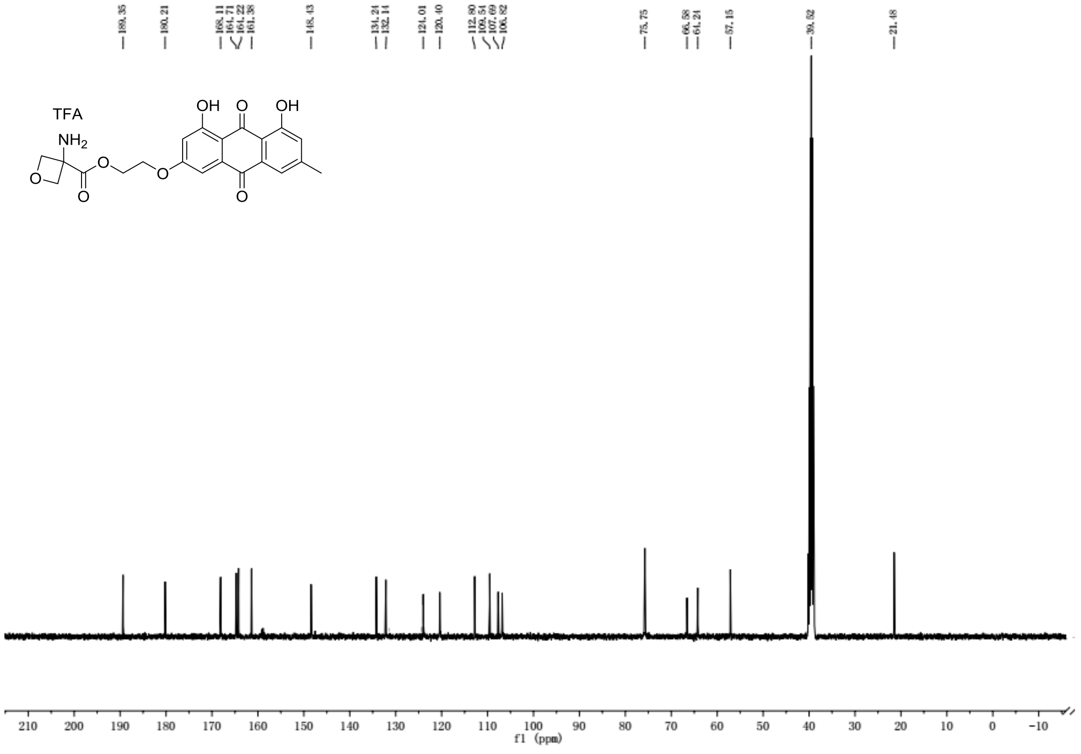

**Figure S40.** $^{13}$C NMR (101 MHz, DMSO–$d_6$) spectrum of compound **3m**.

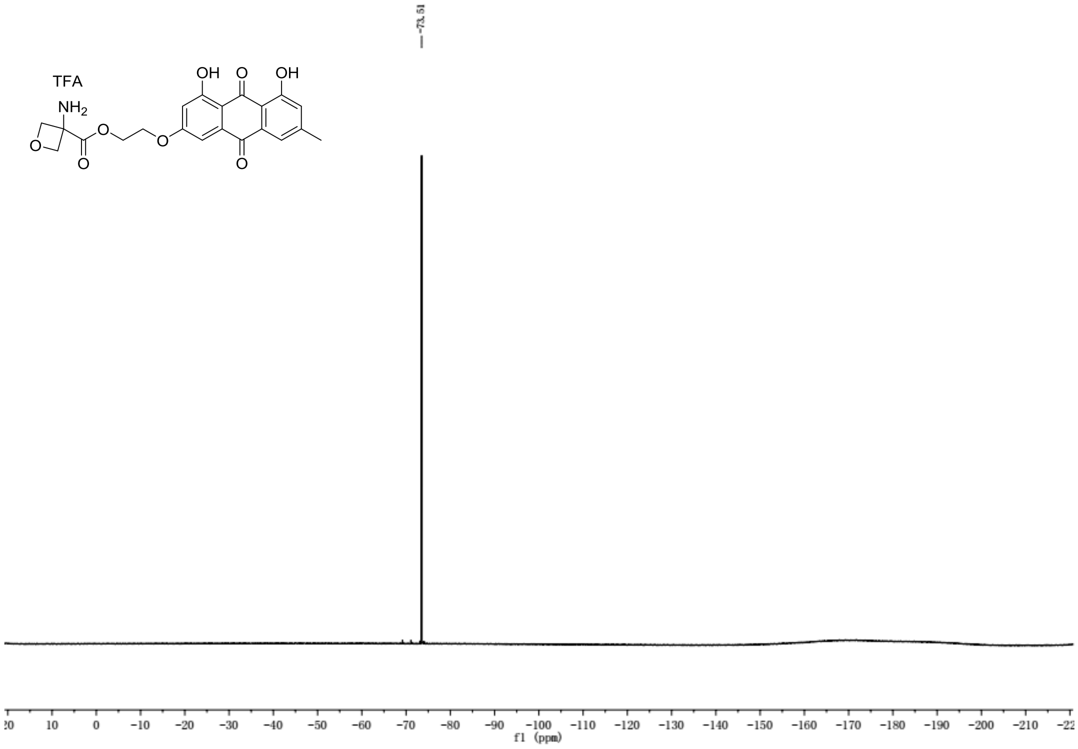

**Figure S41.** $^{19}$F NMR (376 MHz, DMSO–$d_6$) spectrum of compound **3m**.

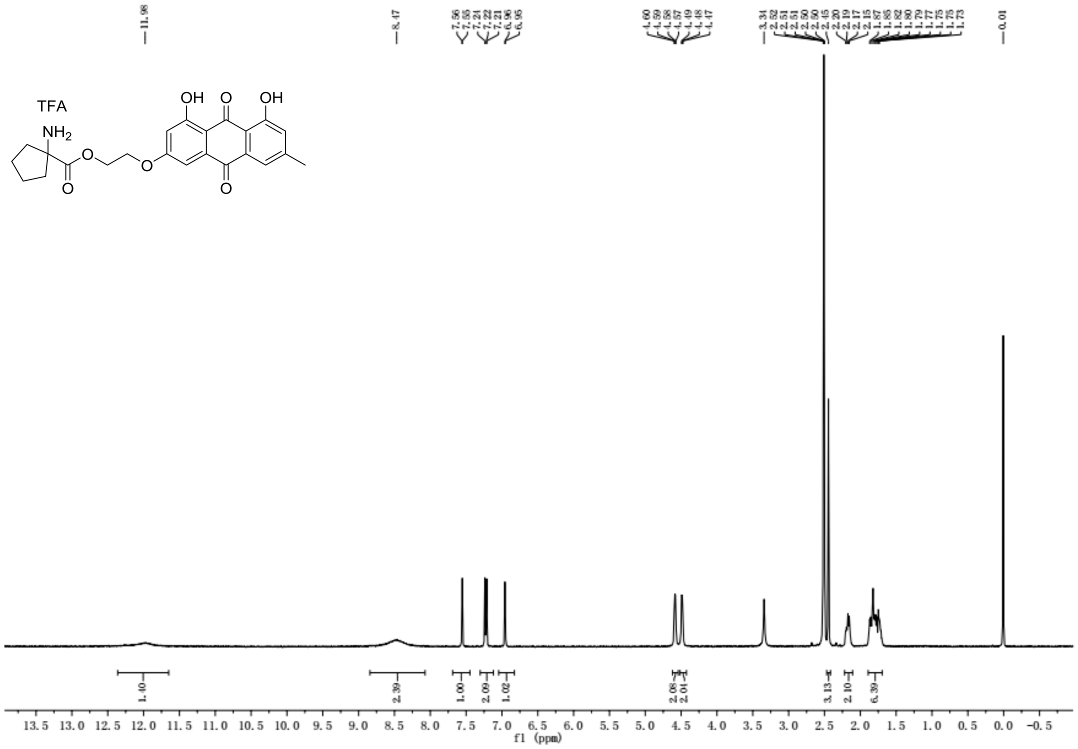

**Figure S42.** $^{1}$H NMR (400 MHz, DMSO–$d_6$) spectrum of compound **3n**.

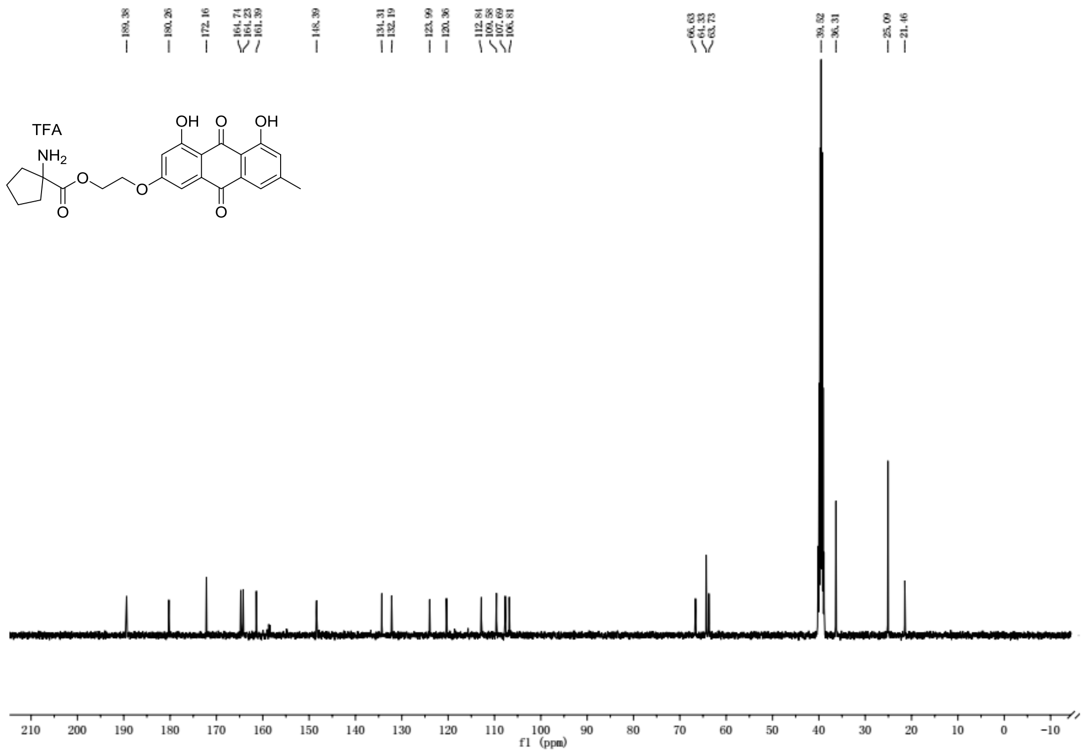

**Figure S43.** $^{13}$C NMR (101 MHz, DMSO–$d_6$) spectrum of compound **3n**.

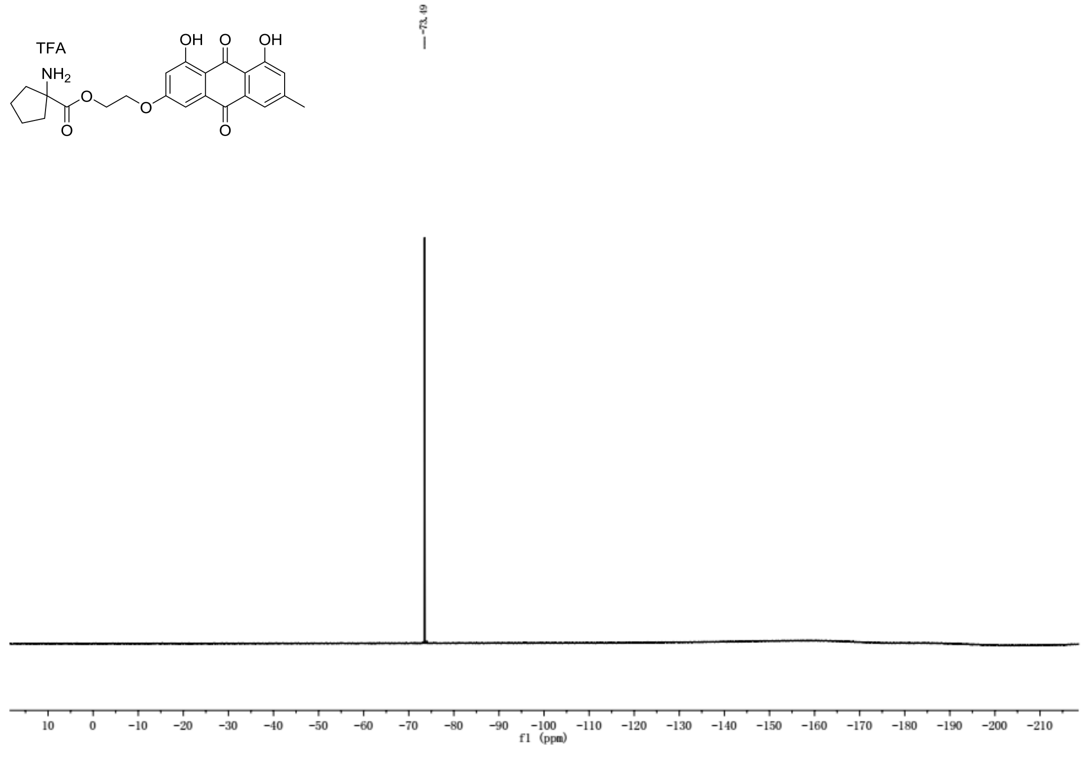

**Figure S44.** $^{19}$F NMR (376 MHz, DMSO–$d_6$) spectrum of compound **3n**.

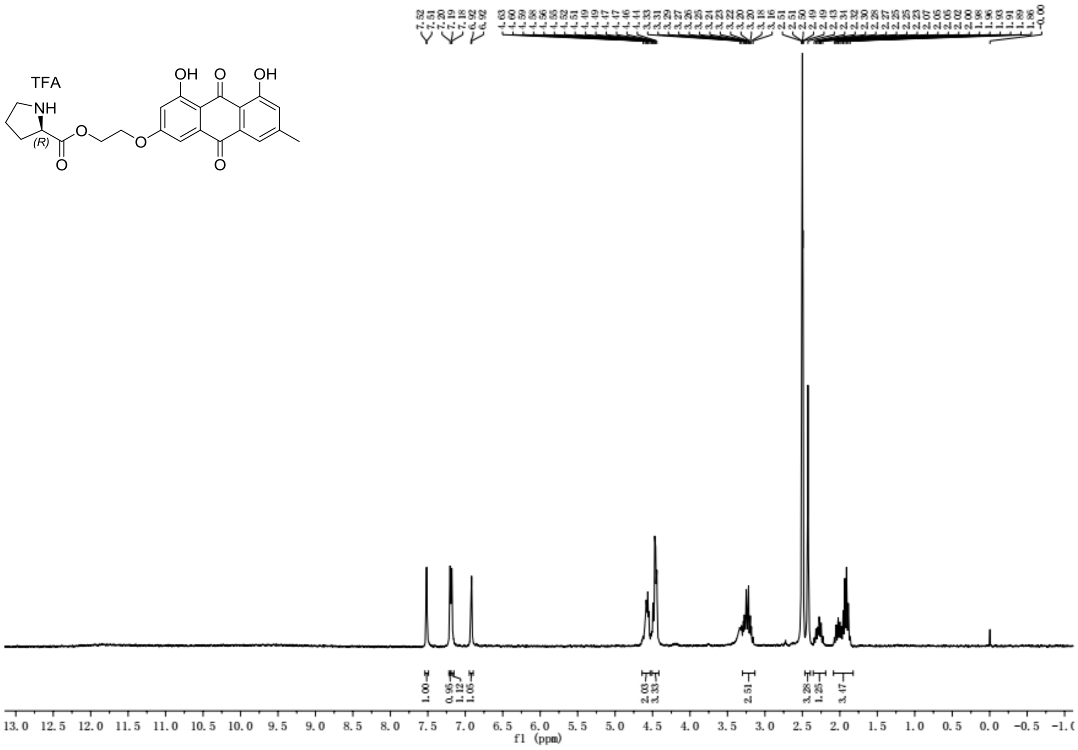

**Figure S45.** $^{1}$H NMR (400 MHz, DMSO–$d_6$) spectrum of compound **3o**.

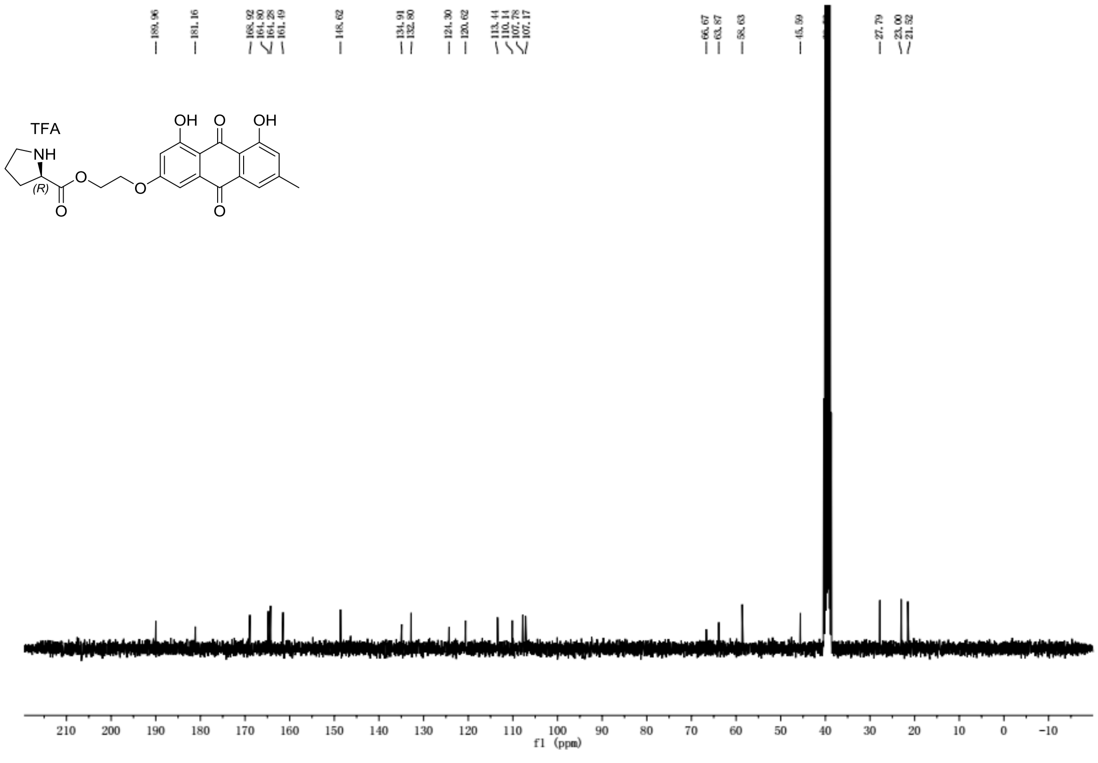

**Figure S46.** $^{13}$C NMR (101 MHz, DMSO–$d_6$) spectrum of compound **3o**.

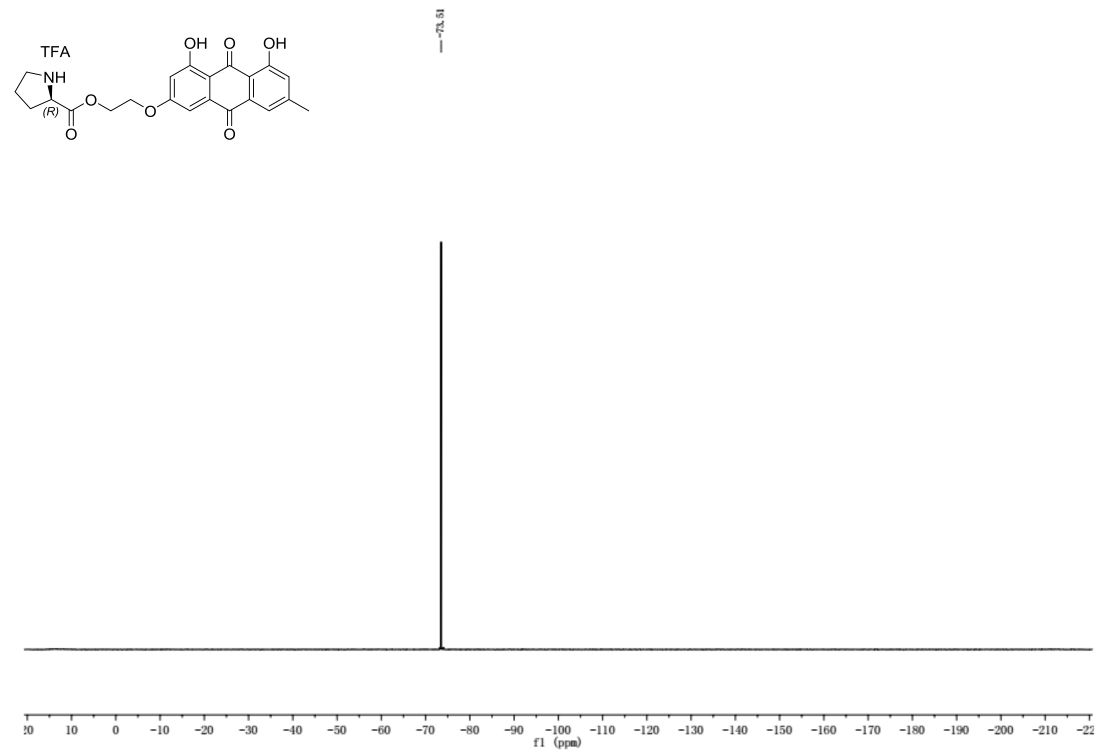

**Figure S47.** $^{19}F$ NMR (376 MHz, DMSO–$d_6$) spectrum of compound **3o**.

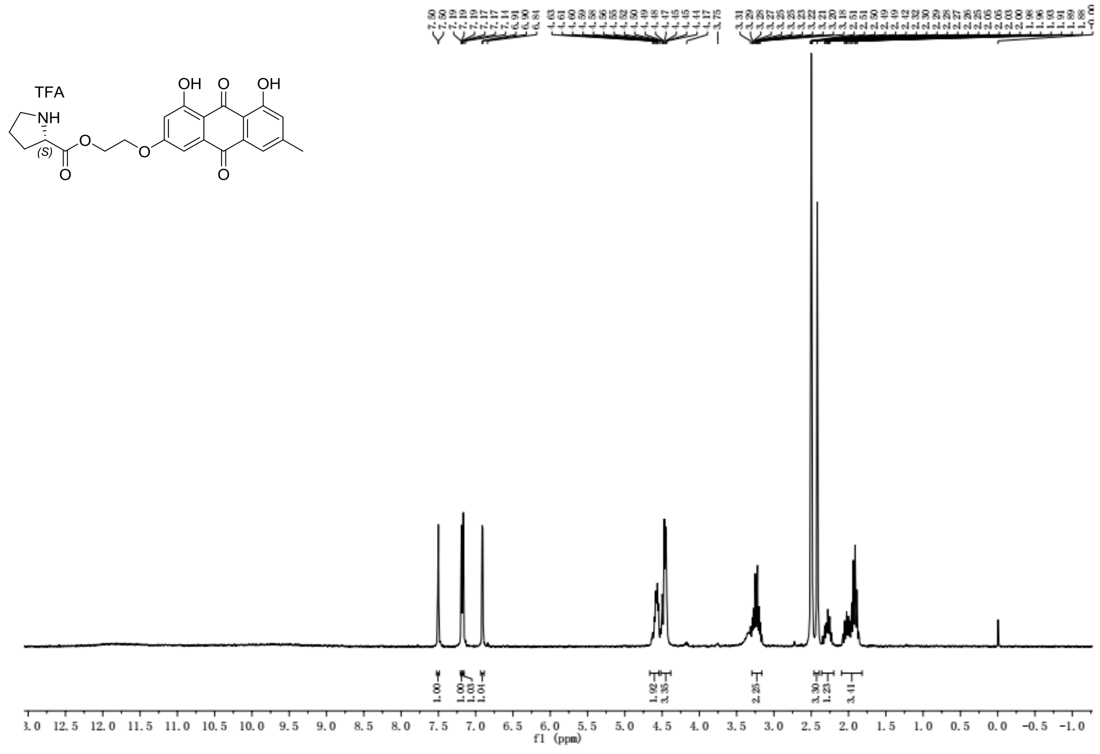

**Figure S48.** $^{1}H$ NMR (400 MHz, DMSO–$d_6$) spectrum of compound **3p**.

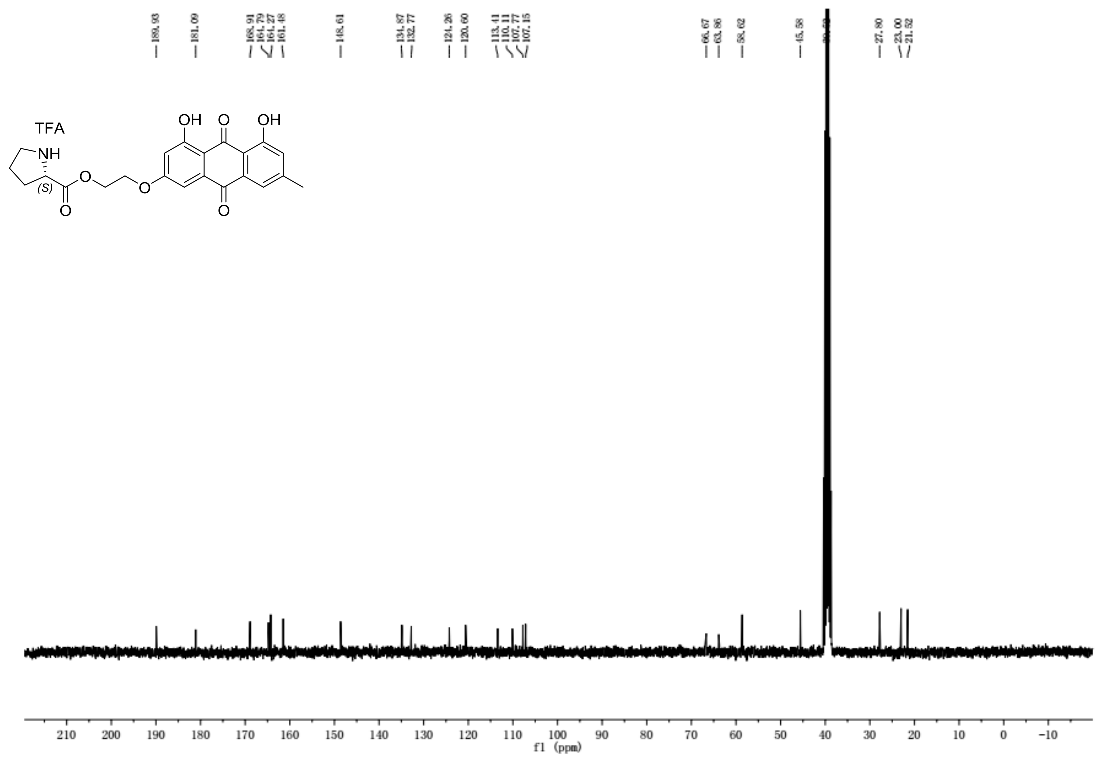


**Figure S49.** $^{13}$C NMR (101 MHz, DMSO–$d_6$) spectrum of compound **3p**.

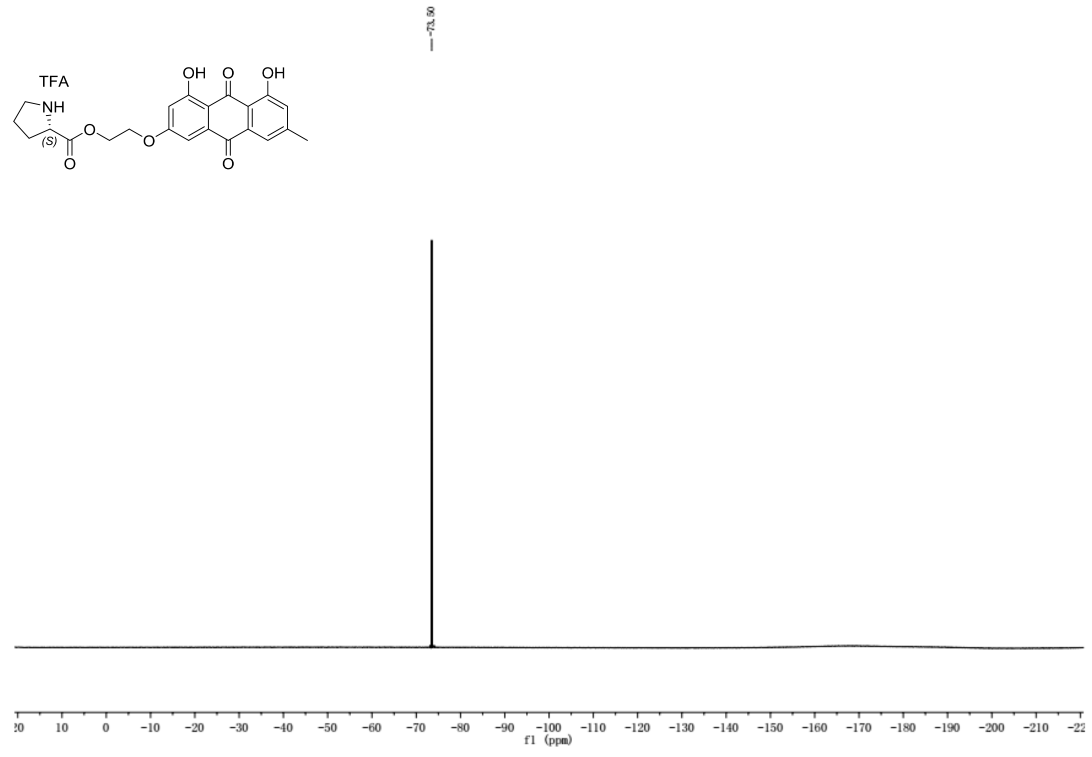


**Figure S50.** $^{19}$F NMR (376 MHz, DMSO–$d_6$) spectrum of compound **3p**.

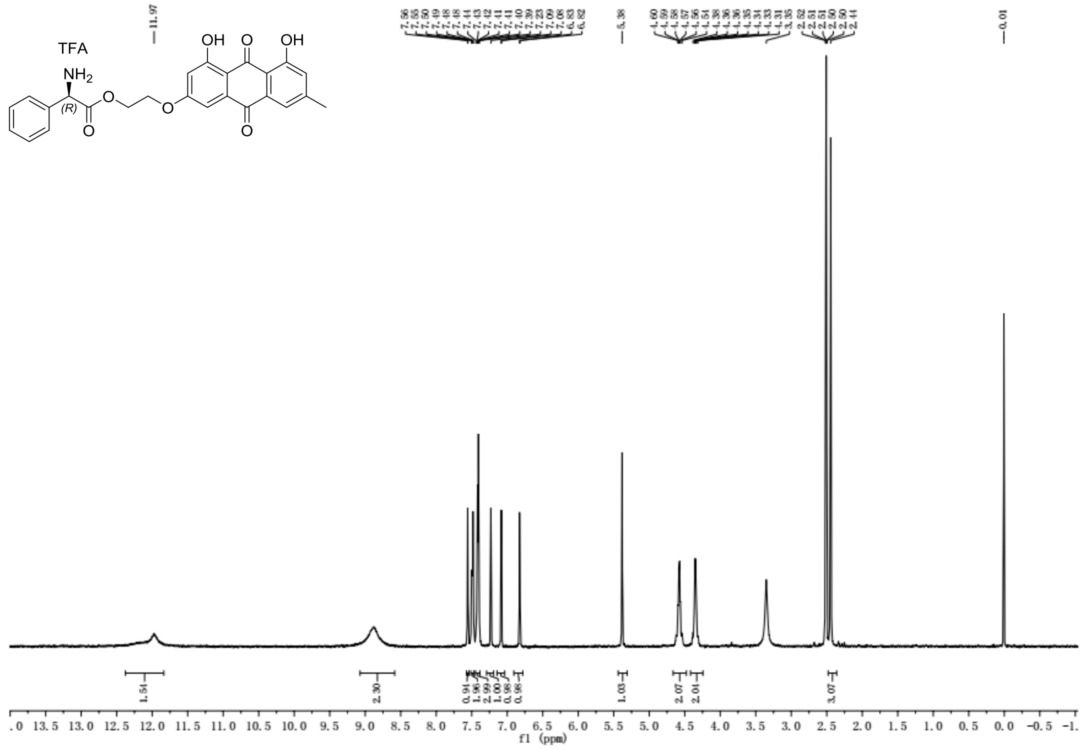

**Figure S51.** $^{1}$H NMR (400 MHz, DMSO–$d_6$) spectrum of compound **3q**.

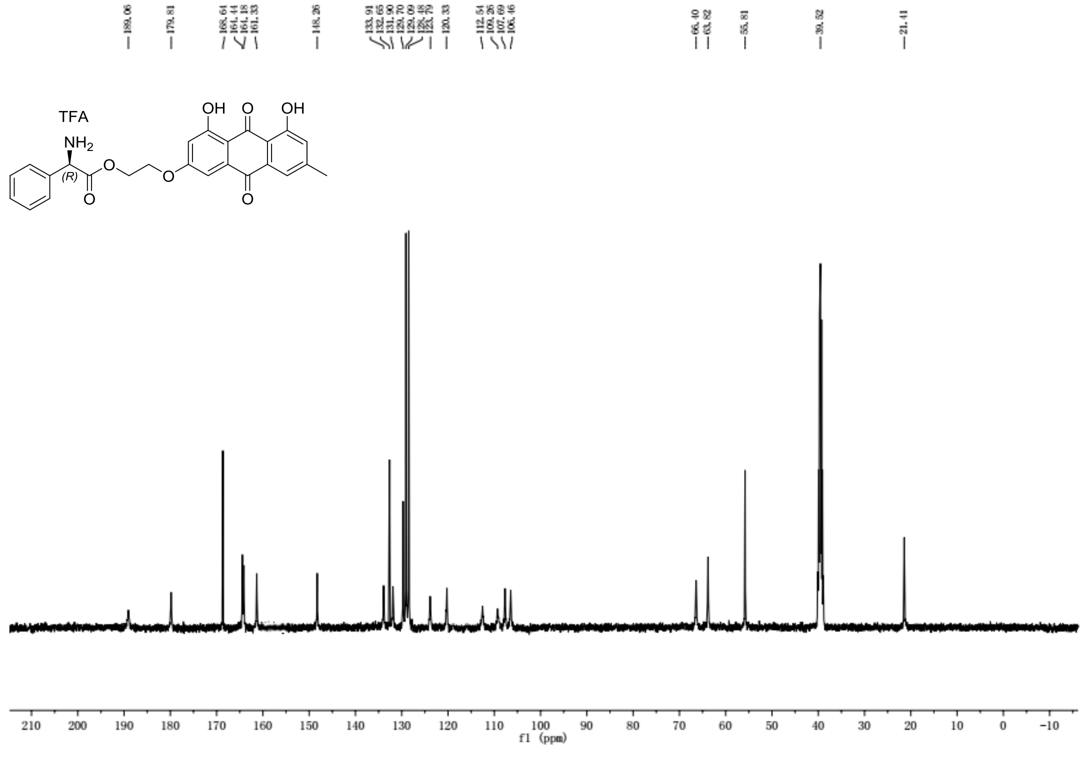

**Figure S52.** $^{13}$C NMR (101 MHz, DMSO–$d_6$) spectrum of compound **3q**.

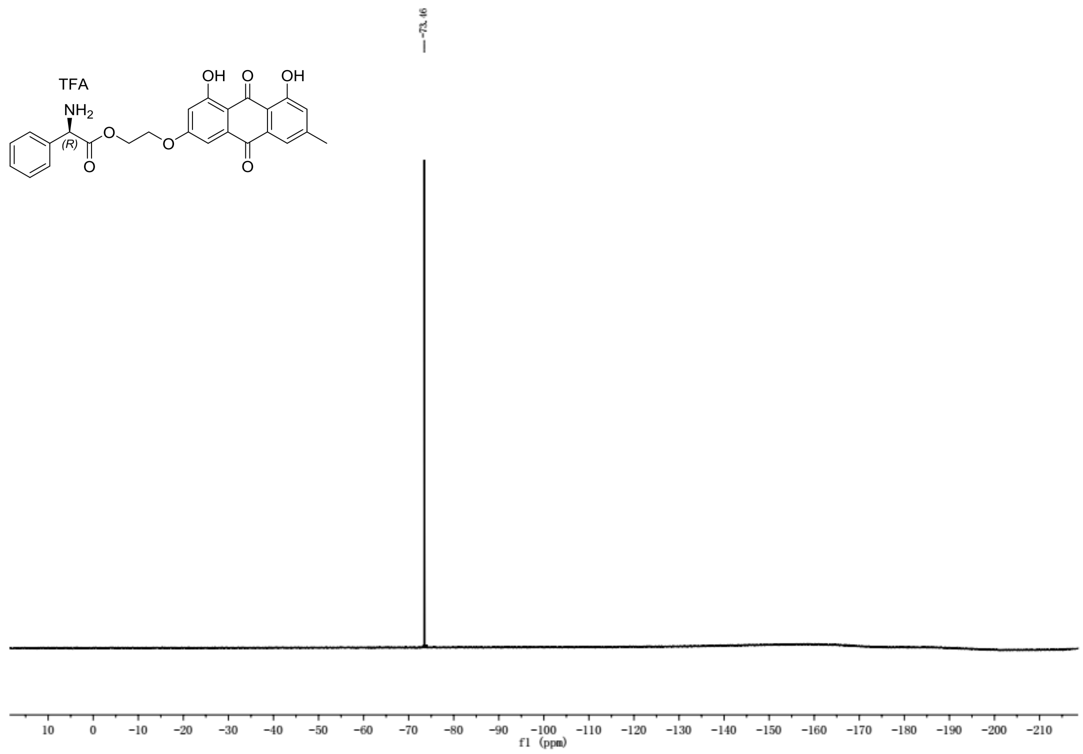

**Figure S53.** $^{19}$F NMR (376 MHz, DMSO–$d_6$) spectrum of compound **3q**.

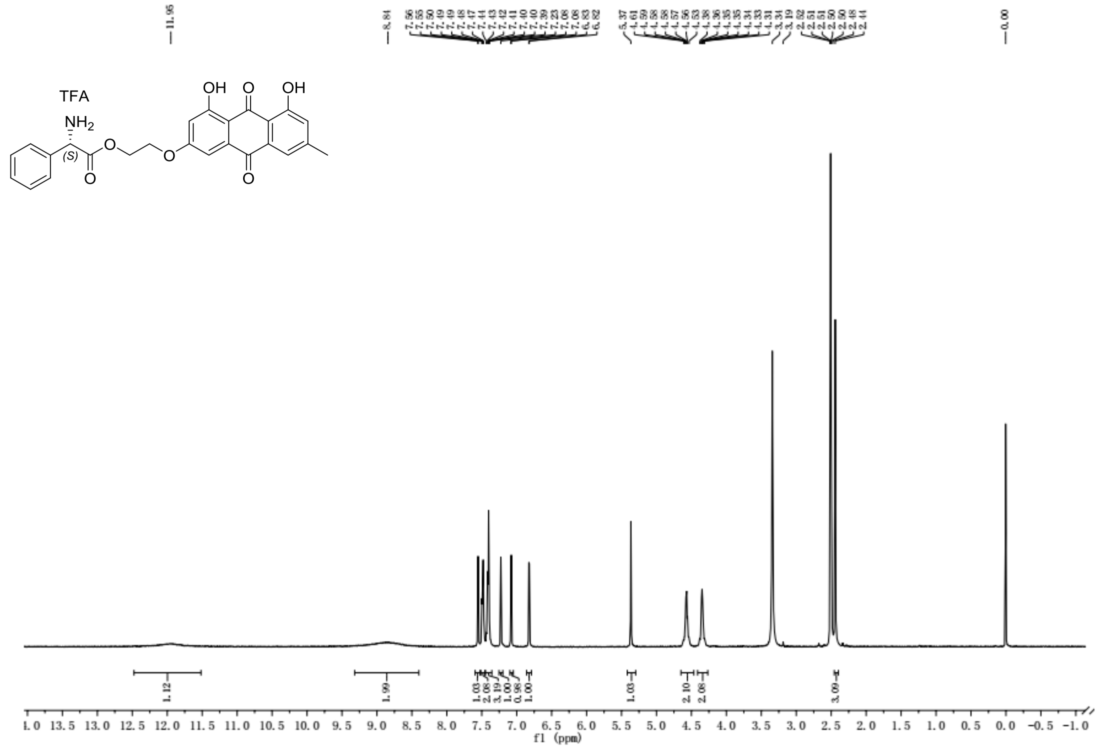

**Figure S54.** $^{1}$H NMR (400 MHz, DMSO–$d_6$) spectrum of compound **3r**.

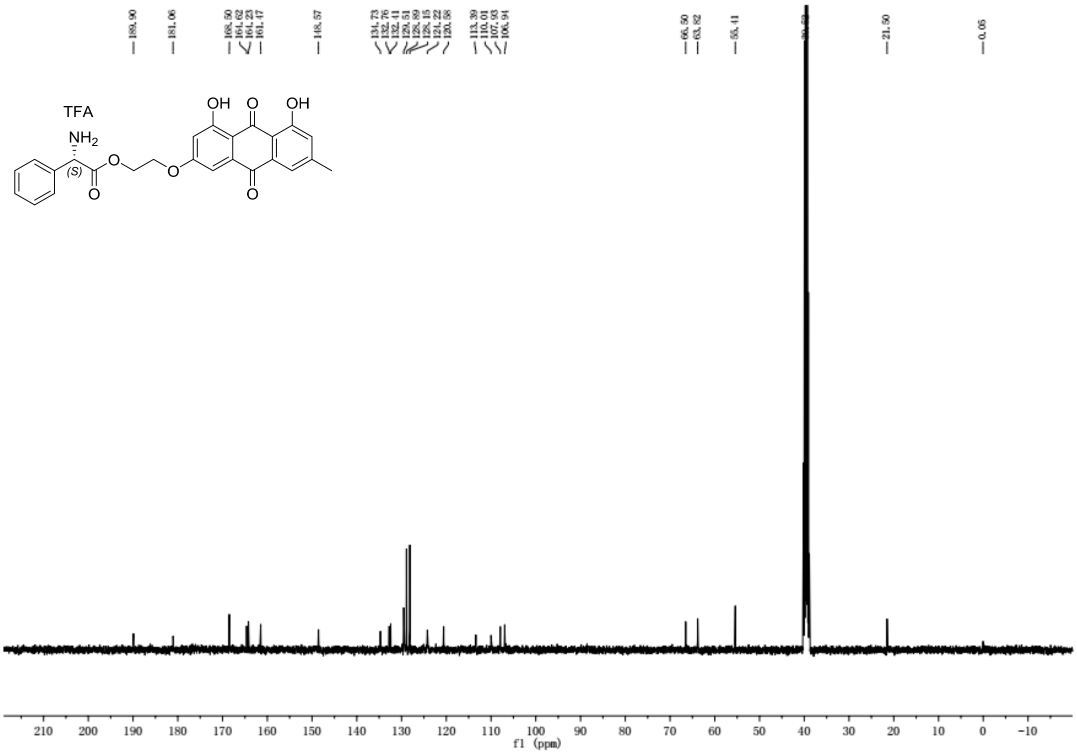

**Figure S55.** $^{13}$C NMR (101 MHz, DMSO–$d_6$) spectrum of compound **3r**.

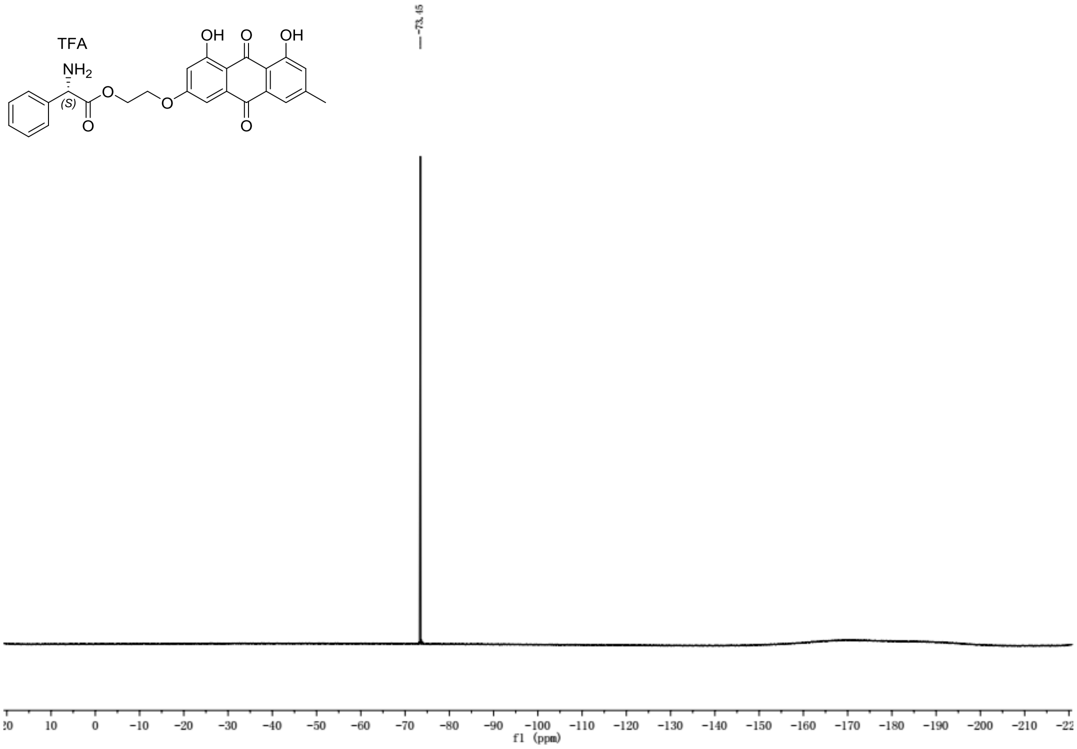

**Figure S56.** $^{19}$F NMR (376 MHz, DMSO–$d_6$) spectrum of compound **3r**.

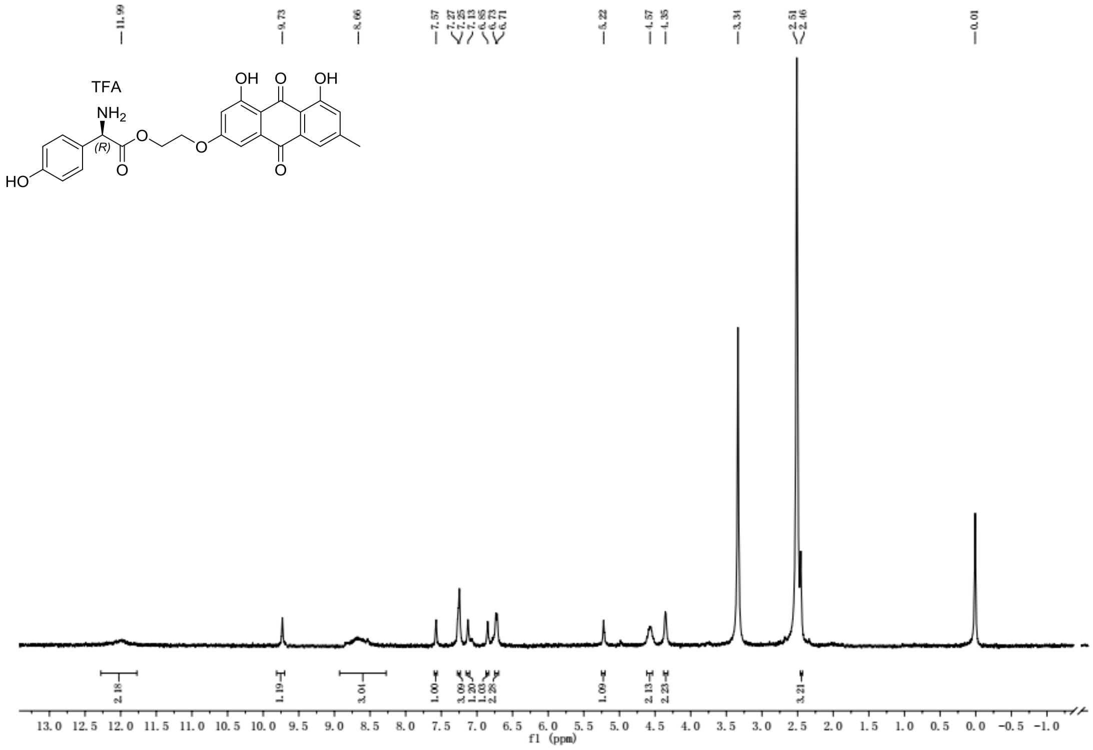


**Figure S57.** $^{1}$H NMR (400 MHz, DMSO–$d_6$) spectrum of compound **3s**.

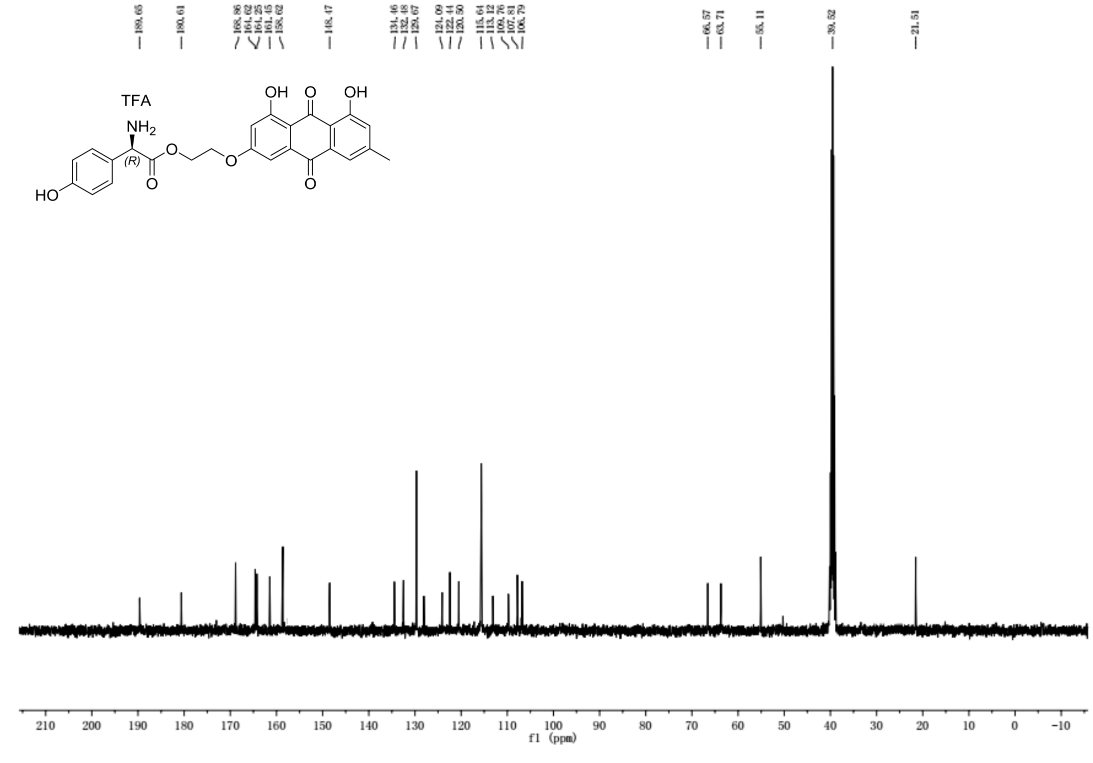


**Figure S58.** $^{13}$C NMR (101 MHz, DMSO–$d_6$) spectrum of compound **3s**.

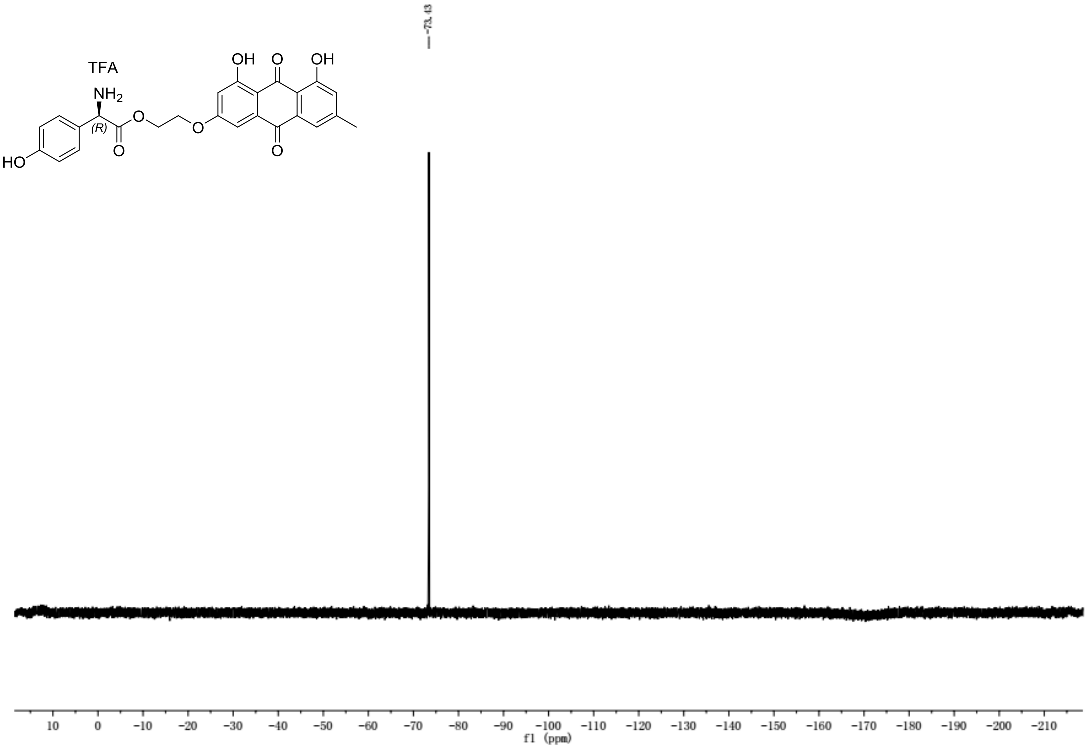


**Figure S59.** $^{19}$F NMR (376 MHz, DMSO–$d_6$) spectrum of compound **3s**.

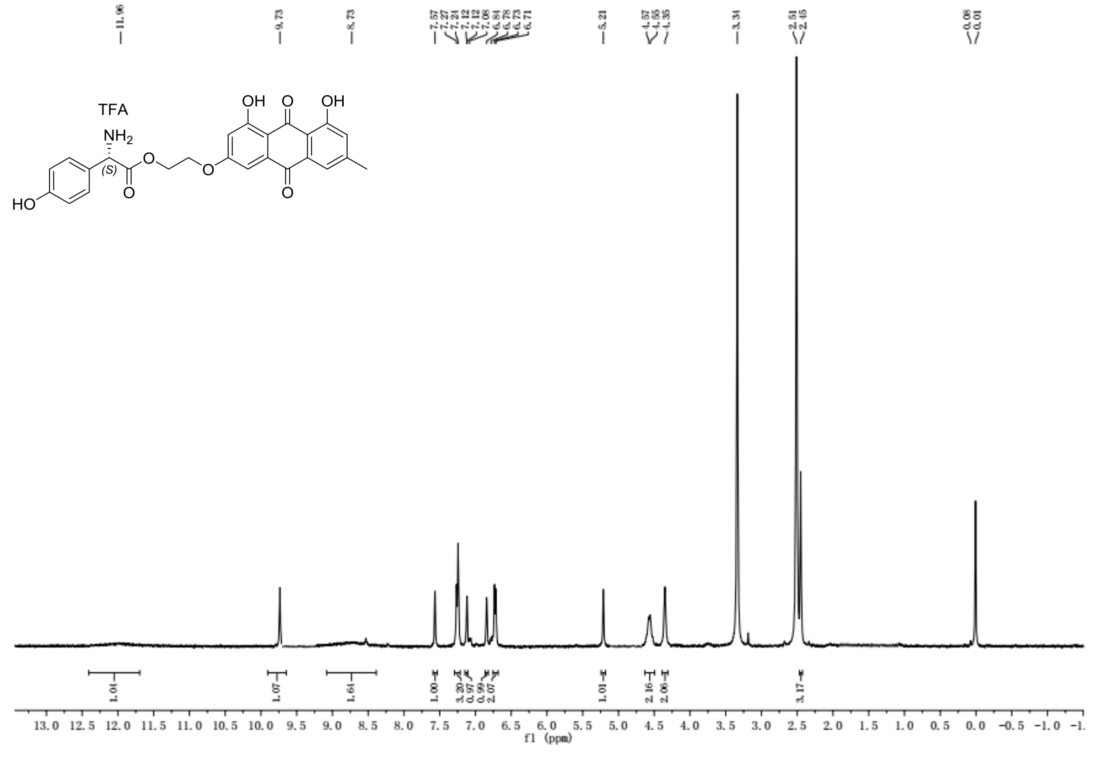


**Figure S60.** $^{1}$H NMR (400 MHz, DMSO–$d_6$) spectrum of compound **3t**.

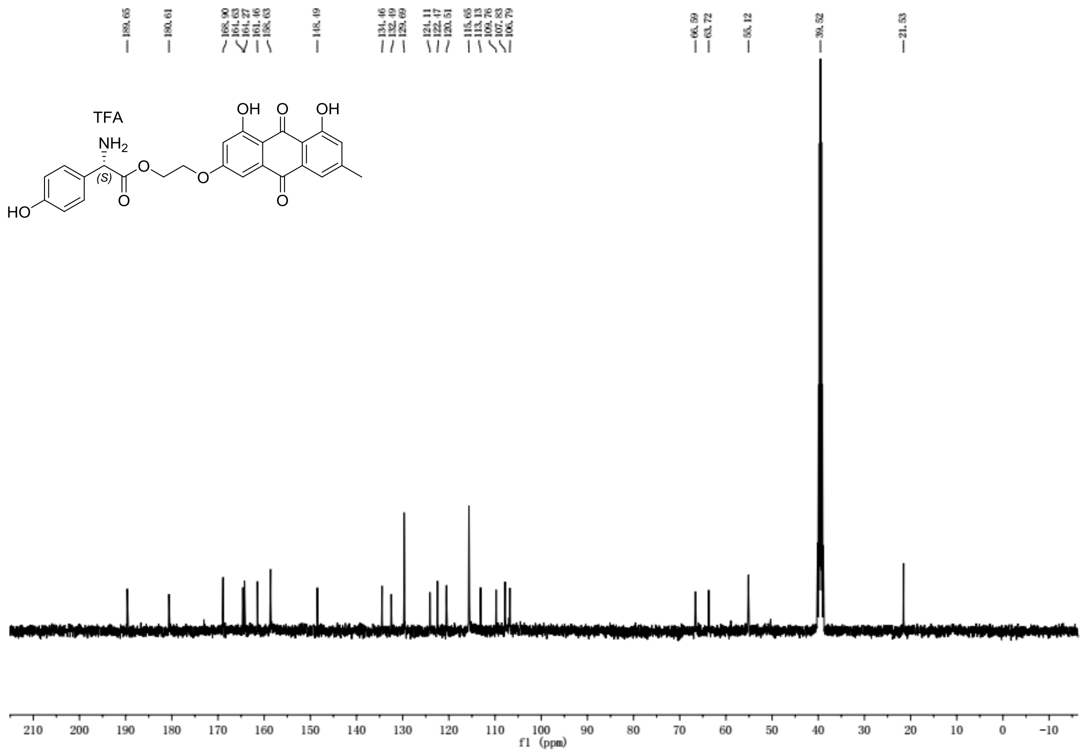

**Figure S61.** $^{13}$C NMR (101 MHz, DMSO–$d_6$) spectrum of compound **3t**.

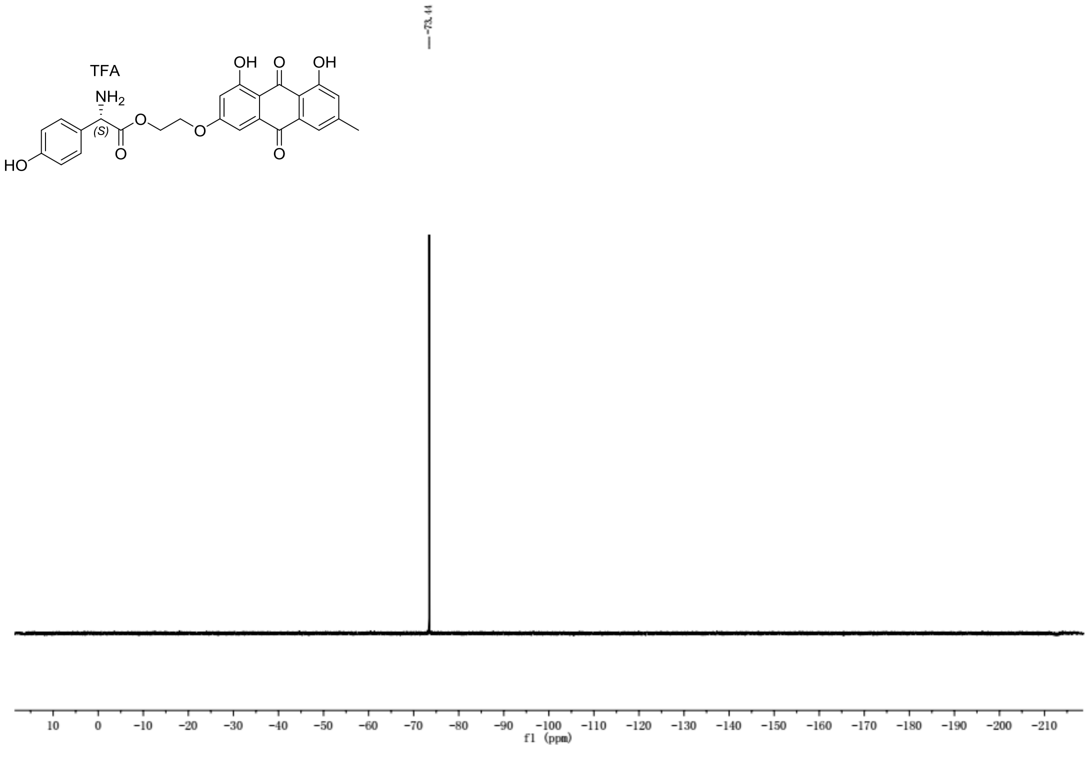

**Figure S62.** $^{19}$F NMR (376 MHz, DMSO–$d_6$) spectrum of compound **3t**.

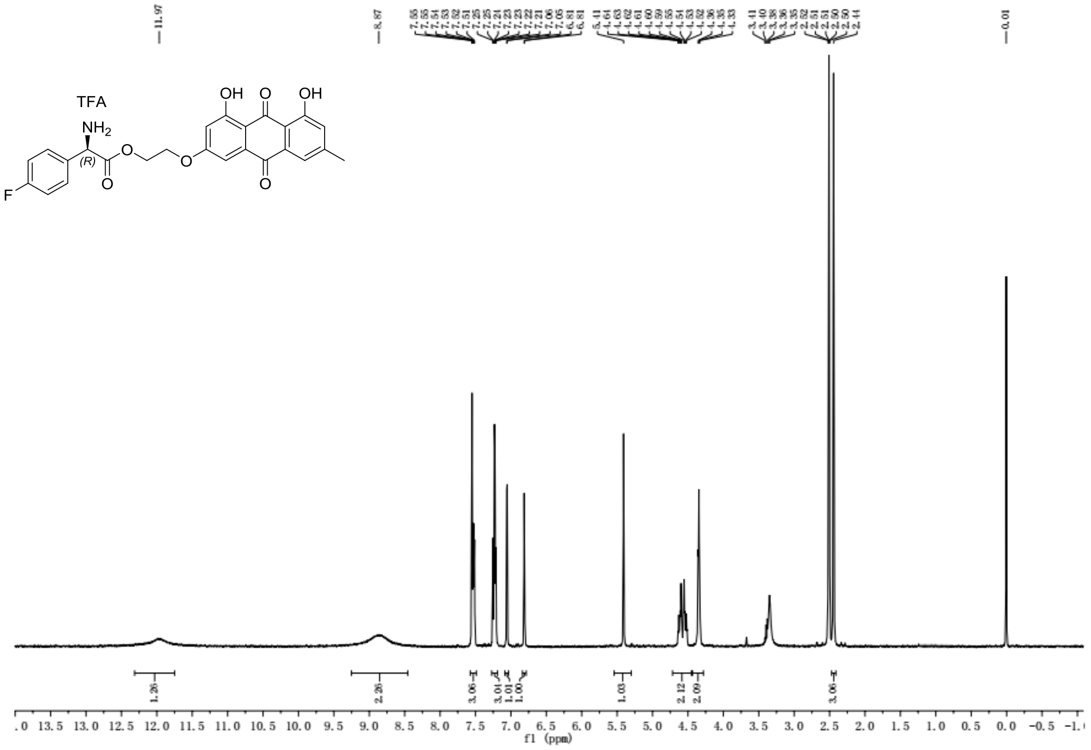


**Figure S63.** $^{1}$H NMR (400 MHz, DMSO–$d_6$) spectrum of compound **3u**.

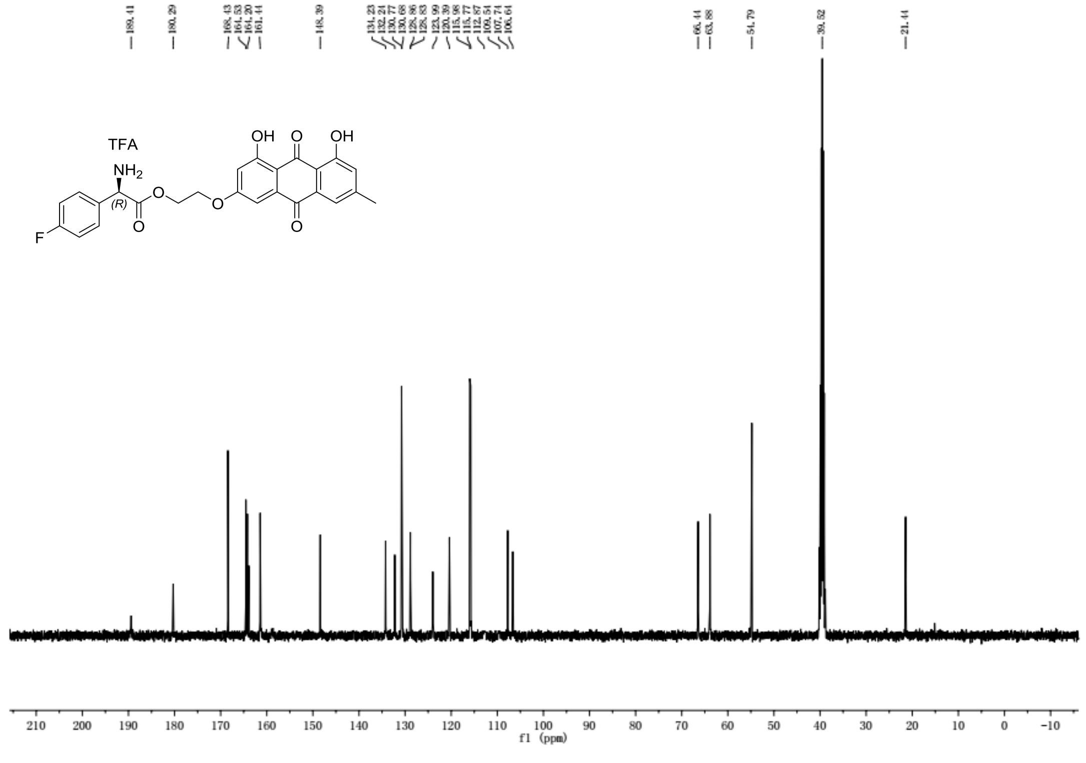


**Figure S64.** $^{13}$C NMR (101 MHz, DMSO–$d_6$) spectrum of compound **3u**.

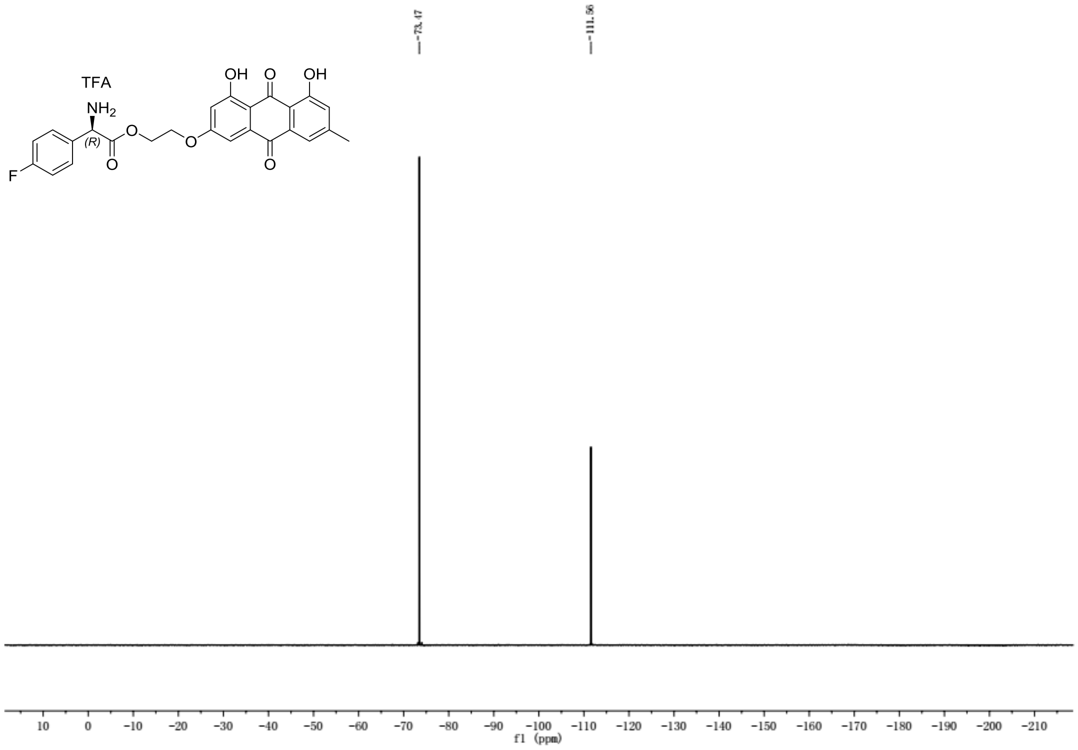

**Figure S65.** $^{19}$F NMR (376 MHz, DMSO–$d_6$) spectrum of compound **3u**.

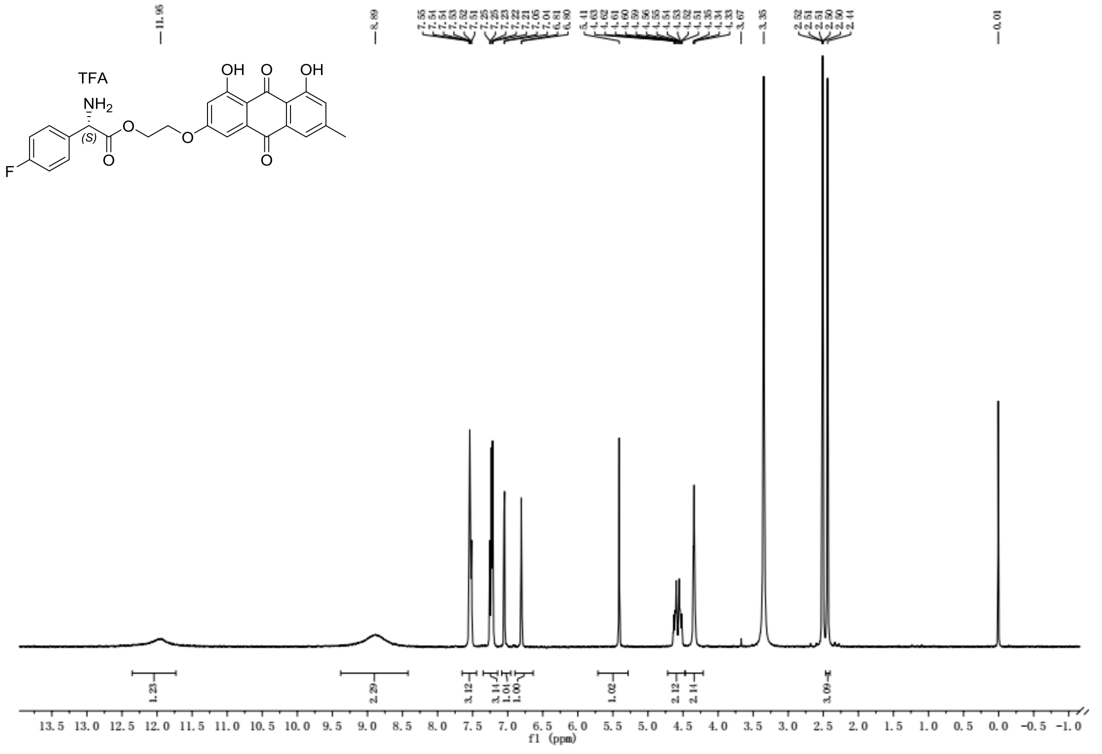

**Figure S66.** $^{1}$H NMR (400 MHz, DMSO–$d_6$) spectrum of compound **3v**.

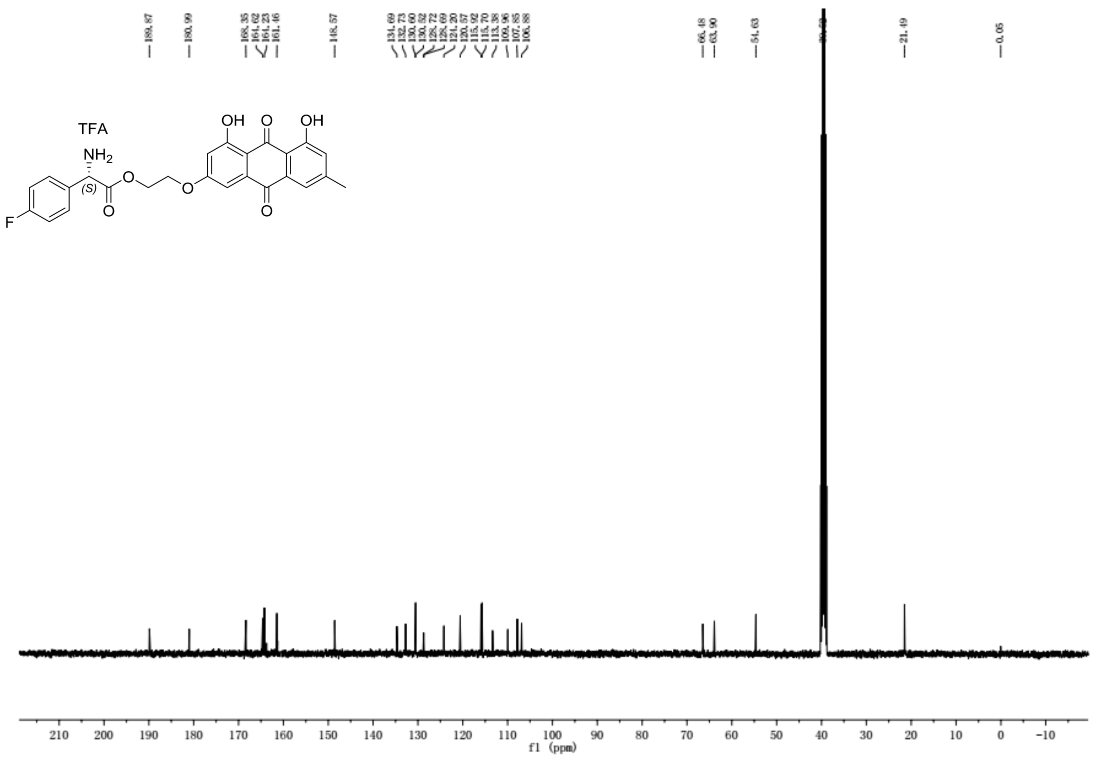

**Figure S67.** $^{13}$C NMR (101 MHz, DMSO–$d_6$) spectrum of compound **3v**.

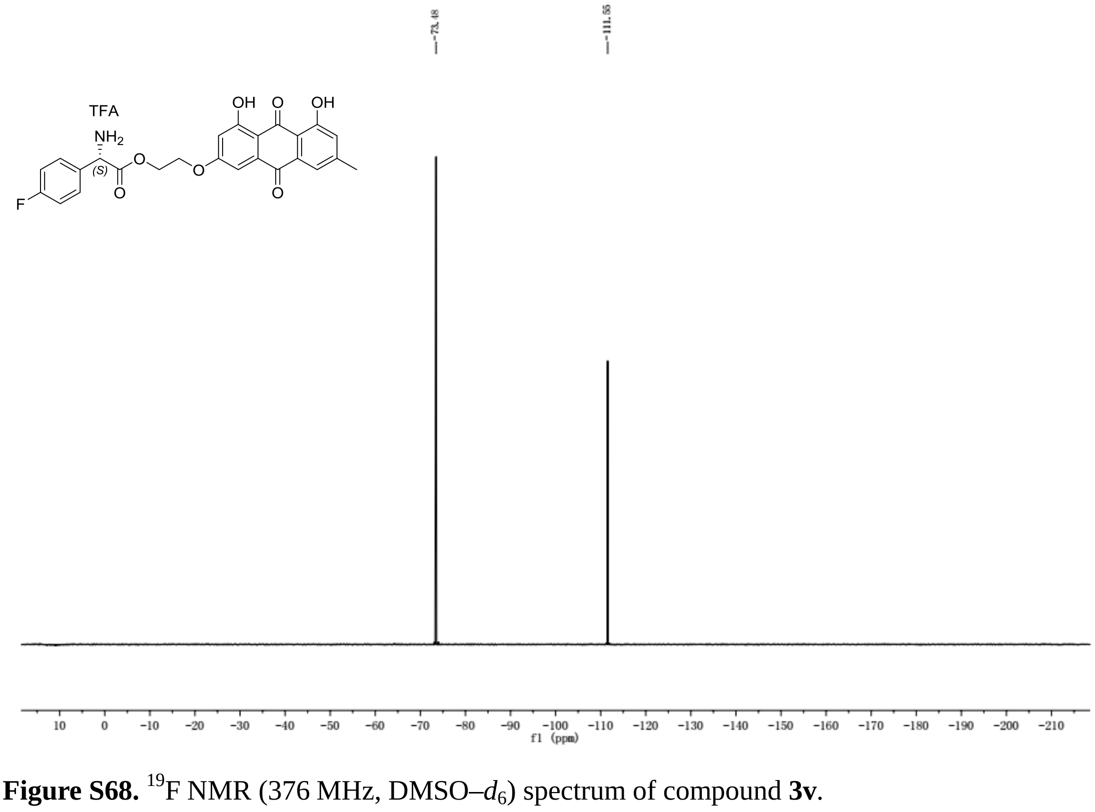

**Figure S68.** $^{19}$F NMR (376 MHz, DMSO–$d_6$) spectrum of compound **3v**.

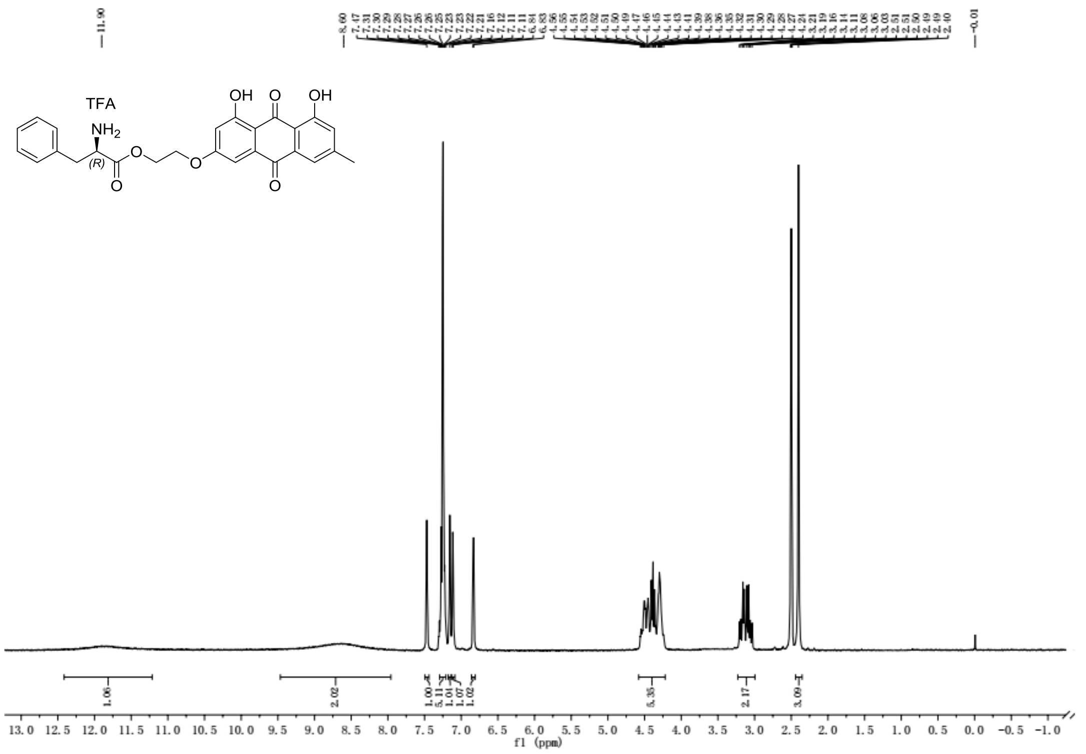

**Figure S69.** $^{1}$H NMR (400 MHz, DMSO–$d_6$) spectrum of compound **3w**.

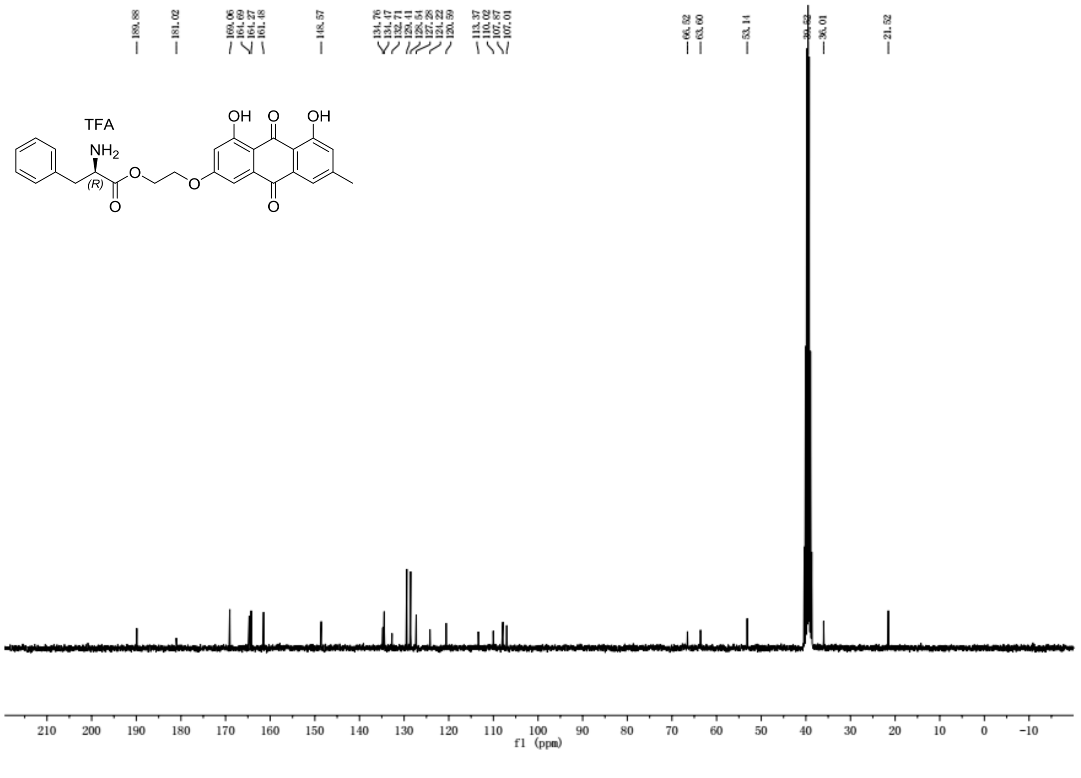

**Figure S70.** $^{13}$C NMR (101 MHz, DMSO–$d_6$) spectrum of compound **3w**.

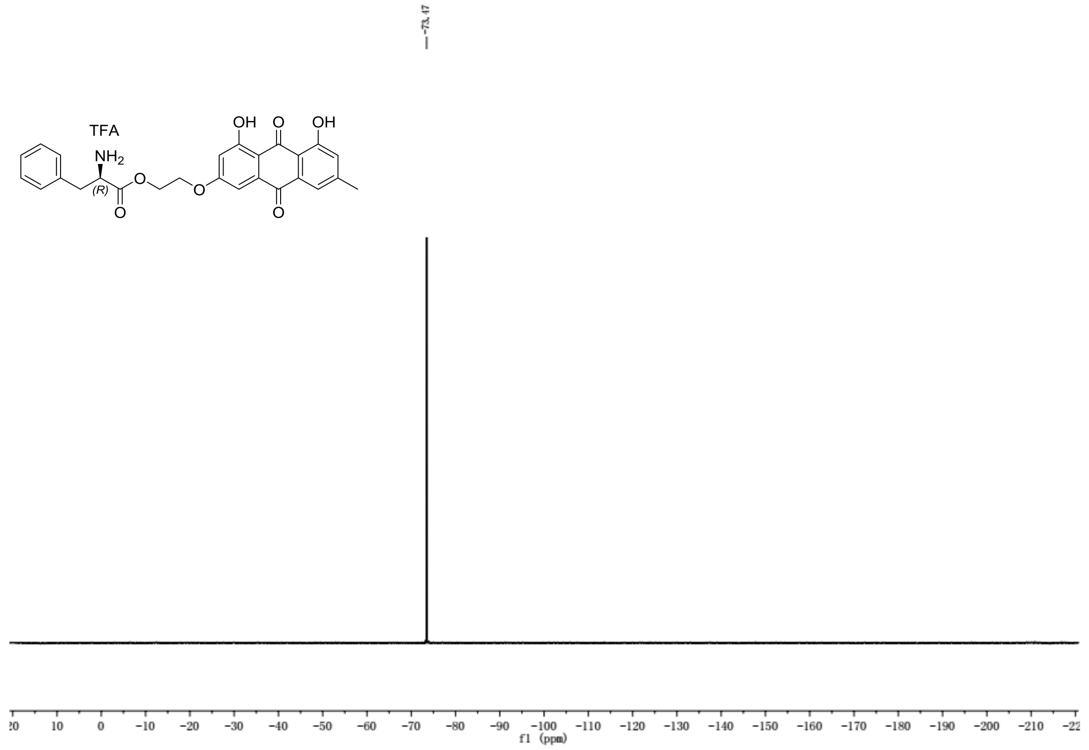


**Figure S71.** $^{19}$F NMR (376 MHz, DMSO–$d_6$) spectrum of compound **3w**.

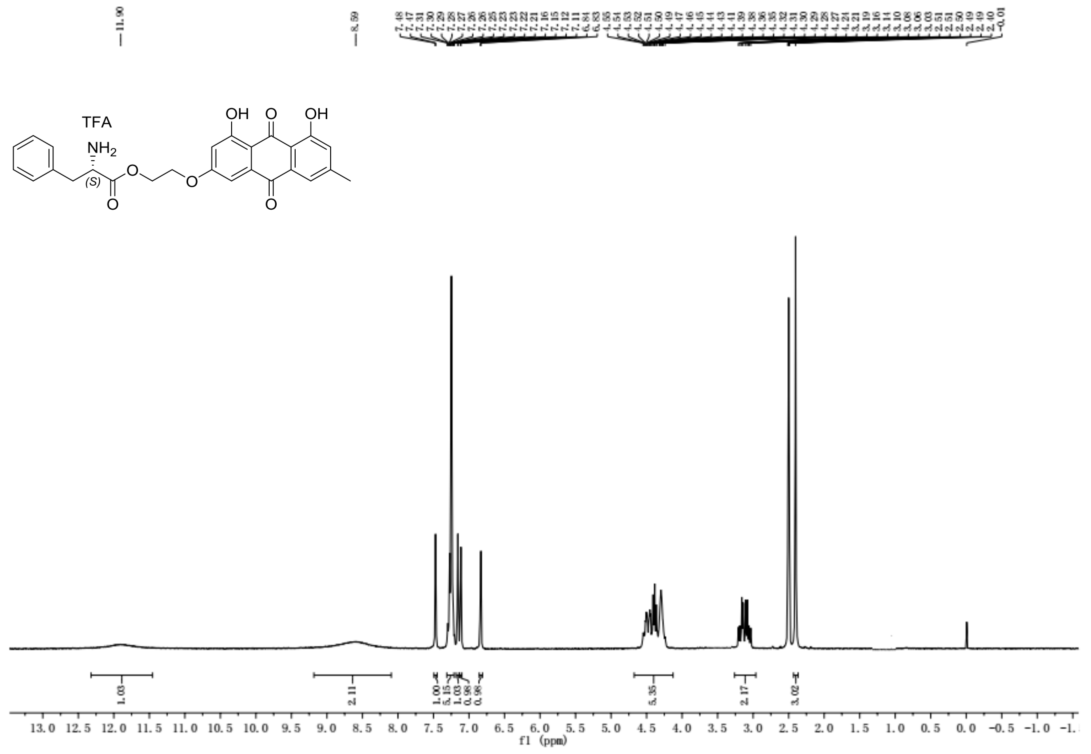


**Figure S72.** $^{1}$H NMR (400 MHz, DMSO–$d_6$) spectrum of compound **3x**.

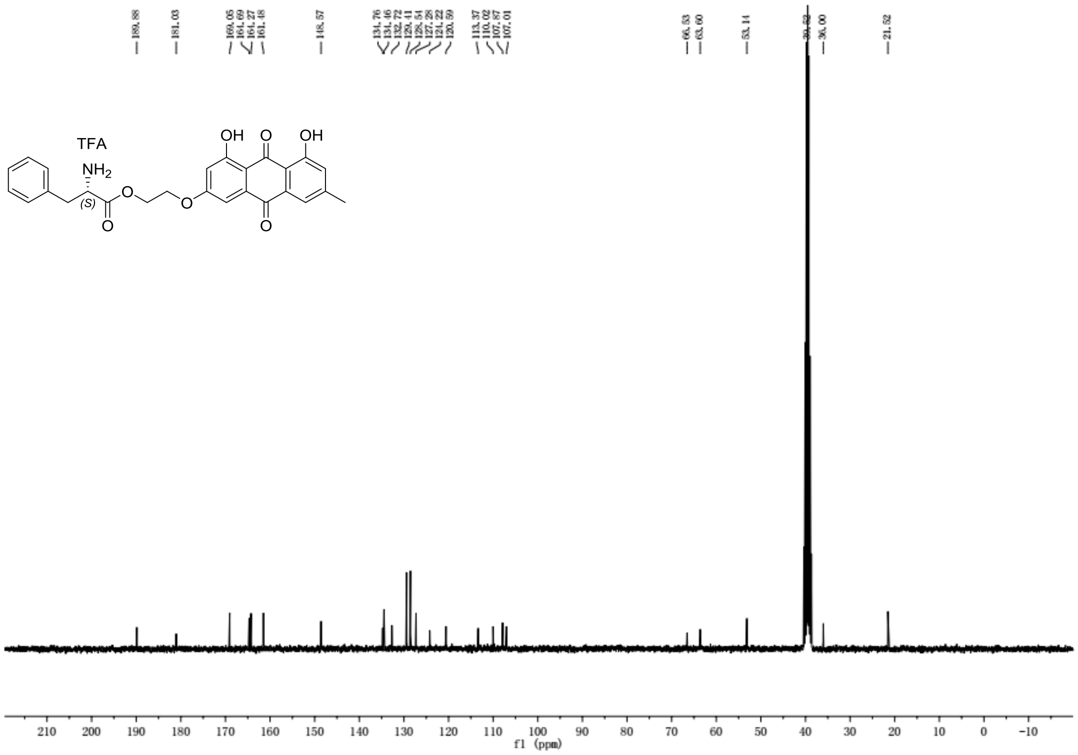

**Figure S73.** $^{13}$C NMR (101 MHz, DMSO–$d_6$) spectrum of compound **3x**.

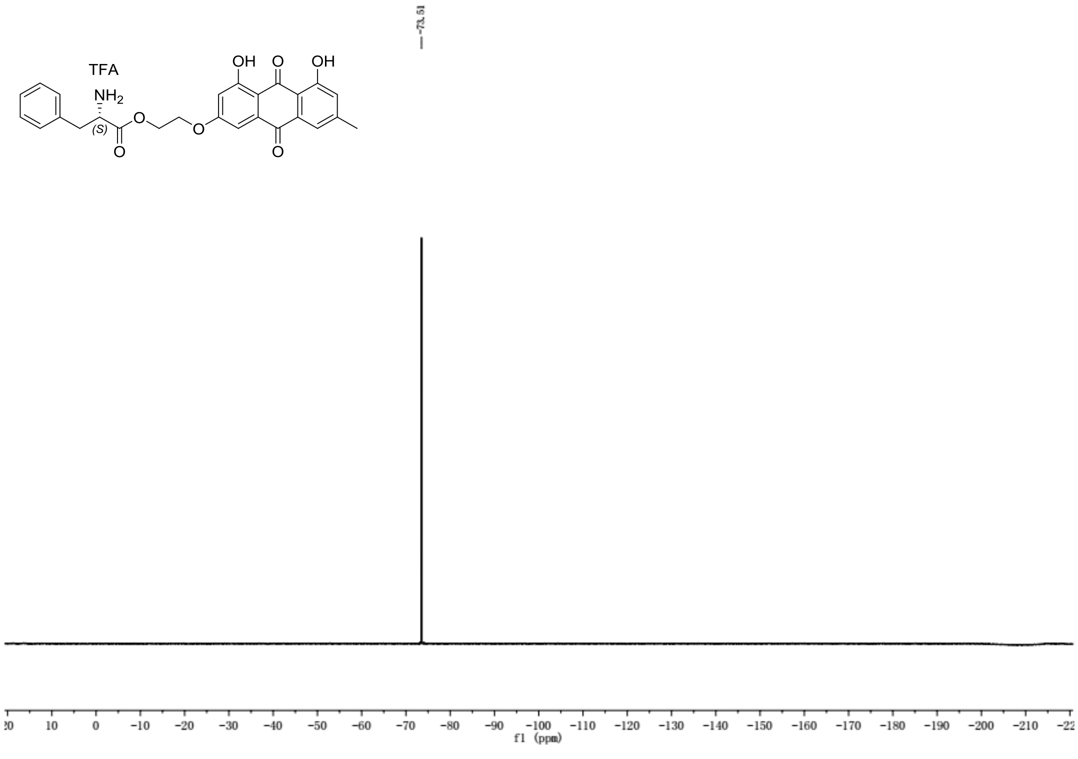

**Figure S74.** $^{19}$F NMR (376 MHz, DMSO–$d_6$) spectrum of compound **3x**.

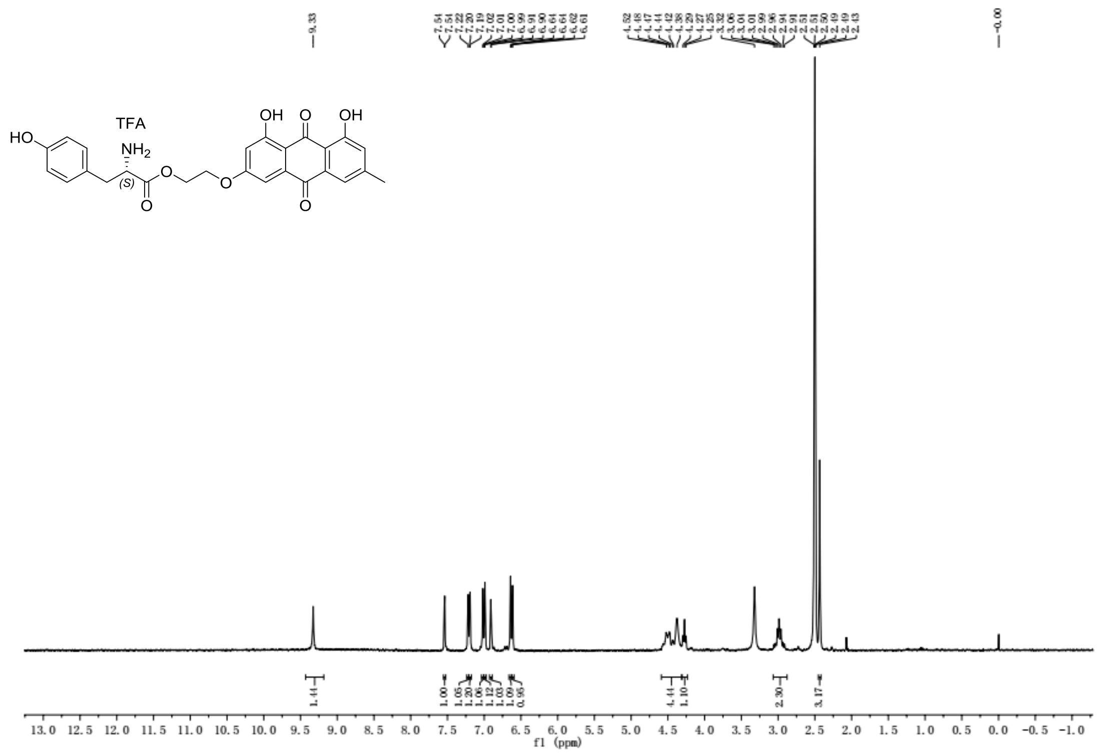

**Figure S75.** ${}^{1}$H NMR (400 MHz, DMSO–$d_6$) spectrum of compound **3y**.

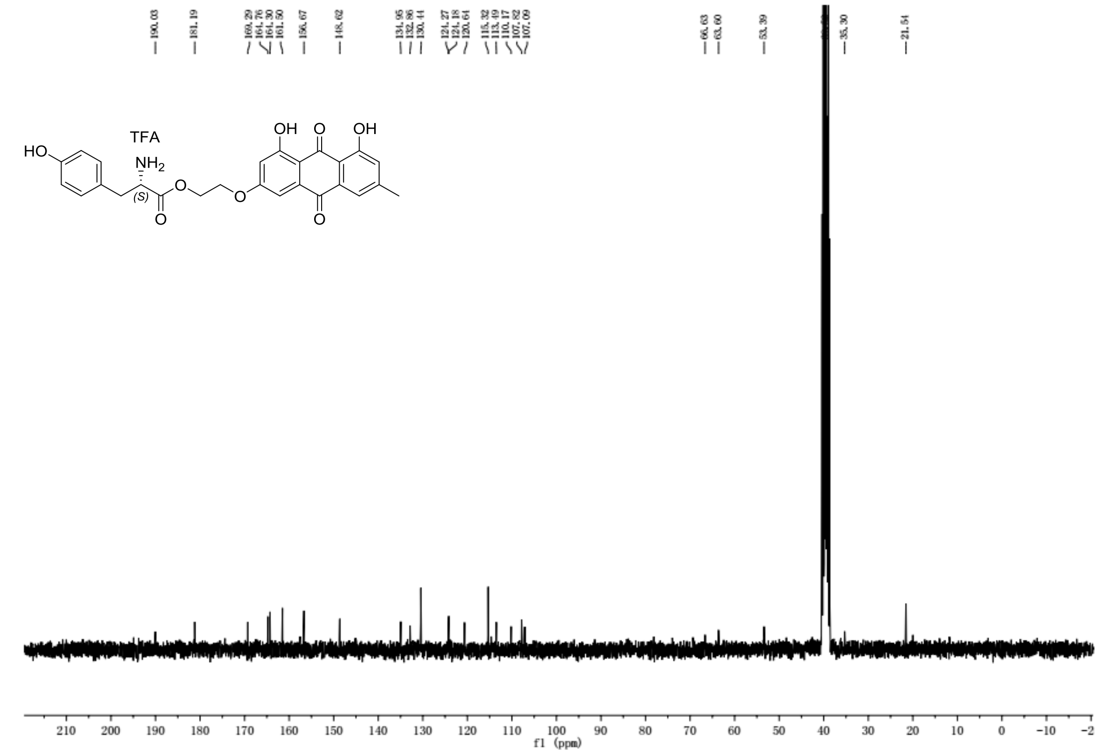

**Figure S76.** ${}^{13}$C NMR (101 MHz, DMSO–$d_6$) spectrum of compound **3y**.

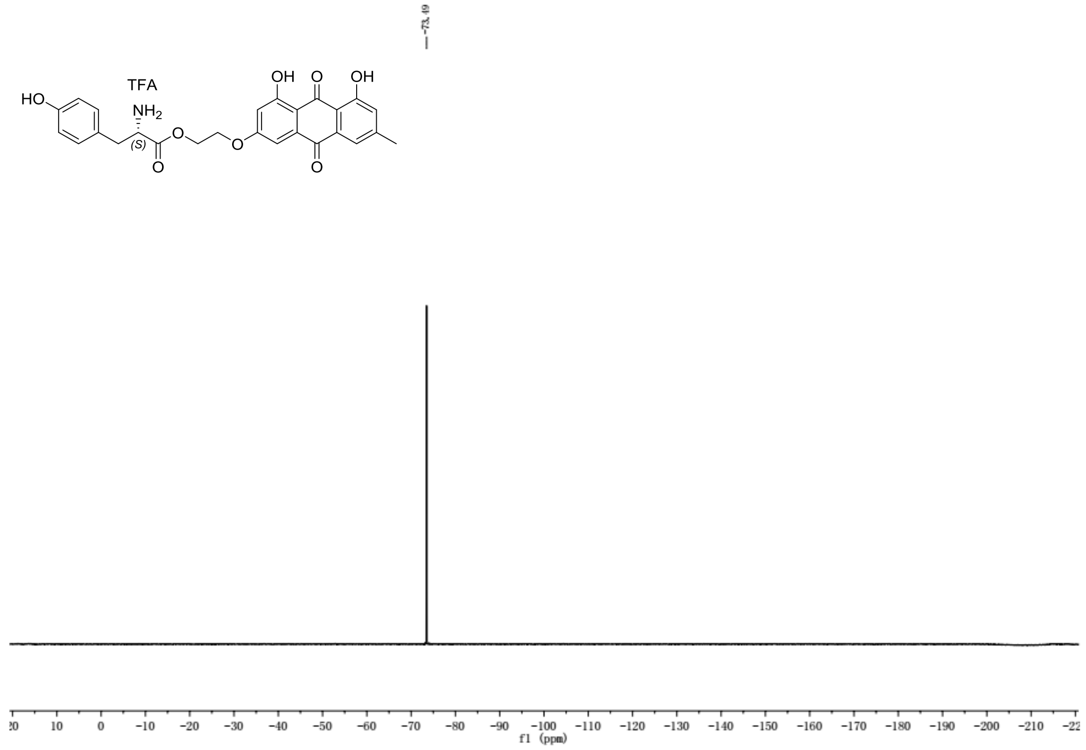

**Figure S77.** $^{19}$F NMR (376 MHz, DMSO–$d_6$) spectrum of compound **3y**.

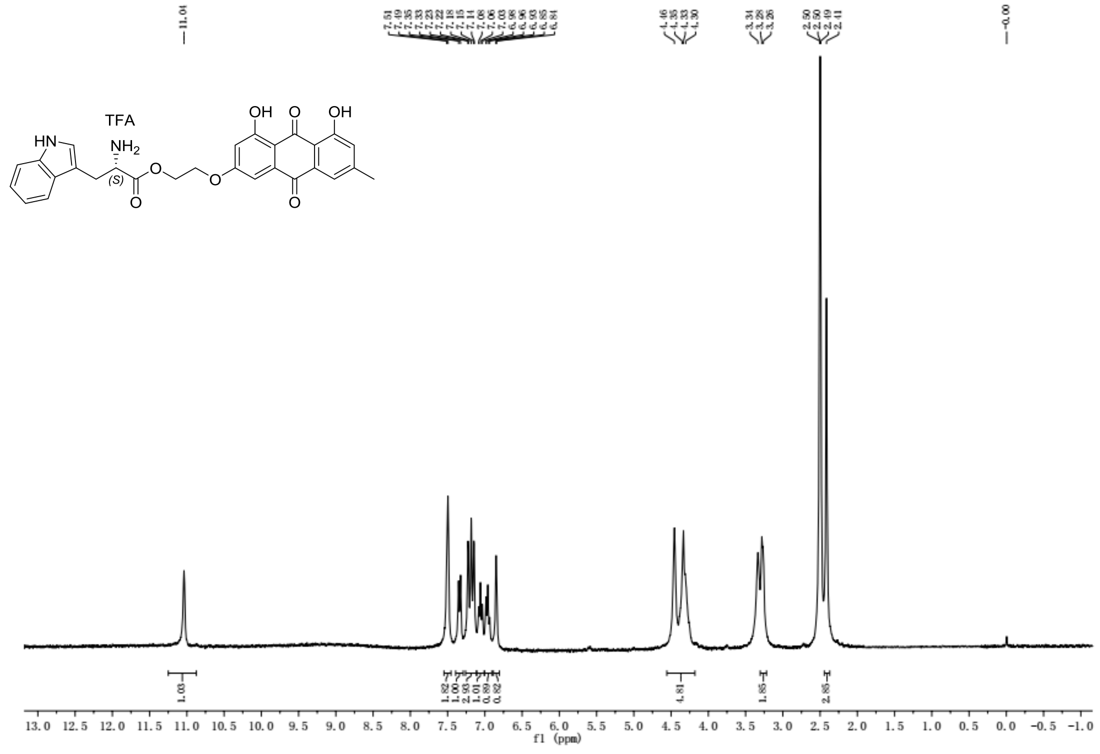

**Figure S78.** $^{1}$H NMR (400 MHz, DMSO–$d_6$) spectrum of compound **3z**.

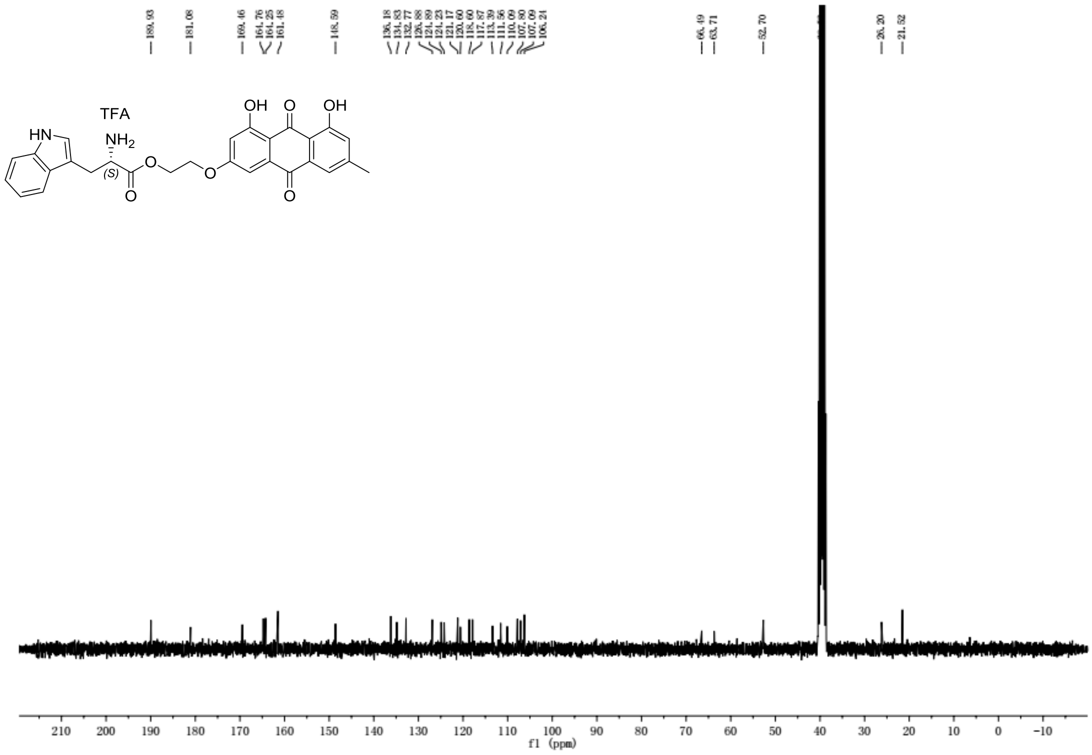


**Figure S79.** $^{13}$C NMR (101 MHz, DMSO–$d_6$) spectrum of compound **3z**.

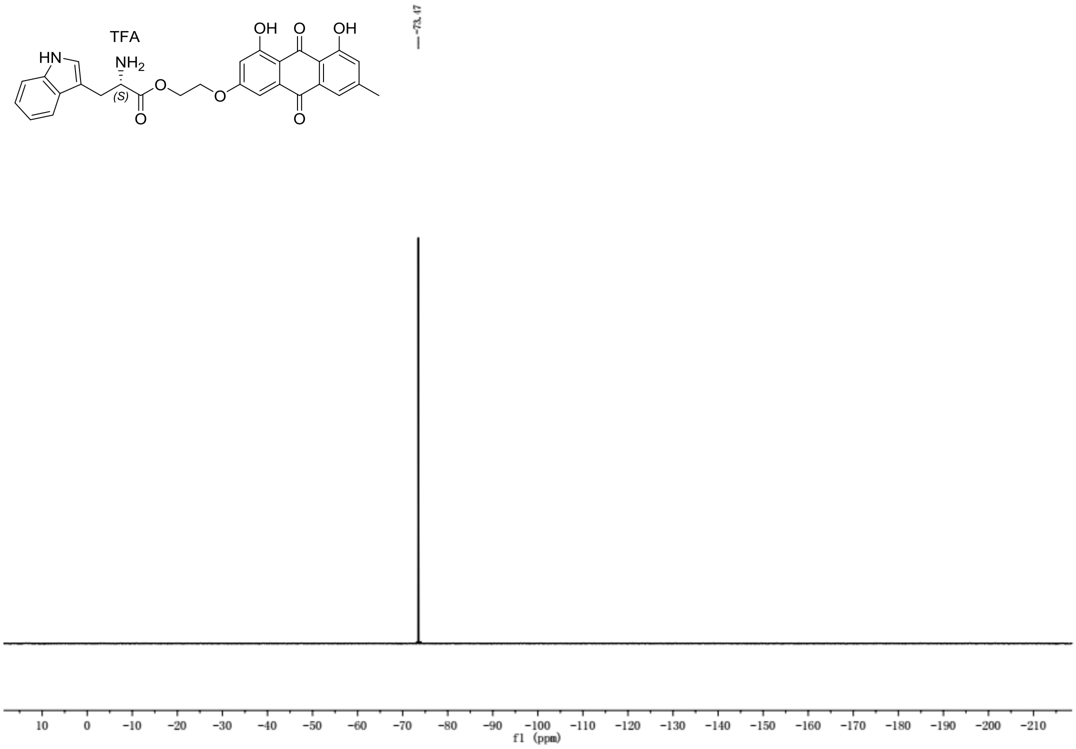


**Figure S80.** $^{19}$F NMR (376 MHz, DMSO–$d_6$) spectrum of compound **3z**.

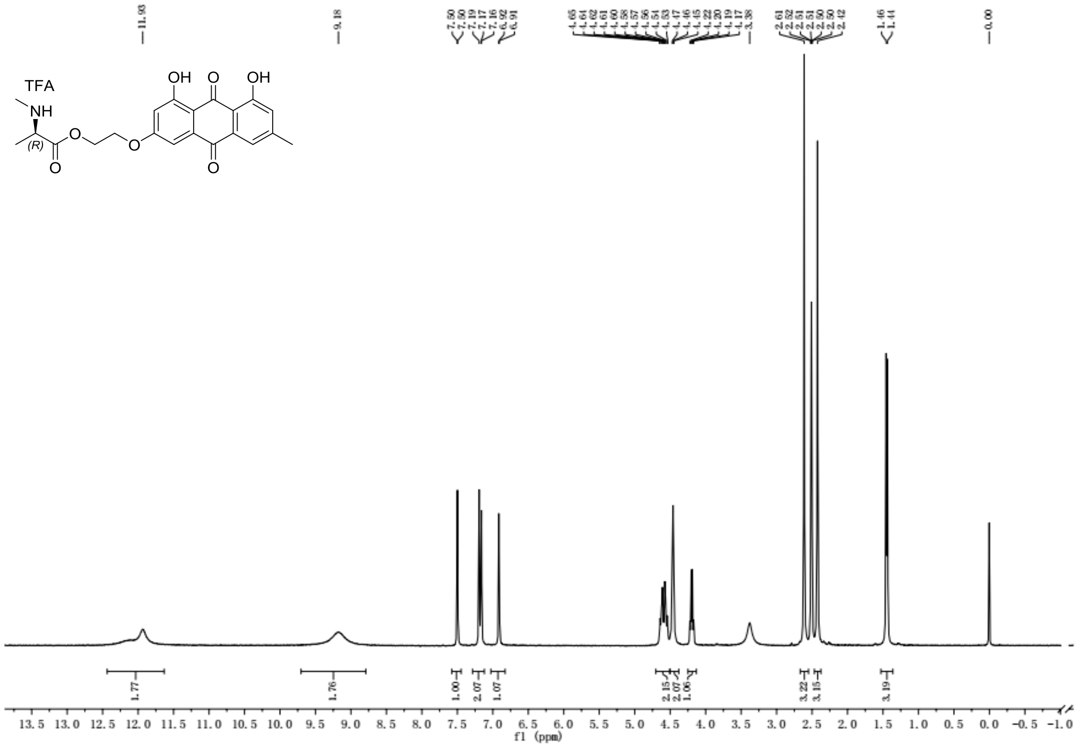

**Figure S81.** $^{1}$H NMR (400 MHz, DMSO–$d_6$) spectrum of compound **4a**.

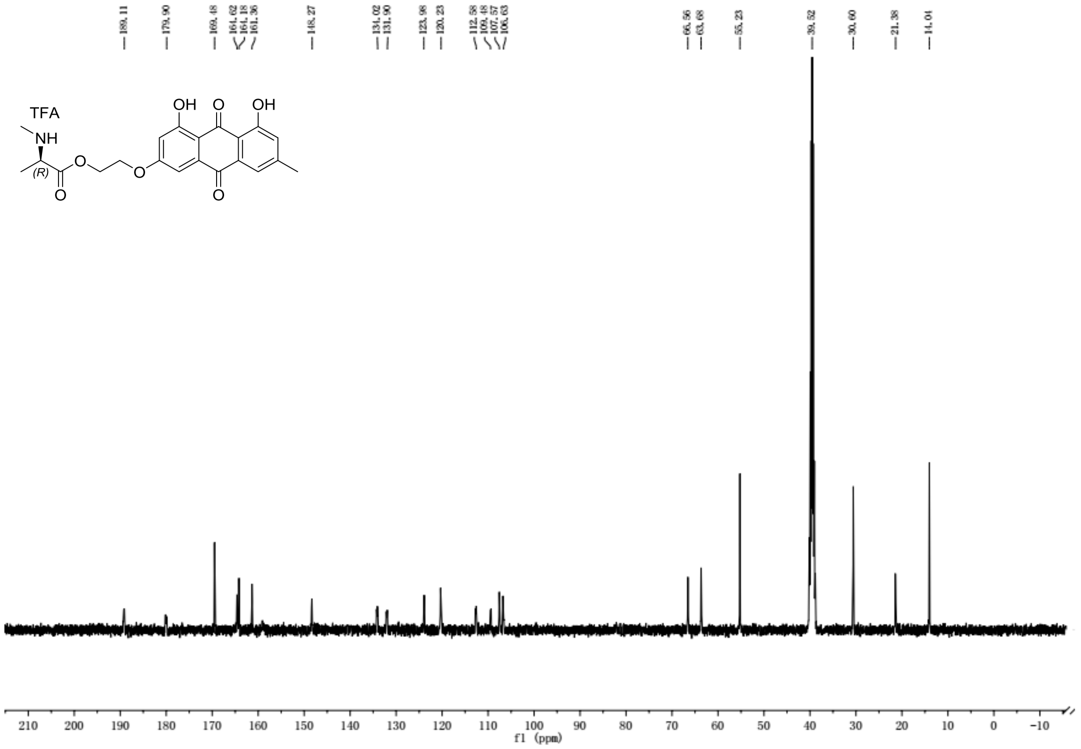

**Figure S82.** $^{13}$C NMR (101 MHz, DMSO–$d_6$) spectrum of compound **4a**.

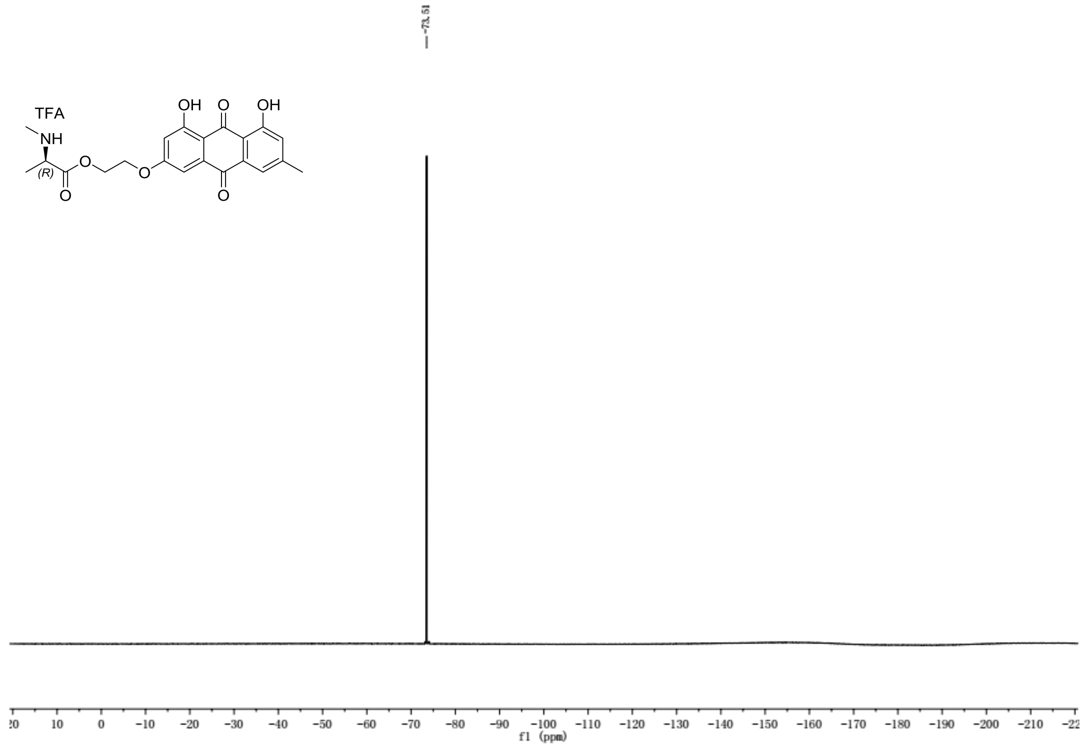

**Figure S83.** $^{19}$F NMR (376 MHz, DMSO–$d_6$) spectrum of compound **4a**.

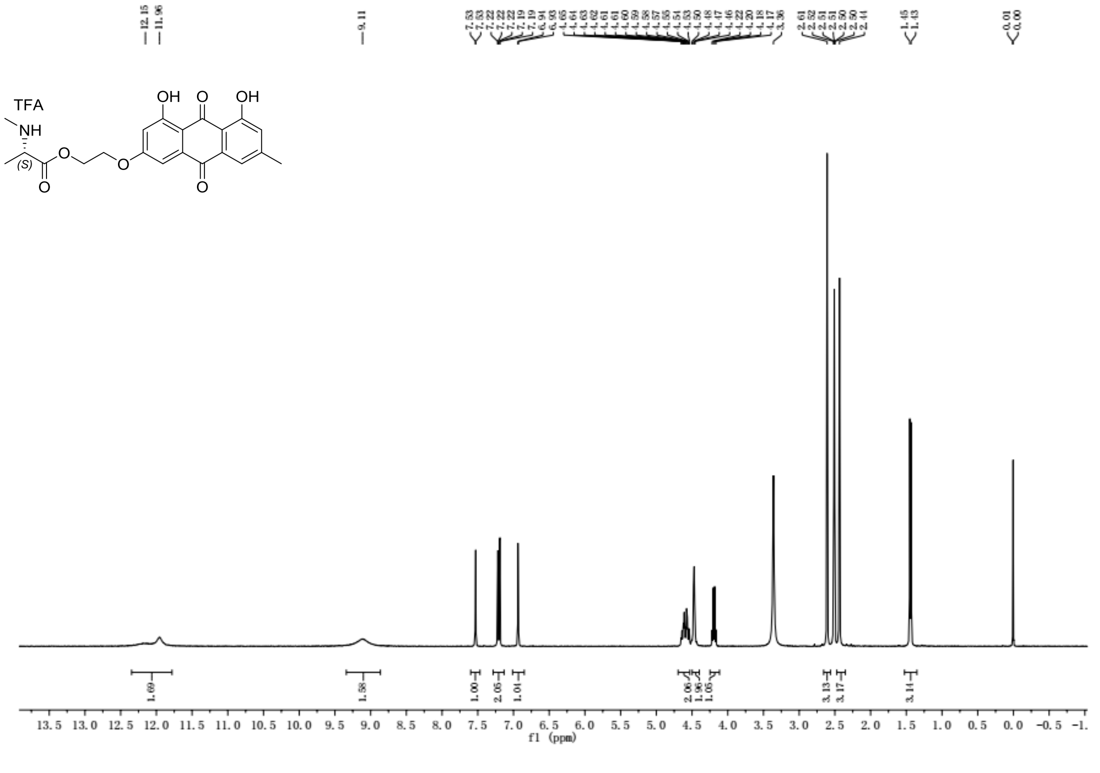

**Figure S84.** $^{1}$H NMR (400 MHz, DMSO–$d_6$) spectrum of compound **4b**.

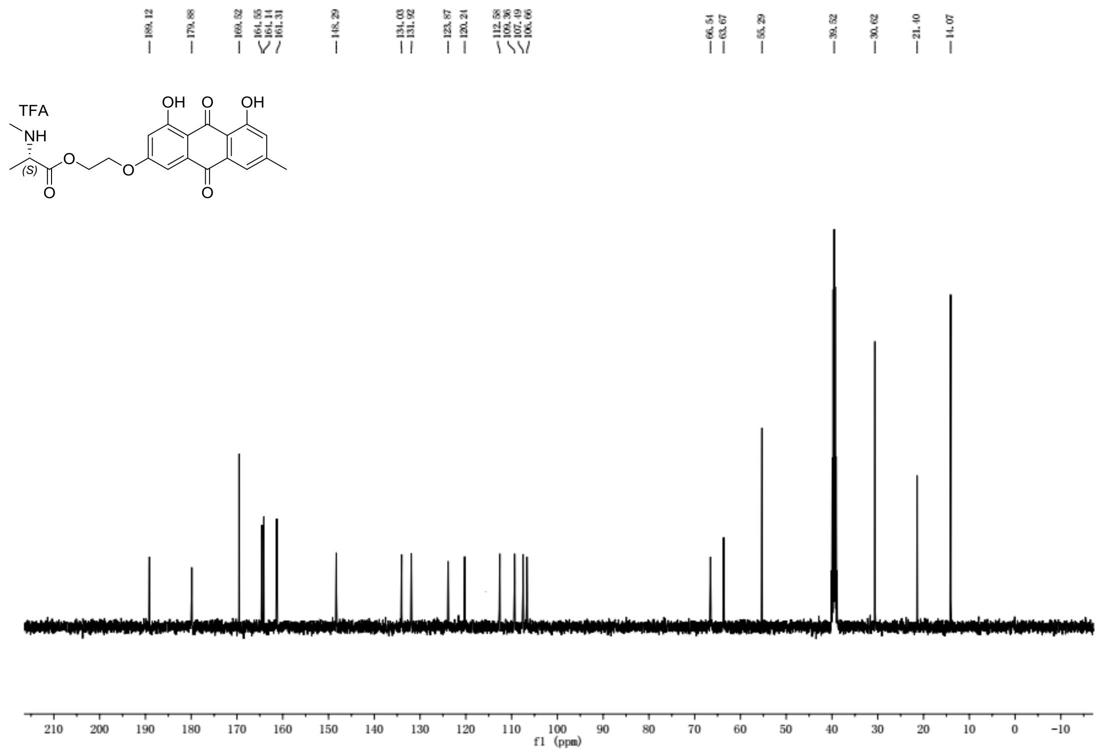


**Figure S85.** $^{13}$C NMR (101 MHz, DMSO–$d_6$) spectrum of compound **4b**.

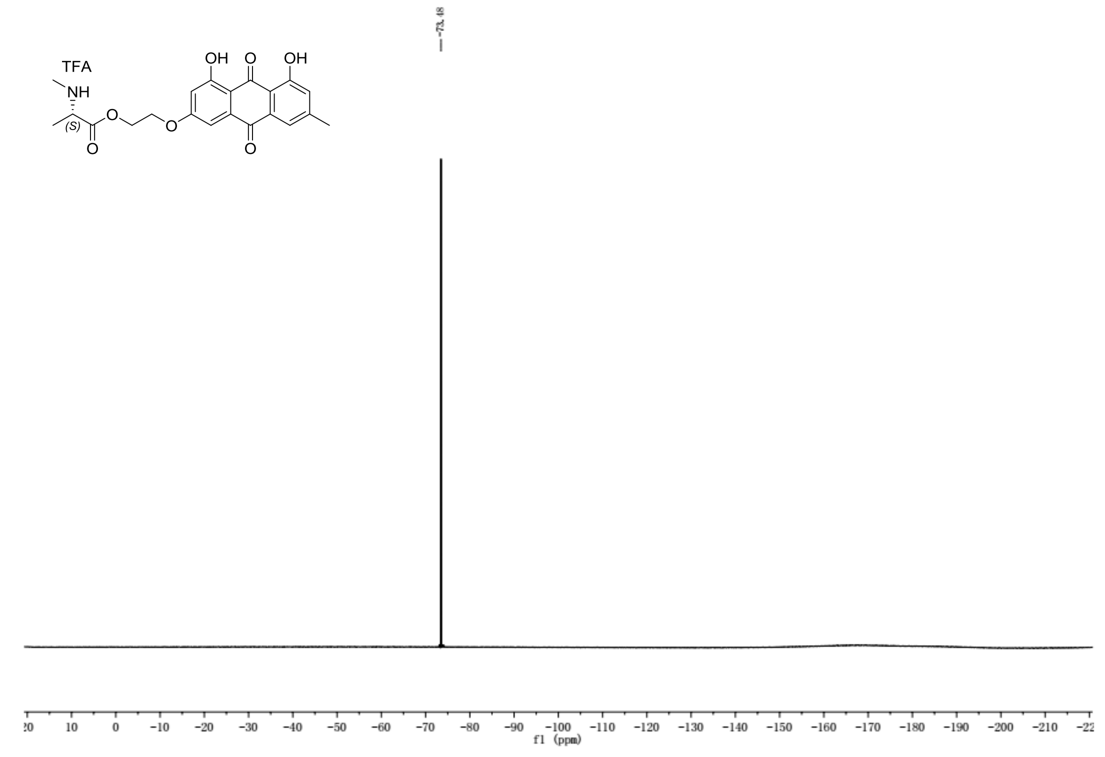


**Figure S86.** $^{19}$F NMR (376 MHz, DMSO–$d_6$) spectrum of compound **4b**.

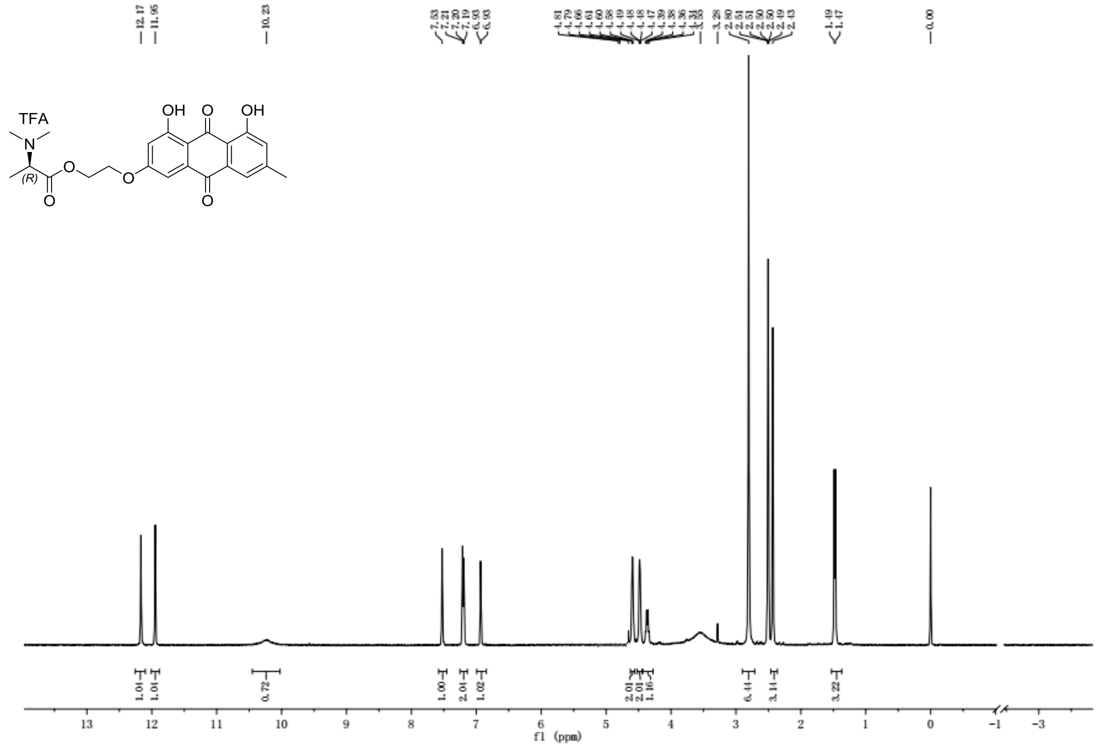

**Figure S87.** $^{1}H$ NMR (400 MHz, DMSO–$d_6$) spectrum of compound **5a**.

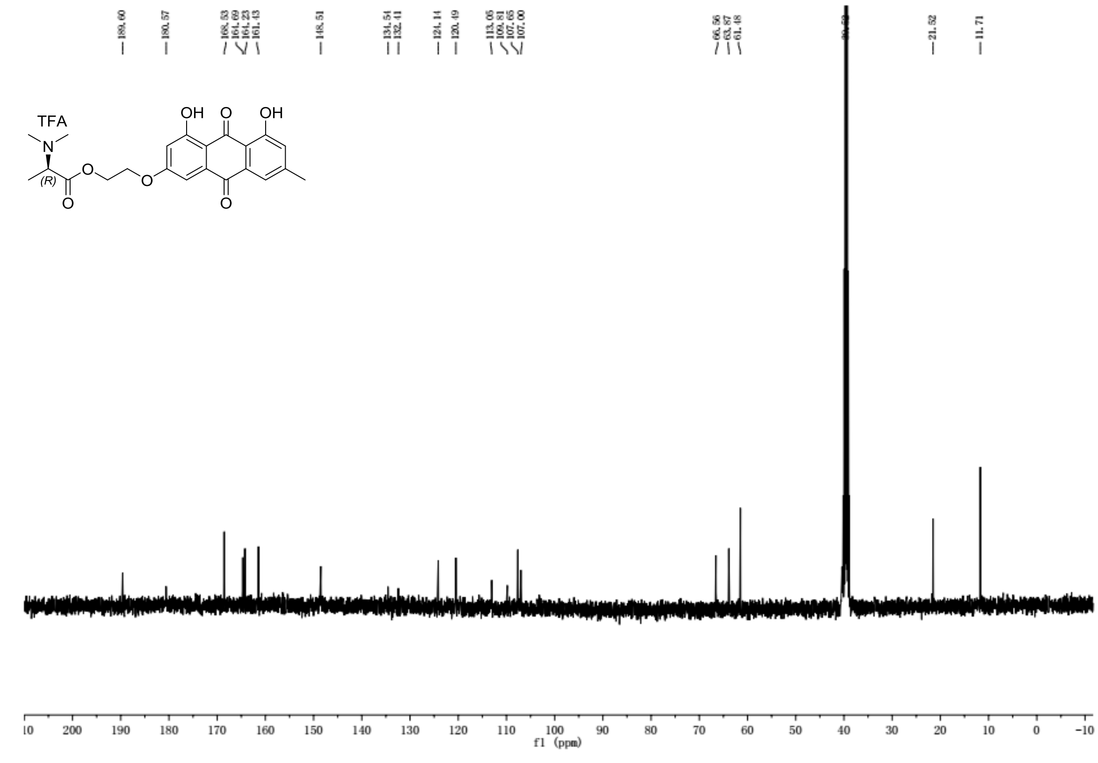

**Figure S88.** $^{13}C$ NMR (101 MHz, DMSO–$d_6$) spectrum of compound **5a**.

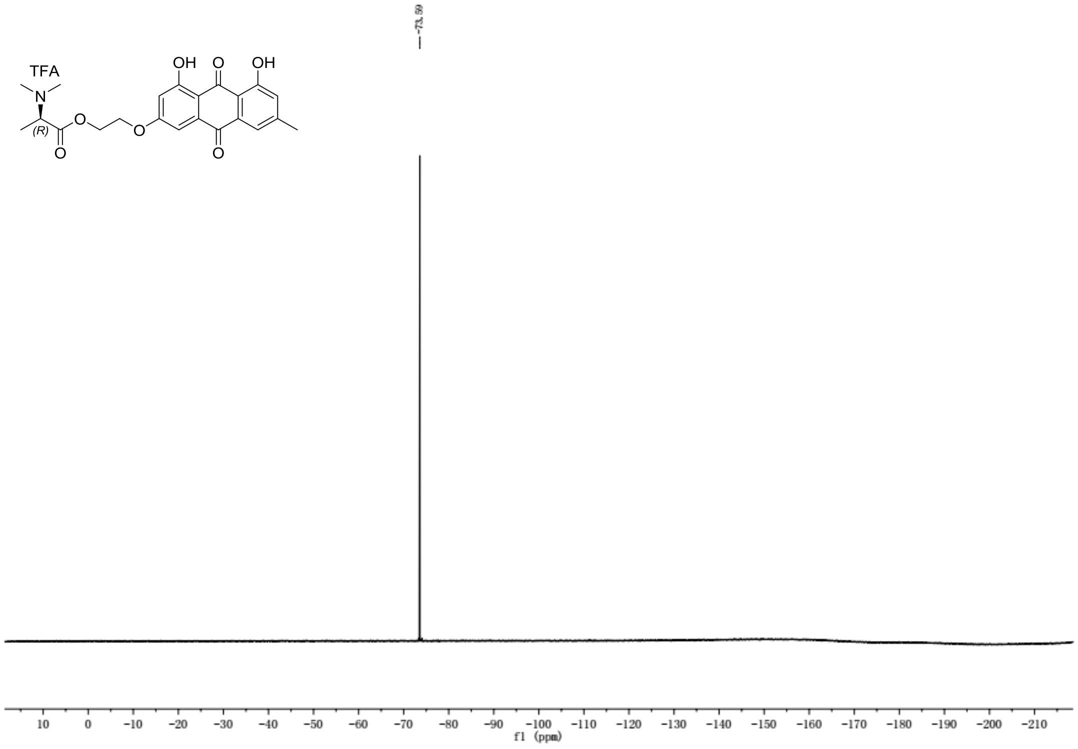


**Figure S89.** $^{19}$F NMR (376 MHz, DMSO–$d_6$) spectrum of compound **5a**.

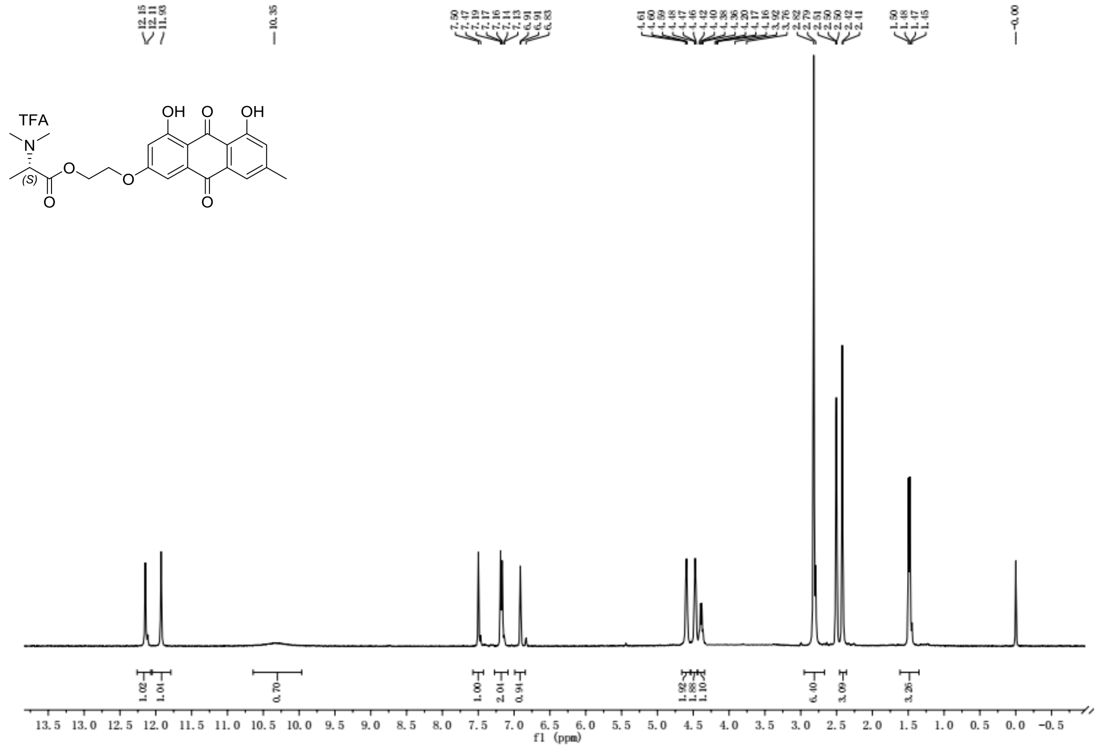


**Figure S90.** $^{1}$H NMR (400 MHz, DMSO–$d_6$) spectrum of compound **5b**.

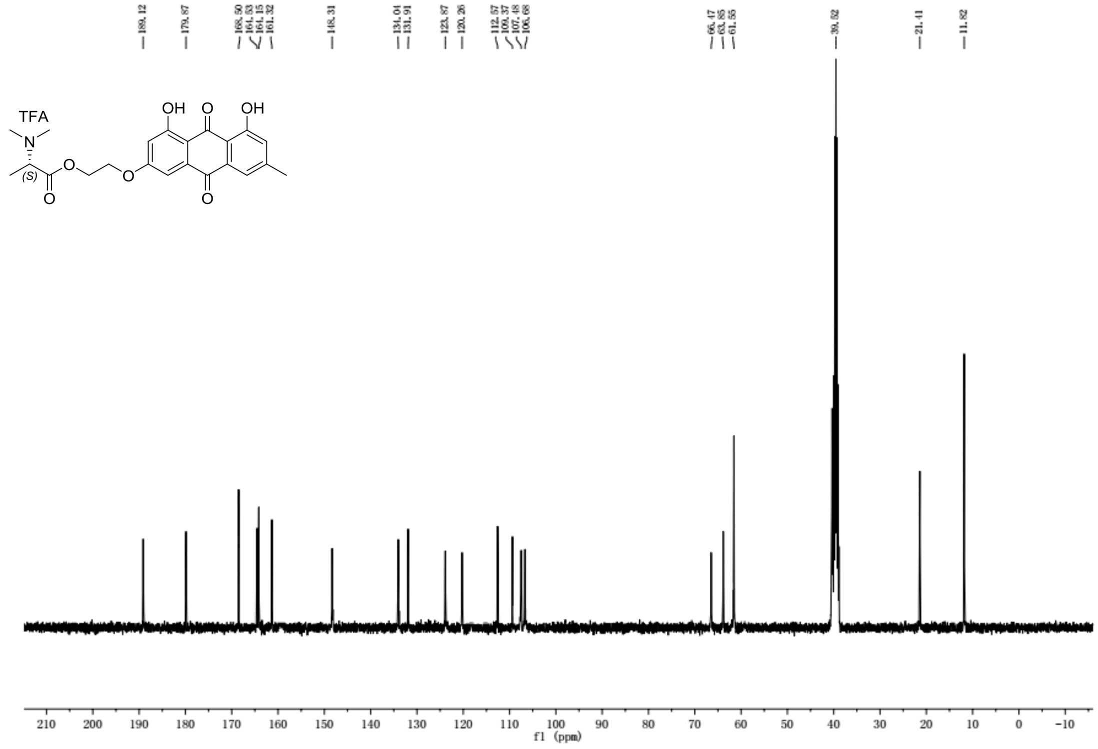


**Figure S91.** $^{13}C$ NMR (101 MHz, DMSO–$d_6$) spectrum of compound **5b**.

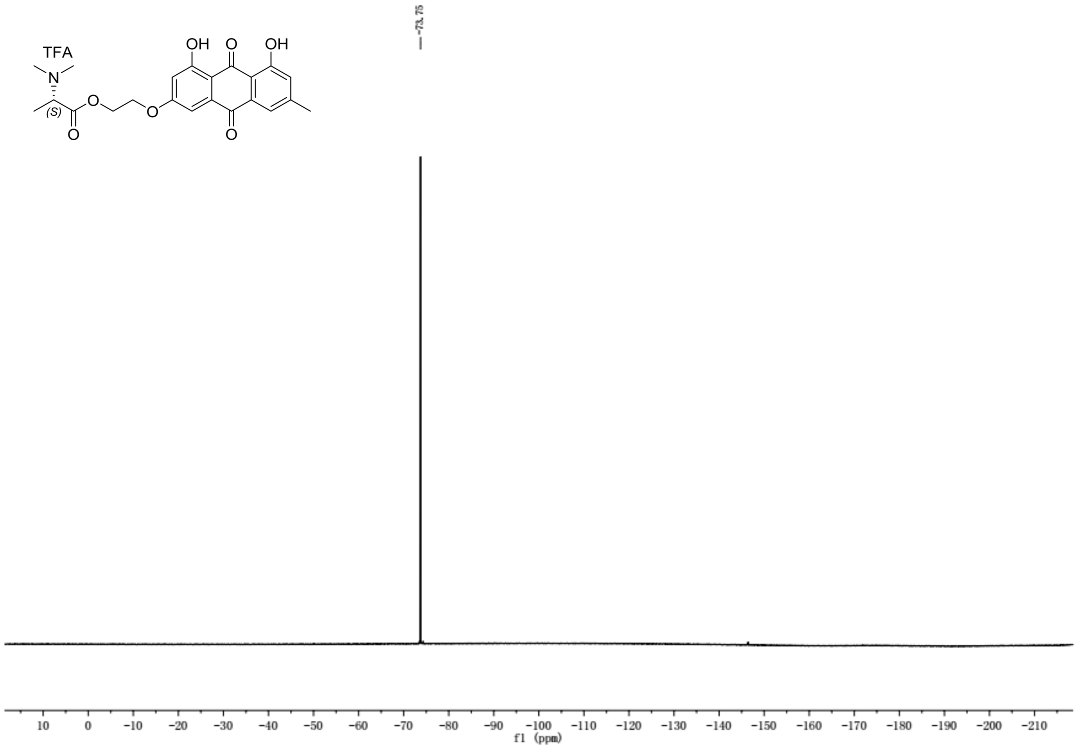


**Figure S92.** $^{19}F$ NMR (376 MHz, DMSO–$d_6$) spectrum of compound **5b**.

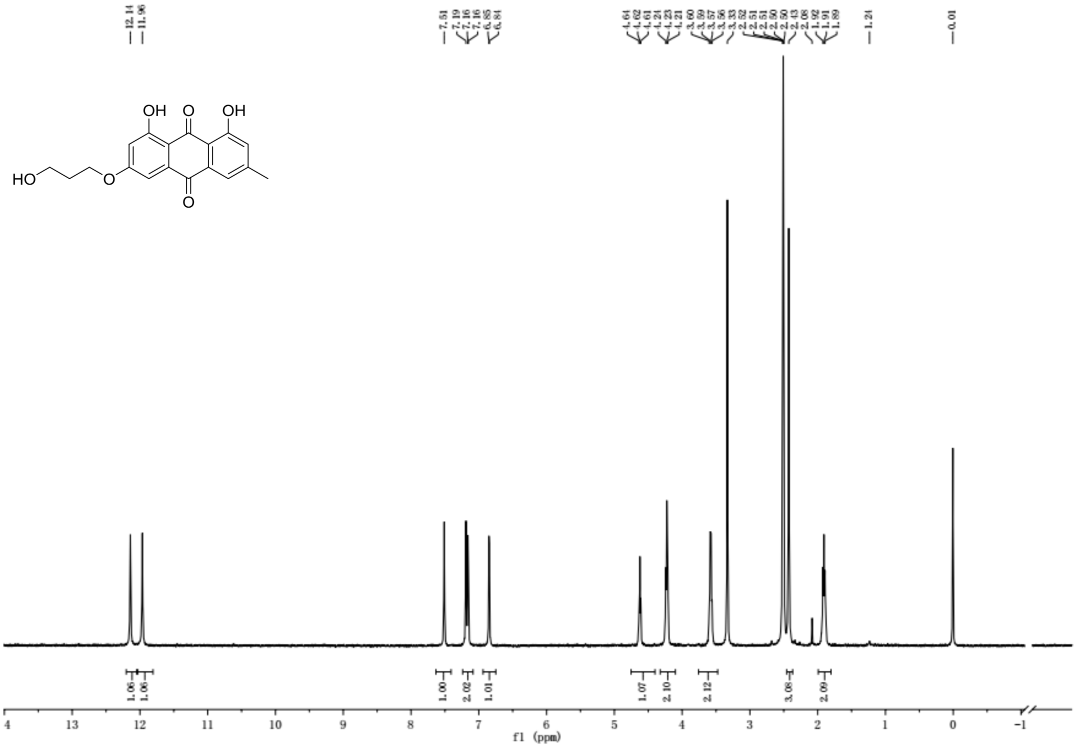


**Figure S93.** $^{1}$H NMR (400 MHz, DMSO–$d_6$) spectrum of compound **6a**.

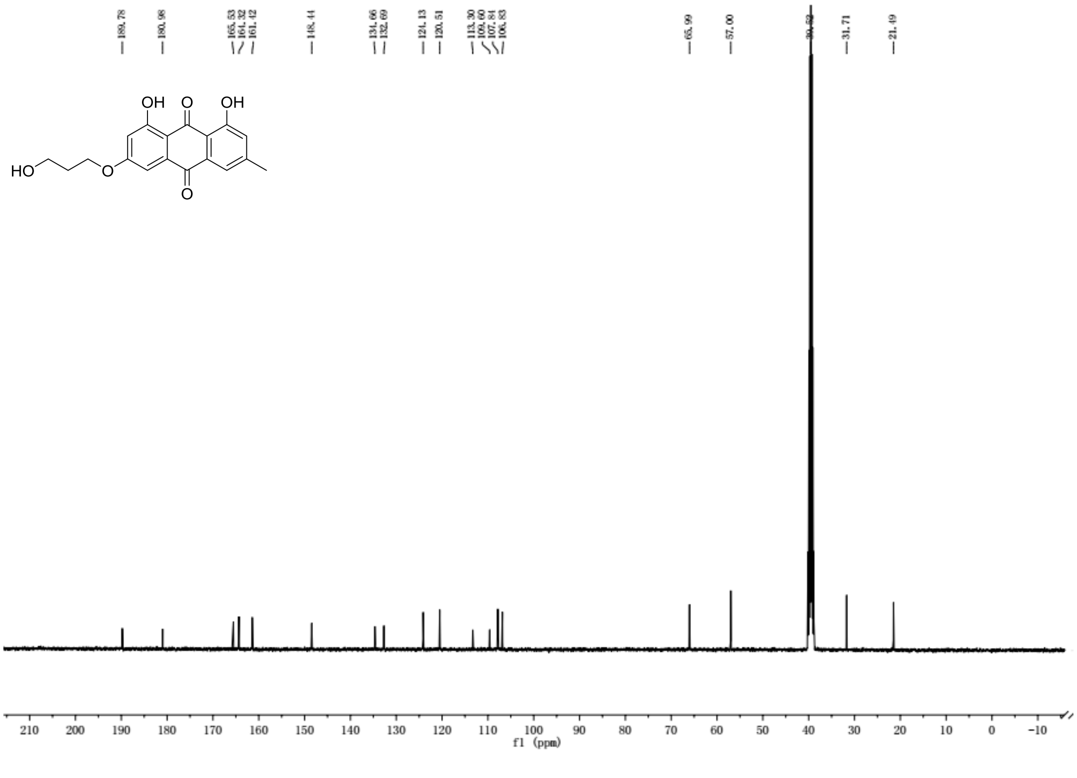


**Figure S94.** $^{13}$C NMR (101 MHz, DMSO–$d_6$) spectrum of compound **6a**.

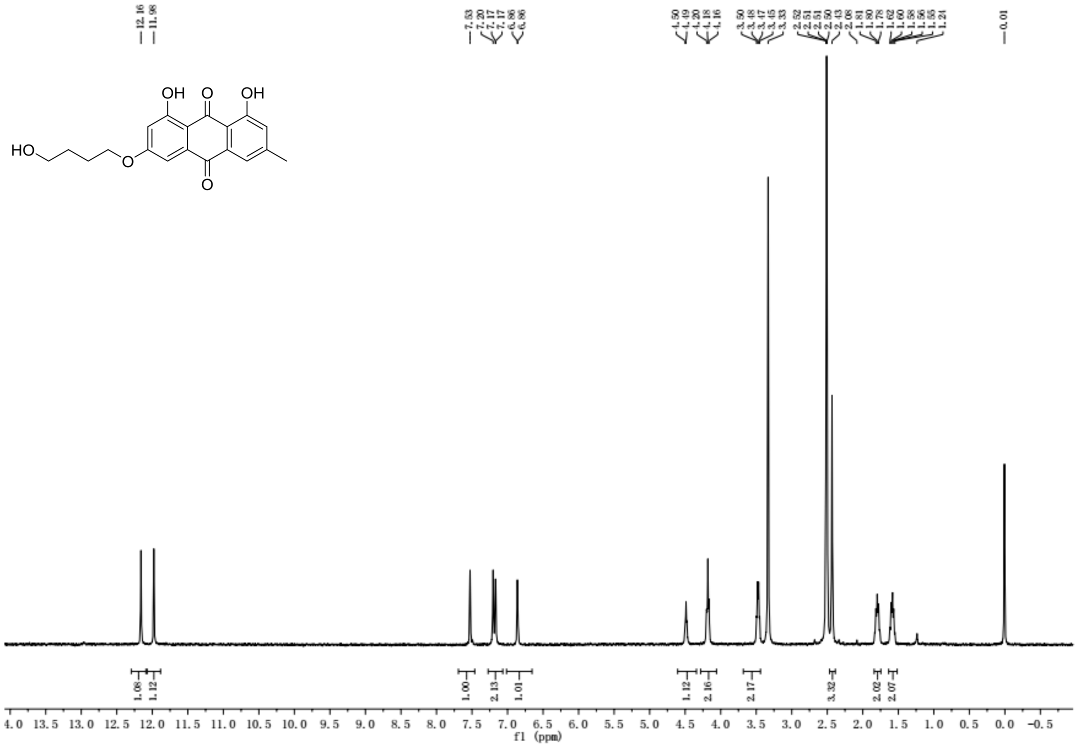


**Figure S95.** $^{1}$H NMR (400 MHz, DMSO–$d_6$) spectrum of compound **6b**.

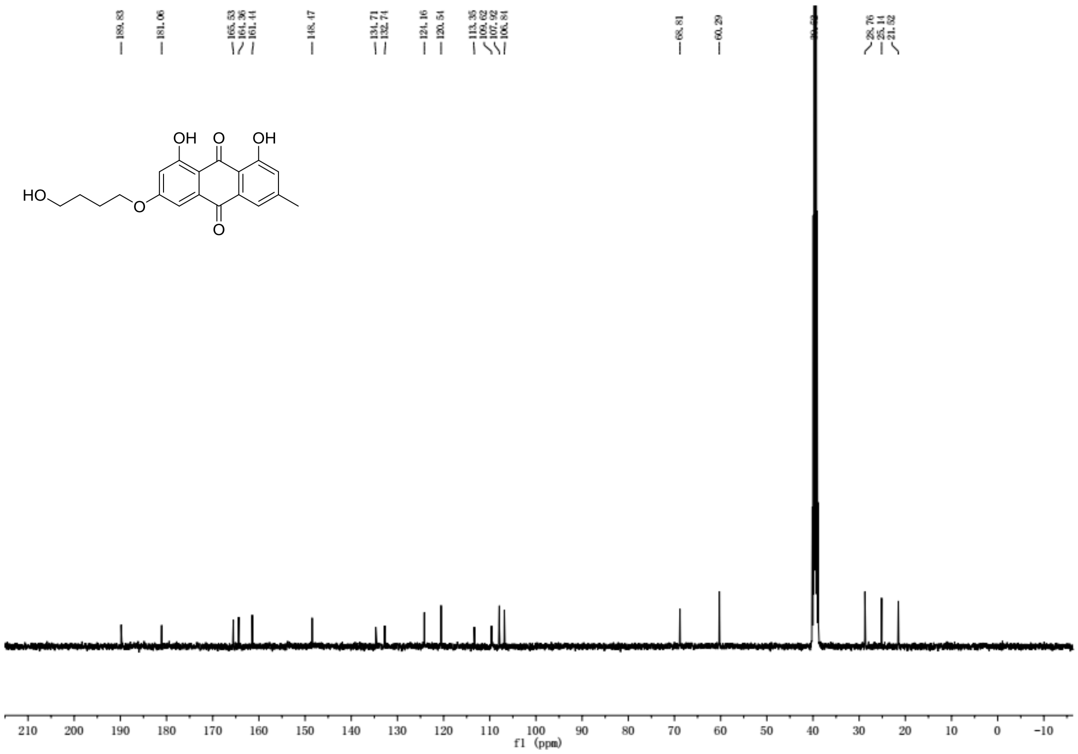


**Figure S96.** $^{13}$C NMR (101 MHz, DMSO–$d_6$) spectrum of compound **6b**.

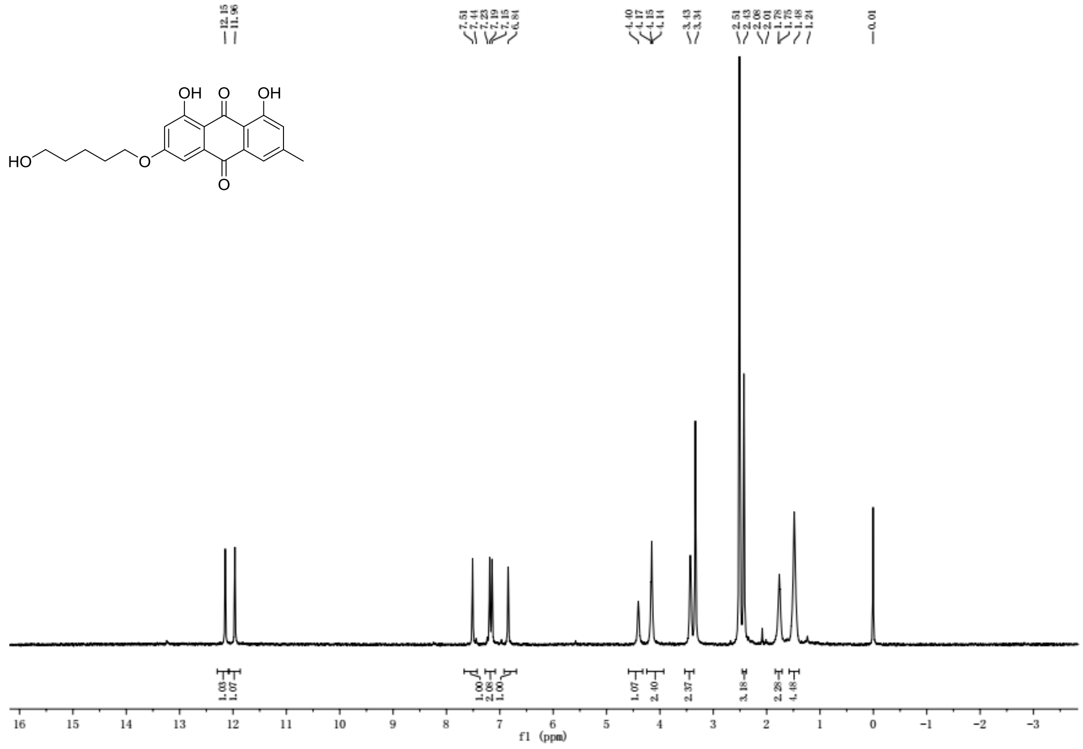


**Figure S97.** $^{1}H$ NMR (400 MHz, DMSO–$d_6$) spectrum of compound **6c**.

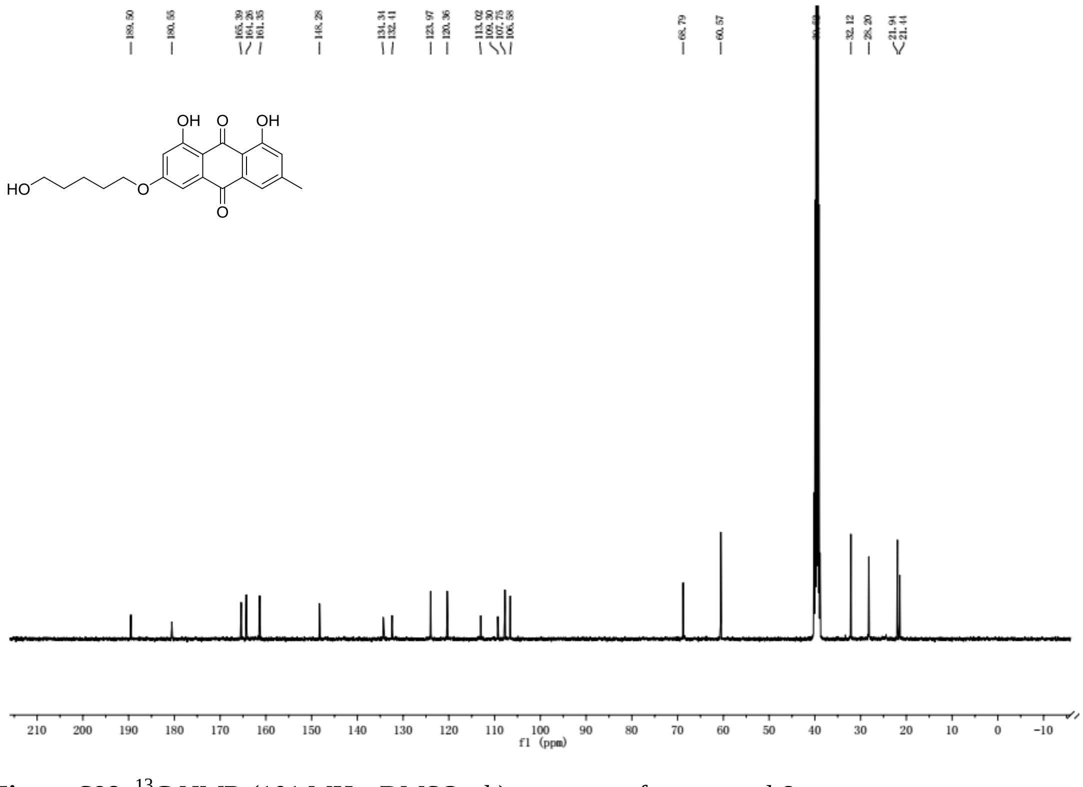


**Figure S98.** $^{13}C$ NMR (101 MHz, DMSO–$d_6$) spectrum of compound **6c**

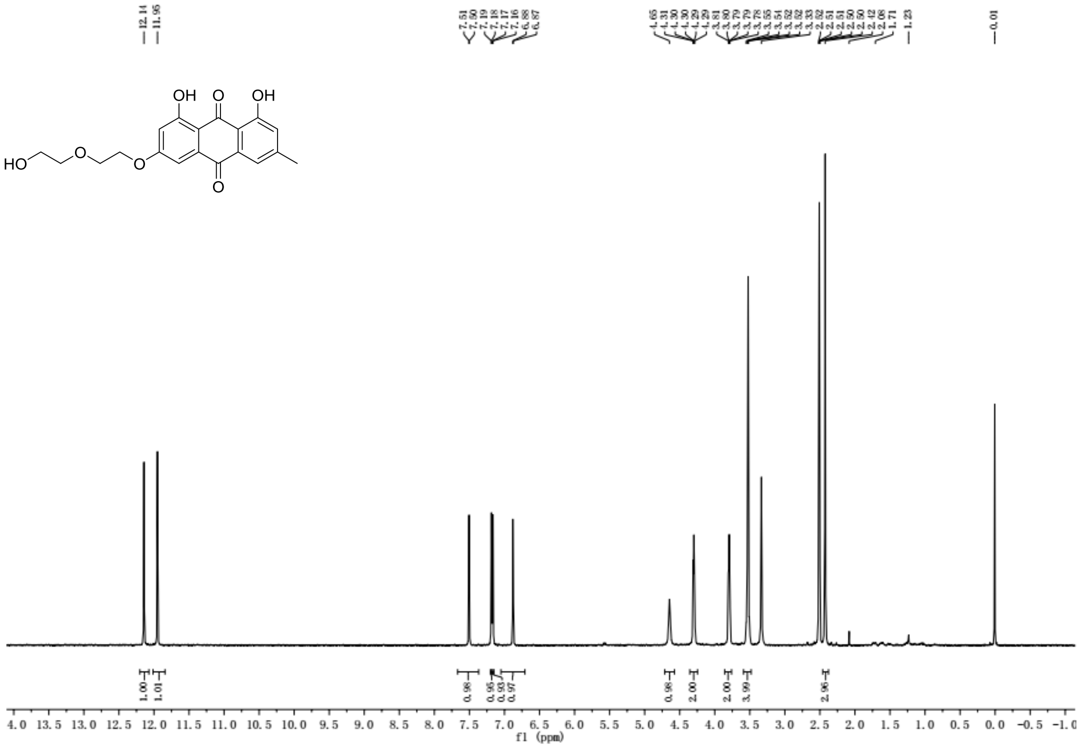

**Figure S99.** $^{1}$H NMR (400 MHz, DMSO–$d_6$) spectrum of compound **6d**.

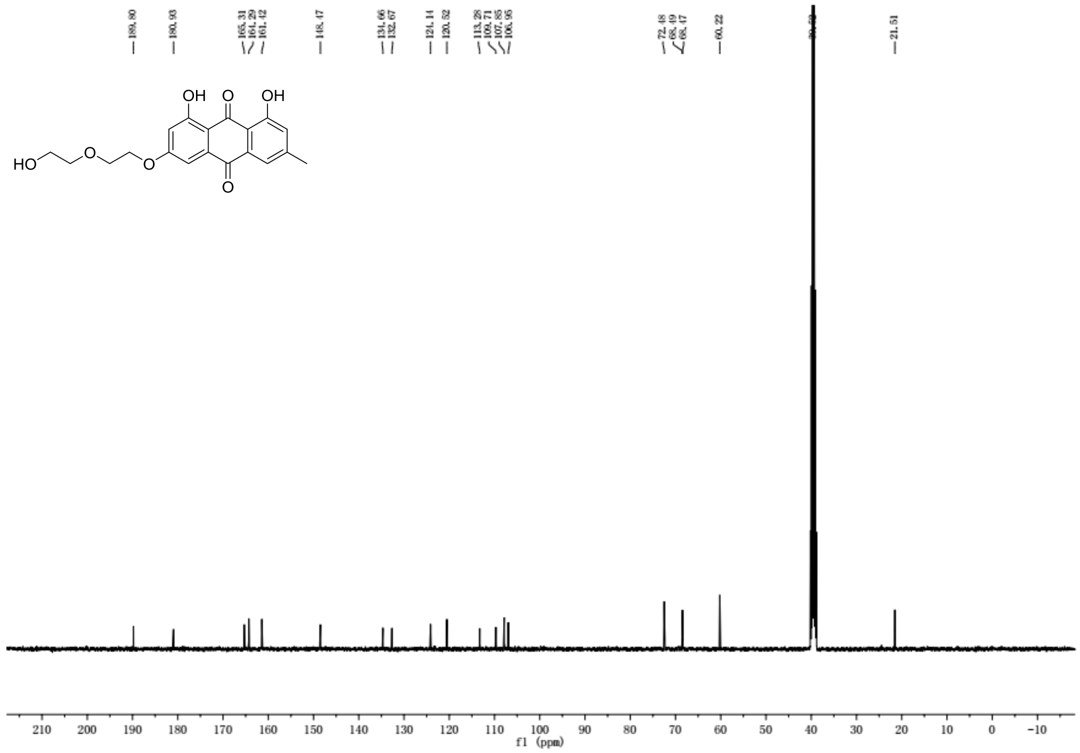

**Figure S100.** $^{13}$C NMR (101 MHz, DMSO–$d_6$) spectrum of compound **6d**.

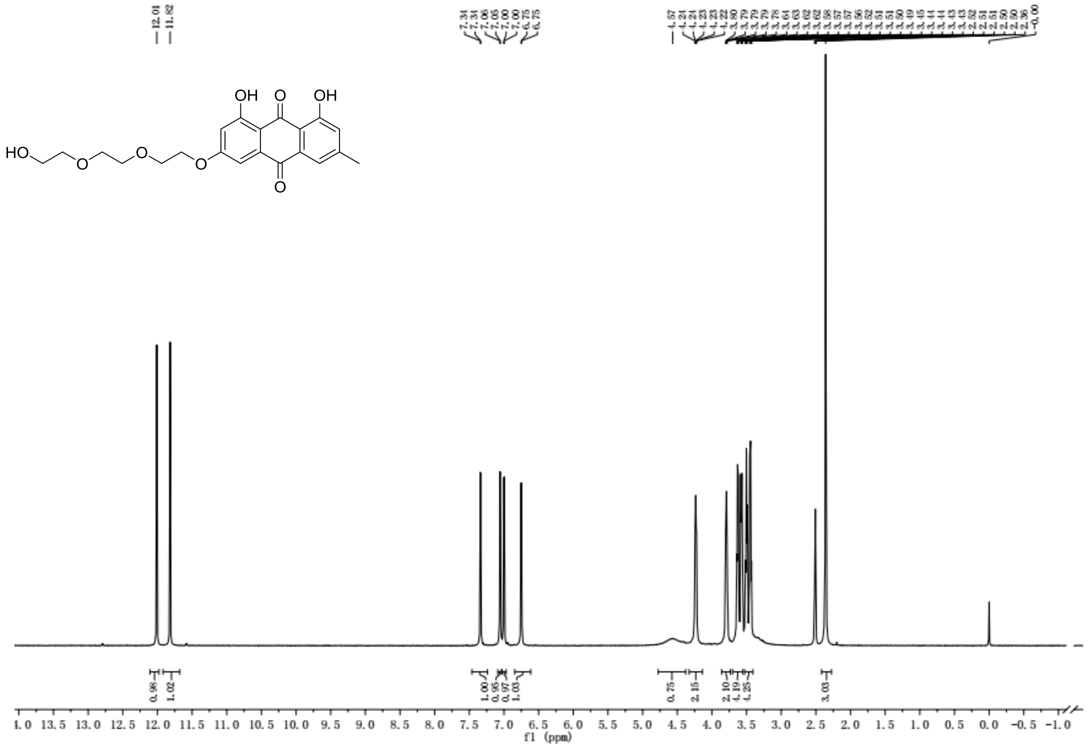

**Figure S101.** $^{1}$H NMR (400 MHz, DMSO–$d_6$) spectrum of compound **6e**.

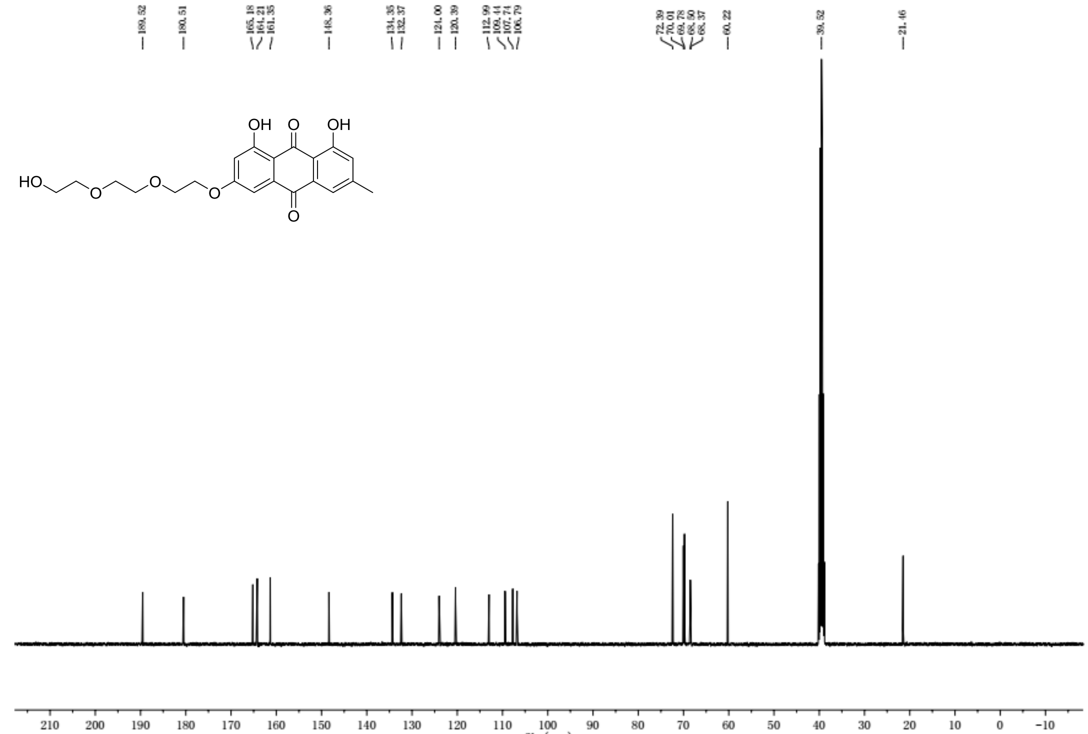

**Figure S102.** $^{13}$C NMR (101 MHz, DMSO–$d_6$) spectrum of compound **6e**.

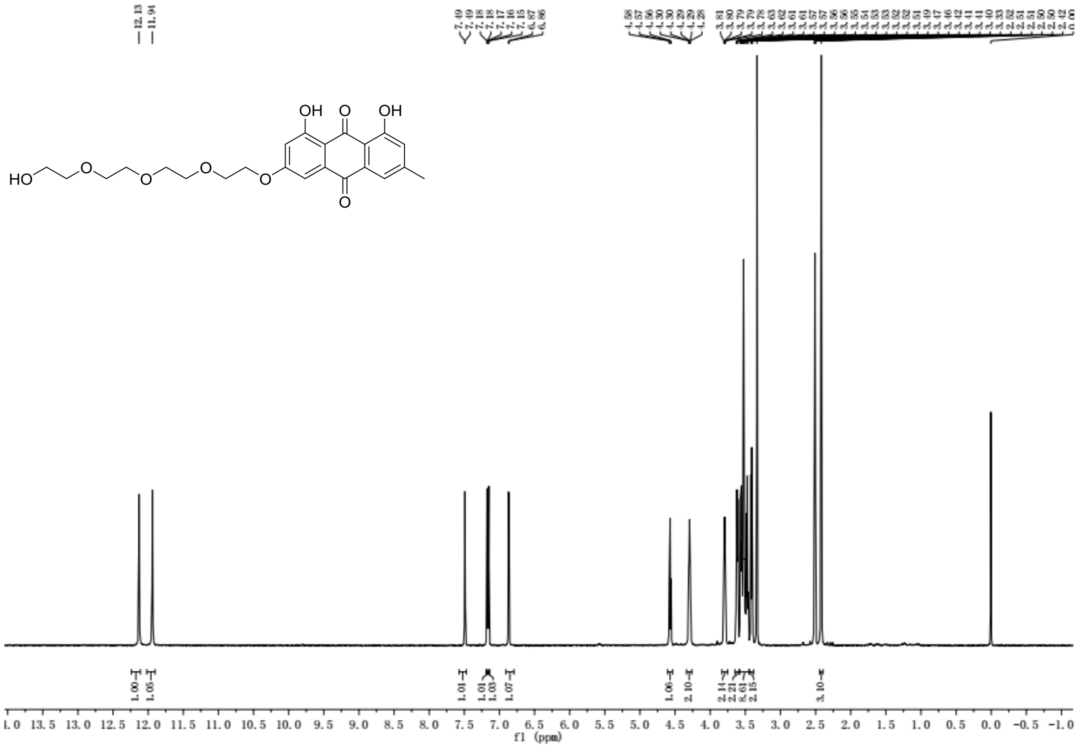

**Figure S103.** $^{1}$H NMR (400 MHz, DMSO–$d_6$) spectrum of compound **6f**.

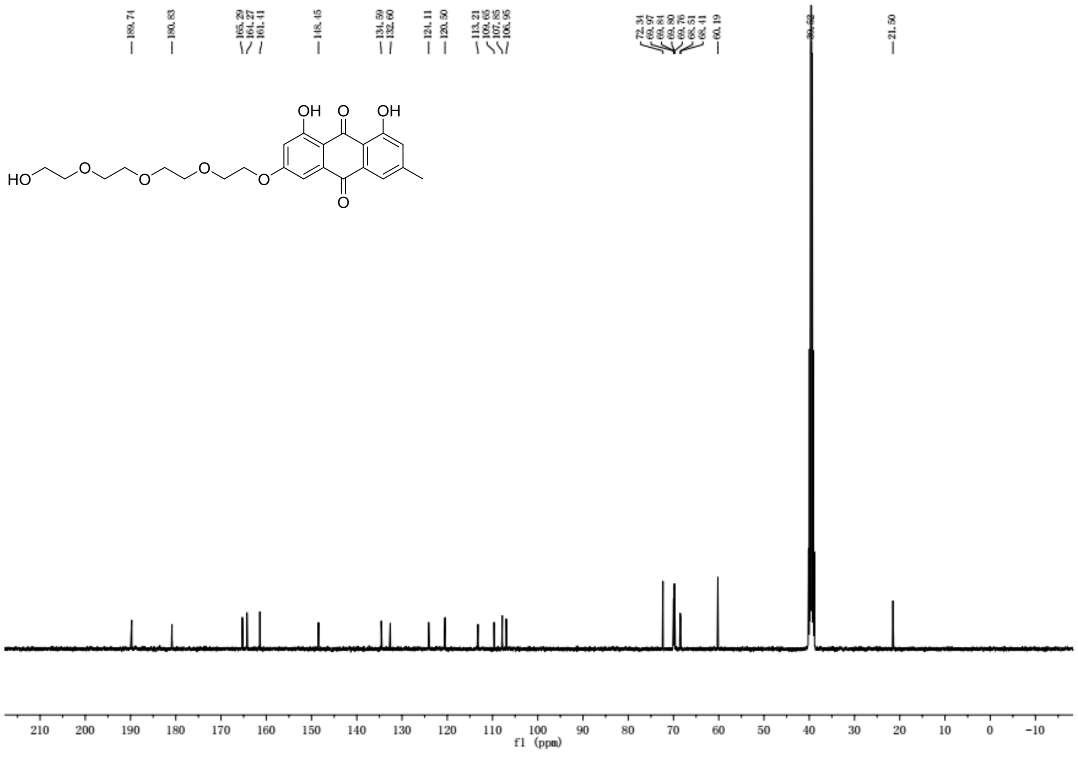

**Figure S104.** $^{13}$C NMR (101 MHz, DMSO–$d_6$) spectrum of compound **6f**.

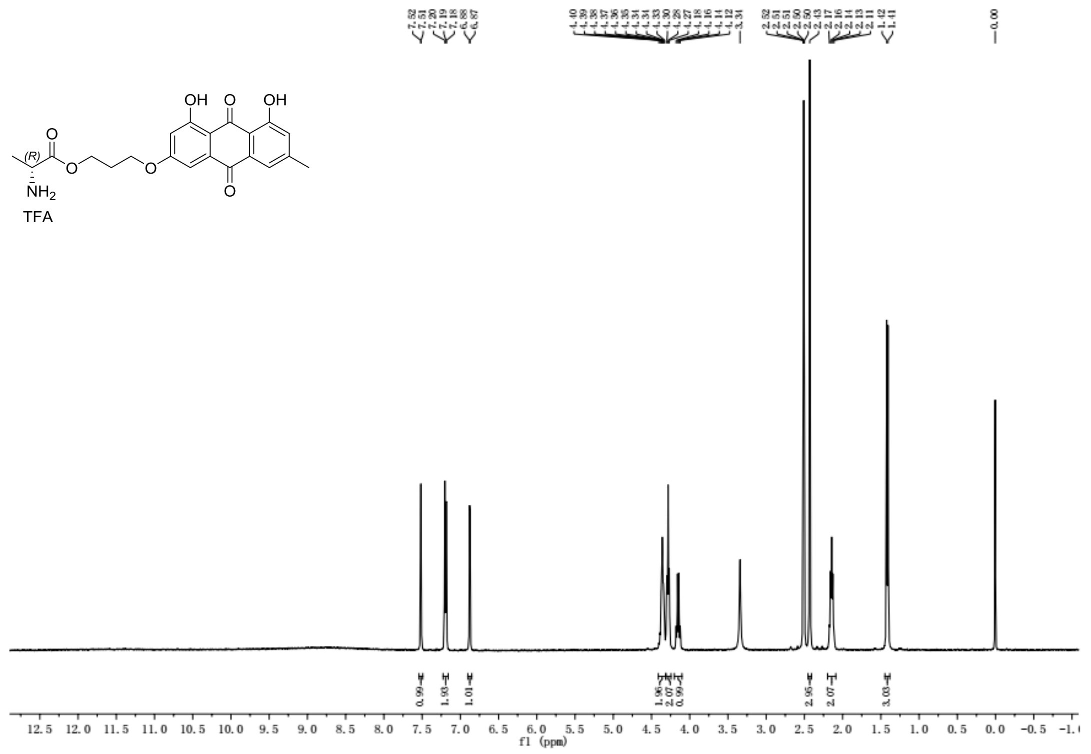


**Figure S105.** $^{1}$H NMR (400 MHz, DMSO–$d_6$) spectrum of compound **7a**.

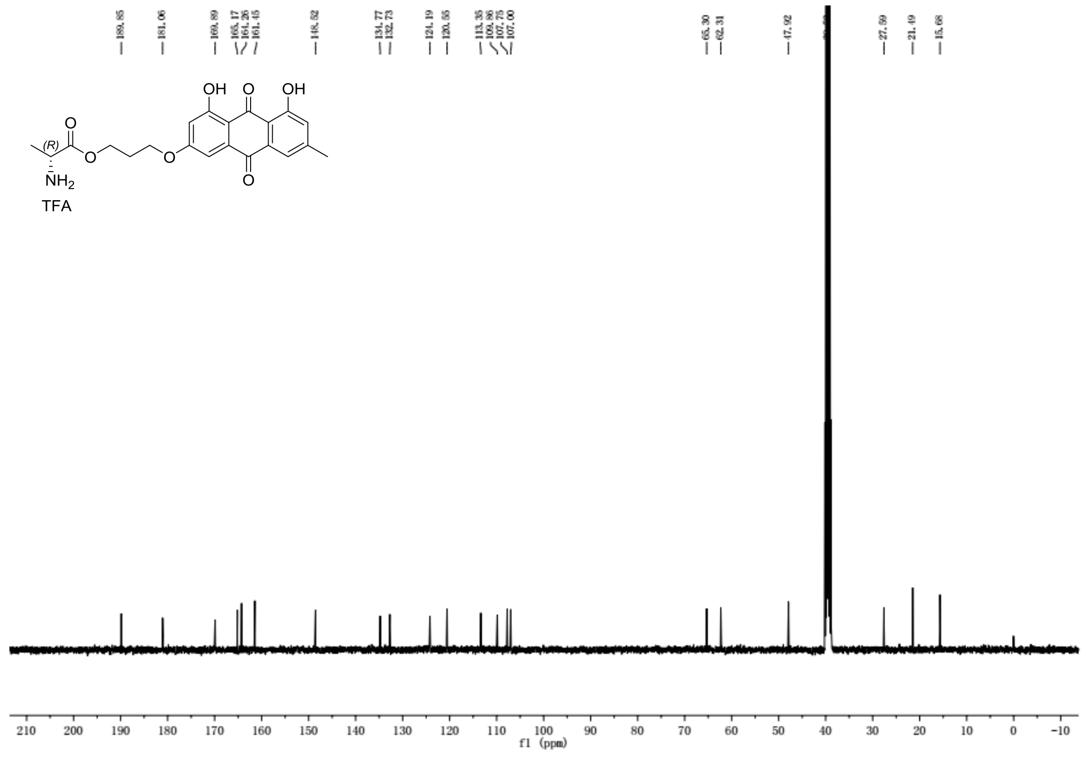


**Figure S106.** $^{13}$C NMR (101 MHz, DMSO–$d_6$) spectrum of compound **7a**.

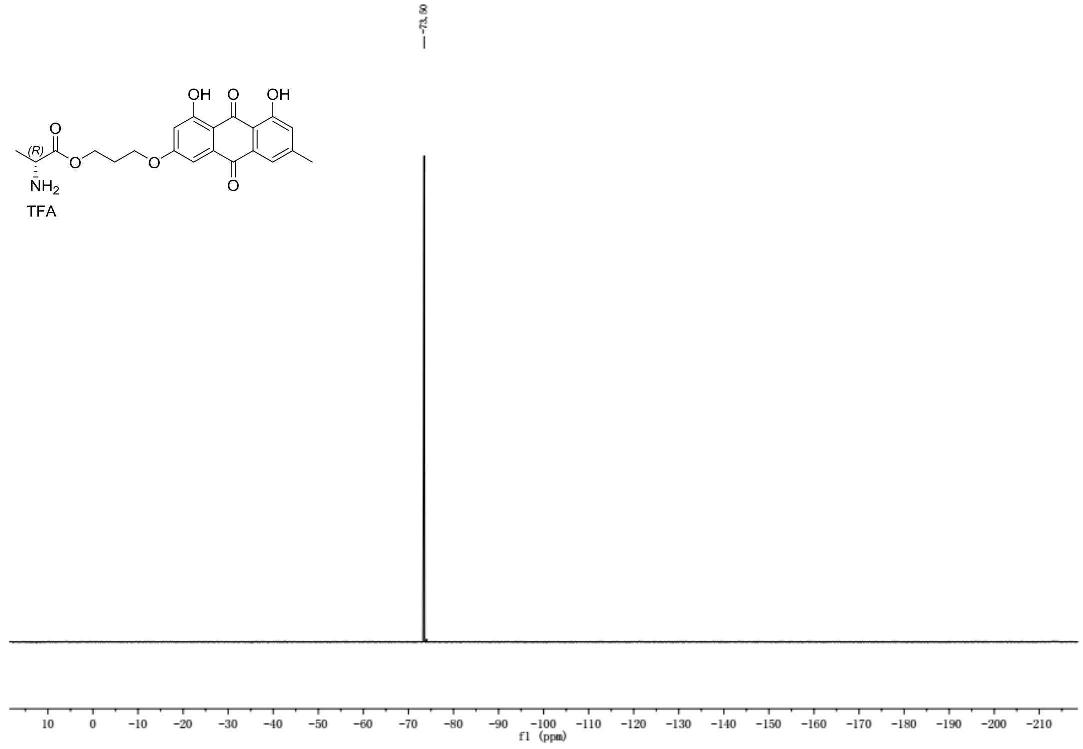

**Figure S107.** $^{19}$F NMR (376 MHz, DMSO–$d_6$) spectrum of compound **7a**.

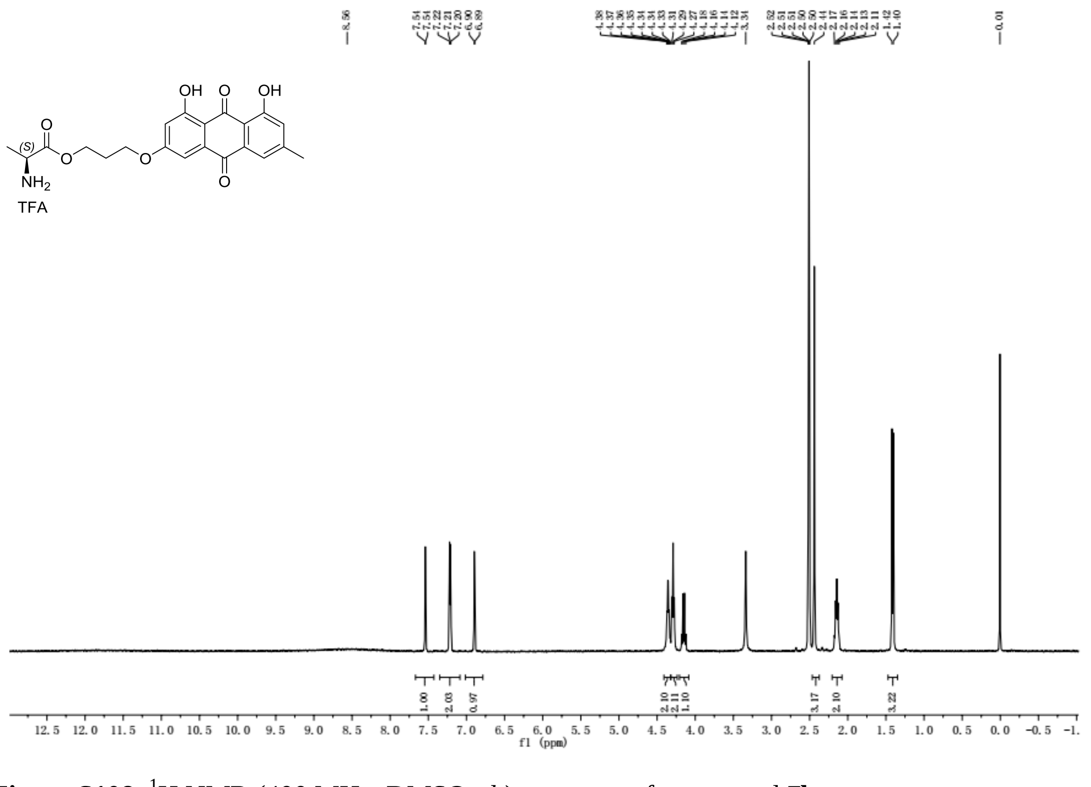

**Figure S108.** $^{1}$H NMR (400 MHz, DMSO–$d_6$) spectrum of compound **7b**.

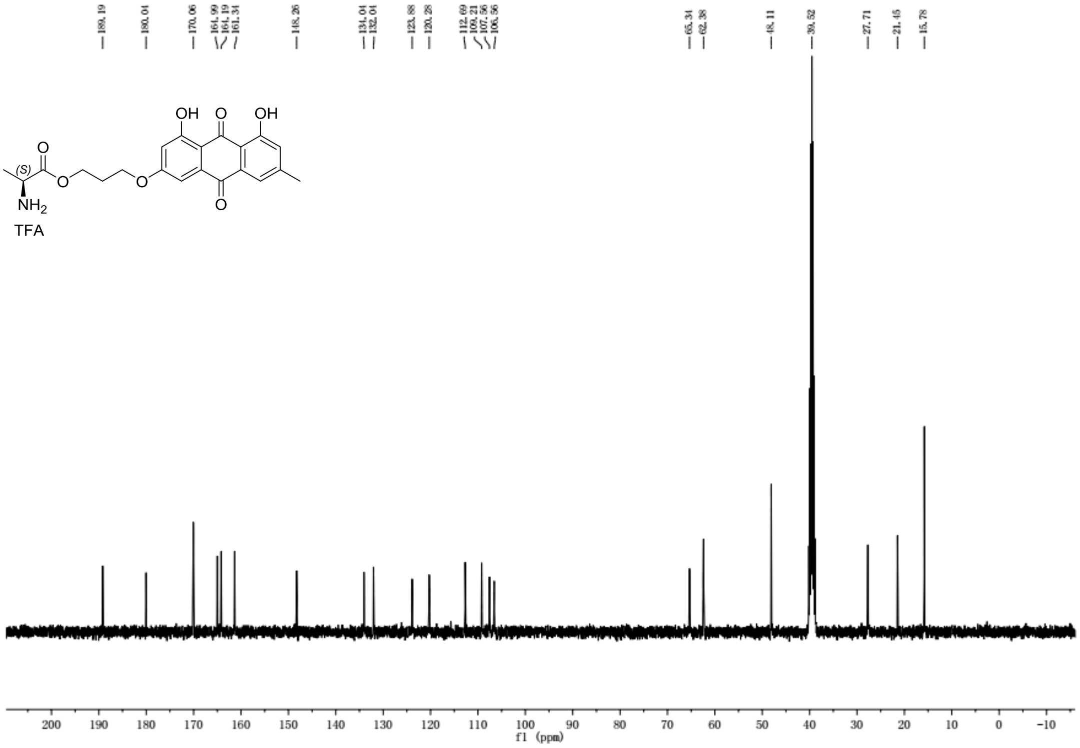

**Figure S109.** $^{13}$C NMR (101 MHz, DMSO–$d_6$) spectrum of compound **7b**.

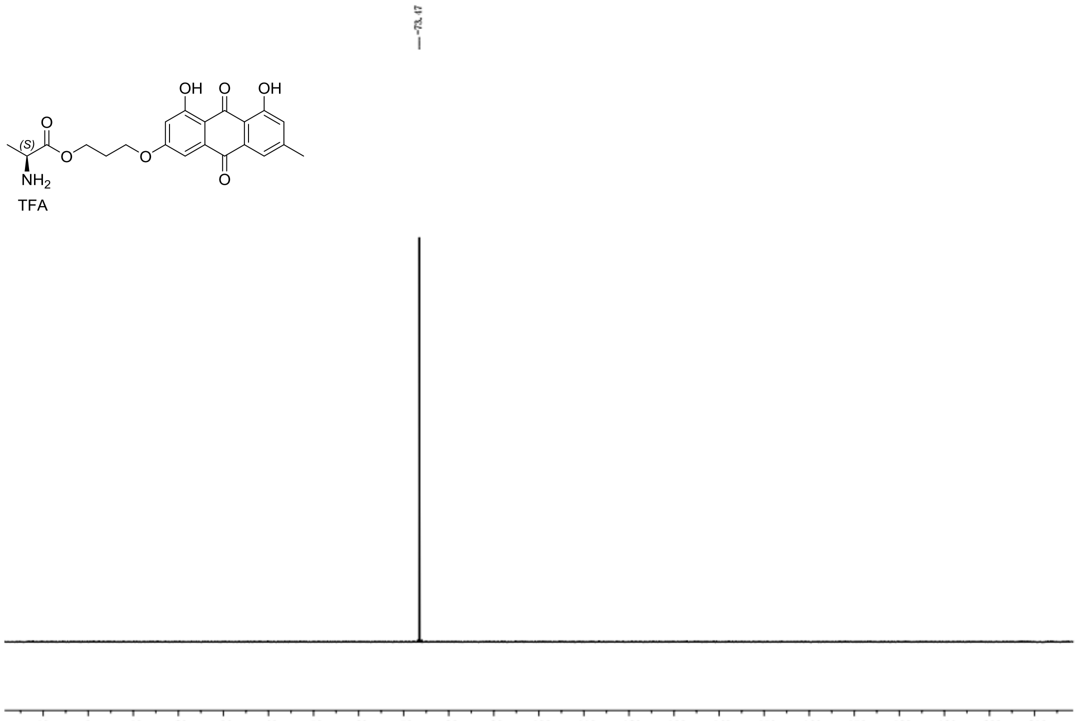

**Figure S110.** $^{19}$F NMR (376 MHz, DMSO–$d_6$) spectrum of compound **7b**.

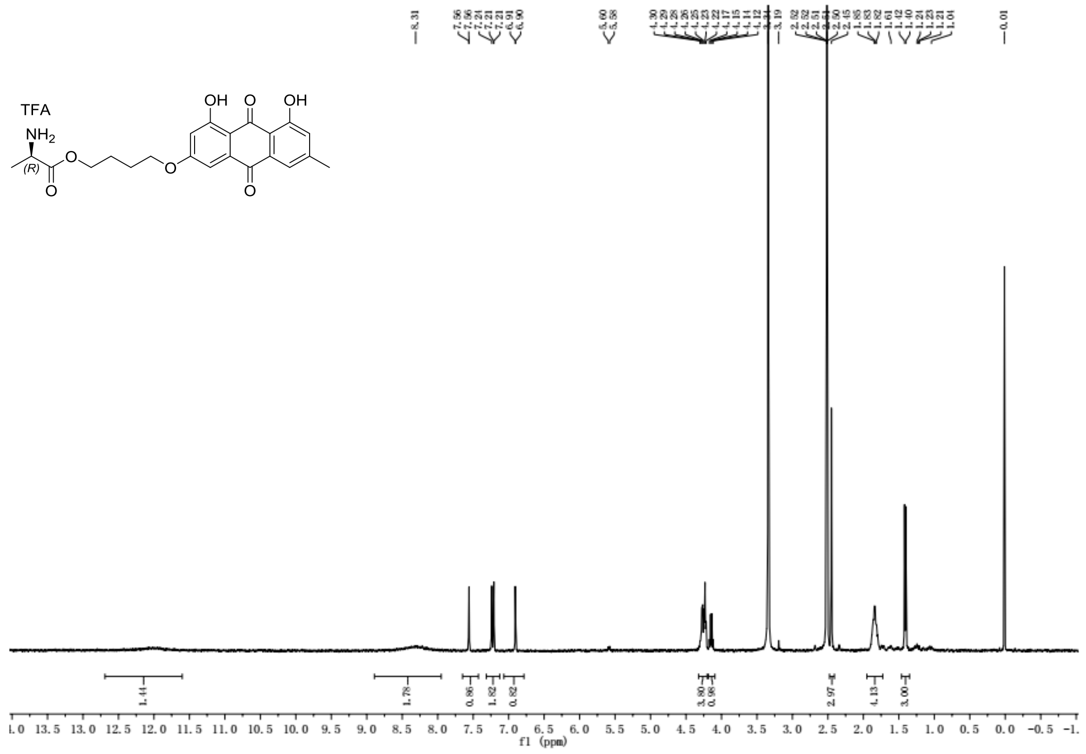


**Figure S111.** $^{1}$H NMR (400 MHz, DMSO–$d_6$) spectrum of compound **7c**.

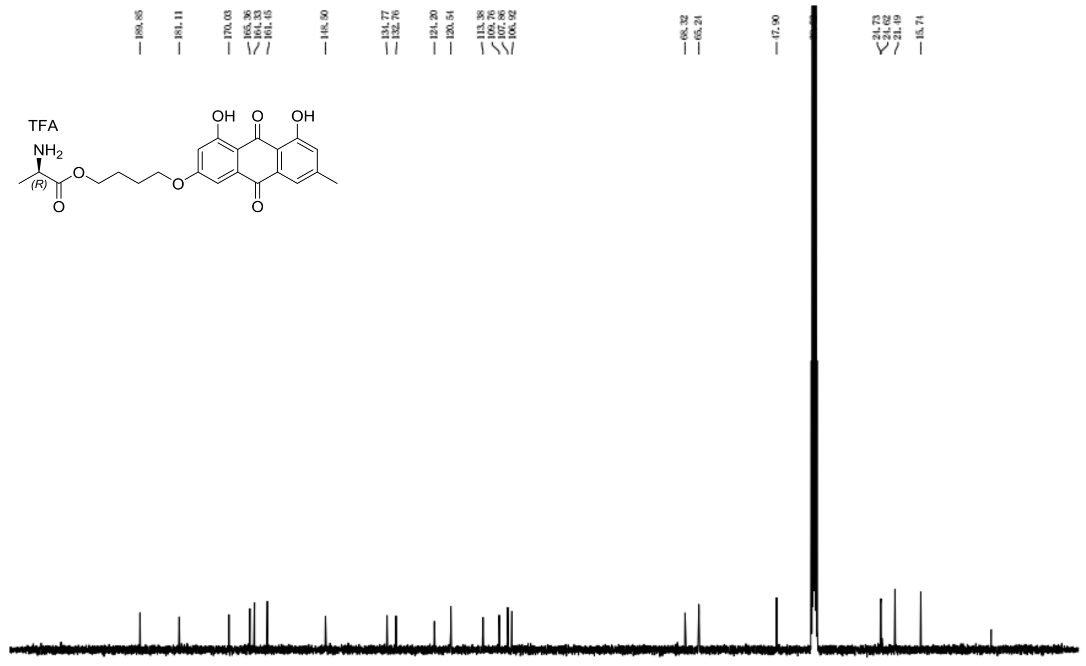


**Figure S112.** $^{13}$C NMR (101 MHz, DMSO–$d_6$) spectrum of compound **7c**.

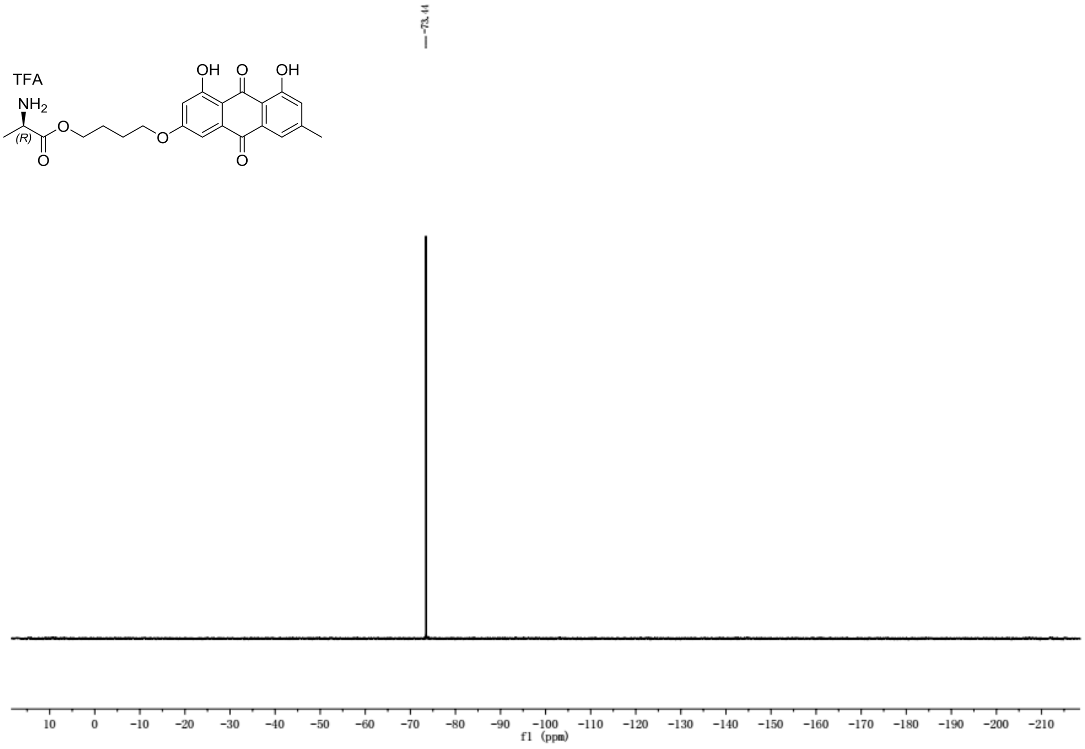

**Figure S113.** $^{19}$F NMR (376 MHz, DMSO–$d_6$) spectrum of compound **7c**.

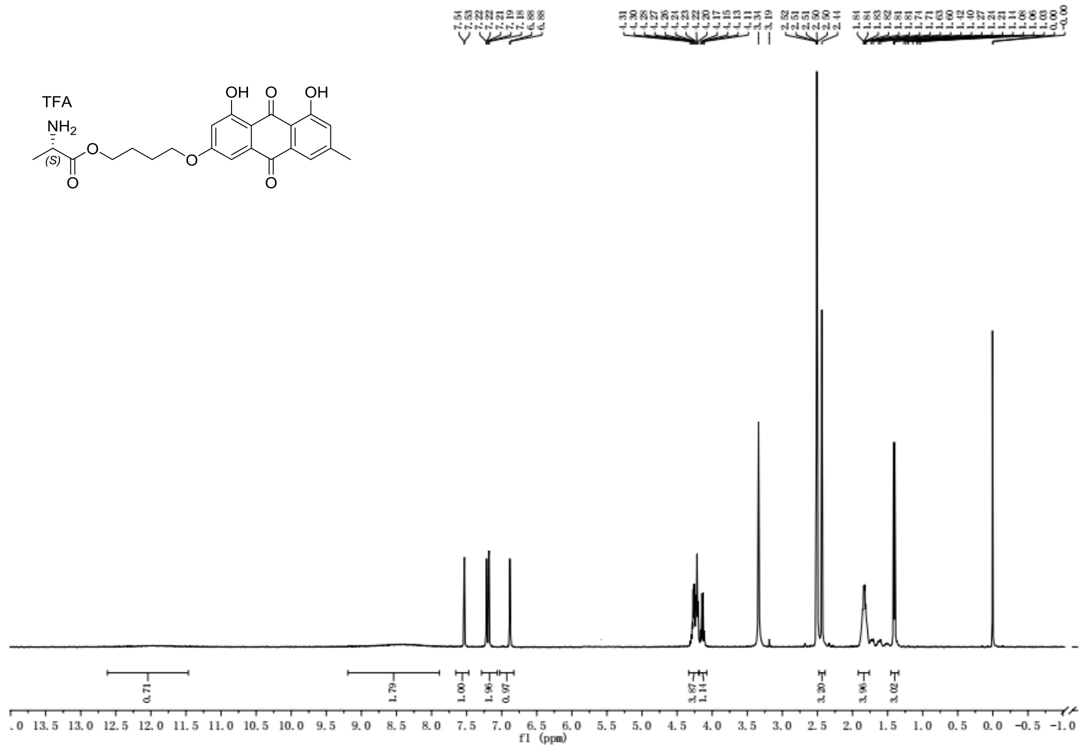

**Figure S114.** $^{1}$H NMR (400 MHz, DMSO–$d_6$) spectrum of compound **7d**.

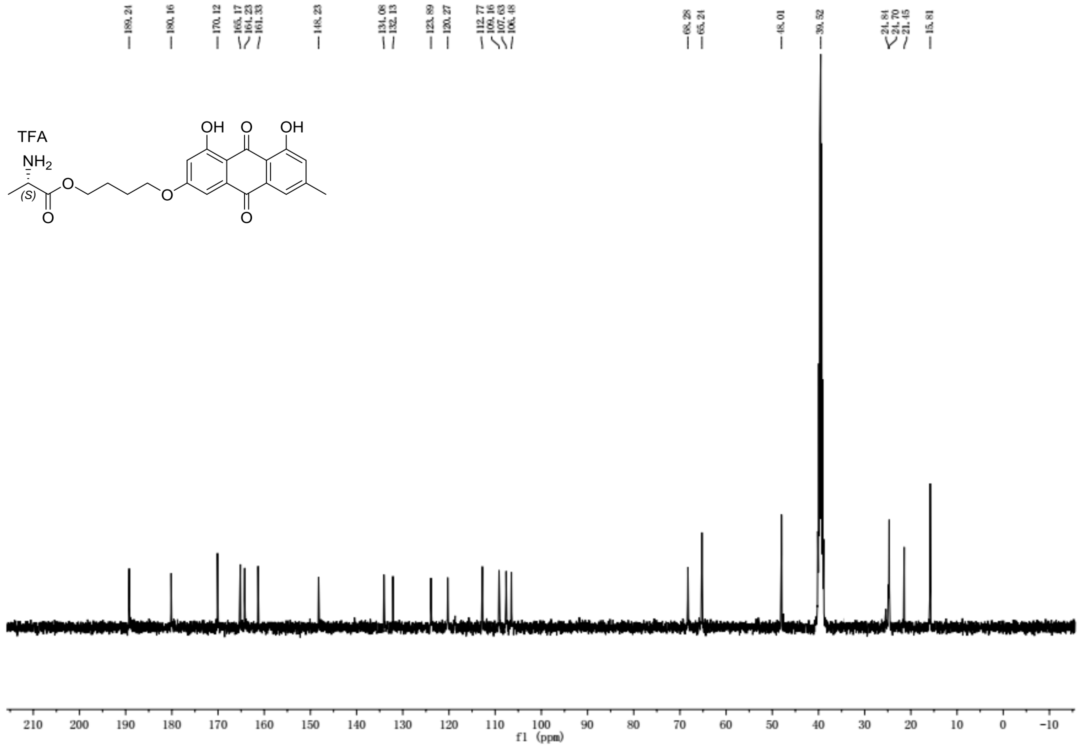


**Figure S115.** $^{13}$C NMR (101 MHz, DMSO–$d_6$) spectrum of compound **7d**.

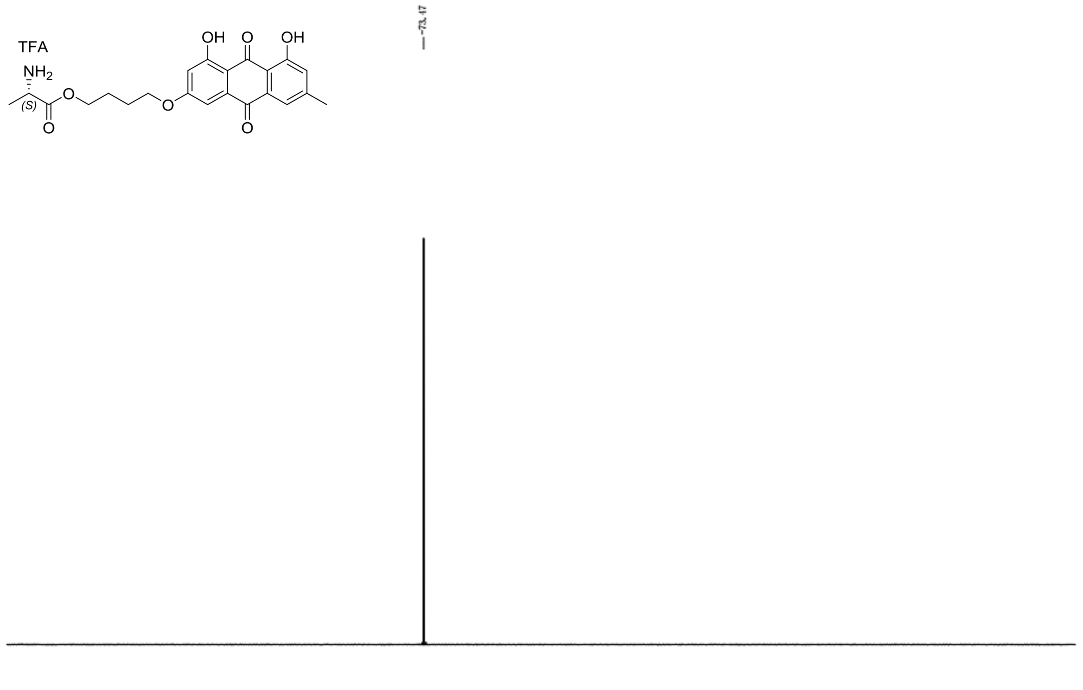


**Figure S116.** $^{19}$F NMR (376 MHz, DMSO–$d_6$) spectrum of compound **7d**.

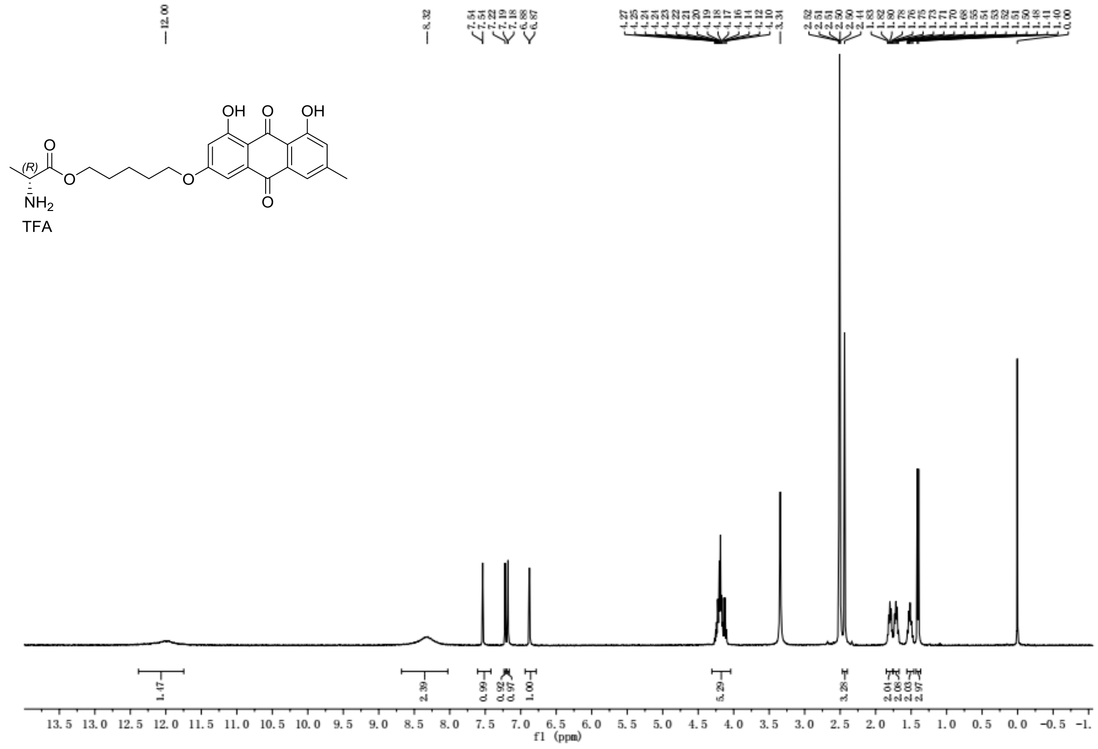

**Figure S117.** $^{1}$H NMR (400 MHz, DMSO–$d_6$) spectrum of compound **7e**.

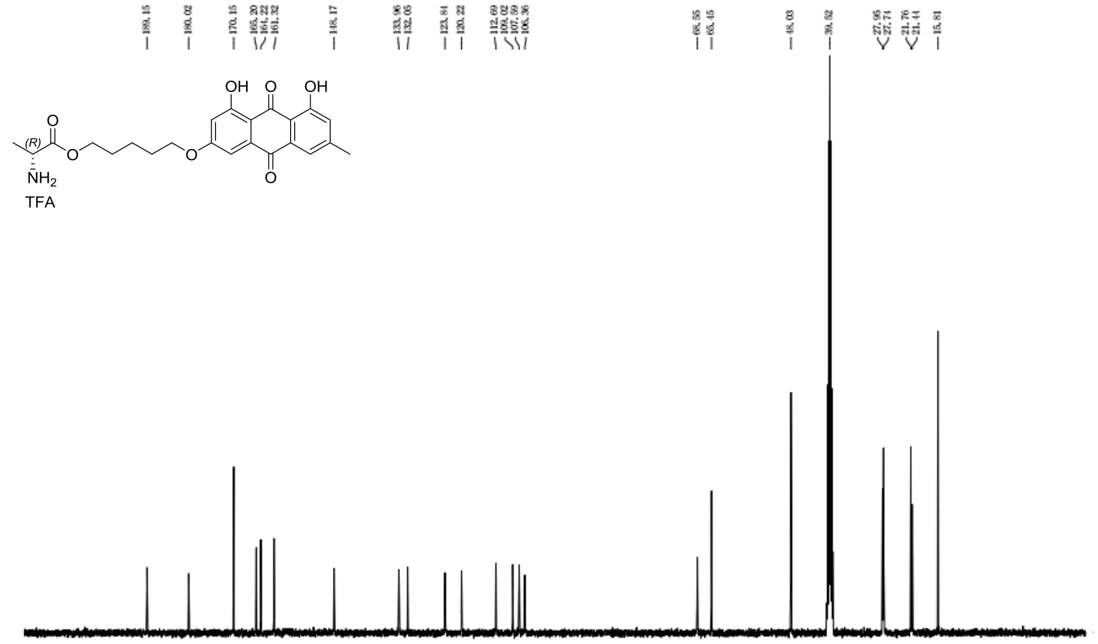

**Figure S118.** $^{13}$C NMR (101 MHz, DMSO–$d_6$) spectrum of compound **7e**.

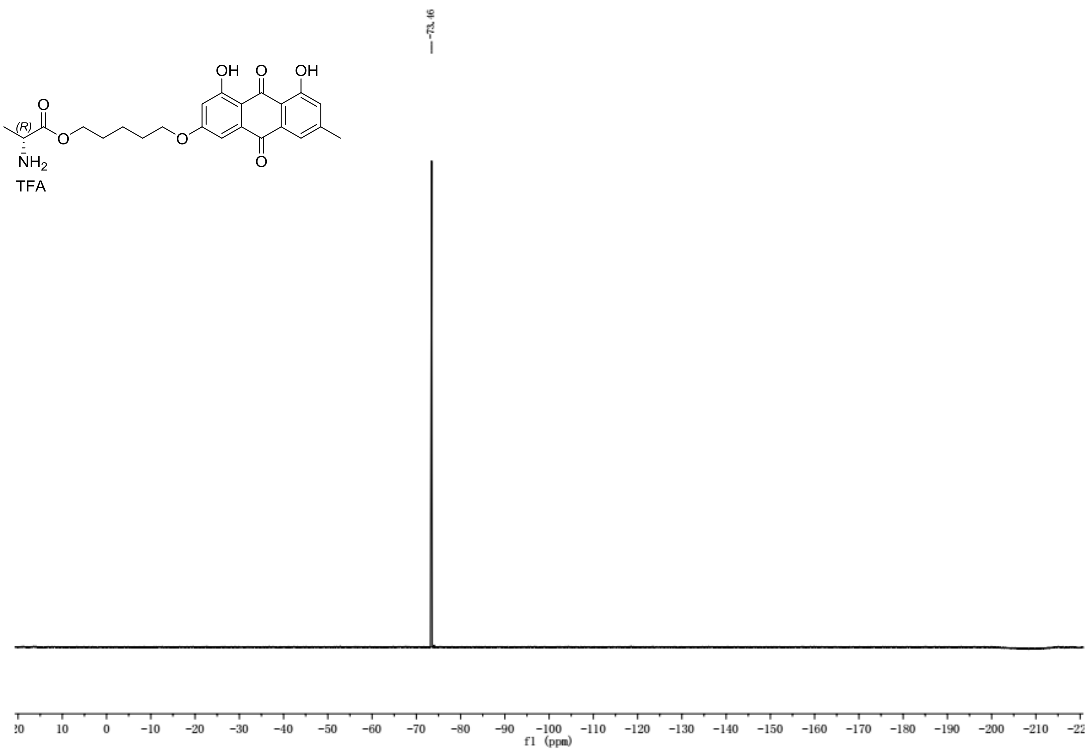

**Figure S119.** $^{19}$F NMR (376 MHz, DMSO–$d_6$) spectrum of compound **7e**.

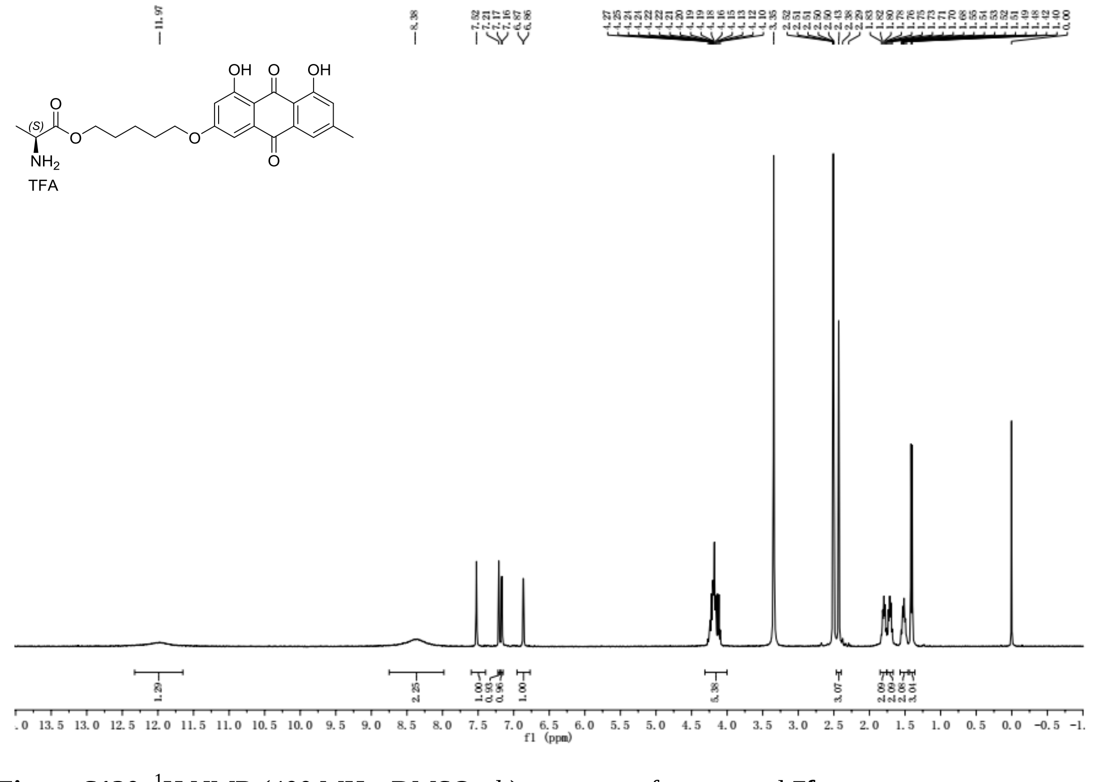

**Figure S120.** $^{1}$H NMR (400 MHz, DMSO–$d_6$) spectrum of compound **7f**.

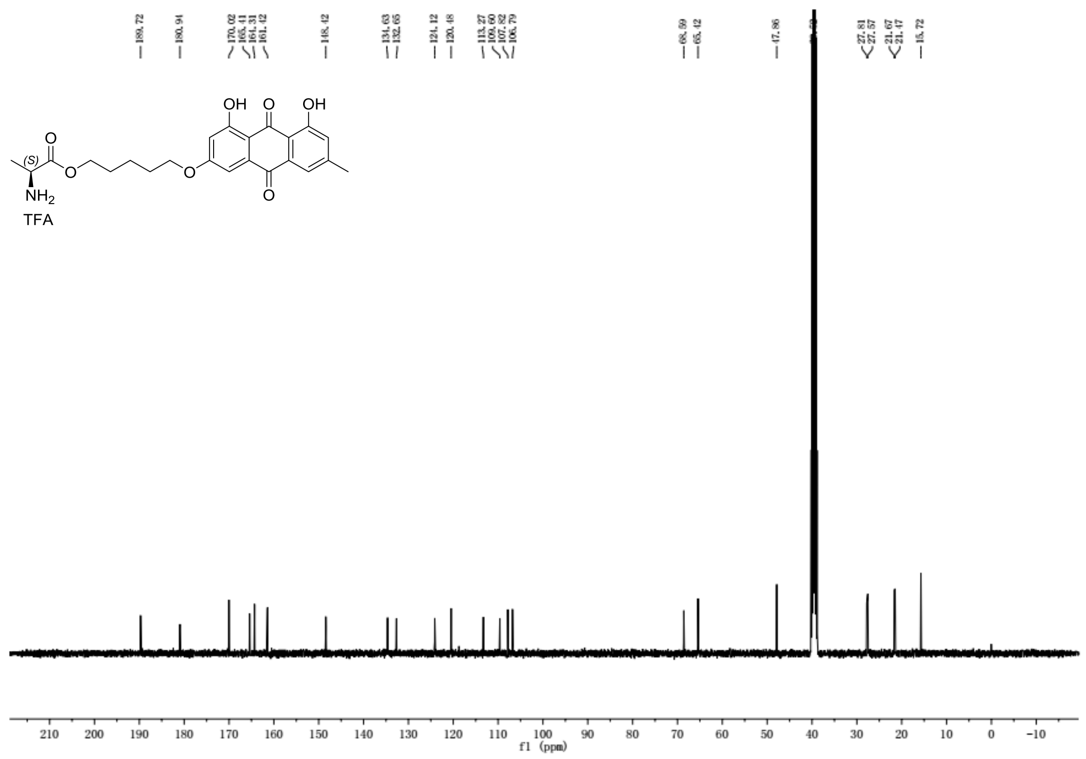

**Figure S121.** $^{13}C$ NMR (101 MHz, DMSO–$d_6$) spectrum of compound **7f**.

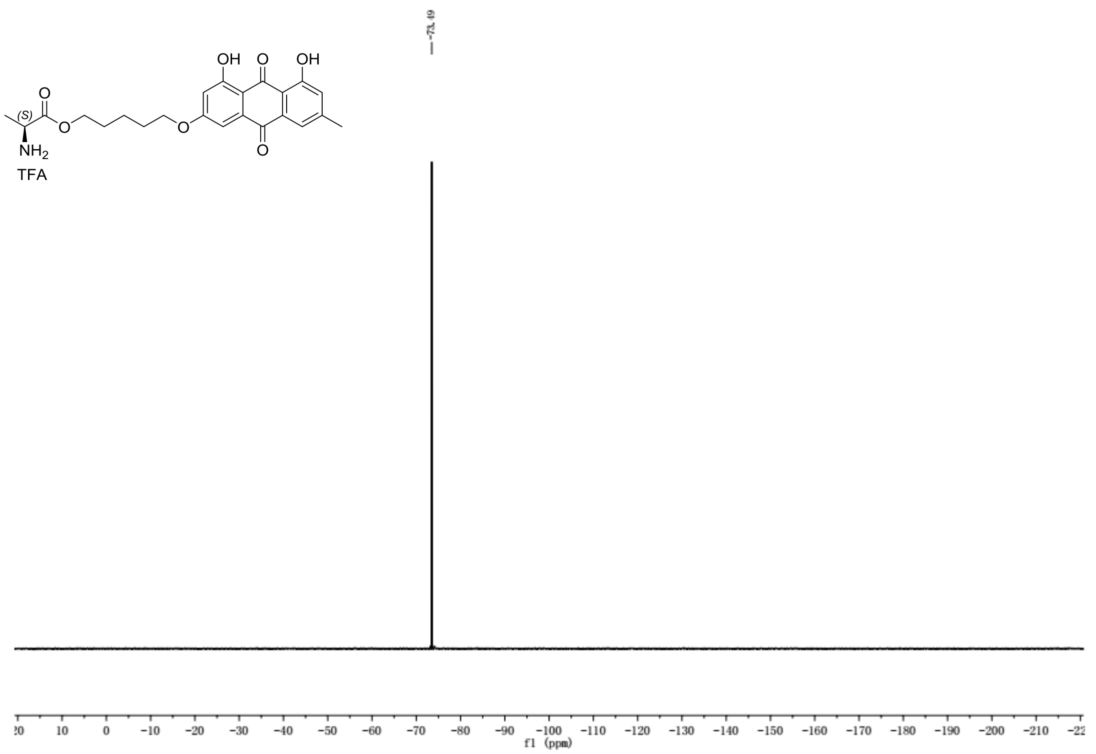

**Figure S122.** $^{19}F$ NMR (376 MHz, DMSO–$d_6$) spectrum of compound **7f**.

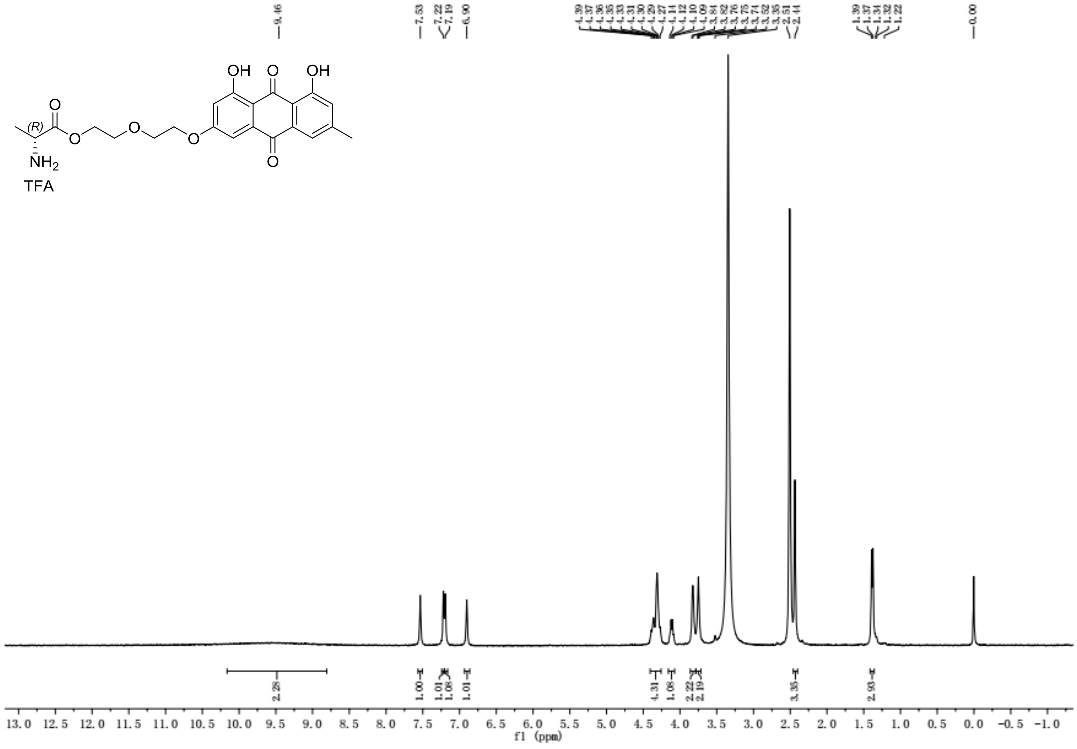

**Figure S123.** $^{1}H$ NMR (400 MHz, DMSO–$d_6$) spectrum of compound **7g**.

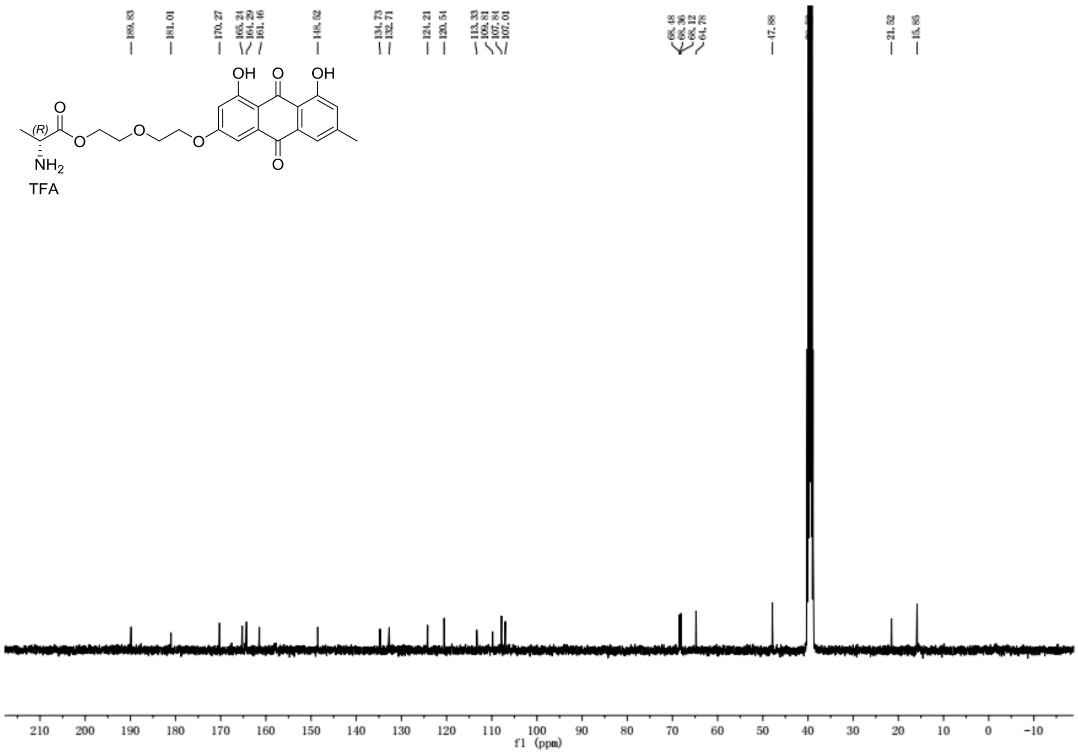

**Figure S124.** $^{13}C$ NMR (101 MHz, DMSO–$d_6$) spectrum of compound **7g**.

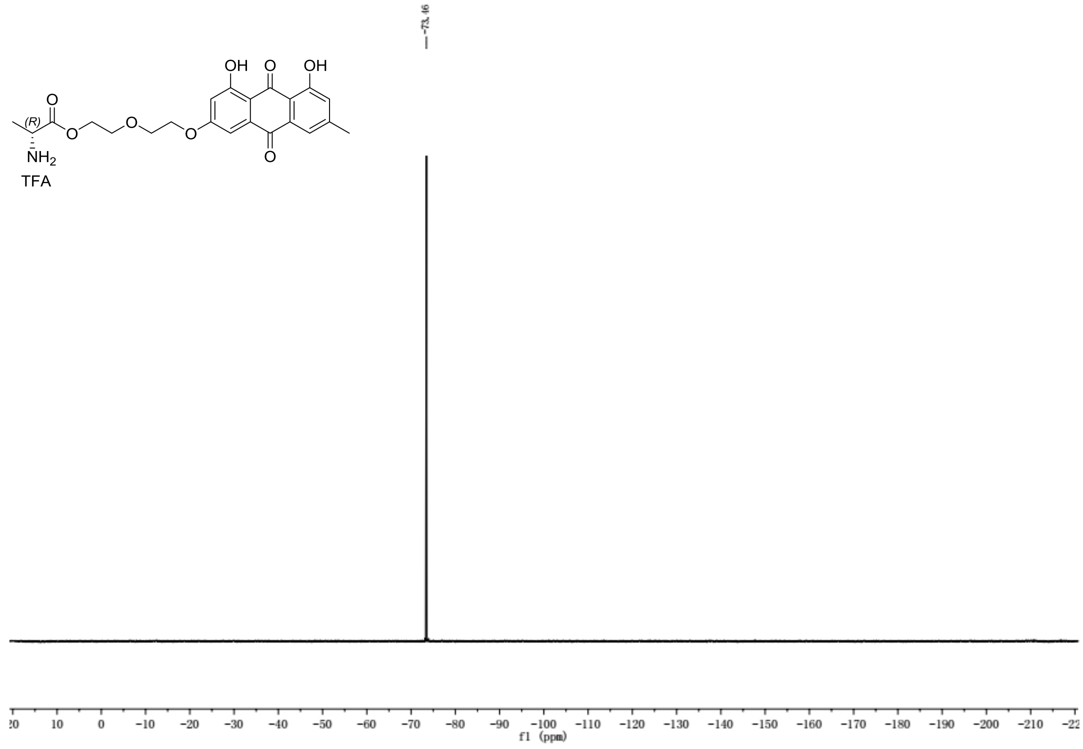

**Figure S125.** $^{19}$F NMR (376 MHz, DMSO–$d_6$) spectrum of compound **7g**.

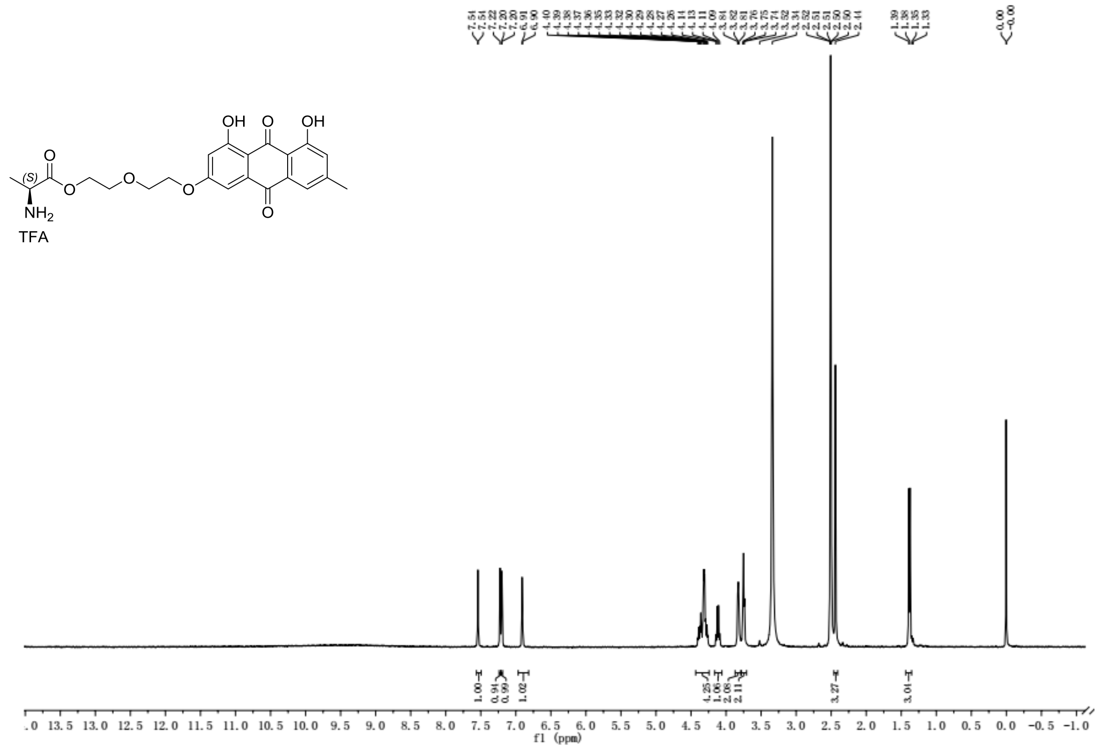

**Figure S126.** $^{1}$H NMR (400 MHz, DMSO–$d_6$) spectrum of compound **7h**.

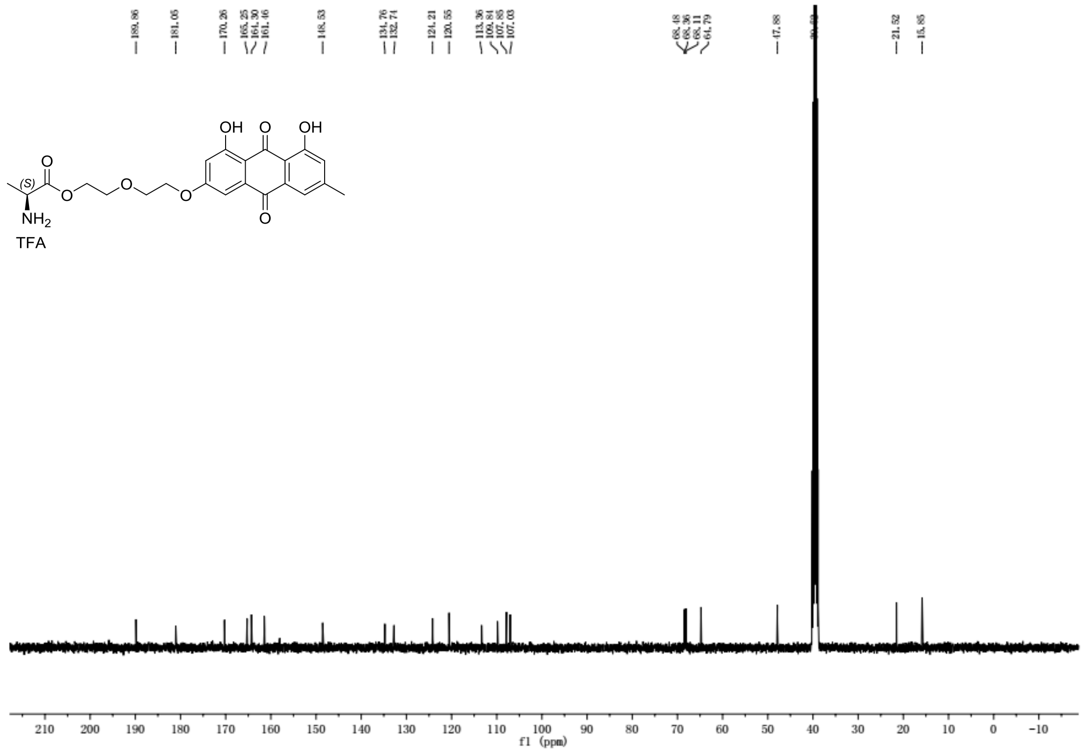


**Figure S127.** $^{13}$C NMR (101 MHz, DMSO–$d_6$) spectrum of compound **7h**.

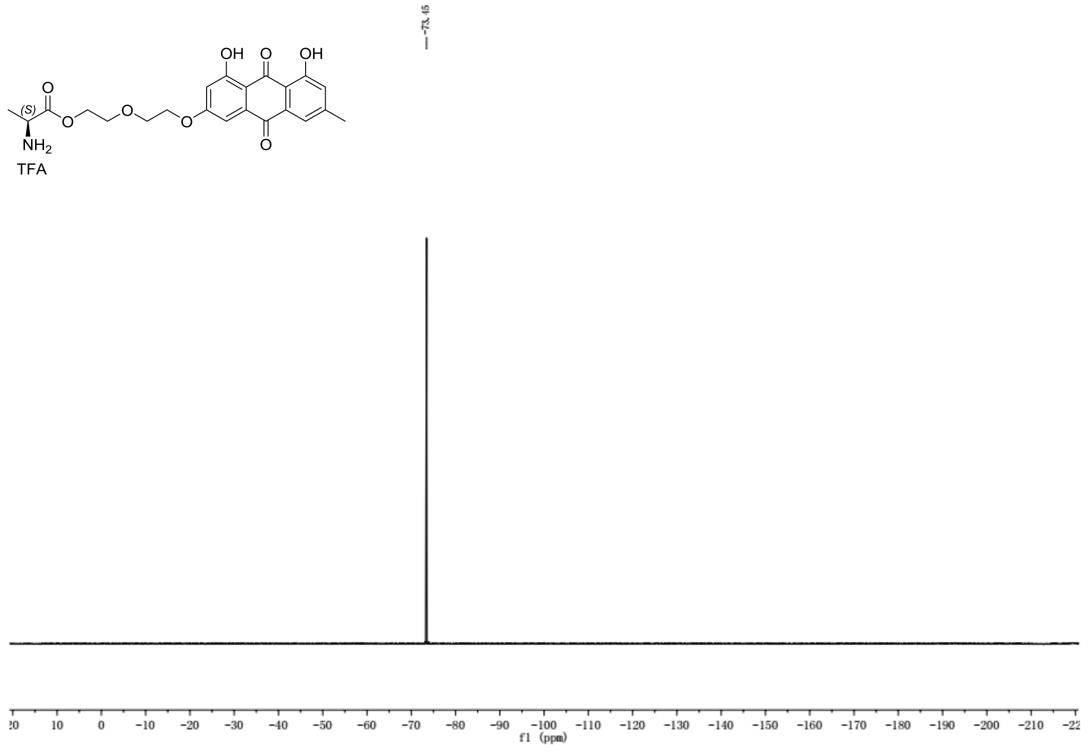


**Figure S128.** $^{19}$F NMR (376 MHz, DMSO–$d_6$) spectrum of compound **7h**.

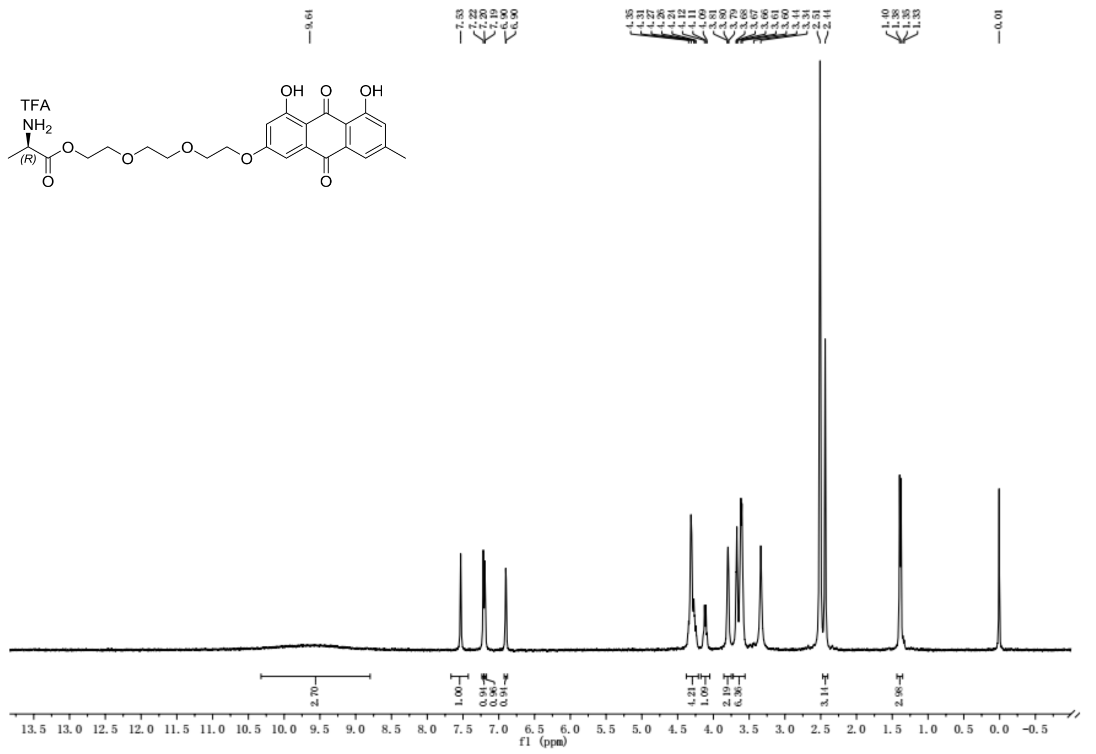

**Figure S129.** $^{1}$H NMR (400 MHz, DMSO–$d_6$) spectrum of compound **7i**.

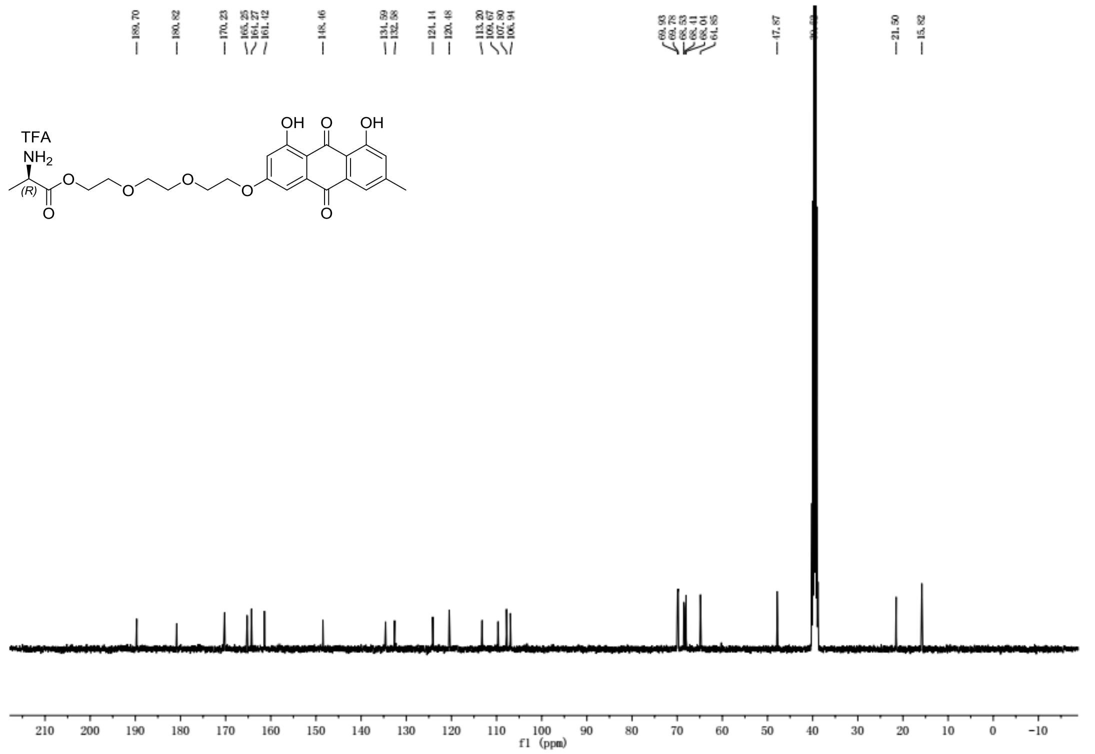

**Figure S130.** $^{13}$C NMR (101 MHz, DMSO–$d_6$) spectrum of compound **7i**.

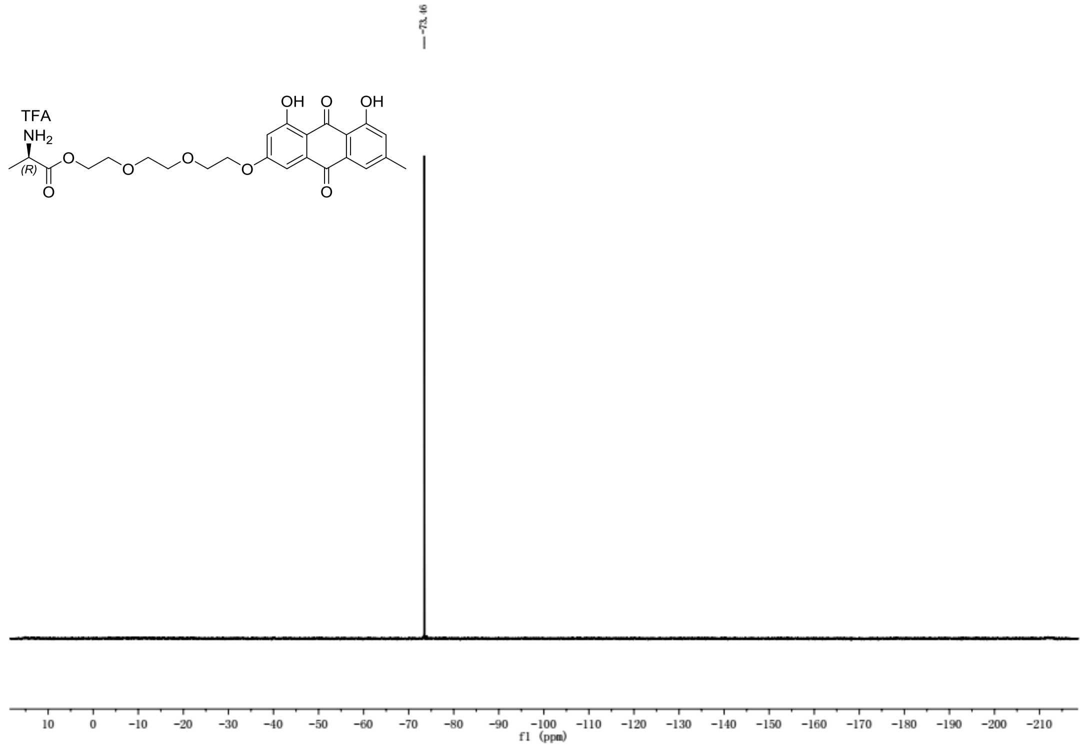


**Figure S131.** $^{19}$F NMR (376 MHz, DMSO–$d_6$) spectrum of compound **7i**.

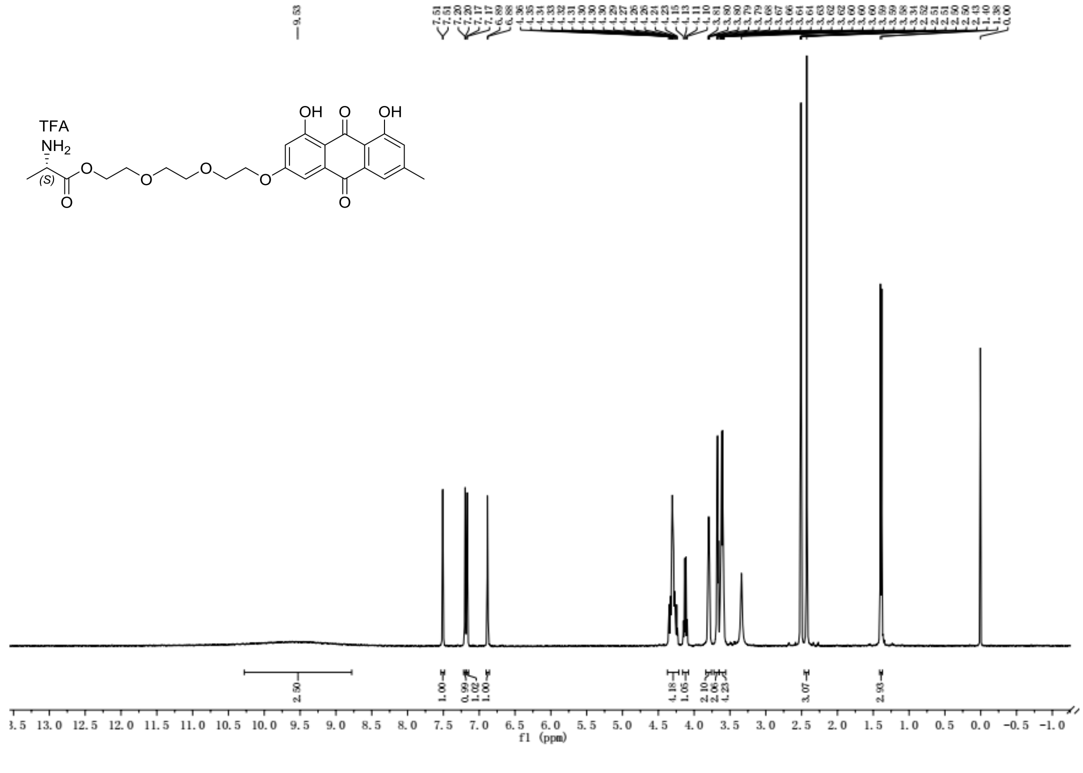


**Figure S132.** $^{1}$H NMR (400 MHz, DMSO–$d_6$) spectrum of compound **7j**.

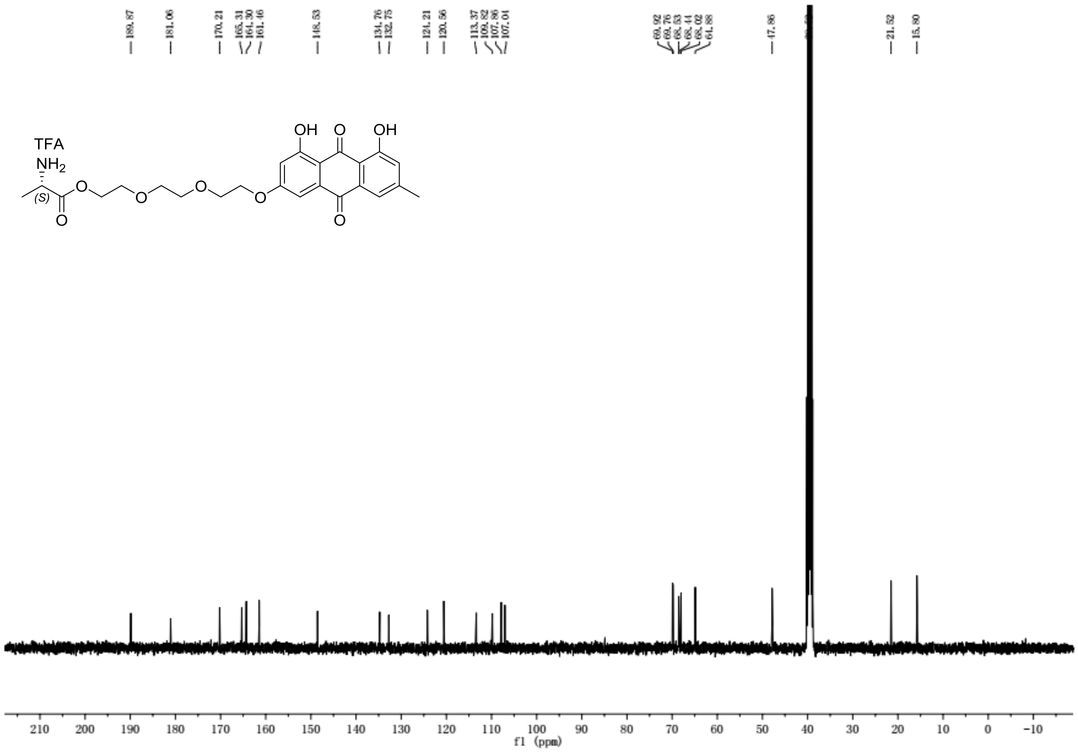

**Figure S133.** $^{13}$C NMR (101 MHz, DMSO–$d_6$) spectrum of compound **7j**.

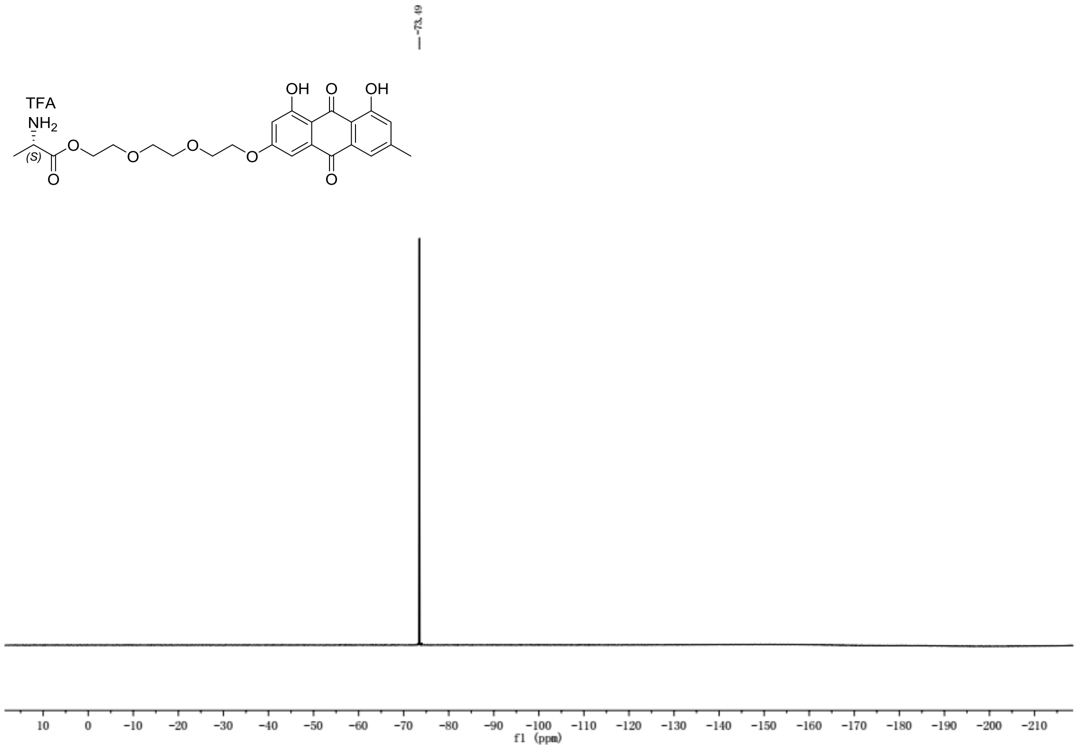

**Figure S134.** $^{19}$F NMR (376 MHz, DMSO–$d_6$) spectrum of compound **7j**.

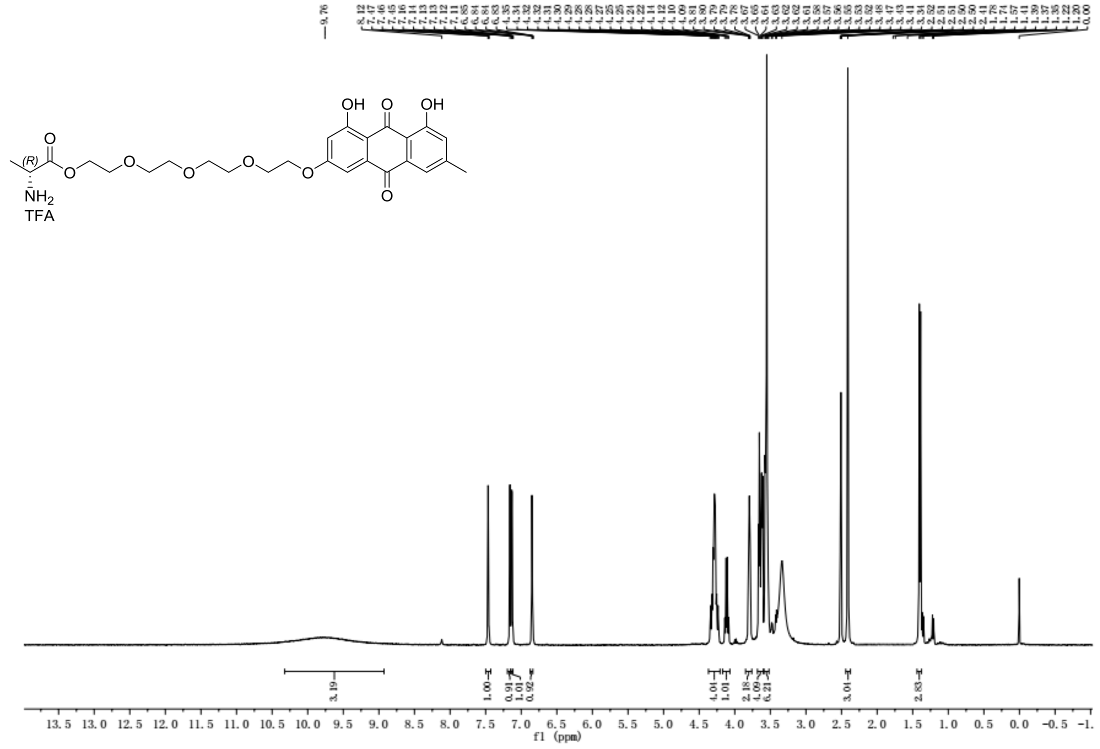

**Figure S135.** $^{1}$H NMR (400 MHz, DMSO–$d_6$) spectrum of compound **7k**.

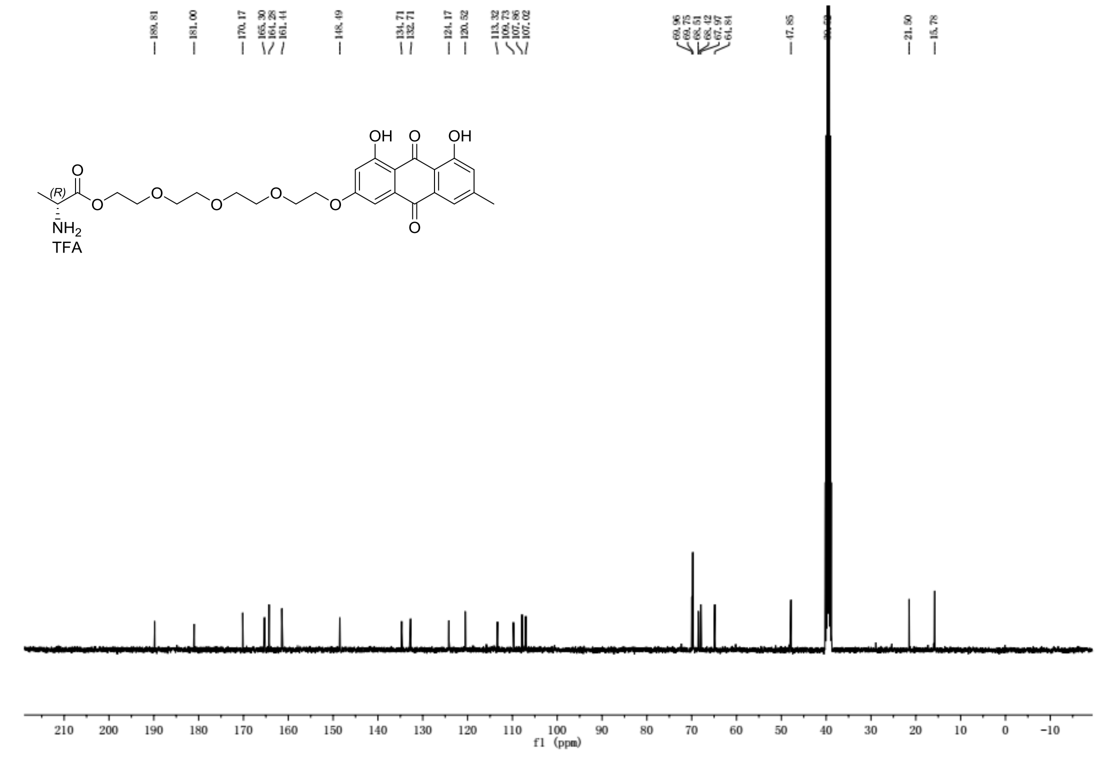

**Figure S136.** $^{13}$C NMR (101 MHz, DMSO–$d_6$) spectrum of compound **7k**.

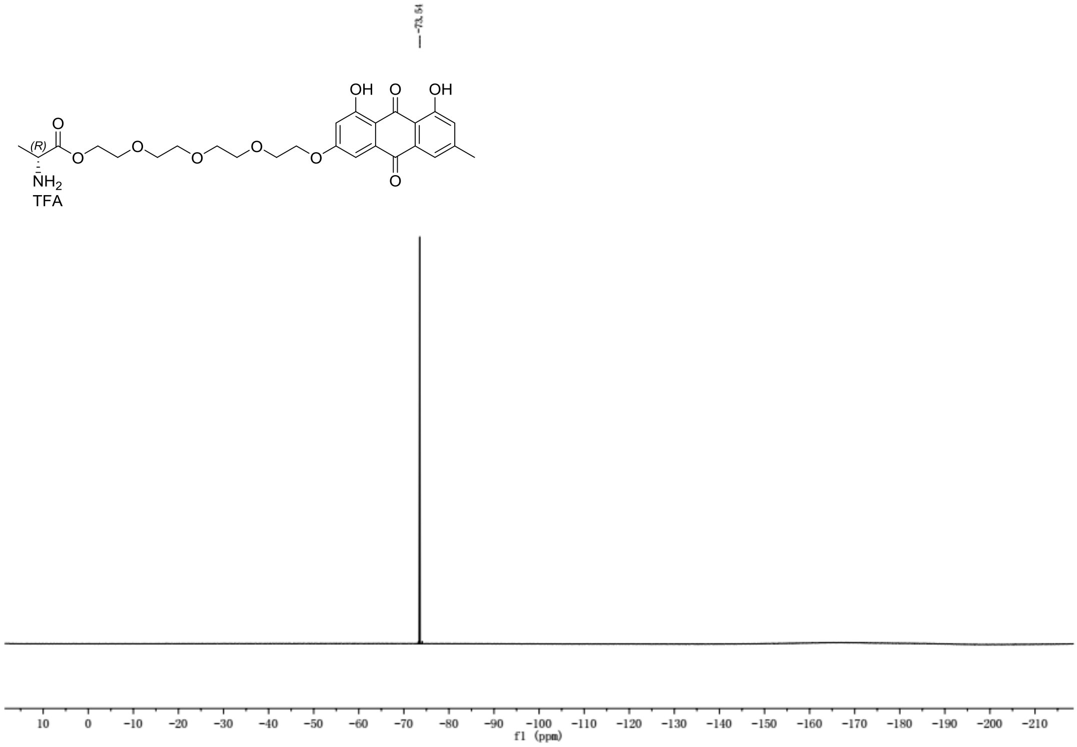


**Figure S137.** $^{19}$F NMR (376 MHz, DMSO–$d_6$) spectrum of compound **7k**.

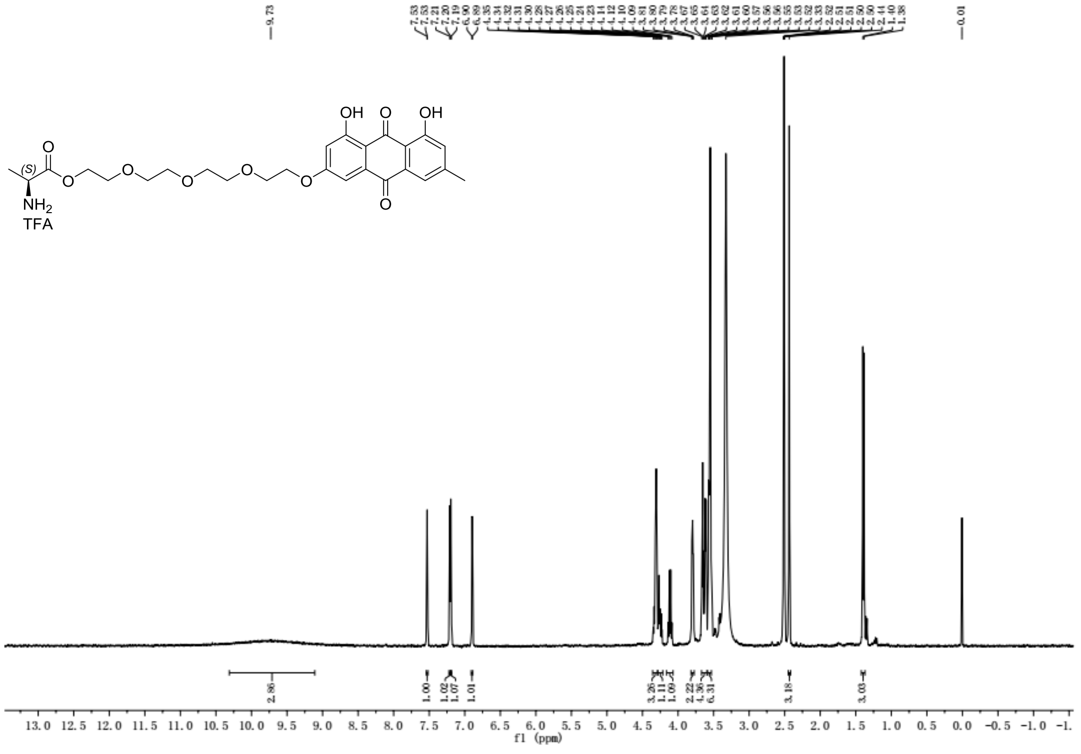


**Figure S138.** $^{1}$H NMR (400 MHz, DMSO–$d_6$) spectrum of compound **7l**.

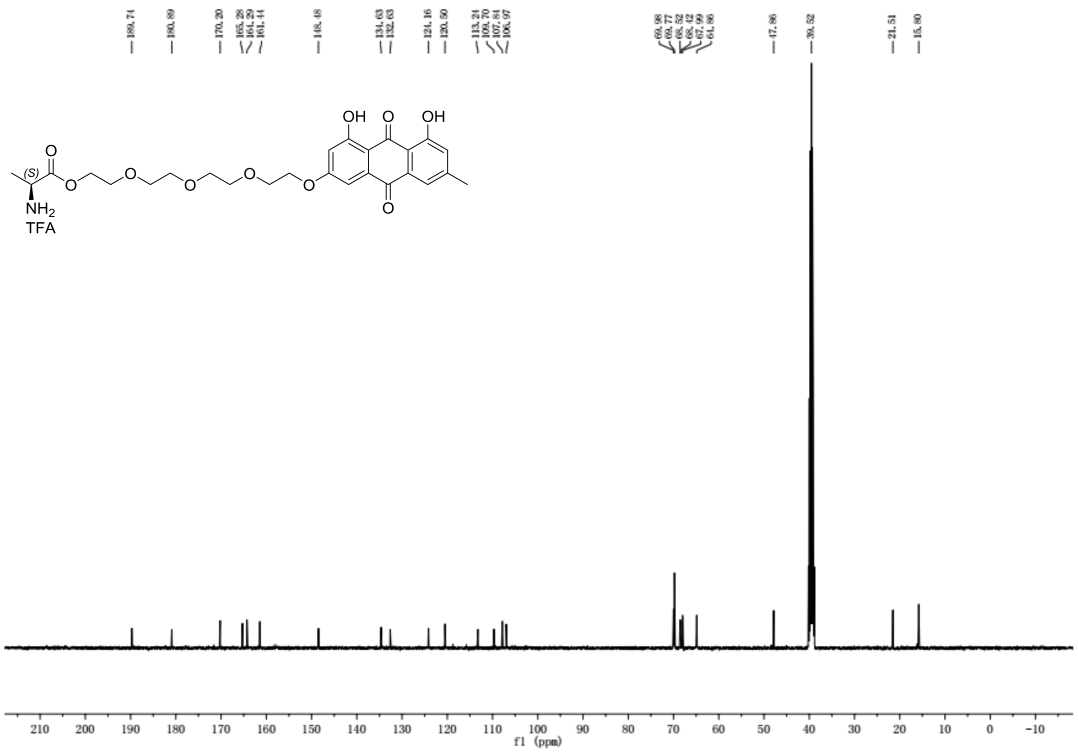

**Figure S139.** $^{13}$C NMR (101 MHz, DMSO–$d_6$) spectrum of compound **7l**.

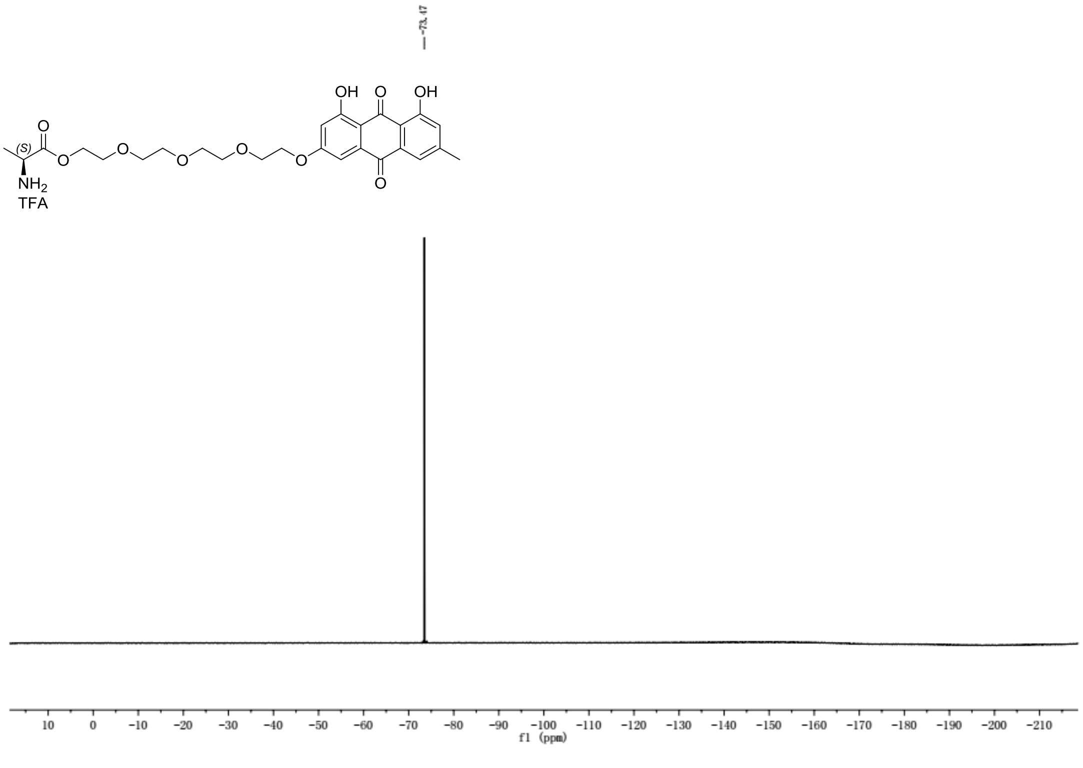

**Figure S140.** $^{19}$F NMR (376 MHz, DMSO–$d_6$) spectrum of compound **7l**.
